# Enantioselective reduction of sulfur-containing cyclic imines through biocatalysis

**DOI:** 10.1038/s41467-018-03841-5

**Published:** 2018-05-16

**Authors:** Nadine Zumbrägel, Christian Merten, Stefan M. Huber, Harald Gröger

**Affiliations:** 1 Chair of Organic Chemistry I, Faculty of Chemistry, Bielefeld University, Universitätsstraße 25, Bielefeld, 33615 Germany; 2 Organic Chemistry II, Faculty of Chemistry and Biochemistry, Ruhr-University Bochum, Universitätsstraße 150, Bochum, 44801 Germany; 3 Organic Chemistry I, Faculty of Chemistry and Biochemistry, Ruhr-University Bochum, Universitätsstraße 150, Bochum, 44801 Germany

## Abstract

The 3-thiazolidine ring represents an important structural motif in life sciences molecules. However, up to now reduction of 3-thiazolines as an attractive approach failed by means of nearly all chemical reduction technologies for imines. Thus, the development of an efficient general and enantioselective synthetic technology giving access to a range of such heterocycles remained a challenge. Here we present a method enabling the reduction of 3-thiazolines with high conversion and high to excellent enantioselectivity (at least 96% and up to 99% enantiomeric excess). This technology is based on the use of imine reductases as catalysts, has a broad substrate range, and is also applied successfully to other sulfur-containing heterocyclic imines such as *2H*-1,4-benzothiazines. Moreover the effiency of this biocatalytic technology platform is demonstrated in an initial process development leading to 99% conversion and 99% enantiomeric excess at a substrate loading of 18 g/L in the presence of designer cells.

## Abstract (web summary)

The 3-thiazolidine ring, a pharmaceutically interesting cyclic structural element found e.g. in some antibiotics, is hard to obtain via currently used approaches. Here, the authors developed a straightforward method to efficiently synthesize a variety of defined, pure 3-thiazolidines.

## Introduction

**Fig. 1:**
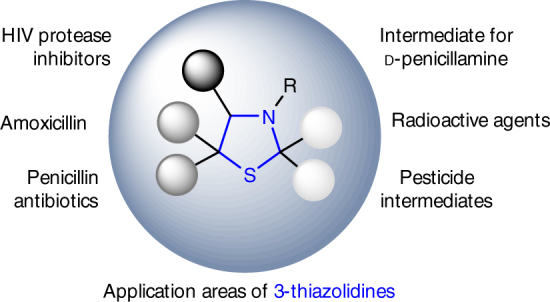
Application areas of 3-thiazolidines. Examples for pharmaceutical relevant structures containing 3-thiazolidines as structural moiety

Cyclic amines with a sulfur-atom in the heterocyclic structure play a distinguished role in nature and medicine. In particular, this is true for the 3-thiazolidine ring, which is one of the two heterocyclic framework structures present in penicillins and penicillin-derived β-lactam antibiotic drugs such as amoxicillin^1^. The 3-thiazolidine moiety is also a structural motif in a range of HIV protease inhibitors^2^ and an industrial key intermediate for the production of the non-proteinogenic amino acid d-penicillamine^3^ (Fig. 1). In addition, *spiro*-type 3-thiazolidines showed activity in human tumor cell lines^4^, and 3-thiazolidines with a less functionalized, alkyl-substitution pattern are of pharmaceutical interest with applications, e.g., as radioprotective agents^5,6^. Besides medicinal purpose, alkyl-functionalized 3-thiazolidines are reported to be relevant for the fields of pesticides^7^ and flavors^8^ (Fig. 1). Due to the importance of such molecules and the lack of efficient general synthetic approaches, the search for attractive synthetic routes towards 3-thiazolidines (and other pharmaceutically interesting sulfur-containing cyclic amines such as 3,4-dihydro-2*H*-1,4-benzothiazines^9^) is still ongoing. The development of a general and enantioselective synthetic platform technology giving access to a broad range of such heterocycles independent of a specific substitution pattern, thus enabling the design of libraries of such heterocycles, as well as efficient processes for their production, would be particularly desirable.

Conceptually a substitution pattern independent access towards 3-thiazolidines **2** can be proposed to proceed through reduction of the C=N double bond in the corresponding 3-thiazolines (**1**), which can be easily prepared through Asinger-type multicomponent reaction^10–13^. However, in spite of the availability of such attractive substrates, in all research work since the 1950s the reduction of 3-thiazolines (**1**) failed by means of nearly all state of the art-type chemical reduction technologies for imines. Such non-successful attempts include numerous typical C = N double bond reduction technologies, which are known to work well for many other imine substrate types. For example, the established hydrogenation technology (Pd/C, H_2_) was found at an early stage not to be suitable due to catalyst poisoning by sulfur^13,14^. These results are in accordance with later results by the Figueras group, finding that metals of supported metal catalysts become poisoned by sulfur^15^. The recently developed organocatalytic reduction with Hantzsch esters for imines^16,17^ also failed to lead the desired transformation due to lack of reactivity (Supplementary Figs. 1, 2, Supplementary Table 1 and Supplementary Methods). Other well-established reduction methods, such as sodium in alcohol, sodium in liquid ammonia or aluminum in the presence of potassium hydroxide or wet ether did not give clear results^13^. When using metal hydrids, such as NaBH_4_ and LiAlH_4_, reduction works but is accompanied with an undesired ring-opening of the *N*,*S*-acetal moiety in the product **2** (Supplementary Fig. 2 and Supplementary Methods)^13,14,18^. The only exception is a catecholborane-type reduction^18^, but in this case the yield was low to moderate and enantioselectivity turned out to be poor with only up to 4% enantiomeric excess (ee). In addition, a high-catalyst loading, as well as an expensive and technically less favored reducing agent (due to high flammability) is needed. Thus, the development of an efficient general method for the reduction of 3-thiazolines (**1**) remained a challenge, as did establishing an asymmetric catalytic version of this reaction. Attracted by the recent successful use of imine reductases (IREDs) for various reductions of cyclic imines^19–30^, we became interested in studying the suitability of this biocatalytic methodology for the reduction of 3-thiazolines (**1**) and other sulfur-containing cyclic imines such as 2*H*-1,4-benzothiazines (**3**).

Here we report such a biocatalytic reduction which firstly enables a smooth reduction of 3-thiazolines (**1**) avoiding undesired ring-opening or other side reactions, secondly allows a highly enantioselective synthesis of the resulting 3-thiazolidines (**2**) by means of such an approach starting from 3-thiazolines (**1**), and thirdly is a broadly applicable reduction platform for sulfur-containing heterocyclic imines including 3-thiazolines (**1**), as well as other sulfur-containing heterocyclic imines such as 2*H*-1,4-benzothiazines (**3**).

## Results

### Proof of concept for 3-thiazoline reduction

**Fig. 2:**
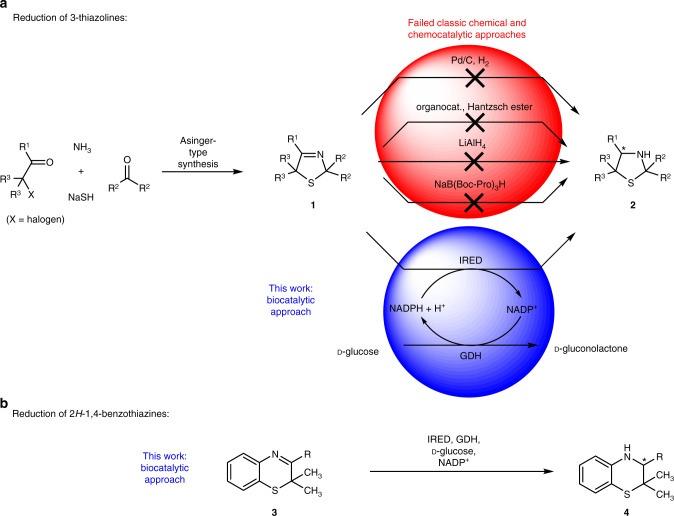
Concept of this work. **a** Access toward 3-thiazolines via Asinger-type synthesis and subsequent reduction to 3-thiazolidines. Whereas nearly all classic chemical and chemocatalytic approaches toward reduction of 3-thiazolines failed, reduction with imine reductases provided 3-thiazolidines in high conversion and enantioselectivity. **b** The biocatalytic approach using imine reductases was also successfully applied for the reduction of other sulfur-containing heterocyclic imines, such as 2*H*-1,4-benzothiazines

**Fig. 3:**
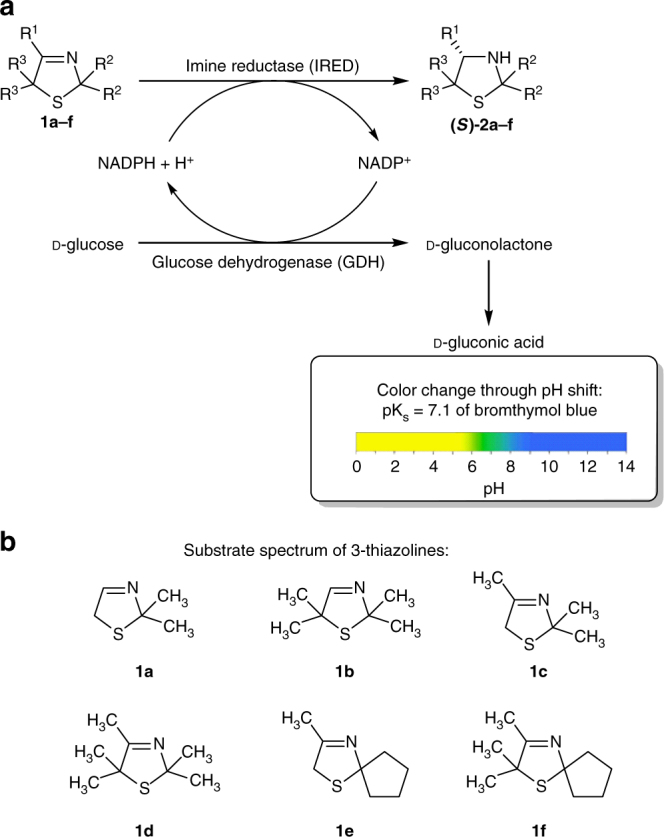
Colorimetric pH shift assay of 3-thiazolines. **a** Concept of colorimetric pH shift assay. Reduction of substrate catalyzed by imine reductase (IRED) and in situ-cofactor-regeneration by glucose dehydrogenase (GDH) results in a pH shift, due to formation of gluconic acid. This pH shift is visualized by bromthymol blue as indicator. **b** Substrate spectrum of 3-thiazolines examined in this work

In initial experiments, we tested whether IREDs are suitable biocatalysts for the reduction of 3-thiazolines (Fig. 2). Although unknown for cyclic imines with additional heteroatoms, we focused on this enzyme class instead of the so-called thiazolinyl IREDs since the latter enzymes (although bearing the term thiazolinyl imine in their name) are known to be substrate-specific for structurally highly functionalized 2-thiazolines reducing an imidothioester moiety^31,32^, thus representing no promising option for developing a broadly applicable synthetic platform for the reduction of the imine bond of 3-thiazolines. Toward this end, we prepared two non-prochiral 3-thiazolines (**1a** and **1b**) and screened them against a set of 31 IREDs, which had been prepared in a recombinant form by means of overexpression in *E*. *coli* BL21 (DE3)^22,29^. As a fast screening methodology a colorimetric pH shift assay^33^ developed by the Sieber group was used. This assay is based on a pH shift (visualized by bromthymol blue), which is caused by gluconic acid formed by consumption of the substrate and in situ-cofactor-regeneration (Fig. 3, Supplementary Figs. 3, 4 and Supplementary Methods).

Color changes from green to yellow were observed for both non-prochiral 3-thiazolines **1a** and **1b** in combination with a range of IREDs (Supplementary Table 2). Negative controls without an IRED showed no color change, indicating that the utilized glucose dehydrogenase (GDH) has no side-activity toward such an imine reduction (in contrast to analogous reductions of other imines as reported in ref. ^34^). We subsequently performed biotransformations at a substrate concentration of 20 mM of 3-thiazoline **1b** using two IREDs, which were prioritized according to the results from the colorimetric pH shift assay. For in situ-cofactor regeneration, once again a GDH from *Bacillus subtilis*^25,35^ was used in combination with d-glucose as a co-substrate. After a reaction time of 24 h, formation of 3-thiazolidine **2b** was demonstrated (Table 1, entry 1), and revealed conversions of 29 and 83% with perfect product selectivity even under non-optimized reaction conditions. Thus, these results demonstrate that IREDs are suitable biocatalysts for the reduction of 3-thiazolines avoiding undesired ring-opening or other side reactions known for classic chemical and chemocatalytic reduction methodologies.

**Table 1:** Reduction of 3-thiazolines using imine reductases (IREDs)

^a^ Examined imine reductases (IREDs) are literature-known enzymes^22,29^; for reasons of clarity, IREDs are numbered throughout this manuscript, and the corresponding names of the original strains with information about the gene sequences are given in Supplementary Table 4 and Supplementary Methods. IRED5: *Cupriavidus* sp. HPC(L)*;* IRED8: *Mycobacterium smegmatis;* IRED24: *Glycomyces tenuis*; total protein concentration (crude extract; for expression, see SDS-PAGE, Supplementary Fig. 39) are given in footnotes d and e

^b^Conversion (conv.) determined by GC

^c^ Enantiomeric excess (ee) was determined by means of chiral SFC-HPLC after derivatization. Absolute configuration was determined by vibrational circular dichroism for (*S*)-**2f** and absolute configuration for other 3-thiazolidines was assigned in analogy according to chiral SFC-HPLC data (for details, see Supplementary Table 6 and Supplementary Methods)

^d^ 5 mg mL^−1^

^e^ 1.2 mg ml^−1^

### Investigation of 3-thiazolines

Next, we focused on the study of the substrate scope, as well as the determination of the enantioselectivity of these IRED-catalyzed reduction of 3-thiazolines. Toward this end, we prepared a range of prochiral 3-thiazolines **1c**–**1f**, comprising monocyclic and *spiro*-type compounds (Fig. 3b and Table 1). Based on the results of the colorimetric pH shift assay for these 3-thiazolines (Supplementary Fig. 4 and Supplementary Table 2), the specific activities were determined by means of a spectrophotometric study, and it is noteworthy that activities were found for all studied 3-thiazolines (Supplementary Figs. 5, 6 and Supplementary Table 3). Utilizing the IREDs which showed the highest specific activities, biotransformations of the prochiral 3-thiazolines running at a 20 mM substrate concentration on a 10 mL scale and in situ-cofactor regeneration with a GDH were performed (Table 1, entry 2–5). We found that for each substrate at least one IRED catalyzes the reduction with good to excellent conversion (82–98%) under non-optimized reaction conditions (Table 1). Activity was higher when R^3^ comprises methyl groups as substituents compared to the sterically less hindered hydrogen. Thus, when starting from prochiral 3-thiazolines **1c** and **1e**, which bear hydrogens at R^3^, a higher total protein concentration of 5 mg mL^−1^ had to be used for achieving good conversions (Table 1, entry 2, 4). In contrast, for good to excellent conversions a much lower amount of IRED8^22^ with a total protein concentration (crude extract) of 1.2 mg mL^−1^ was sufficient when utilizing prochiral 3-thiazolines **1d** and **1f** (Table 1, entry 3, 5). Moreover, a higher activity of IRED8^22^ as well as conversion was found when using *spiro*-cyclic 3-thiazolidine **1e** and **1f** compared to the analogous 3-thiazolidines bearing methyl groups as substituents R^2^, **1c** and **1d**. The highest activity of all tested 3-thiazolines could be determined with the *spiro*-cyclic compound **1f**, which supports our observation that methyl groups at R^3^, as well as a *spiro*-cyclic scaffold as R^2^ substituents represent the best substitution pattern for reduction of 3-thiazolines using IREDs. Negative controls of biotransformations of 3-thiazolines without IRED again showed no conversion, indicating that the utilized GDH has no side-activity towards this imine reduction. Furthermore, we found that IREDs catalyze the reduction of prochiral 3-thiazolines **1c**–**1f** with high to excellent enantioselectivities (96–99% ee) independent of the substituents R^2^ and R^3^ (Table 1, entry 2–5), thus representing the first access to 3-thiazolidines with high-enantiomeric excess via reduction of the C = N double bond in 3-thiazolines (**1**). The absolute configuration of the predominant formation of the enantiomers of 3-thiazolidines turned out to be *S* (section Determination of the absolute configuration of **2f** and Table 1), which is in accordance with the previously reported selectivity of the imine reducases IRED8 and IRED24^22,29^. The opportunity to reduce monocyclic, as well as *spiro*-cyclic 3-thiazolines with high conversion and enantioselectivity underlines the value of this biocatalytic approach. *Spiro*-cyclic scaffolds represent important structural motifs in medicinal chemistry^36,37^ so that in the future this methodology could be useful toward drug discovery by constructing libraries with structurally highly diverse sulfur-containing *spiro*-heterocyclic amines.

### Determination of the absolute configuration of 2f

**Fig. 4:**
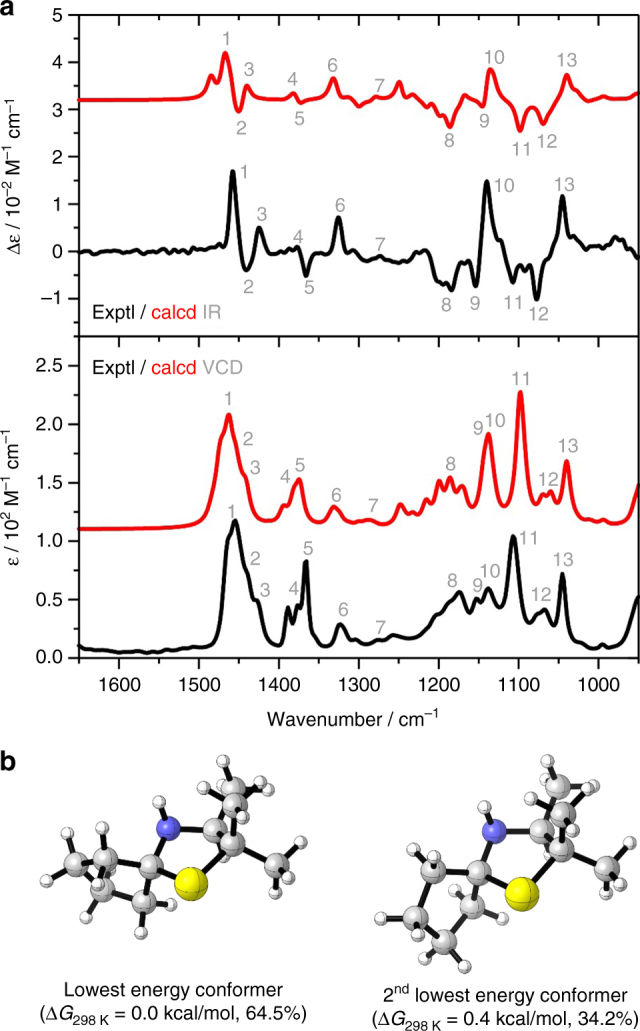
Determination of absolute configuration of **2f**. **a** Comparison of the experimental and calculated IR and VCD spectra of (*S*)-**2f** (numbers indicate some characteristic band assignments). **b** Structures of the two main conformers of (*S*)-**2f**, corresponding relative Gibbs free energies Δ*G*_298 K_ and the corresponding Boltzmann weights

Due to the lack of availability of enantiomerically pure reference compounds (indicating the difficulty to access them by other synthetic methods), we decided to determine the absolute configuration of **2f** (obtained from the experiment at elevated lab scale as described in section Process development on 3-thiazoline reduction) by vibrational circular dichroism (VCD) spectroscopy. The experimental IR and VCD spectra of **2f** were recorded in choloroform and are shown in Fig. 4a (for details, see Supplementary Methods). In order to simulate the spectra for the assignment of the configuration, a conformational analysis was carried out first for (*S*)-**2f** at the MMFF level of theory using Spartan 14 software (Spartan 14, Wavefunction Inc., Irvine, CA, USA (2014)). Subsequently, all eight obtained conformers were subjected to further geometry optimizations followed by spectra calculations at the B3LYP/6-311g++ (2d,p)/IEFPCM(CHCl_3_) level of theory (using the software Gaussian 09 Rev. E01, Frisch, M.J. et al. Gaussian, Inc., Wallingford CT, USA, (2013)). The relative Gibbs free energies Δ*G*_298 K_ and the corresponding Boltzmann weights of the two populated conformers are shown in Fig. 4b. Based on the single-conformer spectra, the IR and VCD spectra were simulated by assigning a Lorentzian band shape to the dipole and rotational strength calculated for each conformer and subsequent Boltzmann-averaging of the spectra. Direct comparison of the resulting simulated IR and VCD spectra with the experimental data, as indicated by the assignments given in Fig. 4a, reveals a very good agreement. Therefore, the absolute configuration can be assigned with very high confidence as (*S*)-**2f**.

### Reduction of *2H*-1,4-benzothiazines

**Fig. 5:**
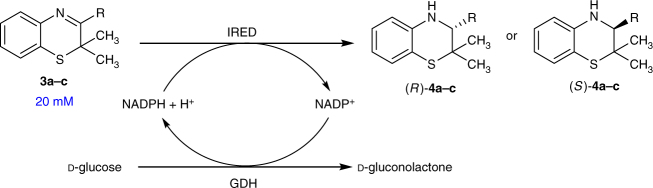
Imine reductase-catalyzed reduction of 2*H*-1,4-benzothiazines

Afterwards we studied further reductions using other sulfur-containing heterocyclic imines such as *2H*-1,4-benzothiazines (**3**) as substrates (Fig. 5), which have already been chemocatalytically reduced in an asymmetric fashion^16,17,38^. Therefore, the *2H*-1,4-benzothiazines **3a**–**3c** were prepared. Among these compounds, the prochiral representatives **3b** and **3c** contain either a methyl or phenyl group as substituents at R (Table 2). Again the colorimetric pH shift assay and subsequent spectrophotometric determination of the enzyme activities were conducted and the most promising IREDs prioritized (Supplementary Figs. 3, 5, 7 and Supplementary Tables 2, 3). It turned out that the specific activity for *2H*-1,4-benzothiazines **3a** and **3b** were about 10-fold higher compared to those for 3-thiazoline **1f**, which showed the highest activity of all tested 3-thiazolines. Moreover, the specific activities for prochiral *2H*-1,4-benzothiazine **3c**, bearing a phenyl as substituent at the prochiral carbon atom, is also about 10-fold higher compared to the 3-thiazolines **1a-e** (Supplementary Figs. 6, 7 and Supplementary Table 3). Based on these encouraging enzyme activities, showing high activity for 2*H*-1,4-benzothiazines also with sterically demanding substituents, as a next step synthetic biotransformations were carried out which also enabled us to gain insight into the enantioselectivity of such reactions (Table 2). These biocatalytic reductions were conducted at substrate concentrations of 20 mM on a 0.5 mL scale and the cofactor NADPH was recycled in situ by means of a GDH and d-glucose (Fig. 5).

**Table 2:** Reduction of 2*H*-1,4-benzothiazines using imine reductases (IREDs)

^a^ Examined imine reductases (IREDs) are literature-known enzymes^22,29^; for reasons of clarity, IREDs are numbered throughout this manuscript, and the corresponding names of the original strains with information about the gene sequences are given in Supplementary Table 4 and Supplementary Methods. IRED4: *Kribbella flavida* DSM 17836*;* IRED5: *Cupriavidus* sp. HPC(L)*;* IRED8: *Mycobacterium smegmatis;* IRED24: *Glycomyces tenuis*; IRED28: *Aeromonas veronii*; IRED29: *Aeromonas veronii*; total protein concentration (crude extract; for expression, see SDS-PAGE, Supplementary Fig. 39) are given in footnotes d and e

^b^Conversion (conv.) determined by SFC-HPLC

^c^ Enantiomeric excess (ee) was determined by means of chiral SFC-HPLC. Absolute configuration was assigned in analogy to the determined absolute configuration for (*S*)-**2f** according to chiral SFC-HPLC data (for details, see Supplementary Table 7 and Supplementary Methods)

^d^ 0.6 mg mL^−1^

^e^ 0.2 mg mL^−1^

Under non-optimized reaction conditions all *2H*-1,4-benzothiazines were converted to the corresponding 3,4-dihydro-*2H*-1,4-benzothiazines with moderate to good conversions of up to >99%, and most IREDs turned out to be suitable for catalyzing these reactions. In accordance with the determined enzyme activities, the amount of biocatalyst needed for the reduction of *2H*-1,4-benzothiazine **3c**, comprising a phenyl group at R, was higher compared to the one for *2H*-1,4-benzothiazines with a methyl group or a hydrogen atom at R (Table 2, entry 1–3). Negative controls of biotransformations of *2H*-1,4-benzothiazines without IRED again showed no conversion, indicating that the utilized GDH has no side-activity toward this imine reduction. Furthermore, we found that IREDs catalyze the reduction of *2H*-1,4-benzothiazines with high to excellent enantioselectivities (83–99% ee), and provided an access to both enantiomers of the 3,4-dihydro-*2H*-1,4-benzothiazines with high enantiomeric excess (Table 2, entry 2–3). Moreover, a higher enantioselectivity was observed for the more sterically demanding substrate **3c**, comprising a phenyl group at R (with 99% ee), compared to the 2*H*-1,4-benzothiazine **3b**, comprising a methyl group at R (with up to 99% ee). The imine reductases IRED5, IRED28 and IRED29^22,29^ were identified as most suitable enzymes for the reduction of *2H*-1,4-benzothiazine **3c** bearing a phenyl group at R. Notably, in addition to all tested 3-thiazolines IRED8^22^ turned out to be able to catalyze also the reduction of *2H*-1,4-benzothiazines **3a** and **3c**, thus representing an IRED being able to accept nearly all of the examined sulfur-containing heterocyclic imines.

### DFT studies

**Fig. 6:**
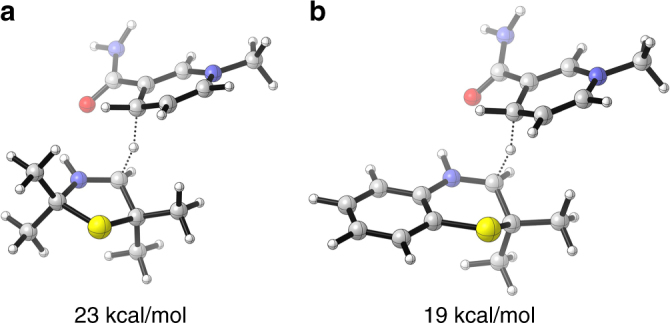
DFT studies. Transition states obtained by DFT calculations for the reduction of the non-substituted protonated 3-thiazoline (**a**) and protonated 2*H*-1,4-benzothiazine (**b**) by means of a dihydropyridine mimic as a reducing agent (Gibbs free energies of activation Δ*G*_298 K_ are given)

Taking into account the different activities of IREDs for 3-thiazolines (**1**) and *2H*-1,4-benzothiazines (**3**) with higher ones for the latter molecules, it is noteworthy that the (same) tendency is also observed for organocatalysts (being able to reduce *2H*-1,4-benzothiazines (**3**) but not 3-thiazolines (**1**) (Supplementary Fig. 1, and Supplementary Methods)). In addition, these findings are in accordance with observations of the Turner group, who found higher activities for six-membered rings compared to five-membered rings when examining the biocatalytic reduction of cyclic imines^20,23^. Therefore, we were interested in rationalizing this phenomena. Interestingly, both reduction methods are based on dihydropyridine moieties as reducing agents, namely the cofactor NADPH + H^+^ and a Hantzsch ester, respectively. Therefore, we performed orientating DFT studies (B3LYP 6-311 + G**; for further details, see Supplementary Methods) with a dihydropyridine mimic in combination with a structurally minimized 3-thiazoline and *2H*-1,4-benzothiazine, both of which were N-protonated. The barriers of activation associated with the transition states are 23 kcal/mol for the former (Fig. 6a) and 19 kcal/mol for the latter (Fig. 6b). Even though these gas-phase calculations are based on simplified (protonated) model systems, the higher barrier for the reduction of 3-thiazolines compared to the one for *2H*-1,4-benzothiazines is in good agreement with the lower activities found for imine reductases, as well as with the lack of reactivity for organocatalysts when reducing 3-thiazolines. This trend was also confirmed by calculations with the SMD intrinsic solvation model, as similar differences in the free energies of activation were obtained with parameters for water (17 kcal/mol vs. 13 kcal/mol) and chloroform (20 kcal/mol vs. 15 kcal/mol).

### Process development on 3-thiazoline reduction

**Fig. 7:**
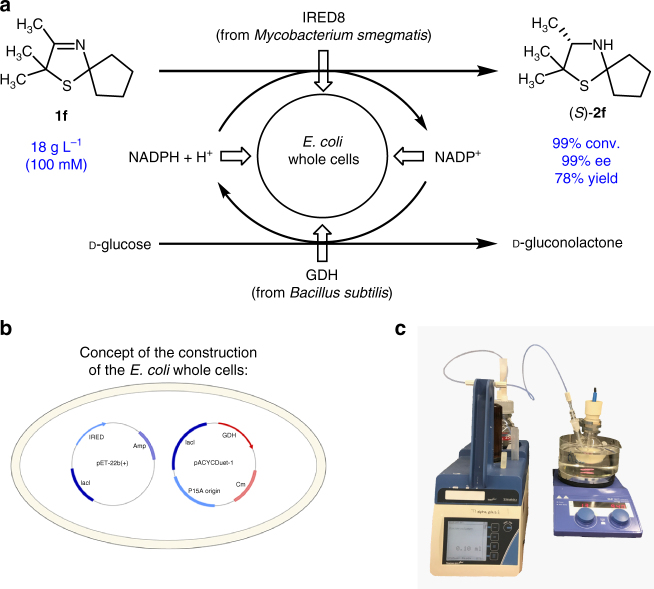
Process development on 3-thiazoline reduction. **a** Whole-cell catalyzed reduction of 3-thiazoline **1f** on 18 g L^−1^ scale, which corresponds to a substrate concentration of 100 mM. After 30 h of reaction time (*S*)-**2f** was obtained with 99% conversion (conv.) and 99% enantiomeric excess (ee) and was isolated with 78% yield. **b** Design of the recombinant whole-cell catalyst, containing imine reductase (IRED) from *Mycobacterium smegmatis* in a pET22b(+) vector and glucose dehydrogenase (GDH) from *Bacillus subtilis* in a pACYCDuet-1 vector. **c** Reaction setup for preparative scale experiment

The positive results concerning IRED-catalyzed reduction of sulfur-containing heterocyclic imines motivated us to start a process development exemplified by the enantioselective reduction of 3-thiazoline **1f** by means of IRED8^22^ (Fig. 7a). Towards this end, in an analogy to our previous work^25,35^ an *E*. *coli* whole-cell catalyst was designed, overexpressing the prioritized IRED8 and a glucose dehydrogenase from *Bacillus subtilis* for in situ-cofactor recycling. Both genes, encoding for IRED and GDH, respectively, have been inserted on different plasmids (Fig. 7b). Preparative scale experiments (40 mL) were performed with a substrate concentration of 100 mM and a lyophilized whole-cell catalyst using a Titrino apparatus to adjust the pH at 7 (Fig. 7c). We found that the reaction proceed smoothly also on preparative scale giving a very high conversion (99%) and enantioselectivity (99% ee for the *S*-enantiomer) within 30 h reaction time. After work up the desired 3-thiazolidine (*S*)-**2f** was isolated in 78% yield (Fig. 7a).

In summary, we reported a methodology which enables a reduction of 3-thiazolines under formation of the resulting 3-thiazolidines with high conversion and enantioselectivity of at least 96% ee and up to 99% ee, avoiding undesired ring-opening or other side reactions. This process technology, which is based on the use of IREDs as catalysts, has a broad substrate range and was also applied successfully to other sulfur-containing heterocyclic imines such as *2H*-1,4-benzothiazines. Furthermore, the trends for the reduction of different substrates could be rationalized by molecular modeling and an initial process development was also conducted, demonstrating the suitability of this method for preparative use. In future work, the focus will be on rationalizing the enzymatic reaction course and the enantioselectivity of this process by means of molecular modeling.

## Methods

### Biotransformations of 3-thiazolines

Biotransformations of 3-thiazolines (which were synthesized as described in Supplementary Methods; related NMR data are shown in Supplementary Figs. 40–55) were performed on 10 mL scale at 30 °C and 500 r.p.m. in 100 mM KP_i_ buffer pH 7, with 2% methanol as cosolvent containing 40 mM d-glucose, 20 mM 3-thiazoline **1a–f**, 1.2 mg mL^−1^ (in case of substrate **1d** and **1f** and IRED8) or 5 mg mL^−1^ IRED crude extract (in the other cases shown in Table 1, see Supplementary Table 4, Supplementary Methods for codon-optimized gene sequences, protein sequences, Supplementary Figs. 8–38 for plasmid structures and Supplementary Fig. 39 for SDS-Page), 24 U (in case of 1.2 mg mL^−1^ IRED) or 100 U of GDH (in the other cases shown in Table 1) and 0.1 mM NADP^+^. After 24 h, the reaction was stopped by adding 200 µL of 32% NaOH solution and 10 mL of dichloromethane. Phase separation was promoted by centrifugation and the conversion was determined by analyzing the organic phase by means of achiral GC (Supplementary Figs. 56–60, Supplementary Table 5 and Supplementary Methods; synthesis of racemic 3-thiazolidine reference compounds is described in Supplementary Methods and related NMR data are shown in Supplementary Figs. 61–70). For determination of the enantiomeric excess, samples were derivatized according to General Procedure 5 in the Supplementary Methods (NMR data of derivatized references are shown in Supplementary Figs. 71–78) and then analyzed by SFC-HPLC (Supplementary Figs. 79–82, Supplementary Table 6 and Supplementary Methods). The results of these experiments are shown in Table 1, details about performing of negative controls are provided in the Supplementary Methods. The biotransformation on preparative scale (40 mL) using a whole-cell catalyst was performed in a similar fashion and is described in detail in Supplementary Methods. ^1^H and ^13^C NMR spectra of (*S*)-**2f** are shown in Supplementary Figs. 83 and 84.

### Biotransformations of 2H-1,4-benzothiazines

Biotransformations of *2H*-1,4-benzothiazines (which were synthesized as described in Supplementary Methods; related NMR data are shown in Supplementary Figs. 40–43 and 85–92) were performed in a similar fashion as the biotransformations of 3-thiazolines. The conversion was determined by analyzing the organic phase by SFC-HPLC (Supplementary Figs. 93–95, Supplementary Table 7 and Supplementary Methods; synthesis of racemic 3-3,4-dihydro-2*H*-1,4-benzothiazine reference compounds is described in Supplementary Methods and related NMR data are shown in Supplementary Figs. 96–101). The results of these experiments are shown in Table 2, details are provided in Supplementary Methods.

### Data availability

The data that supports the findings of this study are available from the corresponding author upon request.

## Electronic supplementary material

Supplementary Information

## References

[1] Elander RP (2003). Industrial production of β-lactam antibiotics. Appl. Microbiol. Biotechnol..

[2] Nakatani S (2008). Combination of non-natural D-amino acid derivatives and allophenylnorstatine-dimethylthioproline scaffold in HIV protease inhibitors have high efficacy in mutant HIV. J. Med. Chem..

[3] Weigert WM, Offermanns H, Scherberich P (1975). D-Penicillamine--production and properties. Angew. Chem. Int. Ed. Engl..

[4] Bertamino A (2013). Synthesis, in vitro, and in cell studies of a new series of indoline-3,2’-thiazolidine-based p53 modulators. J. Med. Chem..

[5] Kaluszyner A, Czerniak P, Bergmann ED (1961). Thiazolidines and aminoalkylthiosulfuric acids as protecting agents against ionizing radiation. Radiat. Res..

[6] Roberts JC, Koch KE, Detrick SR, Warters RL, Lubec G (1995). Thiazolidine prodrugs of cysteamine and cysteine as radioprotective agents. Radiat. Res..

[7] Kitano, M., Yagisawa, M., Morimoto, Y. Method for production of 1,3-thiazolidin-2-ones. *Eur. Pat. Appl*. EP387028A2 (1990).

[8] Rochat S, de Saint Laumer JY, Chaintreau A (2007). Analysis of sulfur compounds from the in-oven roast beef aroma by comprehensive two-dimensional gas chromatography. J. Chromatogr. A.

[9] Armenise D, Trapani G, Stasi F, Morlacchi F (1998). Synthesis and antimicrobial activity of some pyrrolo[1,2,3-de]-1,4-benzothiazines, Part 2. Arch. Pharm. Pharm. Med. Chem..

[10] Asinger F (1956). Über die gemeinsame Einwirkung von Schwefel und Ammoniak auf Ketone. Angew. Chem..

[11] Asinger F, Thiel M (1958). Einfache Synthesen und chemisches Verhalten neuer heterocyclischer Ringsysteme. Angew. Chem..

[12] Drauz K, Koban HG, Martens J, Schwarze W (1985). Phosphonic and phosphinic acid analogs of penicillamine. Liebigs Ann. Chem..

[13] Asinger F, Offermanns H (1967). Syntheses with ketones, sulfur, and ammonia or amines at room temperature. Angew. Chem. Int. Ed. Engl..

[14] Thiel M, Asinger F, Häussler K, Körner T (1959). Über die gemeinsame Einwirkung von elementarem Schwefel und gasförmigem Ammoniak auf Ketone, XXII. Mercaptoamine durch Reduktion von Thiazolinen-Δ^3^ oder Dihydro-Methathiazinen-Δ^3^ mit Lithiumalanat. Liebigs Ann. Chem..

[15] Vu TTH, Kumbhar PS, Figueras F (2003). Base-catalysed hydrogenation of sulphur-containing aldehydes. Adv. Synth. Catal..

[16] Wang Z, Jiang Z (2010). Recent advances in enantioselective organocatalytic reduction of C=N bonds with hantzsch esters as the hydride source. Asian J. Chem..

[17] Rueping M, Antonchick AP, Theissmann T (2006). Remarkably low catalyst loading in Brønsted acid catalyzed transfer hydrogenations: enantioselective reduction of benzoxazines, benzothiazines, and benzoxazinones. Angew. Chem. Int. Ed. Engl..

[18] Reiners I, Gröger H, Martens J (1997). A new enantioselective synthetic approach to β-aminothio-compounds via enantioselective reduction of N,S-heterocyclic imines. J. Prakt. Chem..

[19] Mitsukura K (2011). Purification and characterization of a novel (*R*)-imine reductase from *Streptomyces* sp. GF3587. Biosci. Biotechnol. Biochem..

[20] Leipold F, Hussain S, Ghislieri D, Turner NJ (2013). Asymmetric reduction of cyclic imines catalyzed by a whole-cell biocatalyst containing an (S)-imine reductase. ChemCatChem.

[21] Scheller PN (2014). Enzyme toolbox: novel enantiocomplementary imine reductases. ChemBioChem.

[22] Wetzl D (2015). Expanding the imine reductase toolbox by exploring the bacterial protein-sequence space. ChemBioChem.

[23] Hussain S (2015). An (R)-imine reductase biocatalyst for the ssymmetric reduction of cyclic imines. ChemCatChem.

[24] Maugeri Z, Rother D (2016). Application of imine reductases (IREDs) in micro-aqueous reaction systems. Adv. Synth. Catal..

[25] Zumbrägel N, Wetzl D, Iding H, Gröger H (2017). Asymmetric biocatalytic reduction of cyclic imines: design and application of a tailor-made whole-cell catalyst. Heterocycles.

[26] Gamenara D, Domínguez de María P (2014). Enantioselective imine reduction catalyzed by imine reductases and artificial metalloenzymes. Org. Biomol. Chem..

[27] Schrittwieser JH, Velikogne S, Kroutil W (2015). Biocatalytic imine reduction and reductive amination of ketones. Adv. Synth. Catal..

[28] Grogan G, Turner NJ (2016). InspIRED by nature: NADPH-dependent imine reductases (IREDs) as catalysts for the preparation of chiral amines. Chem. Eur. J..

[29] Wetzl D (2016). Asymmetric reductive amination of ketones catalyzed by imine reductases. ChemCatChem.

[30] Aleku GA (2017). A reductive aminase from. Aspergillus oryzae. Nat. Chem..

[31] Meneely KM, Lamb AL (2012). Two structures of a thiazolinyl imine reductase from *Yersinia enterocolitica* provide insight into catalysis and binding to the nonribosomal peptide synthetase module of HMWP1. Biochemistry.

[32] Meneely KM, Ronnebaum TA, Riley AP, Prisinzano TE, Lamb AL (2016). Holo structure and steady state kinetics of the thiazolinyl imine reductases for siderophore biosynthesis. Biochemistry.

[33] Pick A (2016). Identification and characterization of two new 5-keto-4-deoxy-D-glucarate dehydratases/decarboxylases. BMC Biotechnol..

[34] Roth S (2017). Extended catalytic scope of a well-known enzyme: asymmetric reduction of iminium substrates by glucose dehydrogenase. ChemBioChem.

[35] Biermann M, Bakonyi D, Hummel W, Gröger H (2017). Design of recombinant whole-cell catalysts for double reduction of C=C and C=O bonds in enals and application in the synthesis of Guerbet alcohols as industrial bulk chemicals for lubricants. Green Chem..

[36] Marson CM (2011). New and unusual scaffolds in medicinal chemistry. Chem. Soc. Rev..

[37] Zheng Y, Tice CM, Singh SB (2014). The use of spirocyclic scaffolds in drug discovery. Bioorg. Med. Chem. Lett..

[38] Arai N, Saruwatari T, Isobe K, Ohkuma T (2013). Asymmetric hydrogenation of quinoxalines, benzoxazines, and a benzothiazine catalyzed by chiral ruthenabicyclic complexes. Adv. Synth. Catal..

