# Supplementary material for: Enantioselective reduction of sulfur-containing cyclic imines through biocatalysis
Source: Nat Commun. 2018 May 16;9:1949. doi: 10.1038/s41467-018-03841-5 (PMC5955971; doi:10.1038/s41467-018-03841-5)
Supplement: Supplementary file 1 — Supplementary Information [file 41467_2018_3841_MOESM1_ESM.pdf]

Supplementary Information

Enantioselective reduction of sulfur-containing cyclic imines through biocatalysis

Zumbrägel *et al.*

Supplementary Figures

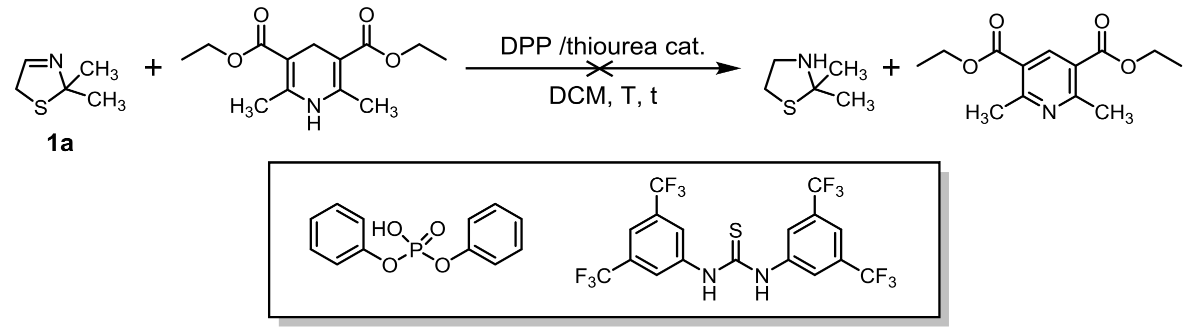

Supplementary Figure 1. Attempts towards reduction using Hantzsch esters. Different attempts towards reduction of 3-thiazoline **1a** by means of Hantzsch ester reduction were examined and are explained in details in Supplementary Table 1 and in Supplementary Methods.

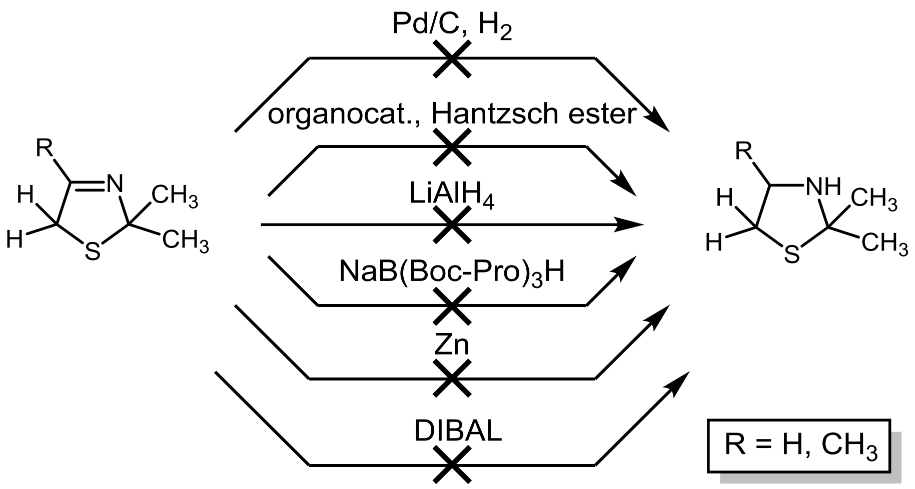

Supplementary Figure 2. Chemocatalytic approaches towards reduction of 3-thiazolines. Several classic chemical approaches were tested for reduction of 3-thiazolines and explained in detail in Supplementary Methods.

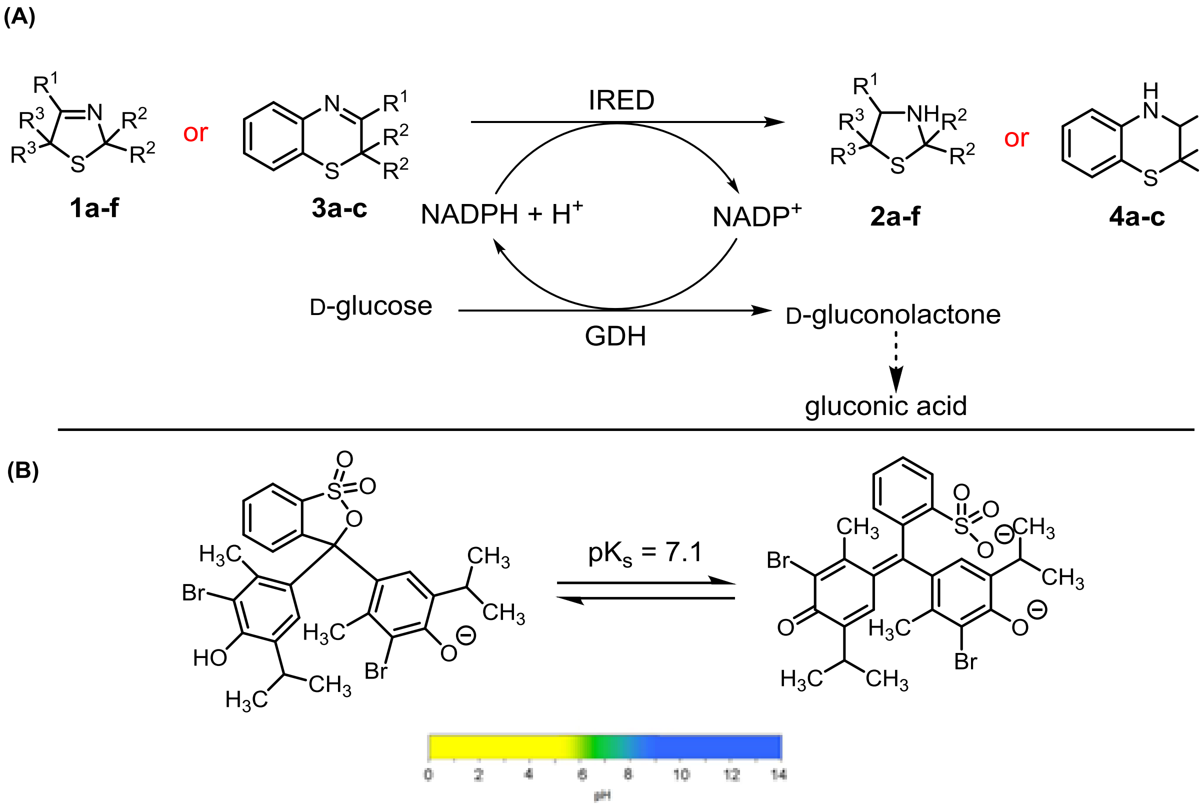

Supplementary Figure 3. Concept of colorimetric pH shift assay. (A) The colorimetric pH shift assay is an indirect screening method, based on a colour change of bromthymolblue depending on the pH. A decrease of pH under 5 leads to a colour change from blue/green to yellow. The formation of gluconic acid due to the consumption of the substrate **1a-f** / **3a-c** and subsequent regeneration of NADPH decreases the pH, resulting in the colour change. **(B)** Different structures and colours of bromthymolblue in dependence of the pH.

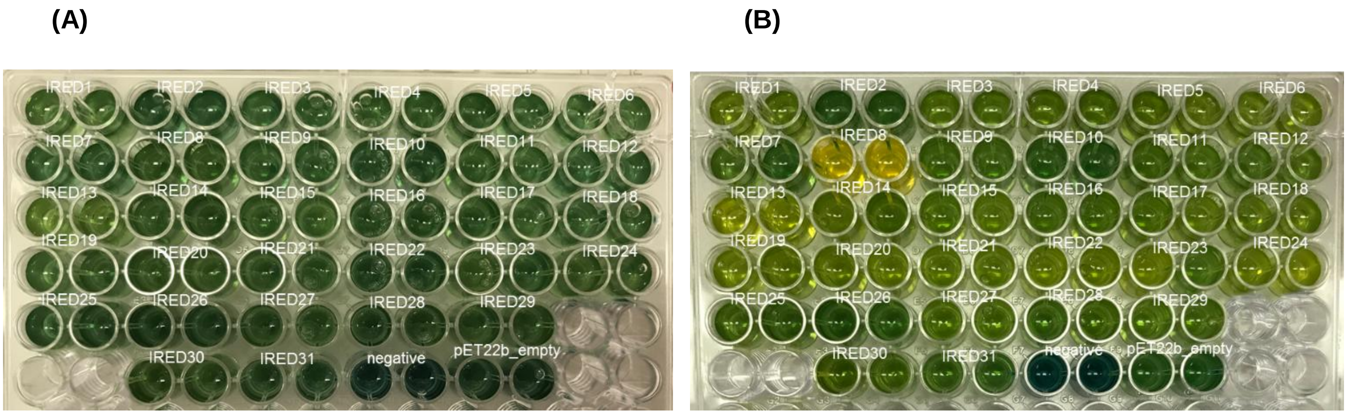

Supplementary Figure 4. Results of colorimetric pH shift assay. Exemplary for 3-thiazoline **1f** **(A)** Start of assay at T= 0 h **(B)** After 24 h. Colour change to yellow indicates positive IREDs.

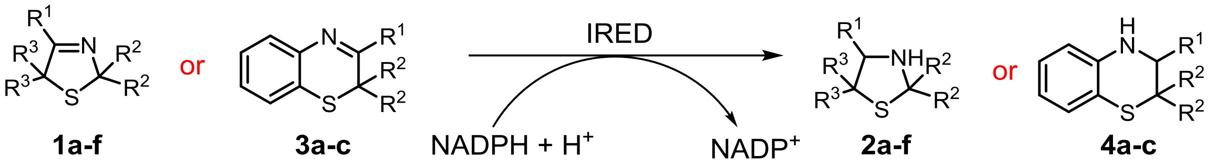

Supplementary Figure 5. Spectrophotometric activity assay. Decrease of NADPH is measured at 340 nm at spectrophotometer for 60 seconds. The spectrophotometric activity assay is explained in detail in the Supplementary Methods.

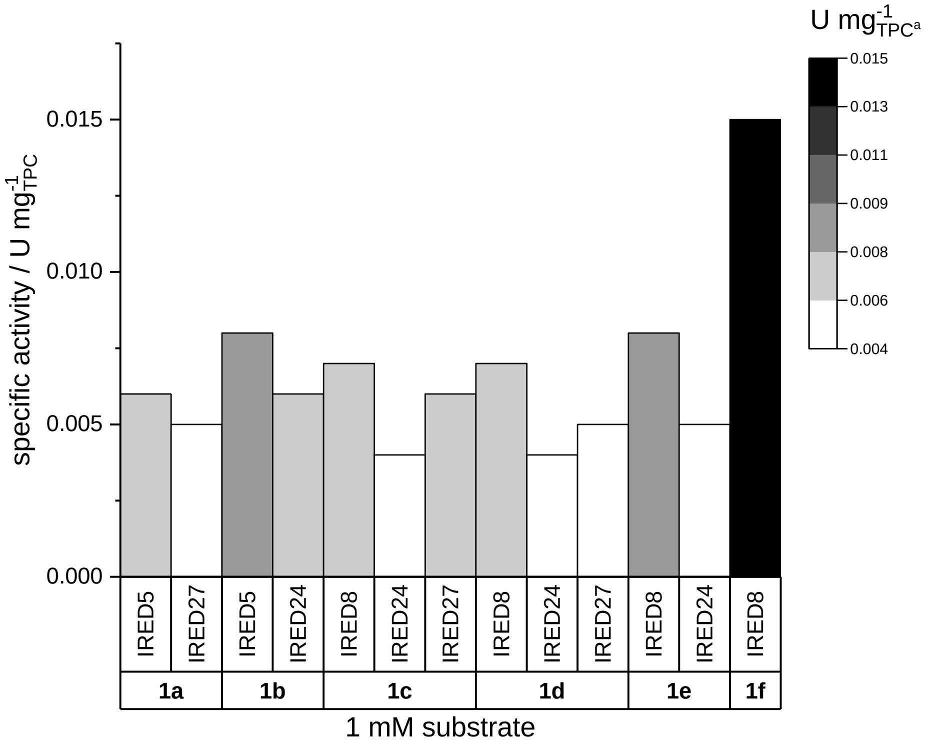

Supplementary Figure 6. Results of activity assay for 3-thiazolines 1a-f. Specific activity is given in U mg^{-1} total protein concentration (TPC). Activity was measured at 1 mM substrate concentration **1a-f**.

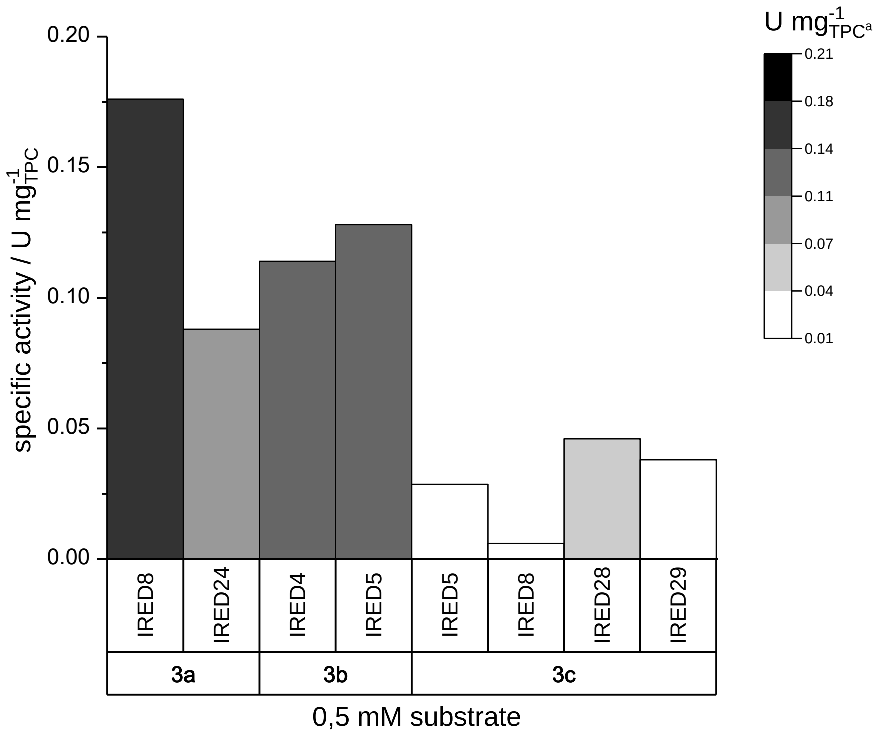

Supplementary Figure 7. Results of activity assay for *2H*-1,4-benzothiazines 3a-c. Specific activity is given in U mg^{-1} total protein concentration (TPC). Activity was measured at 0.5 mM substrate concentration **3a-c**.

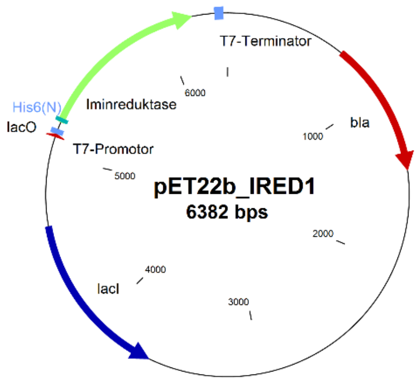

Supplementary Figure 8. Structure of the plasmid encoding for IRED1.[1]

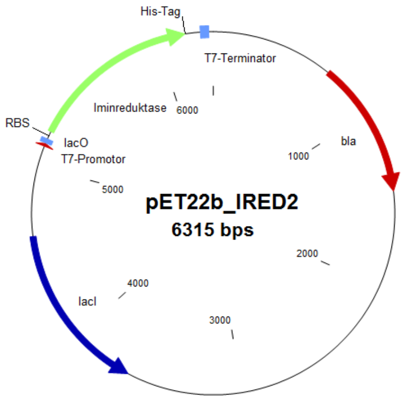

Supplementary Figure 9. Structure of the plasmid encoding for IRED2.[1]

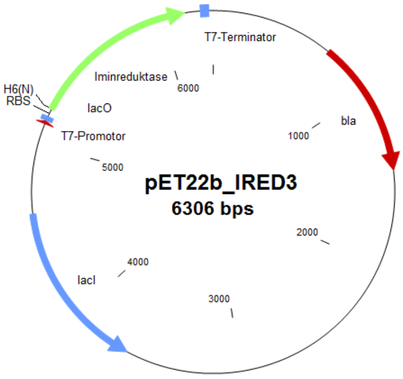

Supplementary Figure 10. Structure of the plasmid encoding for IRED3.[1]

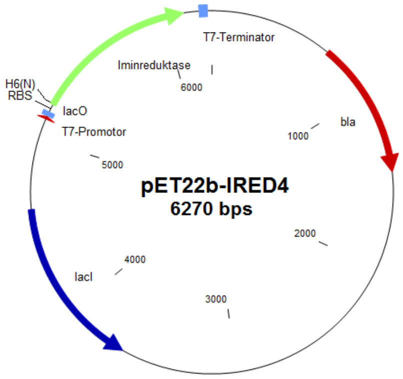

Supplementary Figure 11. Structure of the plasmid encoding for IRED4.[1]

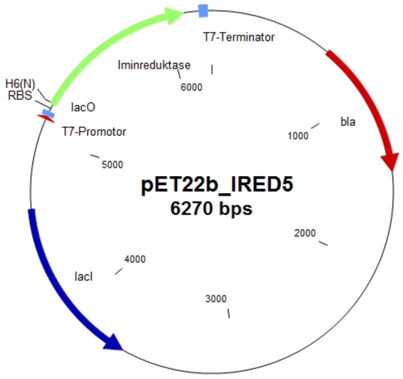

Supplementary Figure 12. Structure of the plasmid encoding for IRED5.[1]

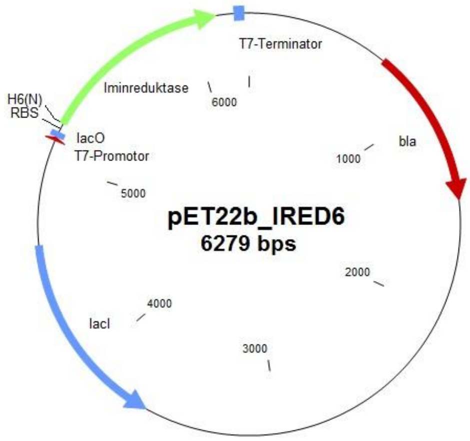

Supplementary Figure 13. Structure of the plasmid encoding for IRED6.[1]

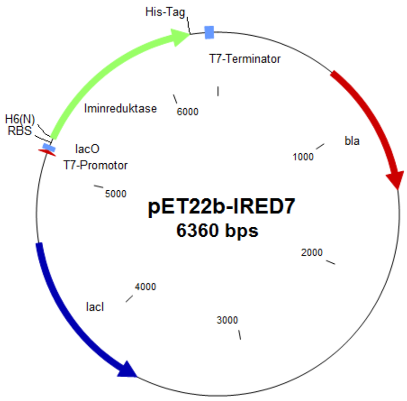

Supplementary Figure 14. Structure of the plasmid encoding for IRED7.[1]

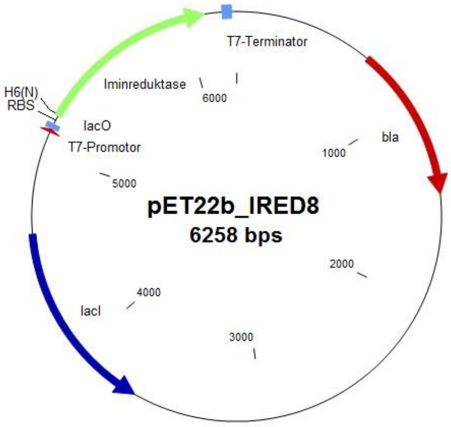

Supplementary Figure 15. Structure of the plasmid encoding for IRED8.[1]

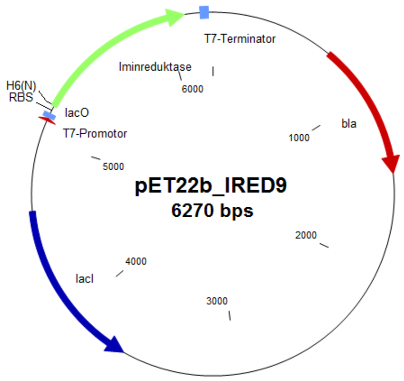

Supplementary Figure 16. Structure of the plasmid encoding for IRED9.[1]

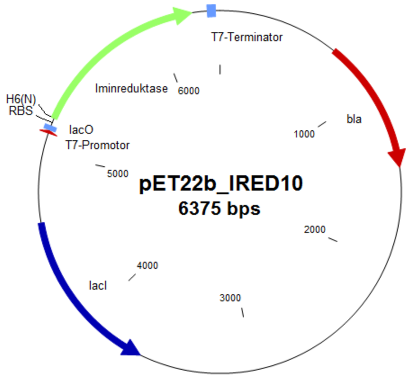

Supplementary Figure 17. Structure of the plasmid encoding for IRED10.[1]

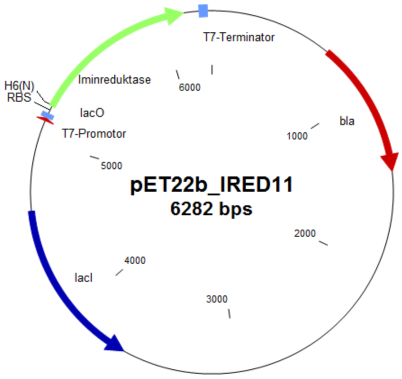

Supplementary Figure 18. Structure of the plasmid encoding for IRED11.[1]

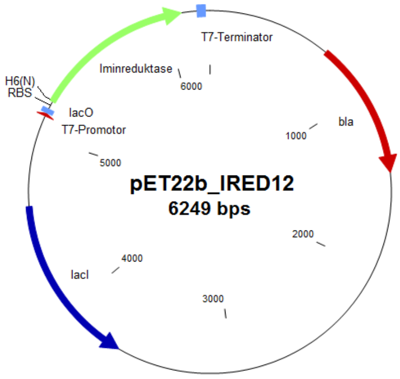

Supplementary Figure 19. Structure of the plasmid encoding for IRED12.[1]

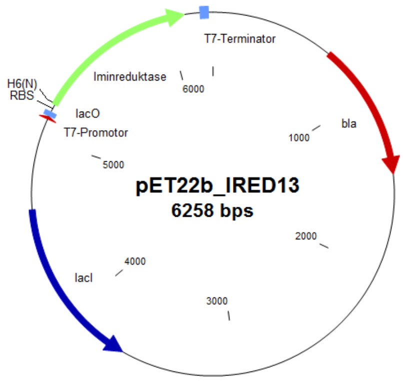

Supplementary Figure 20. Structure of the plasmid encoding for IRED13.[1]

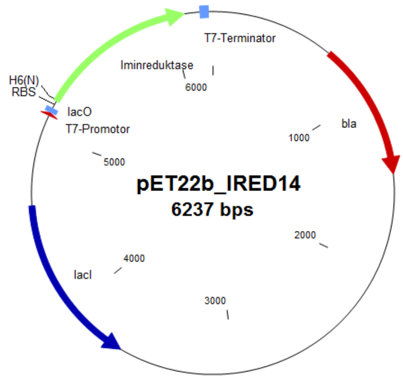

Supplementary Figure 21. Structure of the plasmid encoding for IRED14.[1]

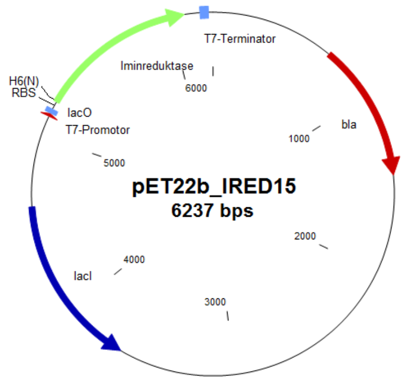

Supplementary Figure 22. Structure of the plasmid encoding for IRED15.[1]

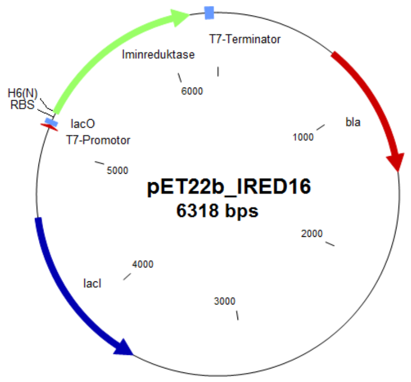

Supplementary Figure 23. Structure of the plasmid encoding for IRED16.[1]

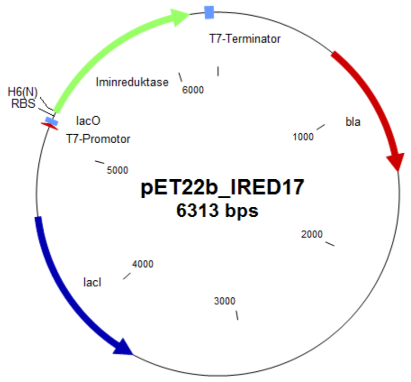

Supplementary Figure 24. Structure of the plasmid encoding for IRED17.[1]

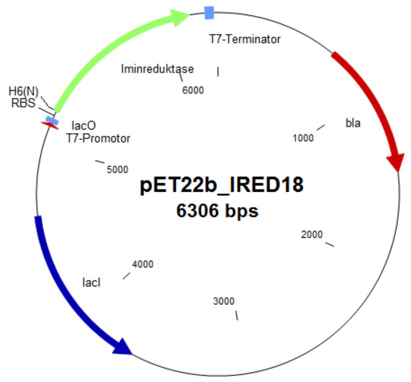

Supplementary Figure 25. Structure of the plasmid encoding for IRED18.[1]

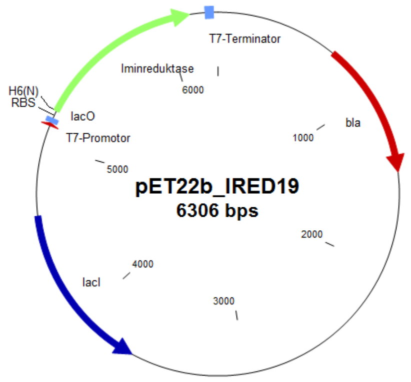

Supplementary Figure 26. Structure of the plasmid encoding for IRED19.[1]

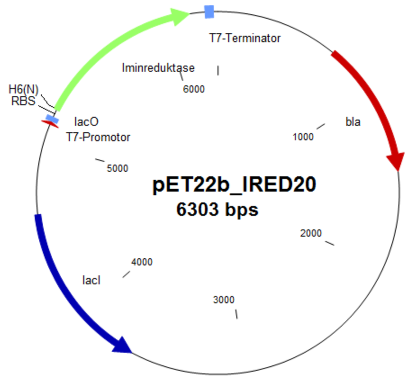

Supplementary Figure 27. Structure of the plasmid encoding for IRED20.[1]

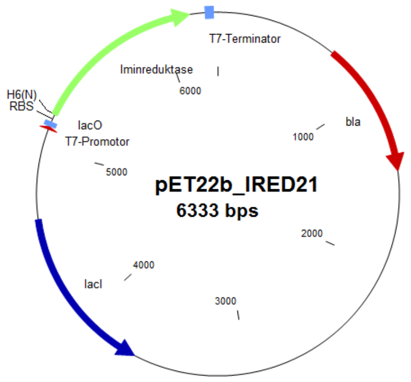

Supplementary Figure 28. Structure of the plasmid encoding for IRED21.[2]

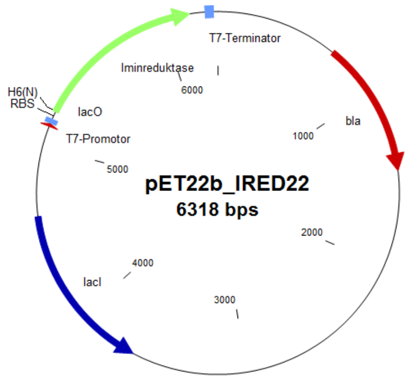

Supplementary Figure 29. Structure of the plasmid encoding for IRED22.[2]

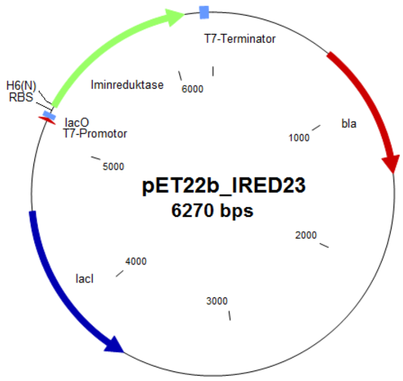

Supplementary Figure 30. Structure of the plasmid encoding for IRED23.[2]

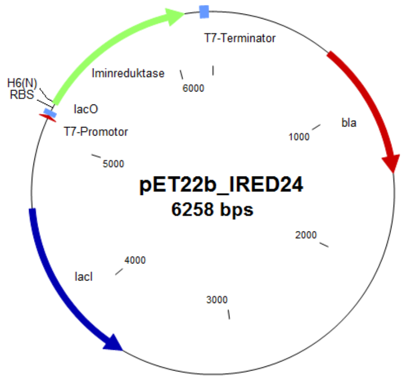

Supplementary Figure 31. Structure of the plasmid encoding for IRED24.[2]

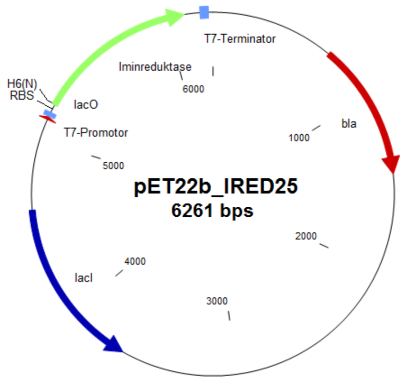

Supplementary Figure 32. Structure of the plasmid encoding for IRED25.[2]

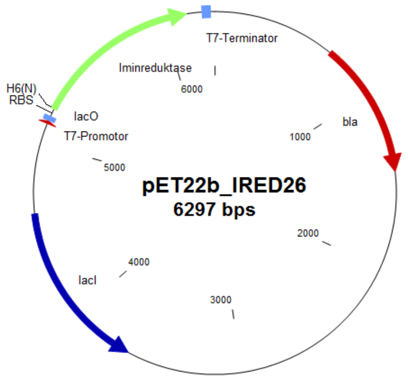

Supplementary Figure 33. Structure of the plasmid encoding for IRED26.[2]

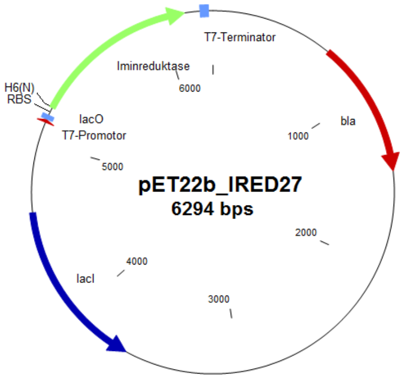

Supplementary Figure 34. Structure of the plasmid encoding for IRED27.[2]

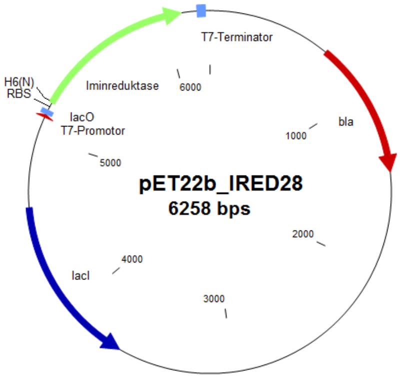

Supplementary Figure 35. Structure of the plasmid encoding for IRED28.[2]

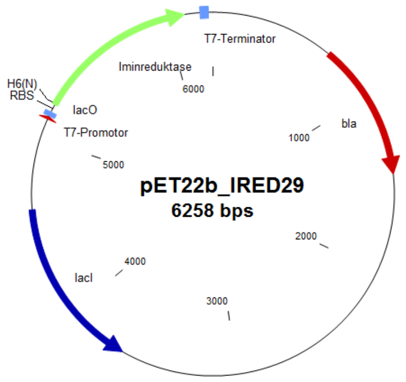

Supplementary Figure 36. Structure of the plasmid encoding for IRED29.[2]

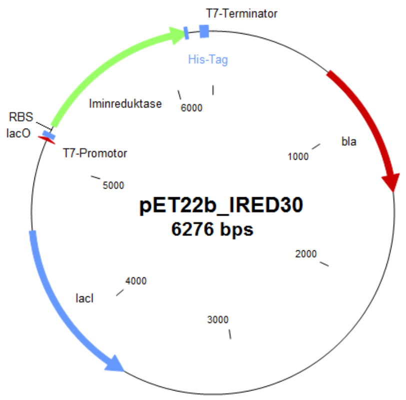

Supplementary Figure 37. Structure of the plasmid encoding for IRED30.[3]

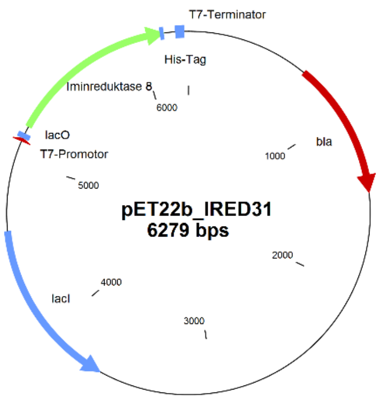

Supplementary Figure 38. Structure of the plasmid encoding for IRED31.[3]

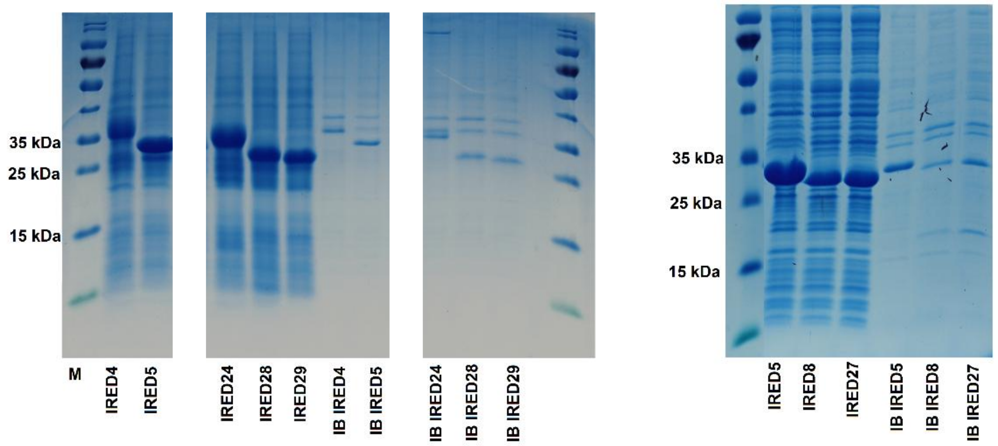

Supplementary Figure 39. SDS-PAGE of IRED crude extracts. M: Marker (PageRuler™ Prestained Protein Ladder, 10 to 180 kDa, Thermo Fisher Scientific), IB: Inclusion Bodies. All IREDs used for biotransformations were successfully overexpressed in *E. coli* BL21 (DE3).

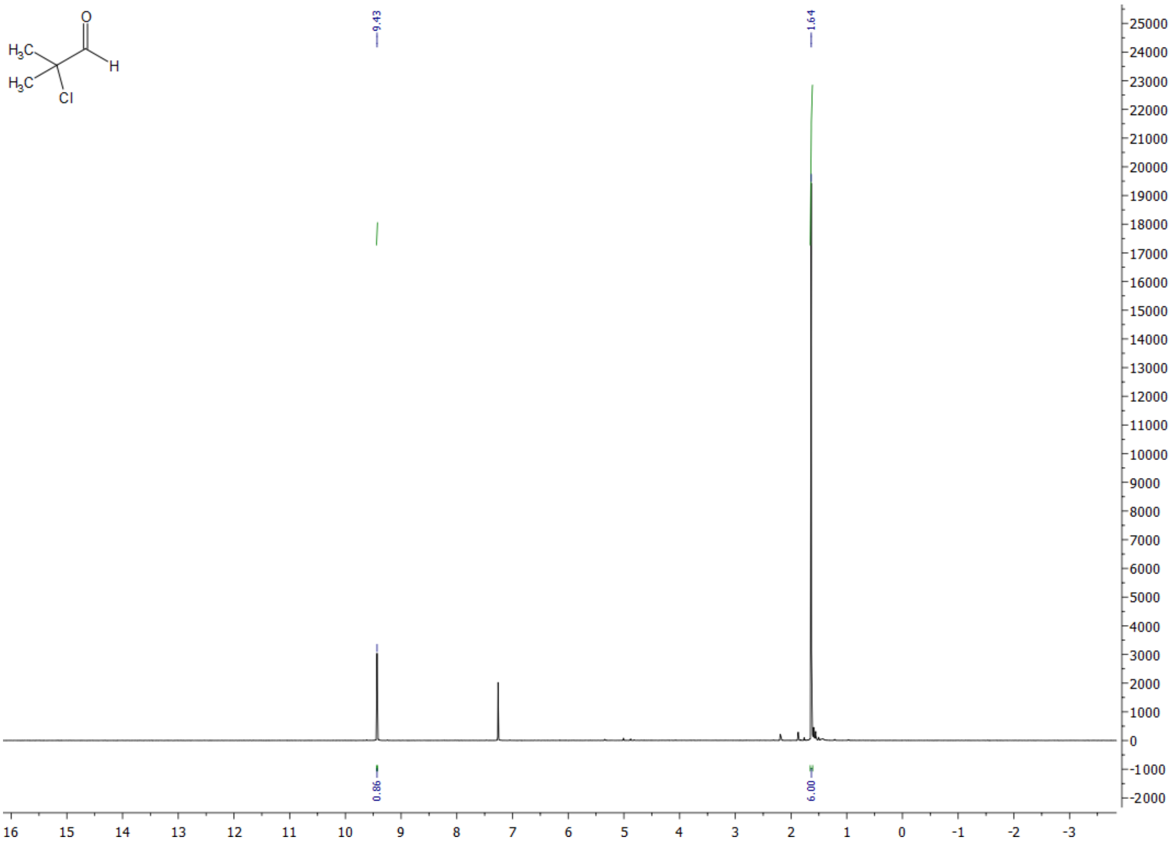

Supplementary Figure 40. ^{1}H NMR spectra of 2-chloro-2-methylpropanal.

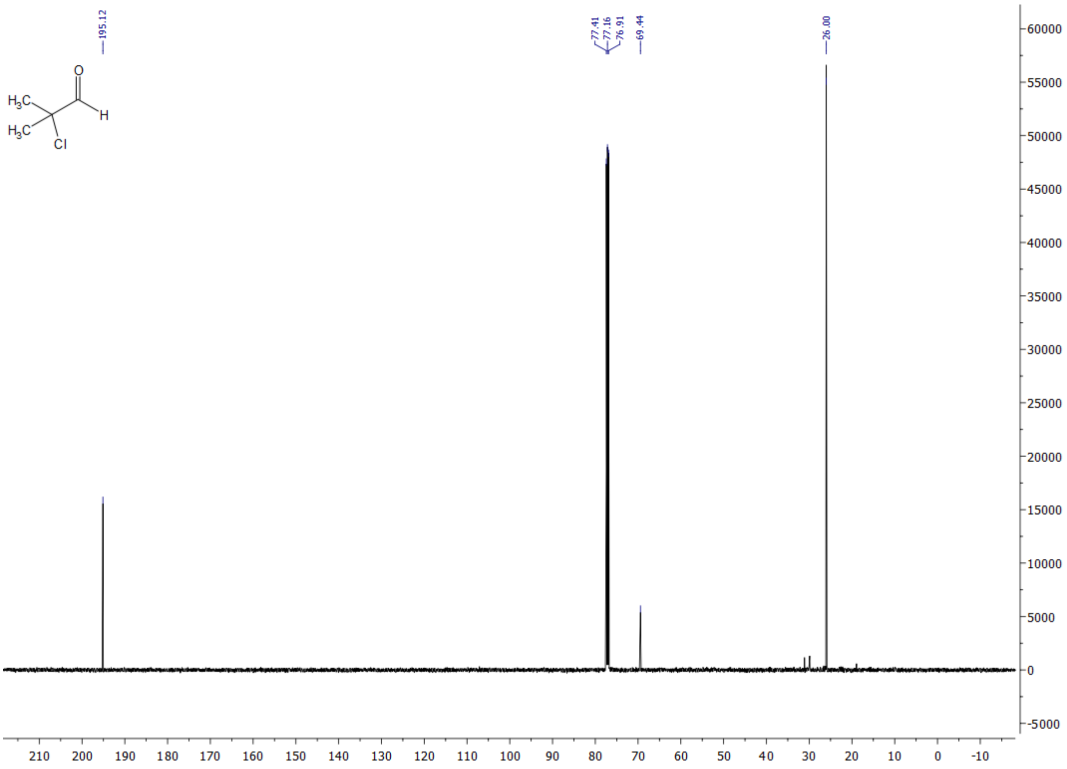

Supplementary Figure 41. ^{13}C NMR spectra of 2-chloro-2-methylpropanal.

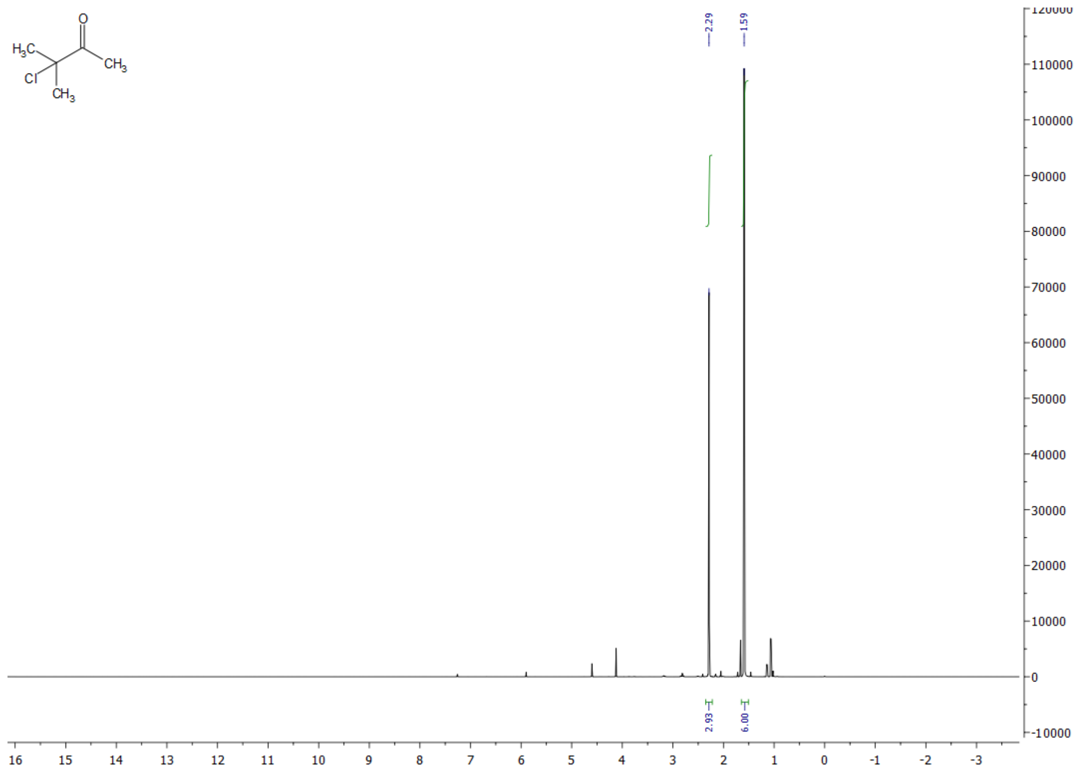

Supplementary Figure 42. ^{1}H NMR spectra of 3-chloro-3-methyl-2-butanone.

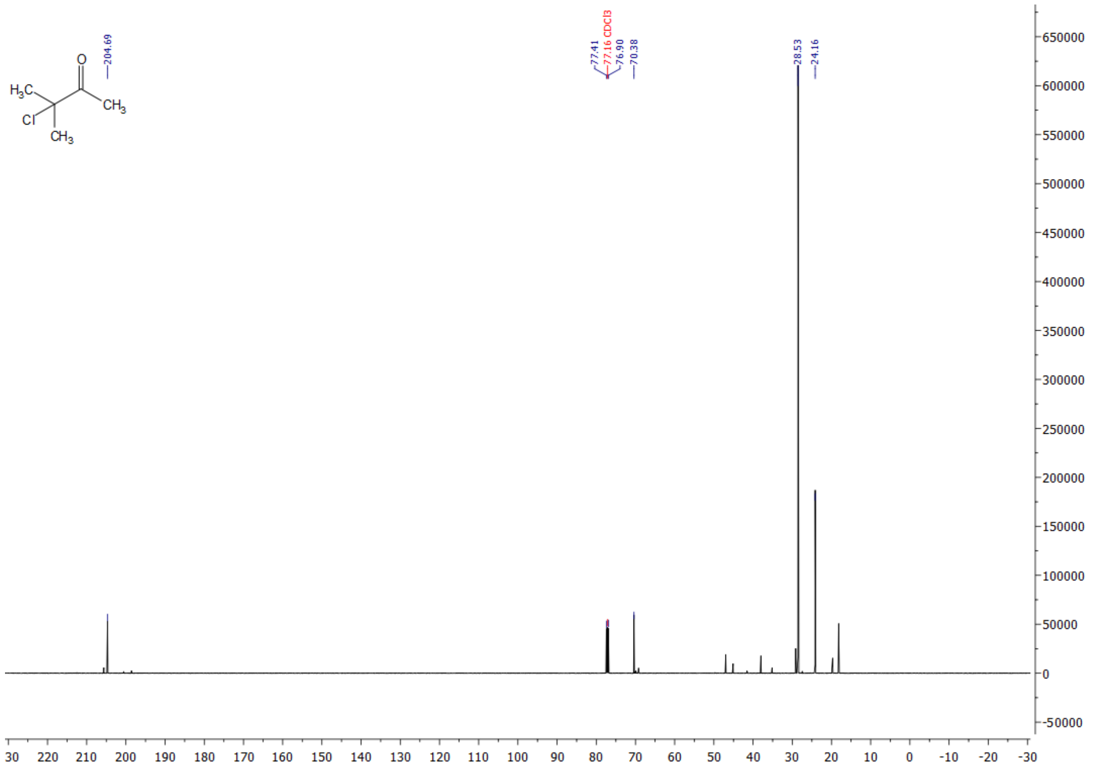

Supplementary Figure 43. ^{13}C NMR spectra of 3-chloro-3-methyl-2-butanone.

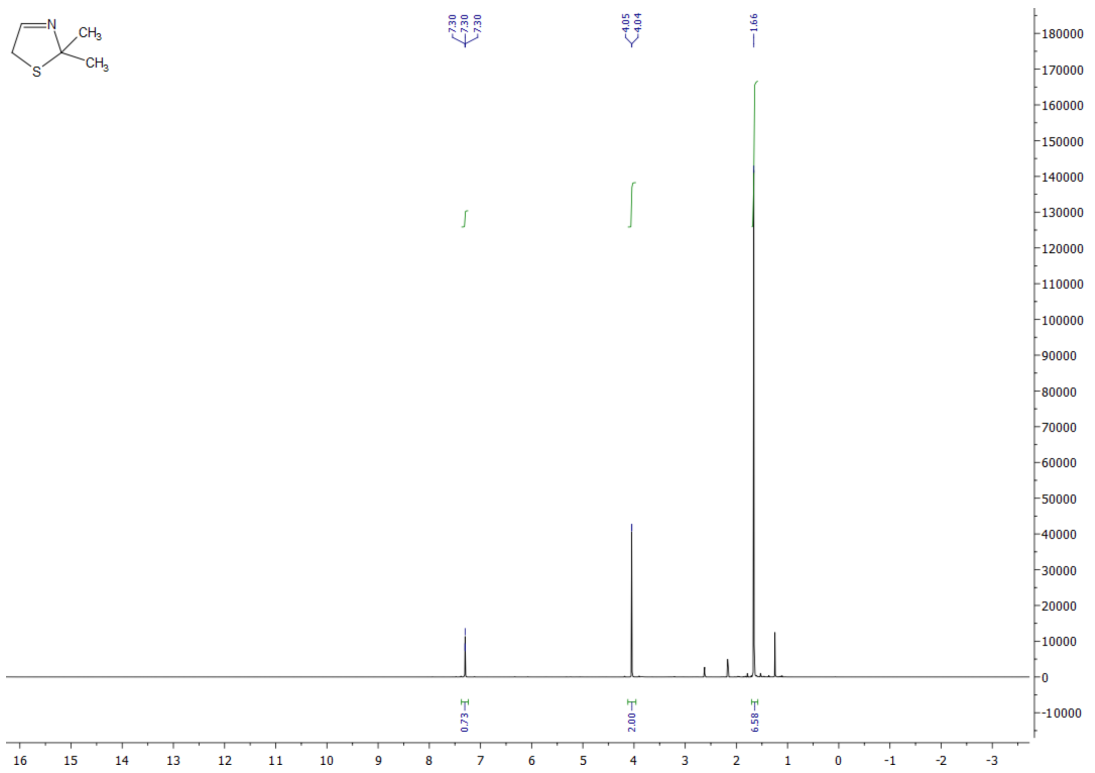

Supplementary Figure 44. ^{1}H NMR spectra of 2,2-dimethyl-3-thiazoline (1a).

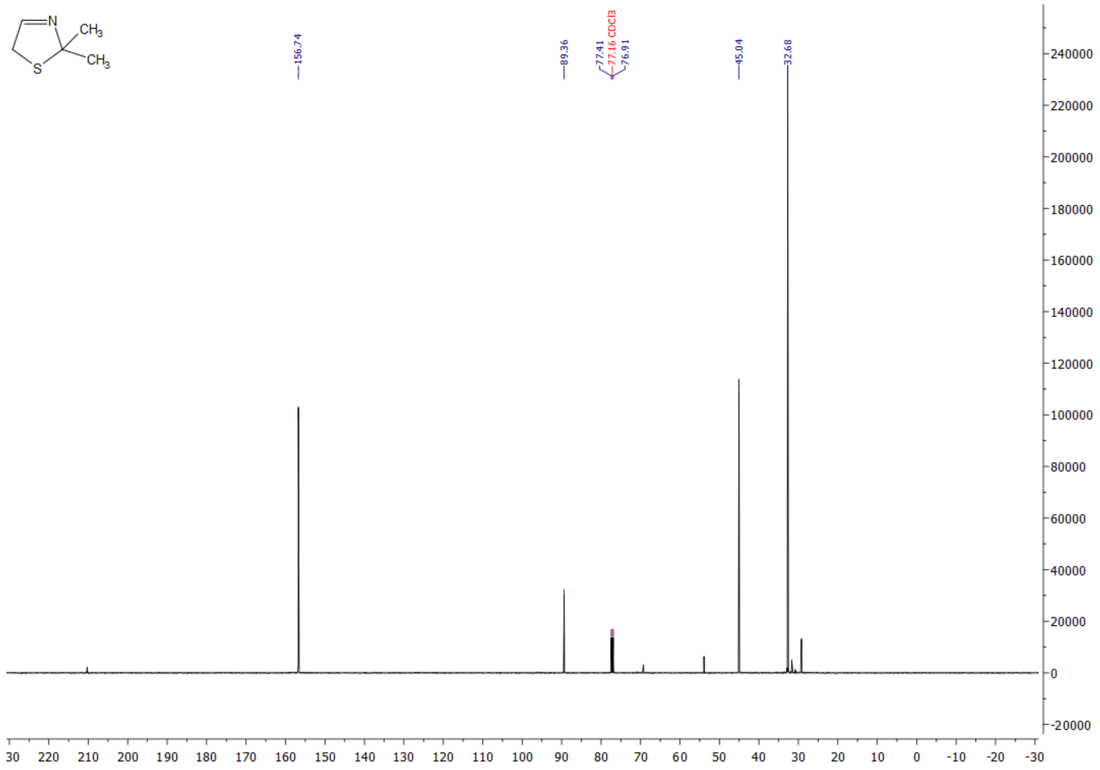

Supplementary Figure 45. ^{13}C NMR spectra of 2,2-dimethyl-3-thiazoline (1a).

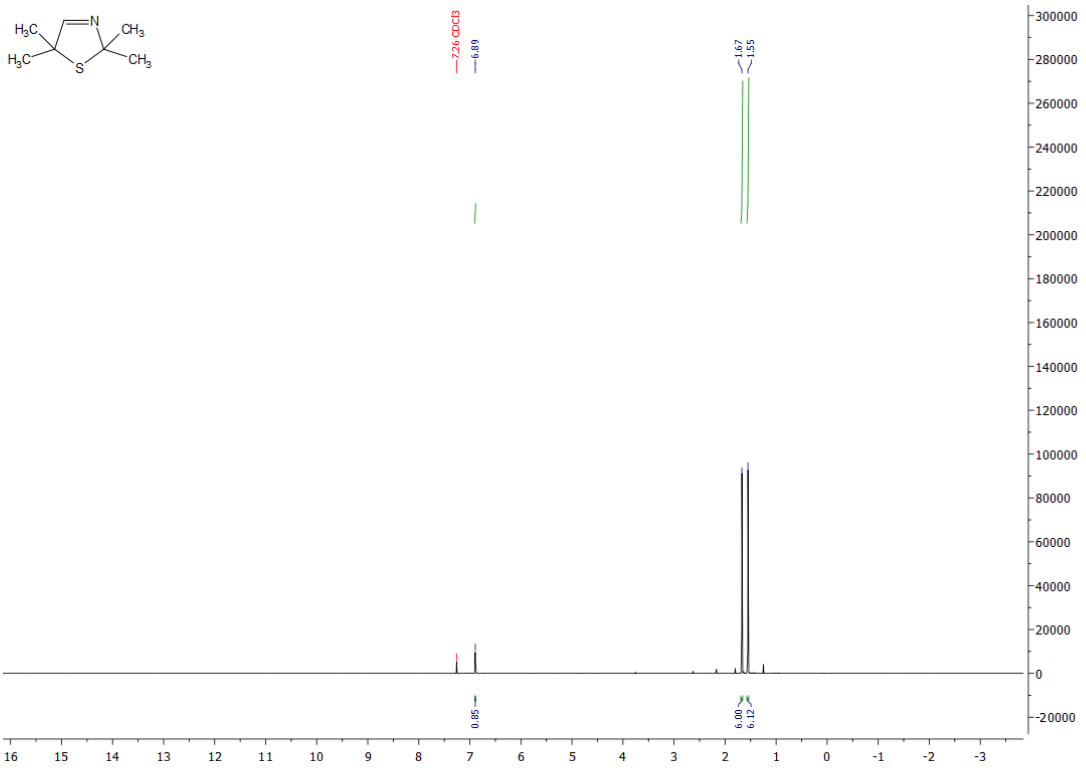

Supplementary Figure 46. ^{1}H NMR spectra of 2,2,5,5-tetramethyl-3-thiazoline (1b).

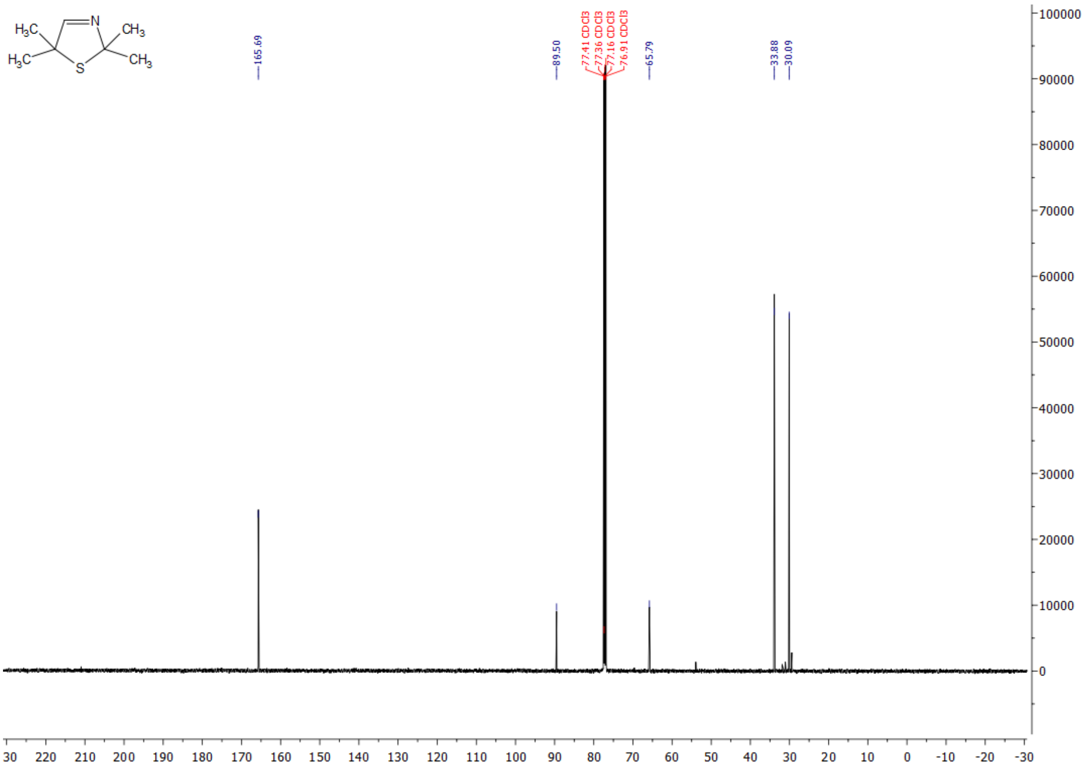

Supplementary Figure 47. ^{13}C NMR spectra of 2,2,5,5-tetramethyl-3-thiazoline (1b).

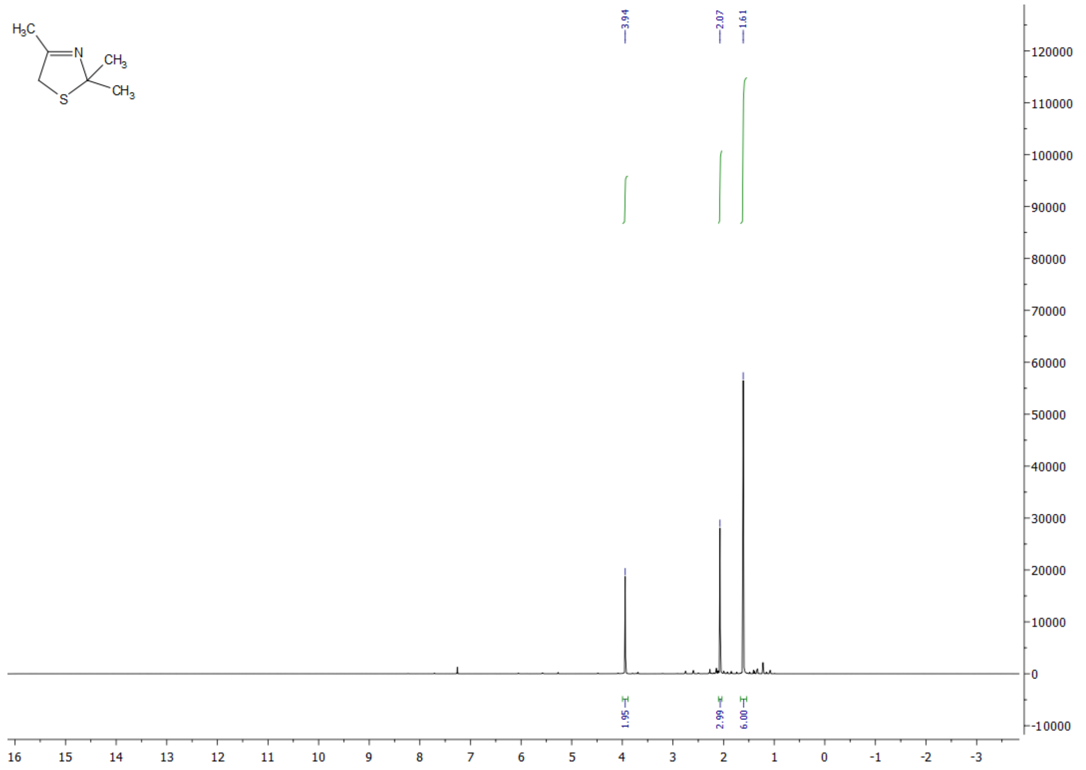

Supplementary Figure 48. ^{1}**H NMR spectra of 2,2,4-trimethyl-3-thiazoline (1c).**

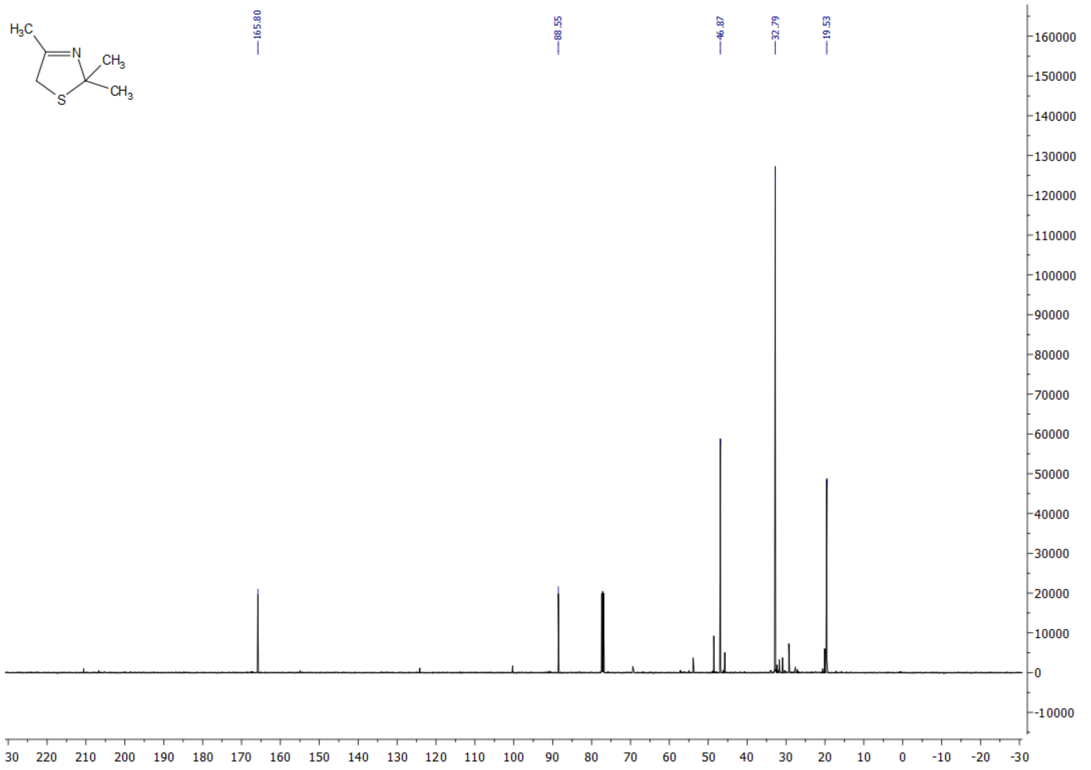

Supplementary Figure 49. ^{13}**C NMR spectra of 2,2,4-trimethyl-3-thiazoline (1c).**

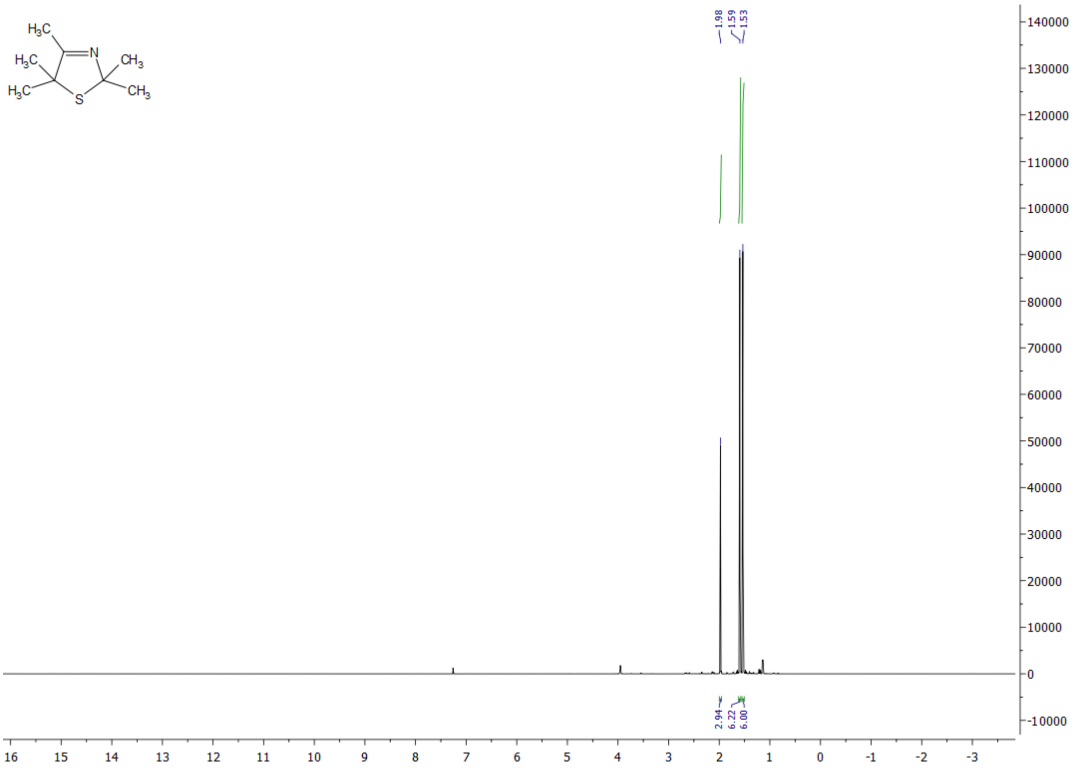

Supplementary Figure 50. ¹H NMR spectra of 2,2,4,5,5-pentamethyl-3-thiazoline (1d).

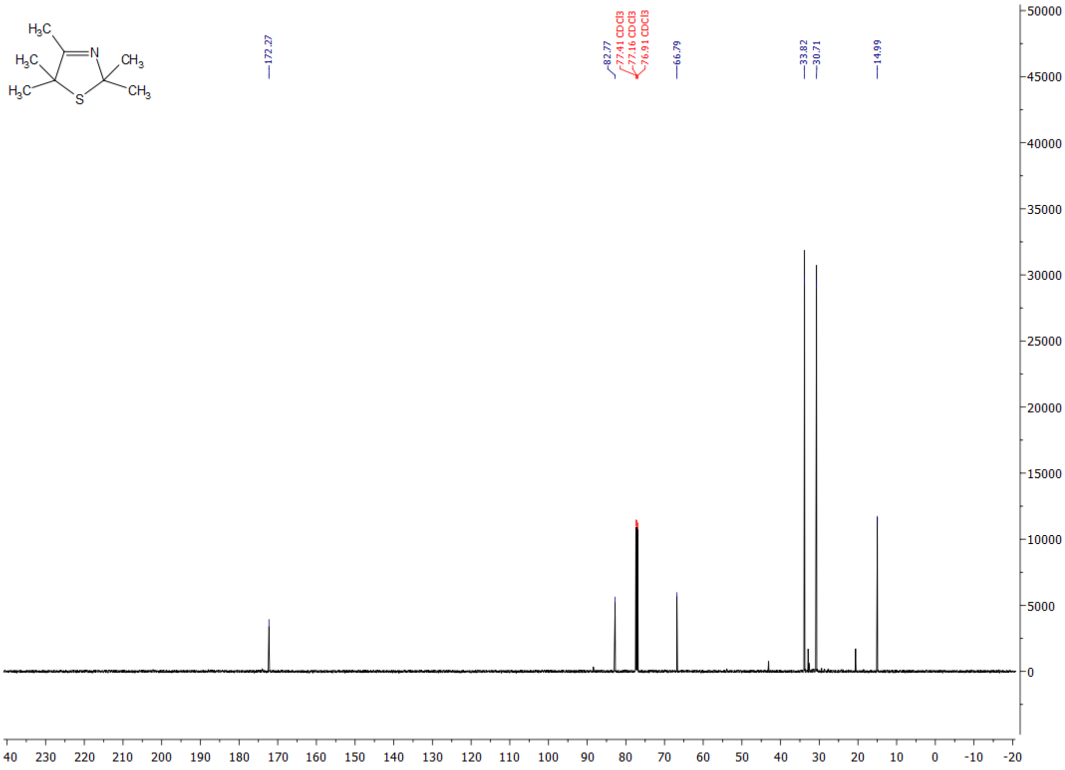

Supplementary Figure 51. ¹³C NMR spectra of 2,2,4,5,5-pentamethyl-3-thiazoline (1d).

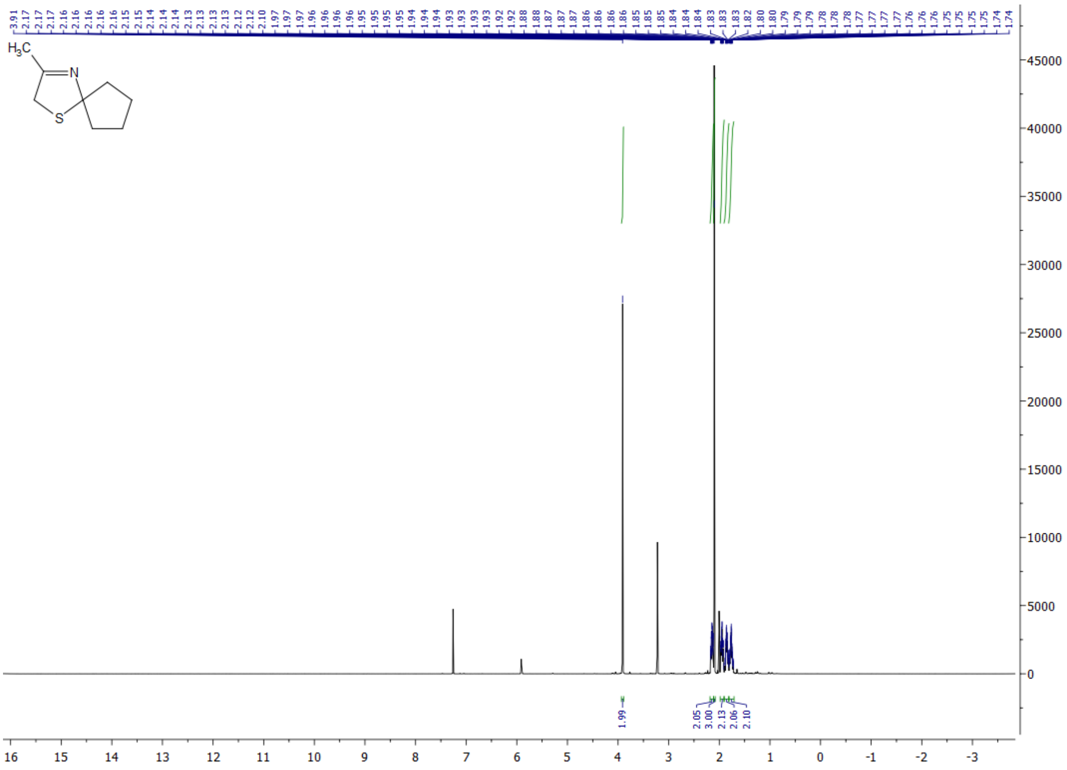

Supplementary Figure 52. **^{1}H NMR spectra of 1e.**

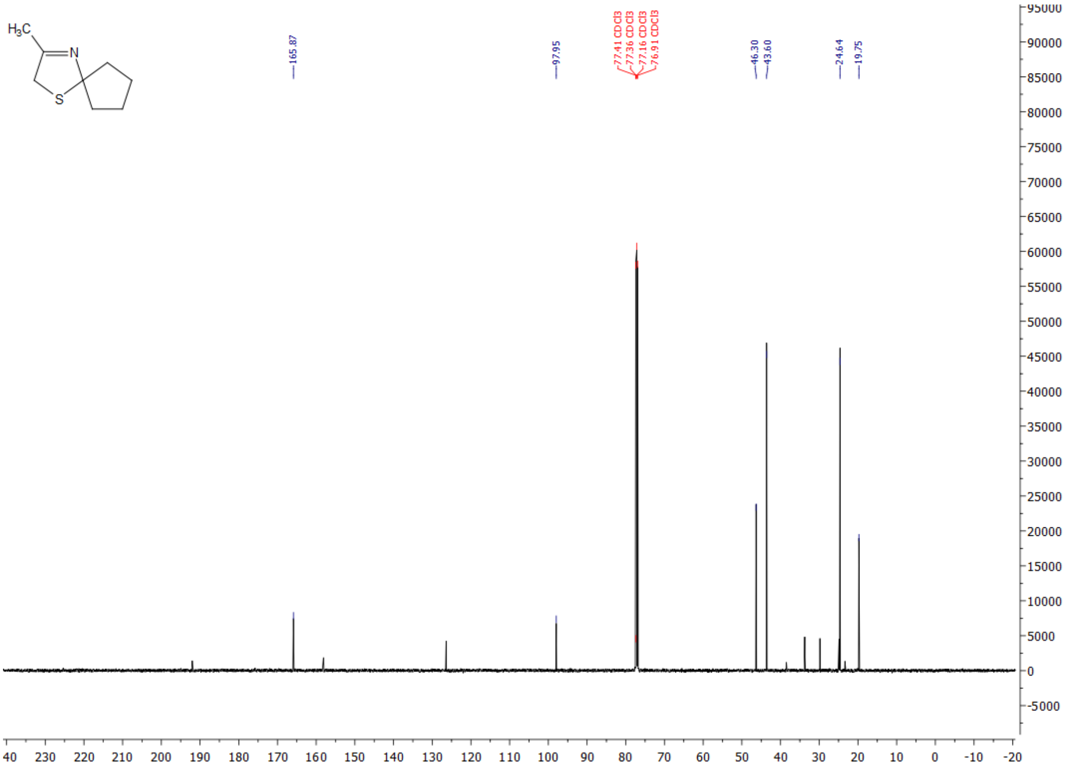

Supplementary Figure 53. **^{13}C NMR spectra of 1e.**

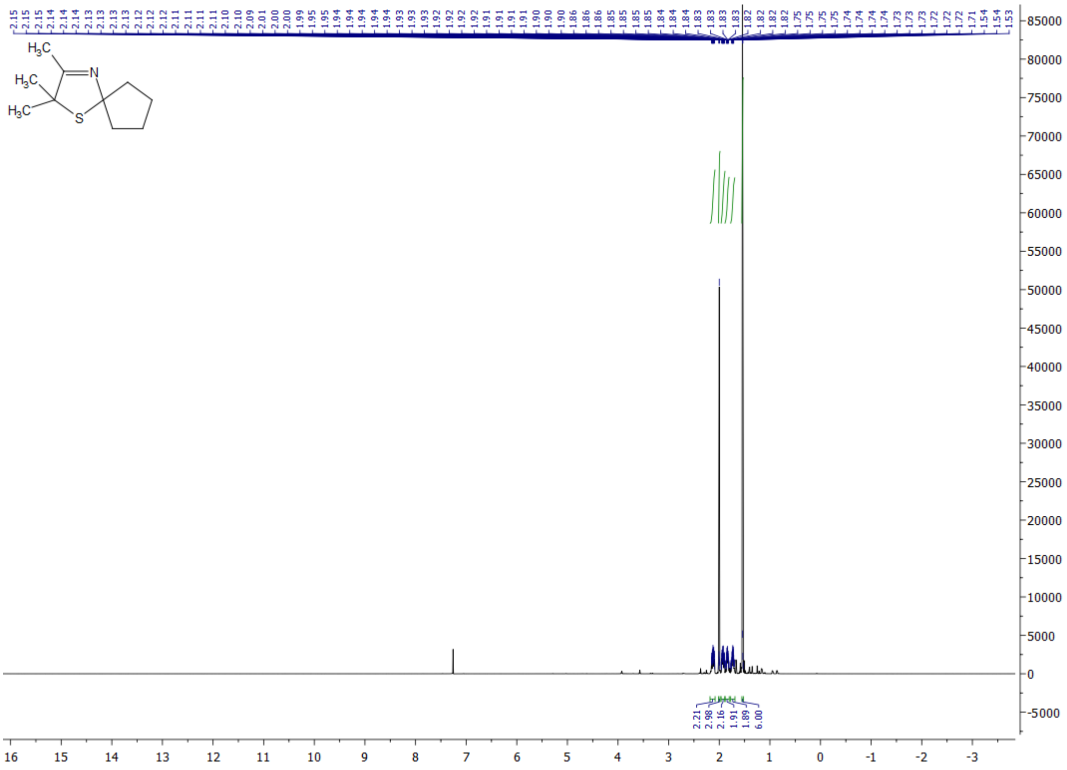

Supplementary Figure 54. ^{1}H NMR spectra of 1f.

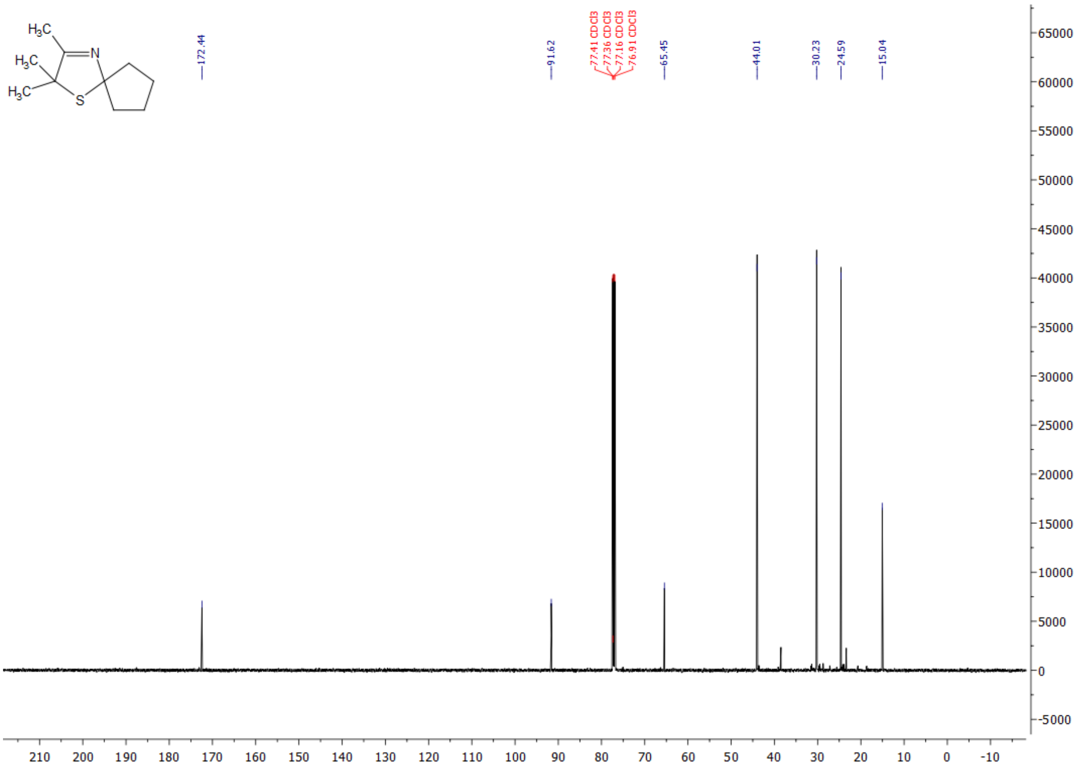

Supplementary Figure 55. ^{13}C NMR spectra of 1f.

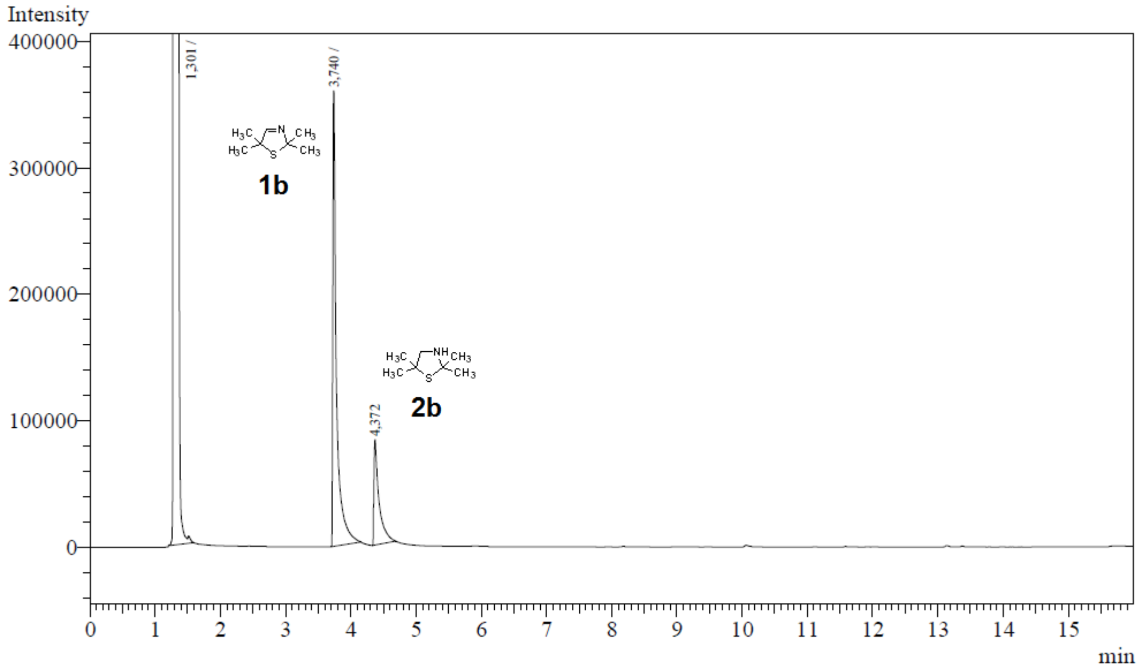

Supplementary Figure 56. Achiral GC chromatogram of 1b and rac-2b.

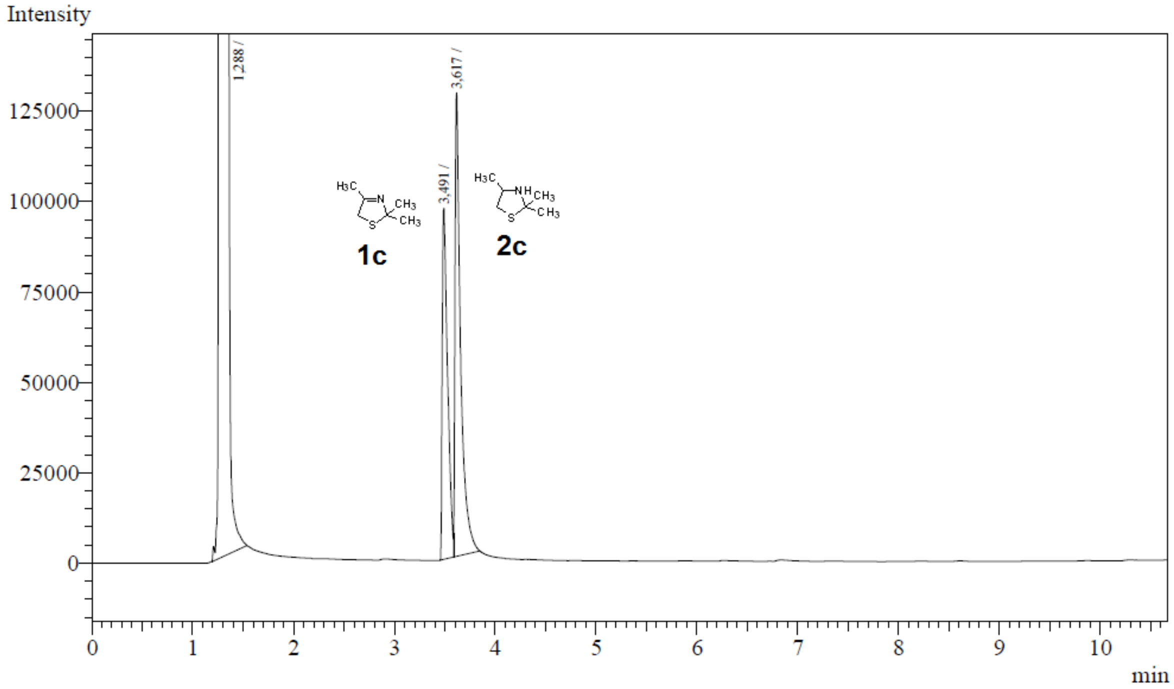

Supplementary Figure 57. Achiral GC chromatogram of 1c and rac-2c.

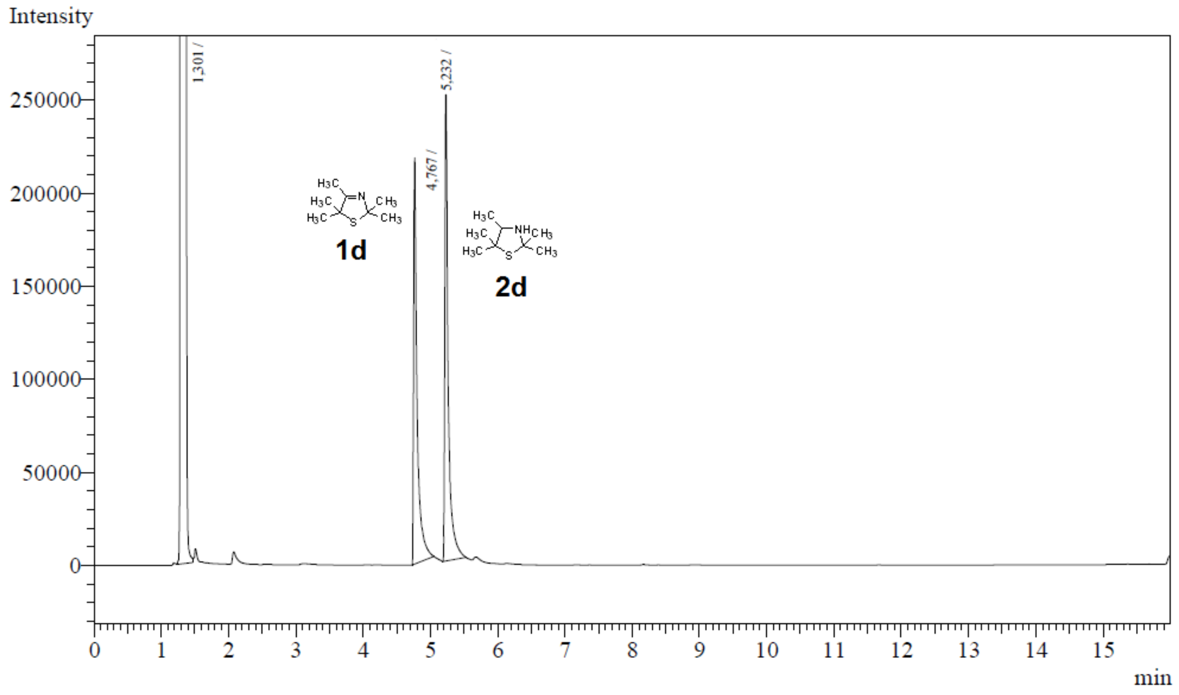

Supplementary Figure 58. Achiral GC chromatogram of 1d and rac-2d.

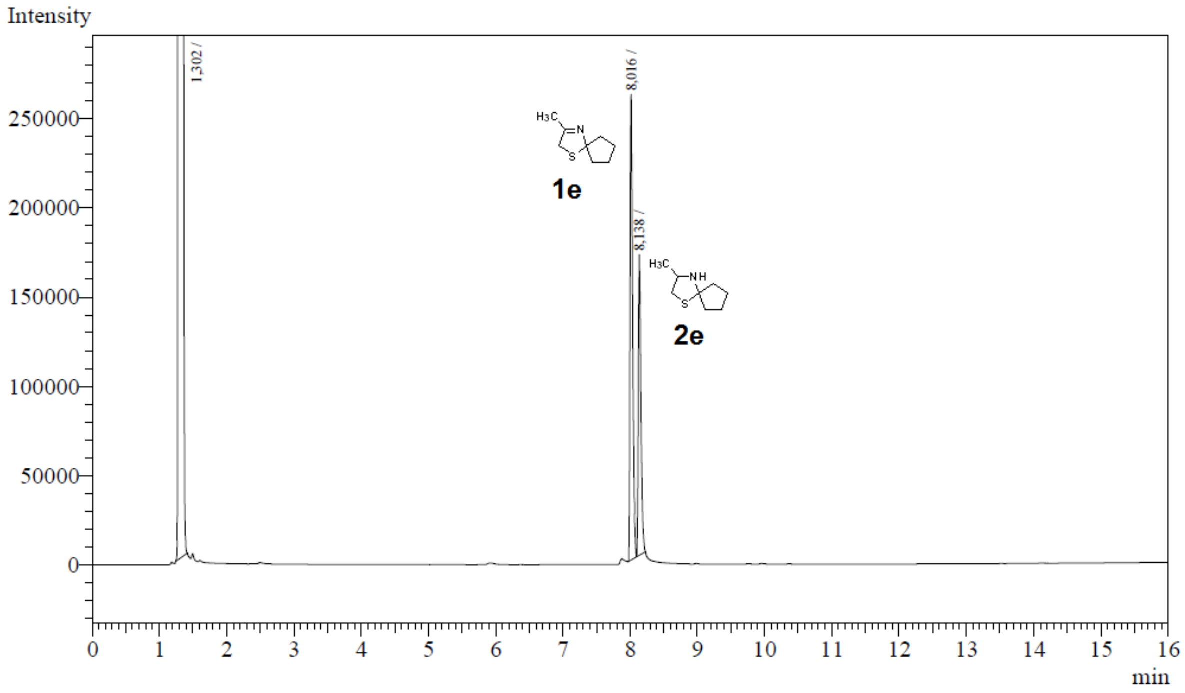

Supplementary Figure 59. Achiral GC chromatogram of 1e and rac-2e.

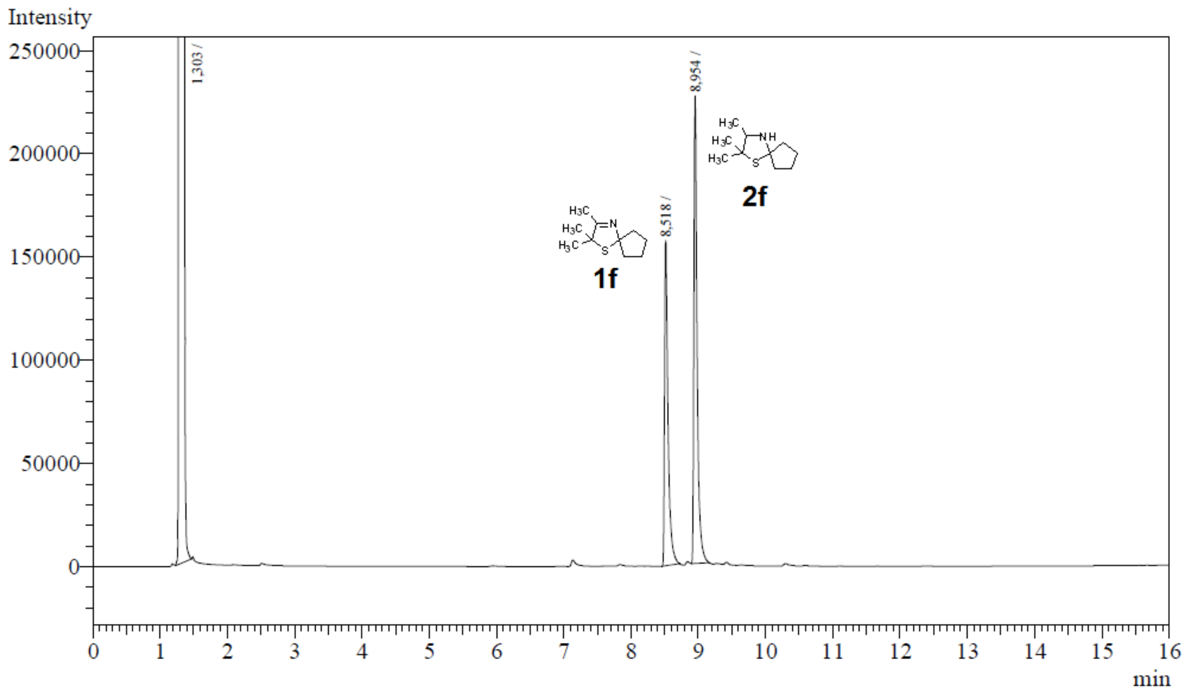

Supplementary Figure 60. Achiral GC chromatogram of 1f and rac-2f.

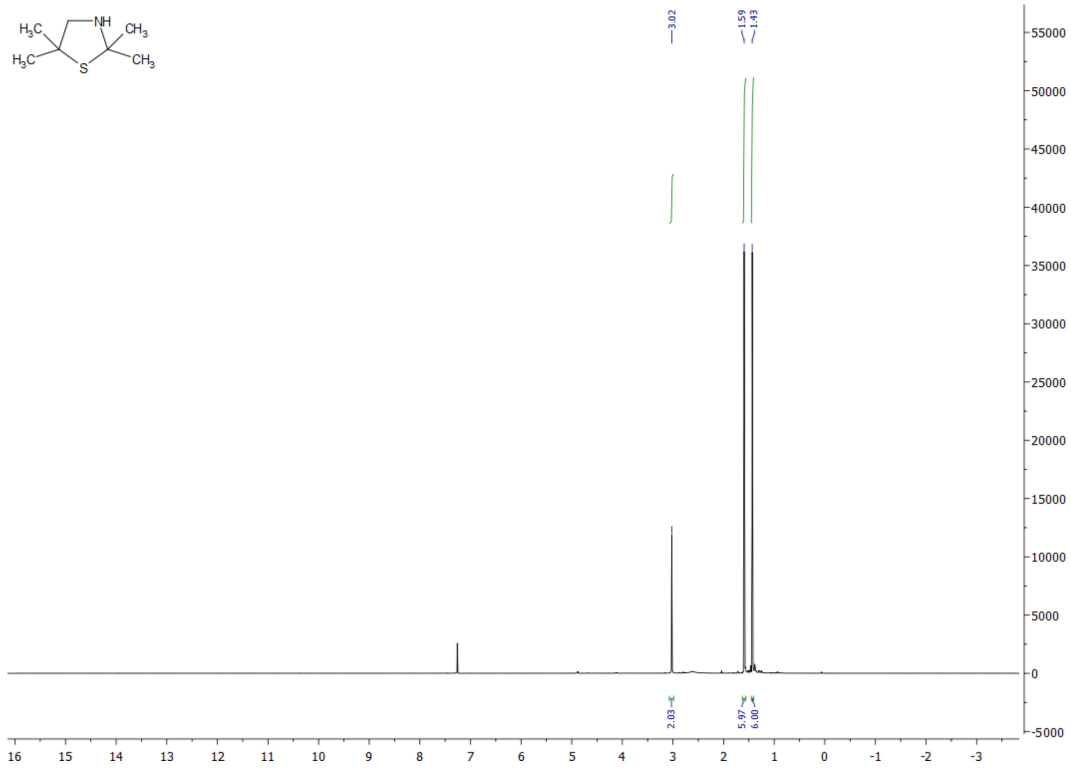

Supplementary Figure 61. ^{1}H NMR spectra of 2,2,5,5-tetramethyl-3-thiazolidine (2b).

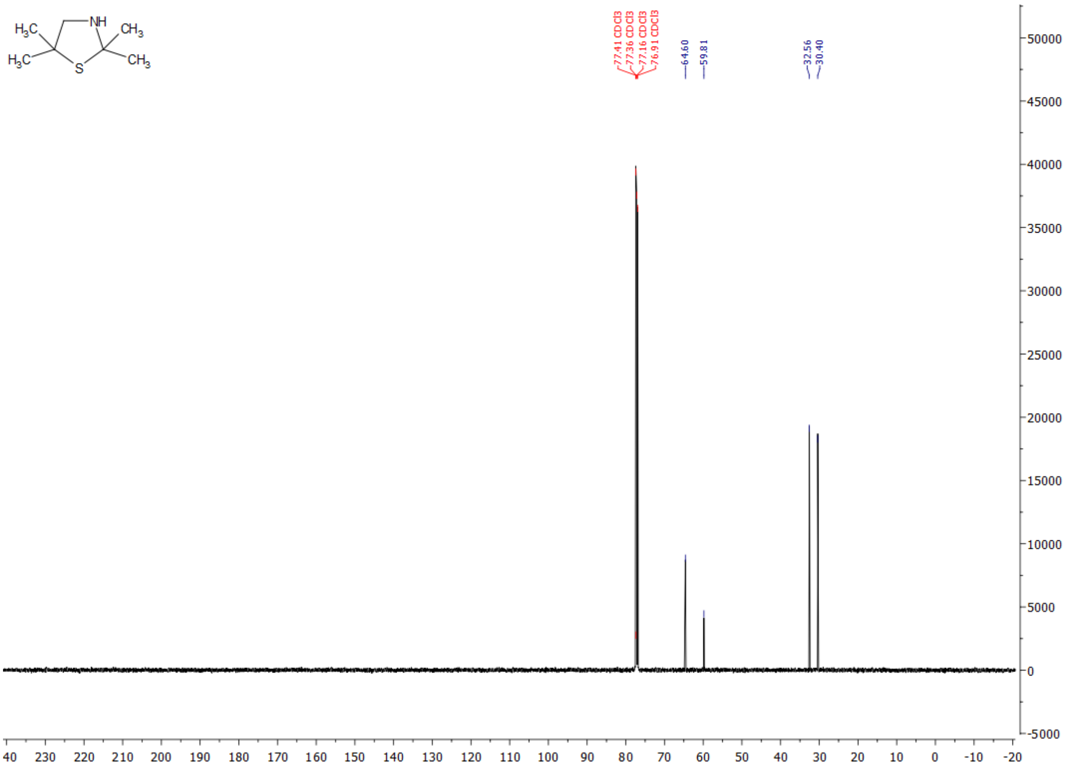

Supplementary Figure 62. ^{13}**C NMR spectra of 2,2,5,5-tetramethyl-3-thiazolidine (2b).**

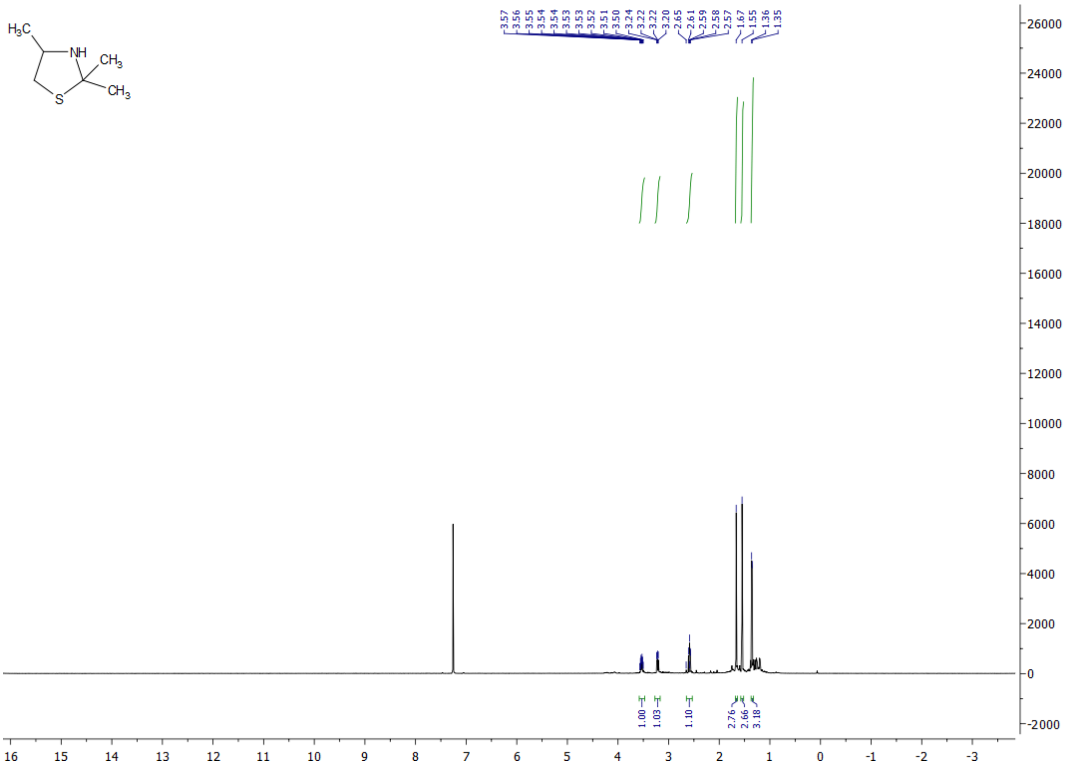

Supplementary Figure 63. ^{1}**H NMR spectra of rac-2,2,4-trimethyl-3-thiazolidine (2c).**

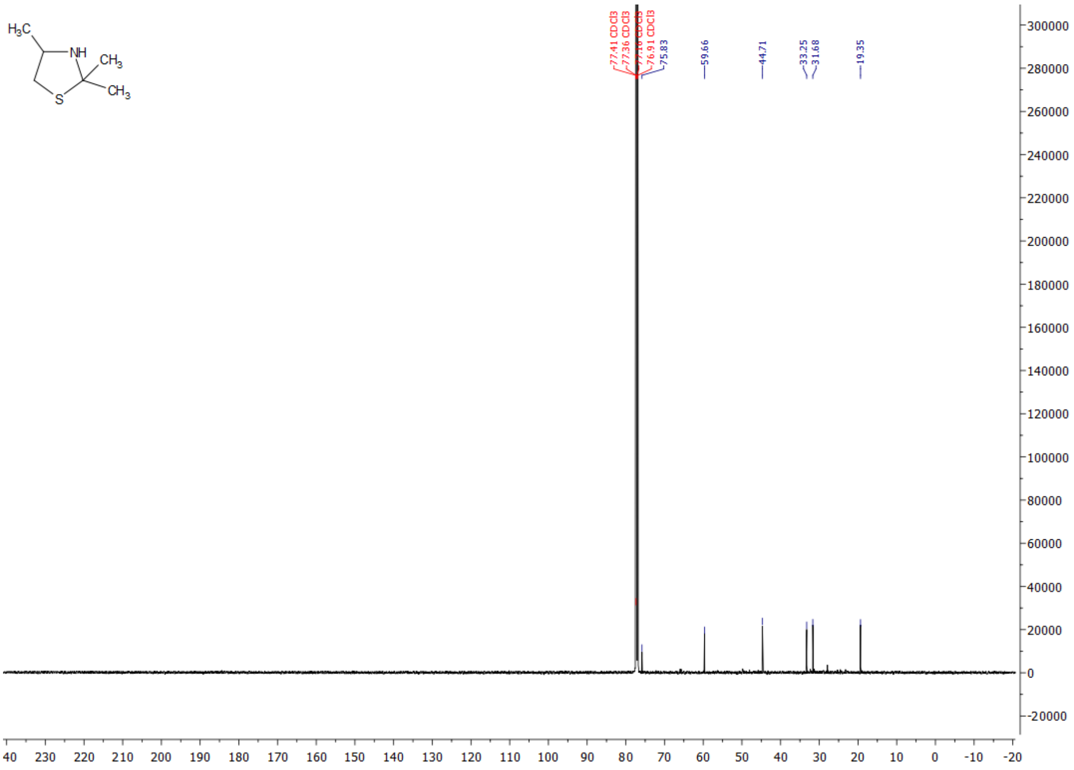

Supplementary Figure 64. ^{13}C NMR spectra of rac-2,2,4-trimethyl-3-thiazolidine (2c).

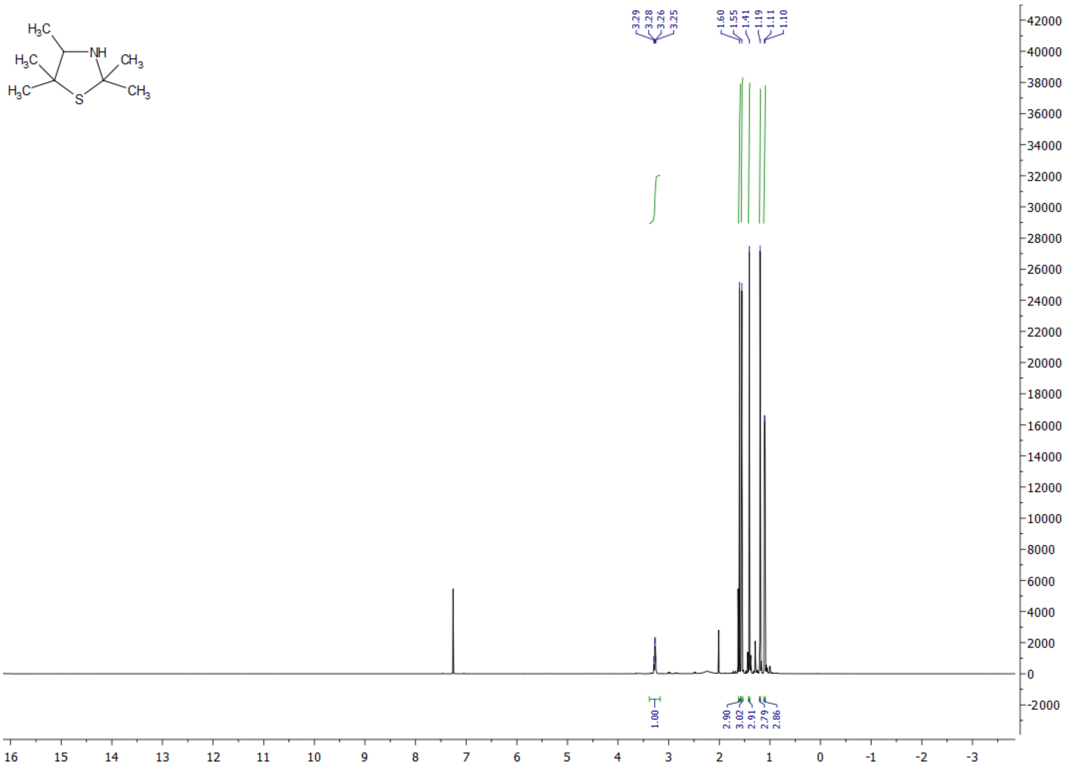

Supplementary Figure 65. ^{1}H NMR spectra of rac-2d.

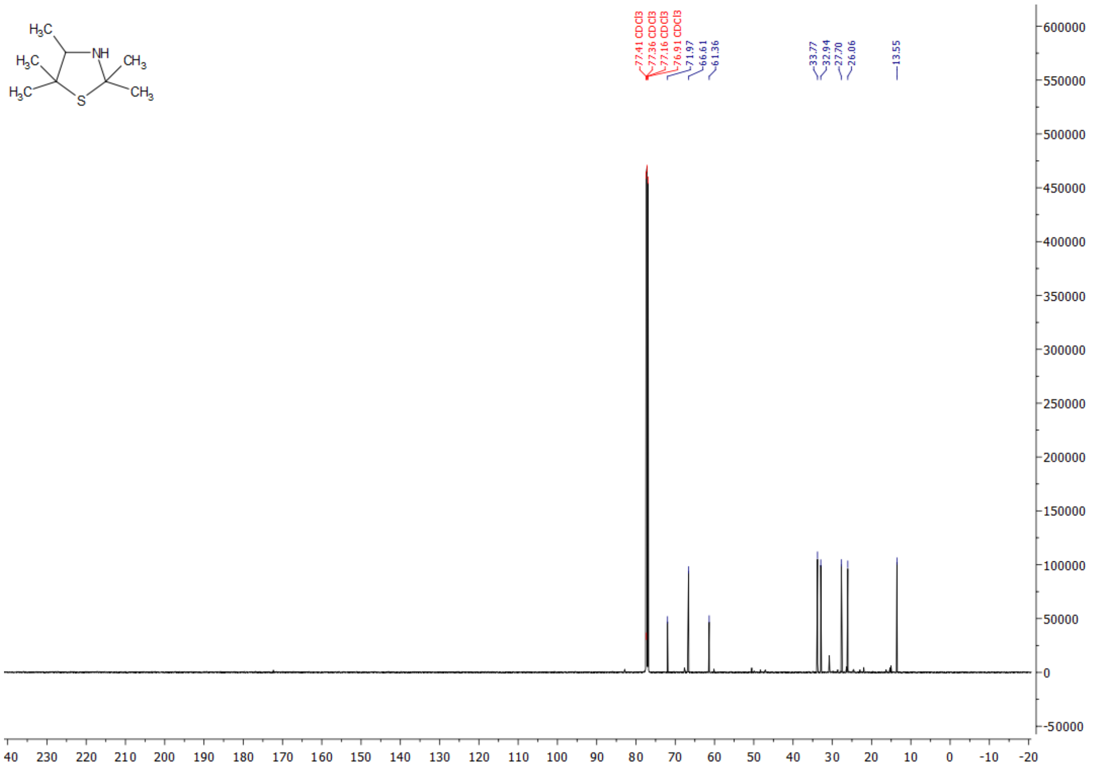

Supplementary Figure 66. ^{13}C NMR spectra of rac-2d.

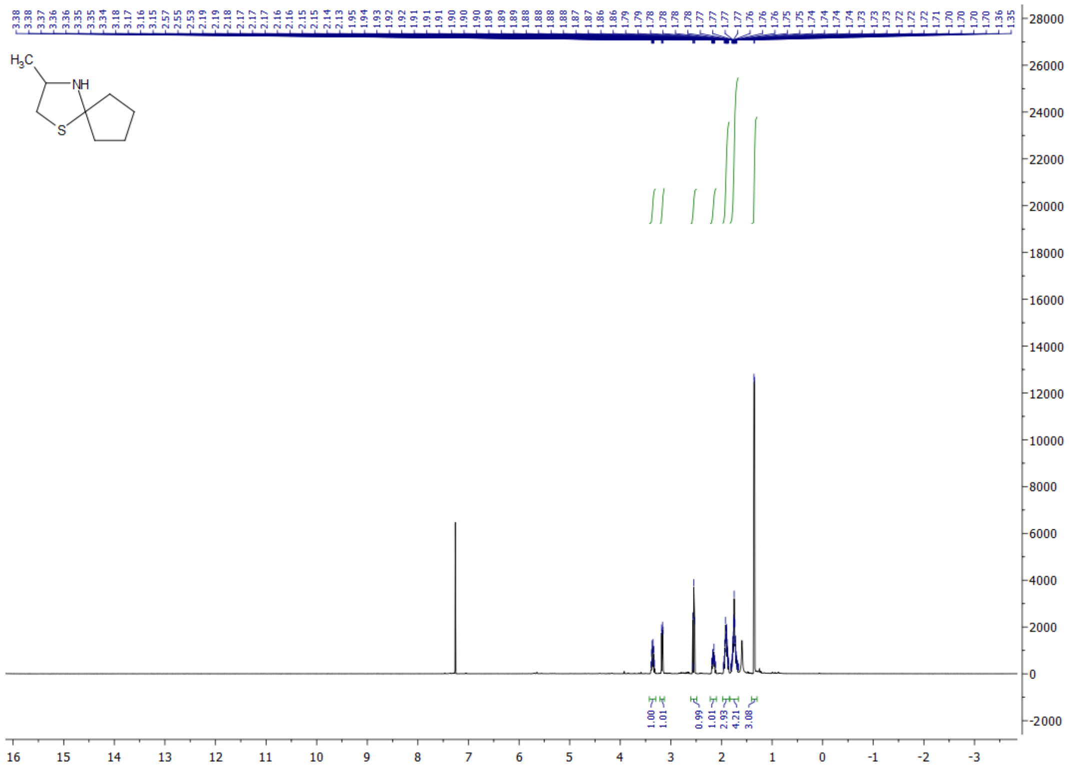

Supplementary Figure 67. ^{1}H NMR spectra of rac-2e.

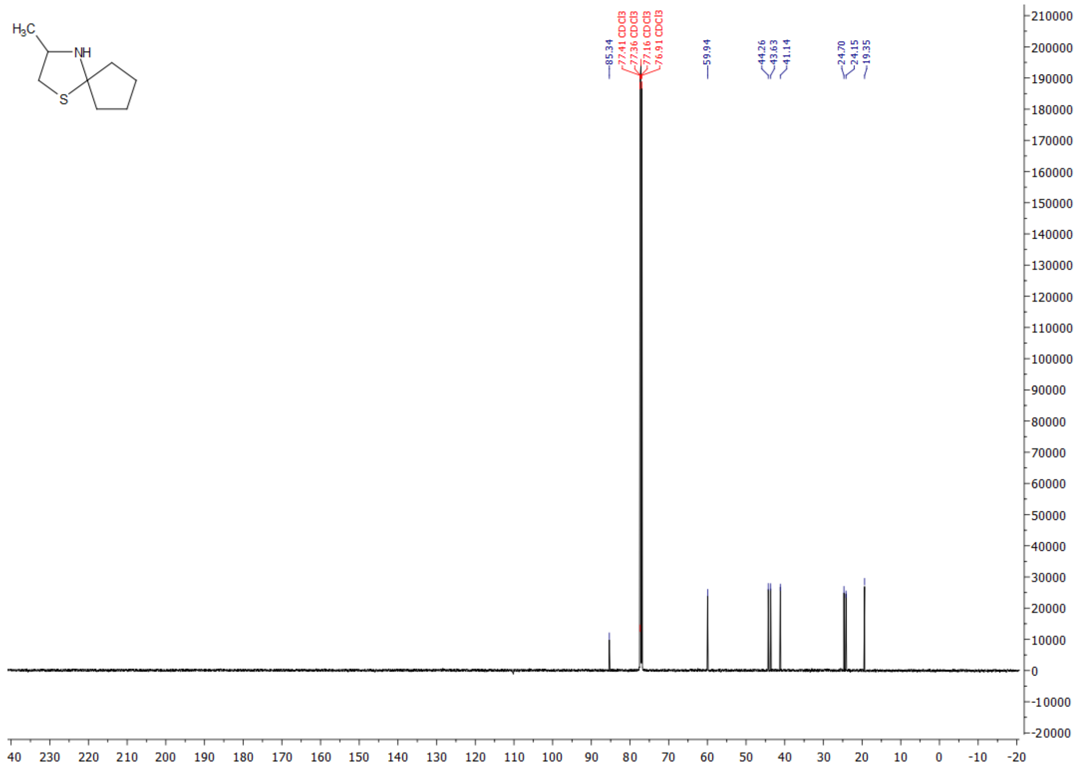

Supplementary Figure 68. ^{13}C NMR spectra of rac-2e.

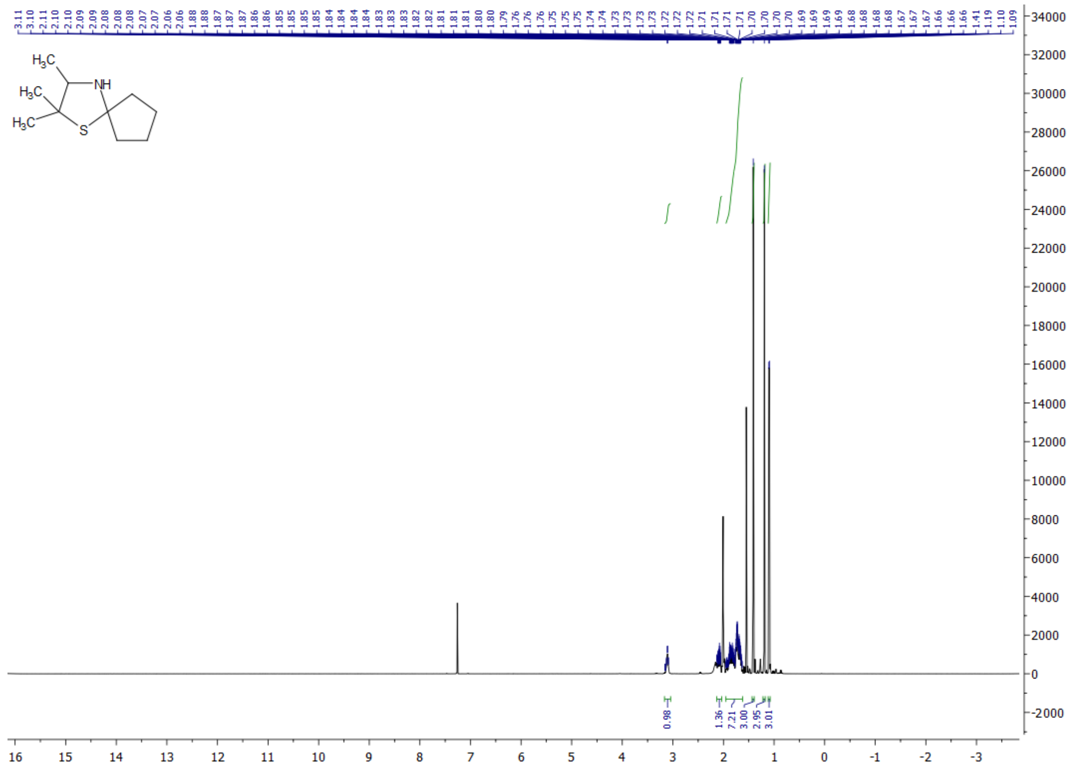

Supplementary Figure 69. ^{1}H NMR spectra of rac-2f.

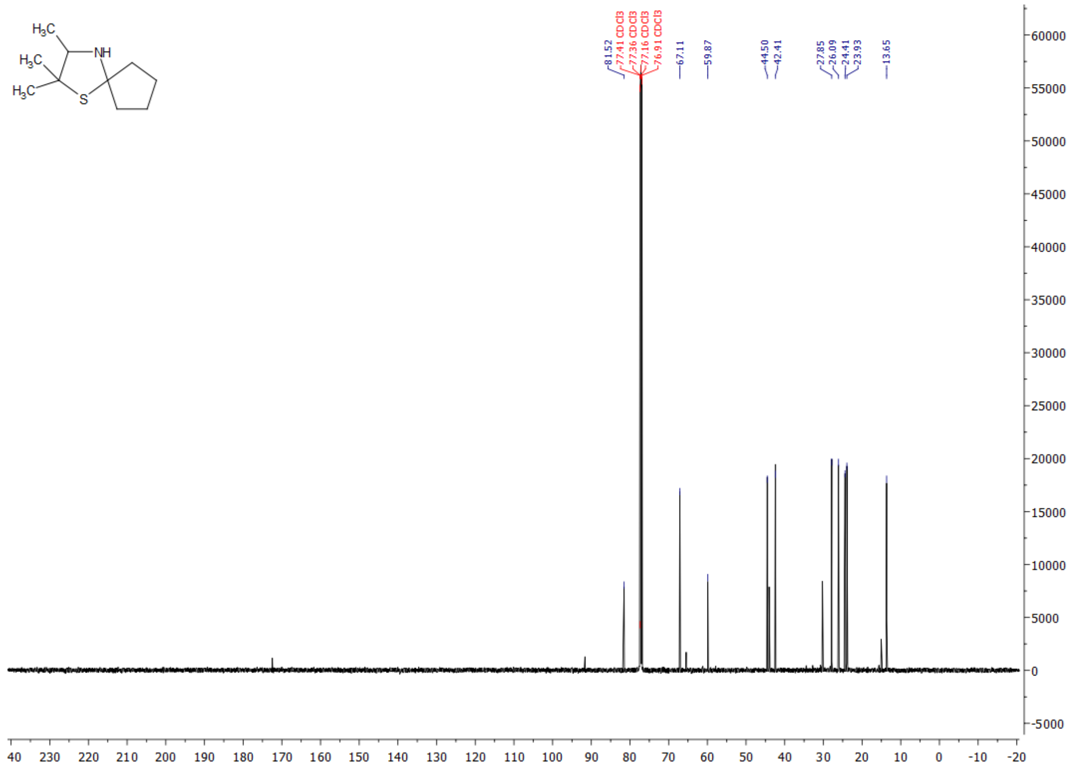

Supplementary Figure 70. ^{13}C NMR spectra of rac-2f.

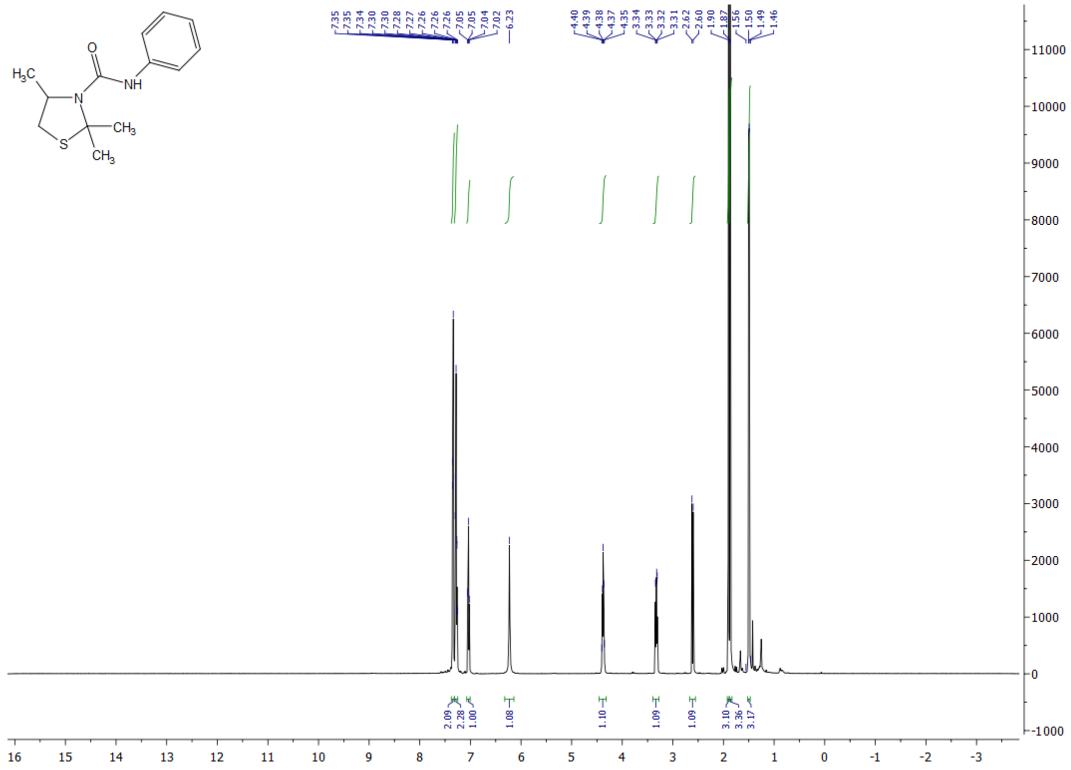

Supplementary Figure 71. ^{1}H NMR spectra of rac-5c.

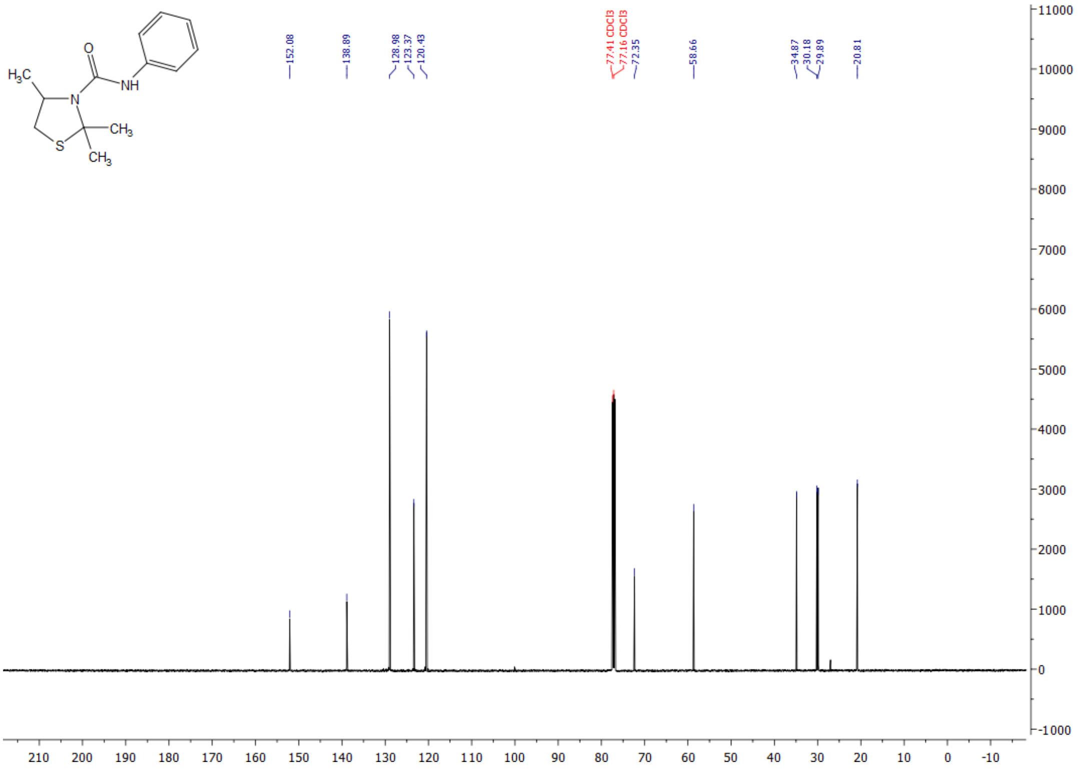

Supplementary Figure 72. ^{13}C NMR spectra of rac-5c.

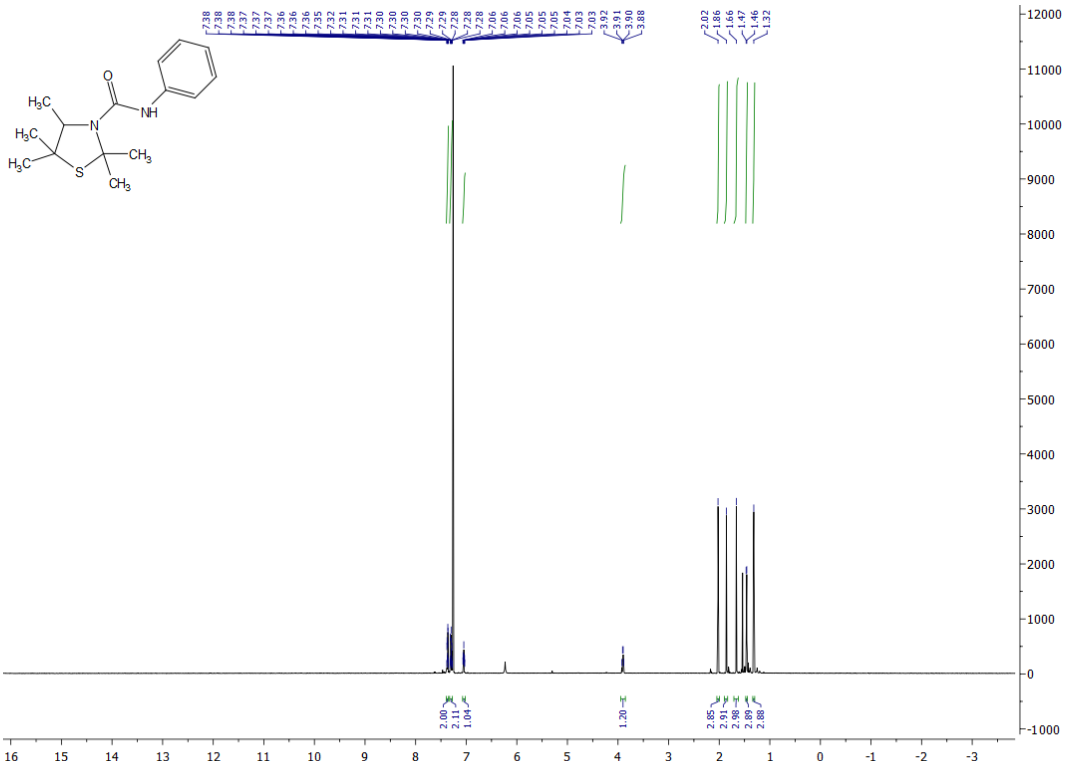

Supplementary Figure 73. ^{1}H NMR spectra of rac-5d.

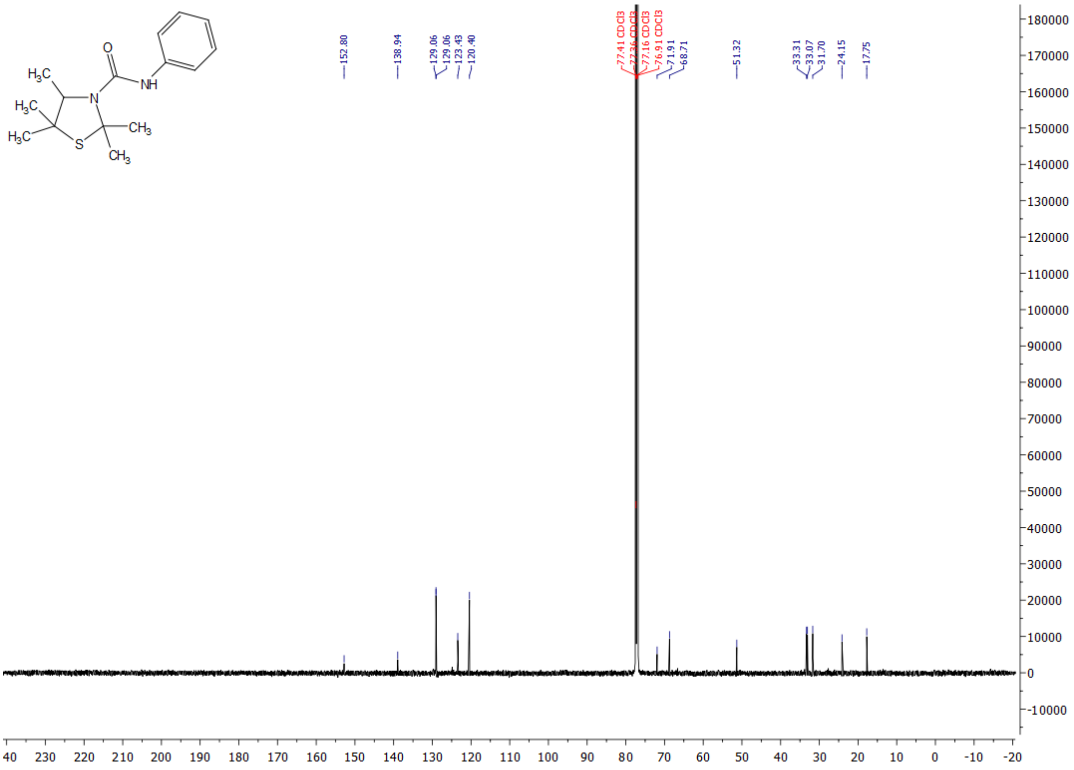

Supplementary Figure 74. ^{13}**C NMR spectra of rac-5d.**

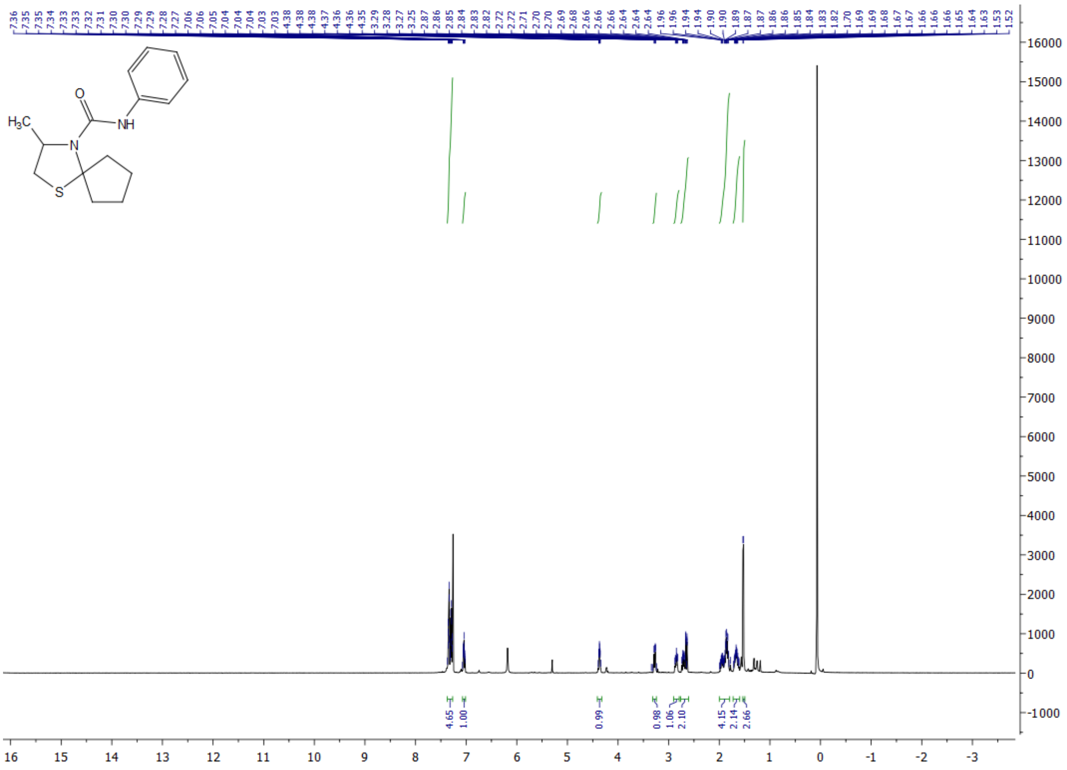

Supplementary Figure 75. ^{1}**H NMR spectra of rac-5e.**

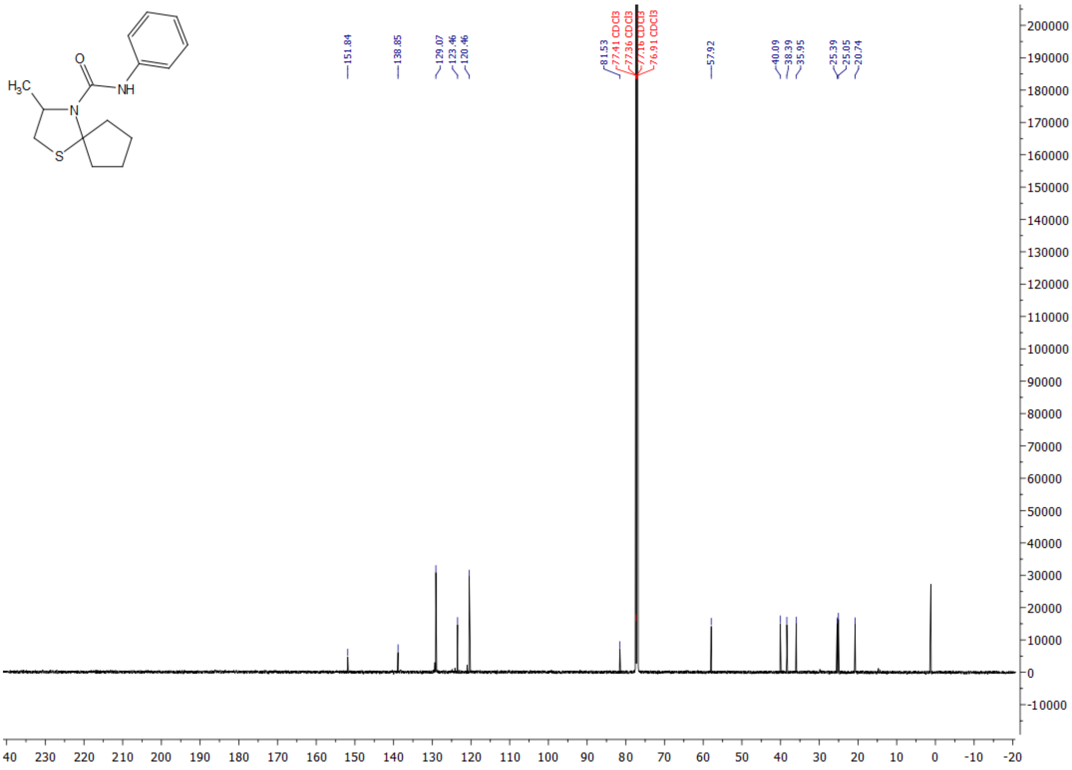

Supplementary Figure 76. ^{13}**C NMR spectra of rac-5e.**

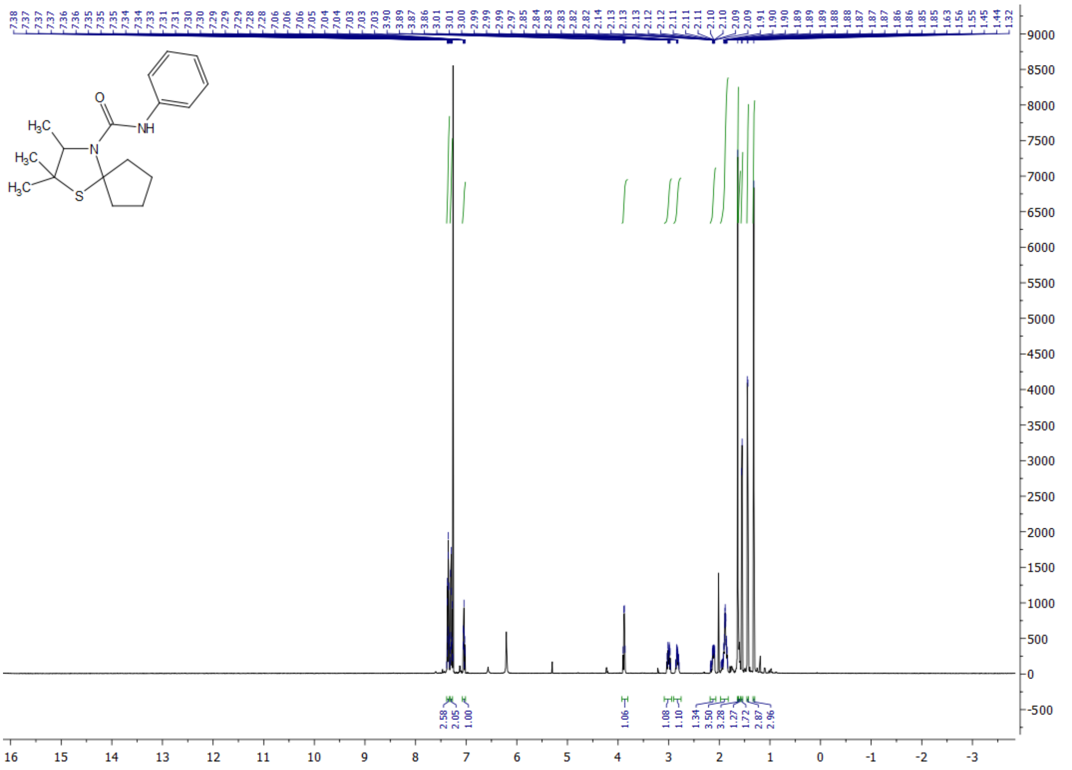

Supplementary Figure 77. ^{1}**H NMR spectra of rac-5f.**

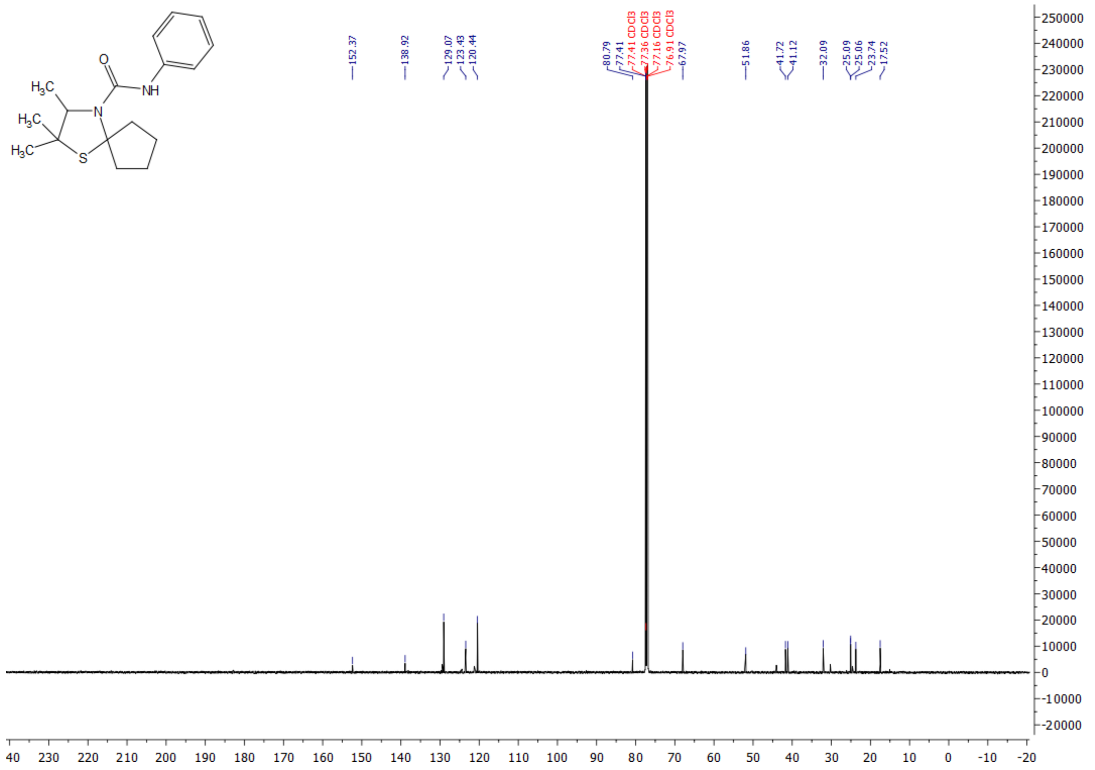

Supplementary Figure 78. ^{13}C NMR spectra of rac-5f.

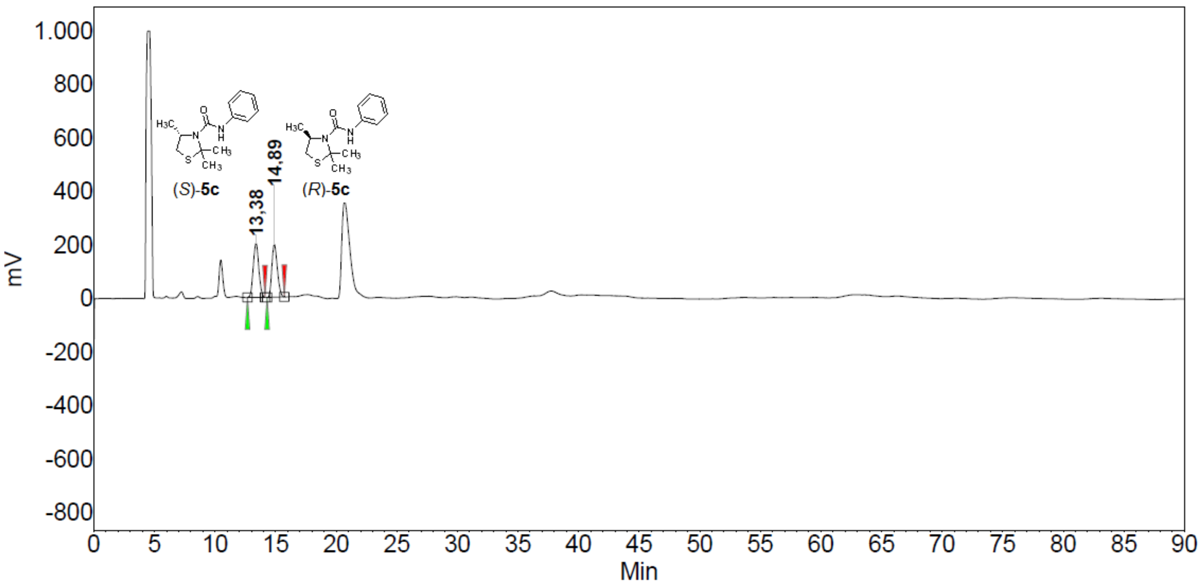

Supplementary Figure 79. Chiral HPLC chromatogram of (*S*)-5c and (*R*)-5c.

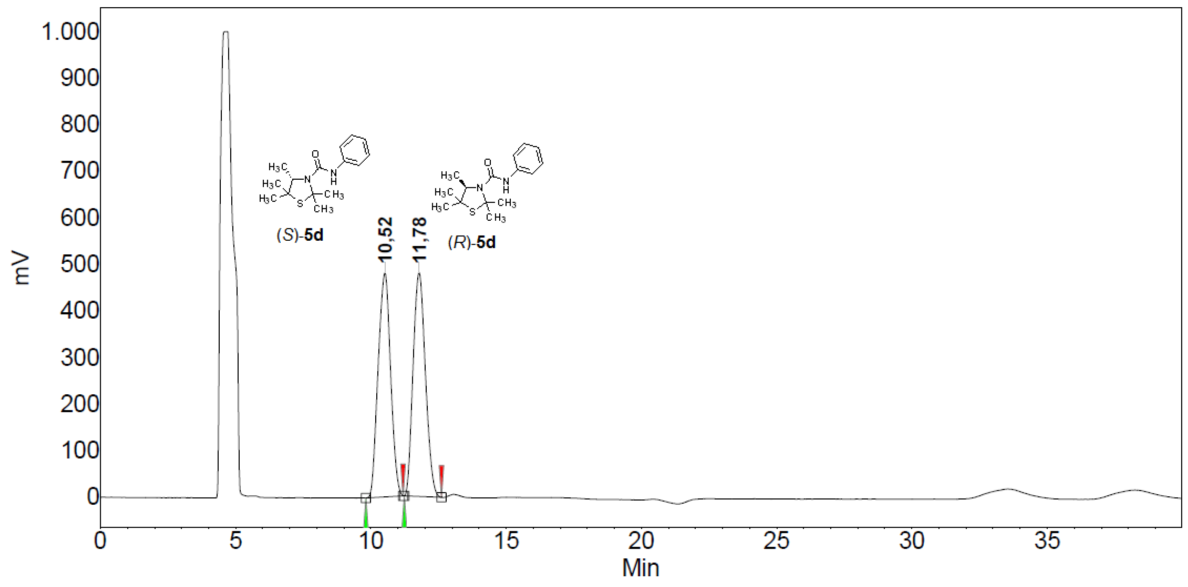

Supplementary Figure 80. Chiral HPLC chromatogram of (*S*)-5d and (*R*)-5d.

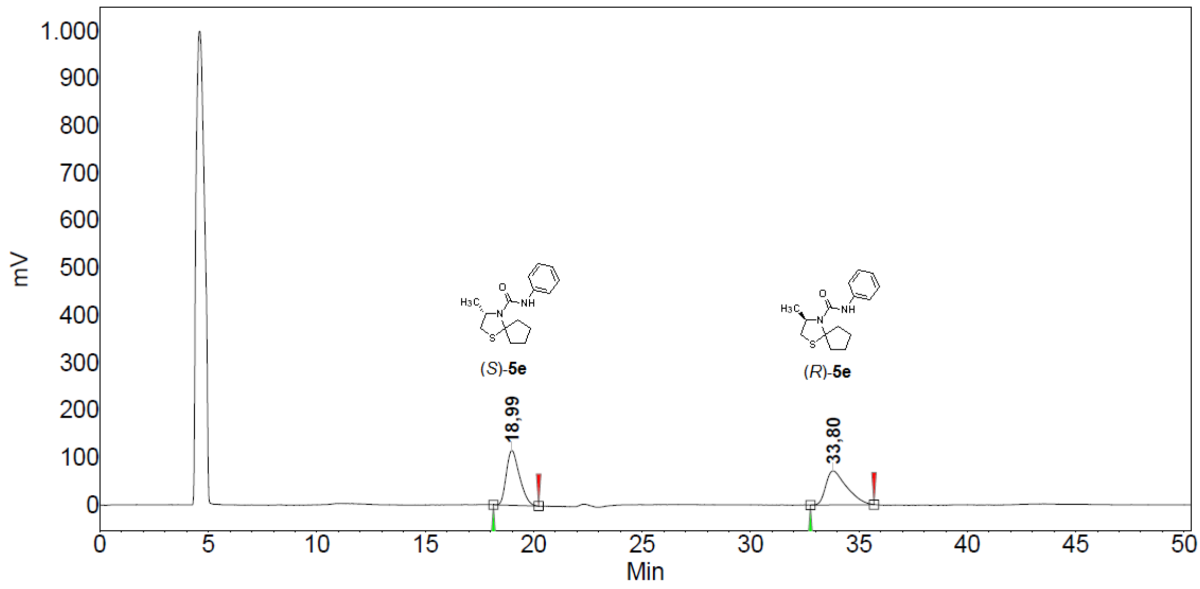

Supplementary Figure 81. Chiral HPLC chromatogram of (*S*)-5e and (*R*)-5e.

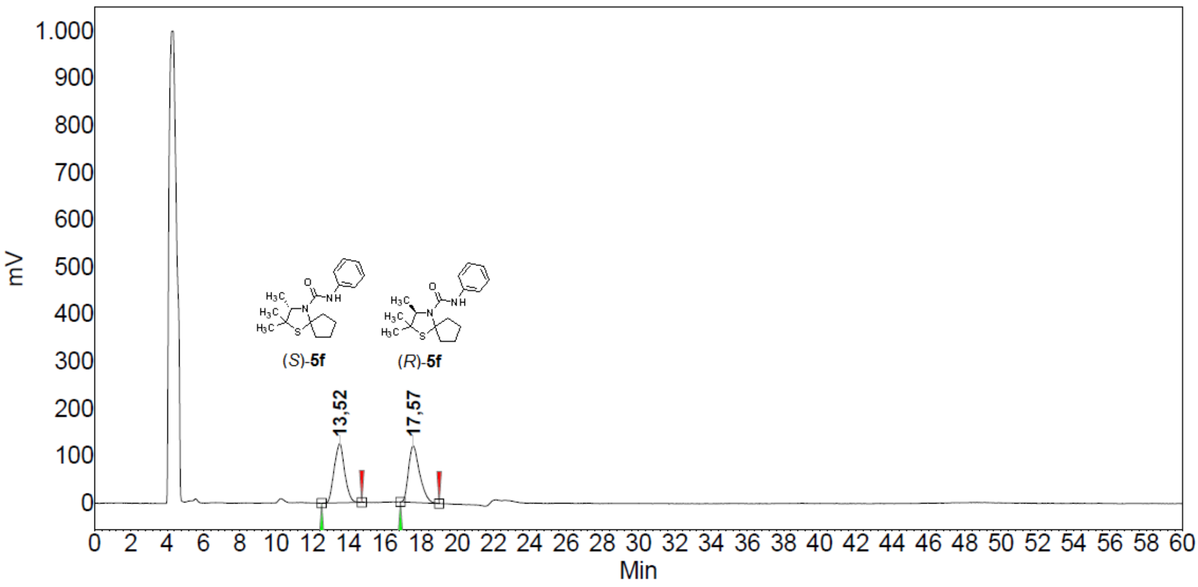

Supplementary Figure 82. Chiral HPLC chromatogram of (*S*)-5f and (*R*)-5f.

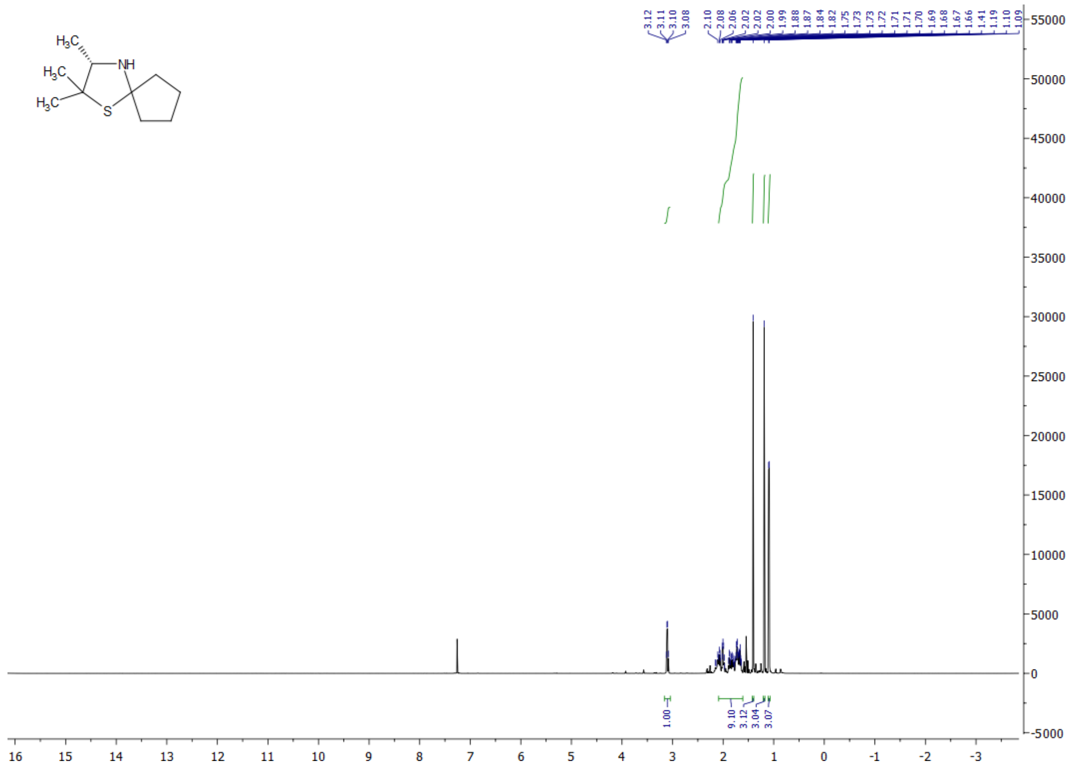

Supplementary Figure 83. ^{1}H NMR spectra of (*S*)-2f.

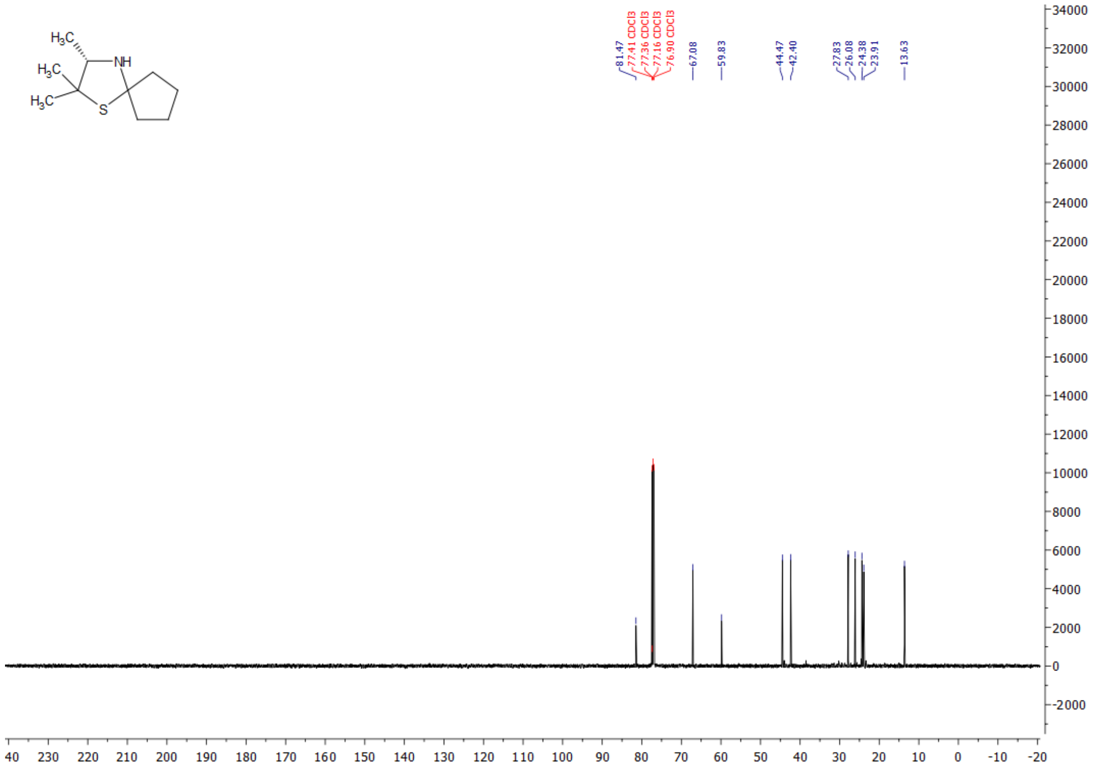

Supplementary Figure 84. ^{13}**C NMR spectra of (*S*)-2f.**

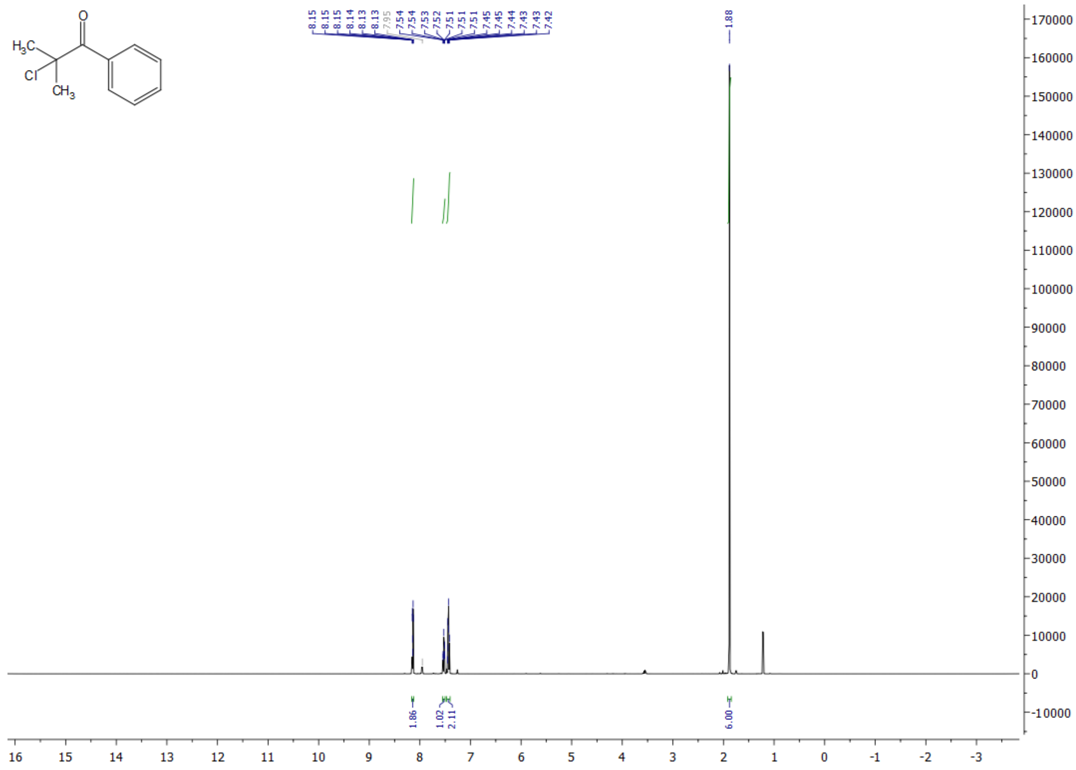

Supplementary Figure 85. ^{1}**H NMR spectra of 2-chloro-2-methyl-1-phenyl-1-propanone.**

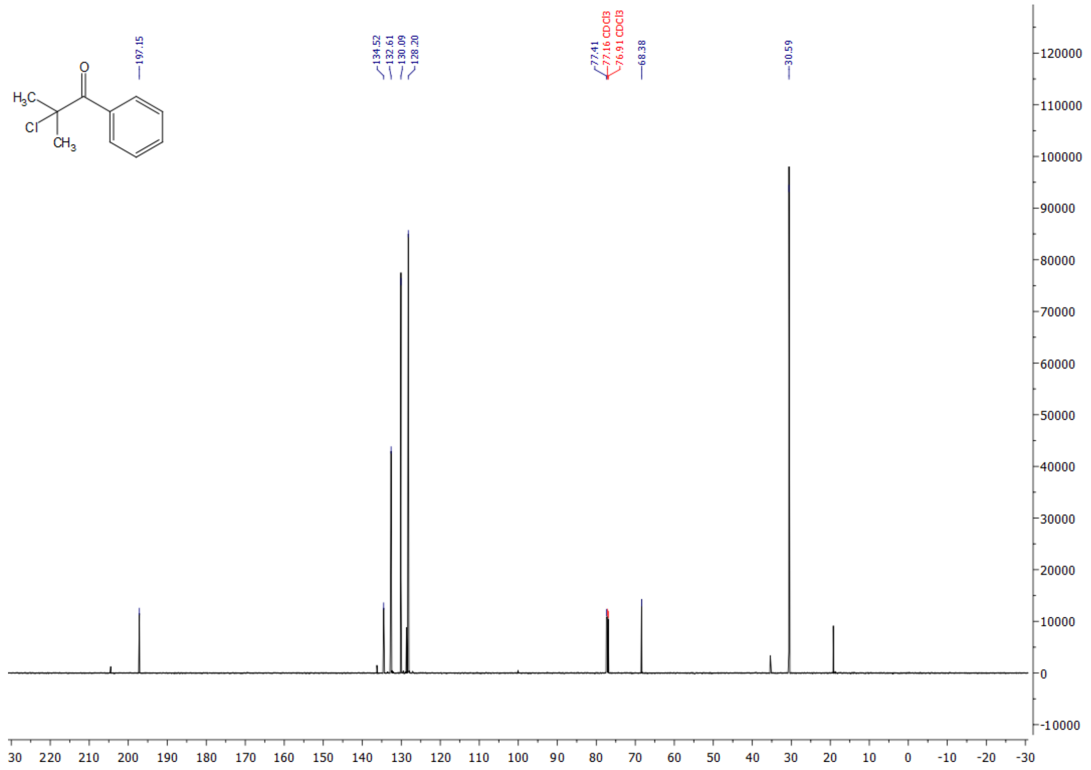

Supplementary Figure 86. ^{13}C NMR spectra of 2-chloro-2-methyl-1-phenyl-1-propanone.

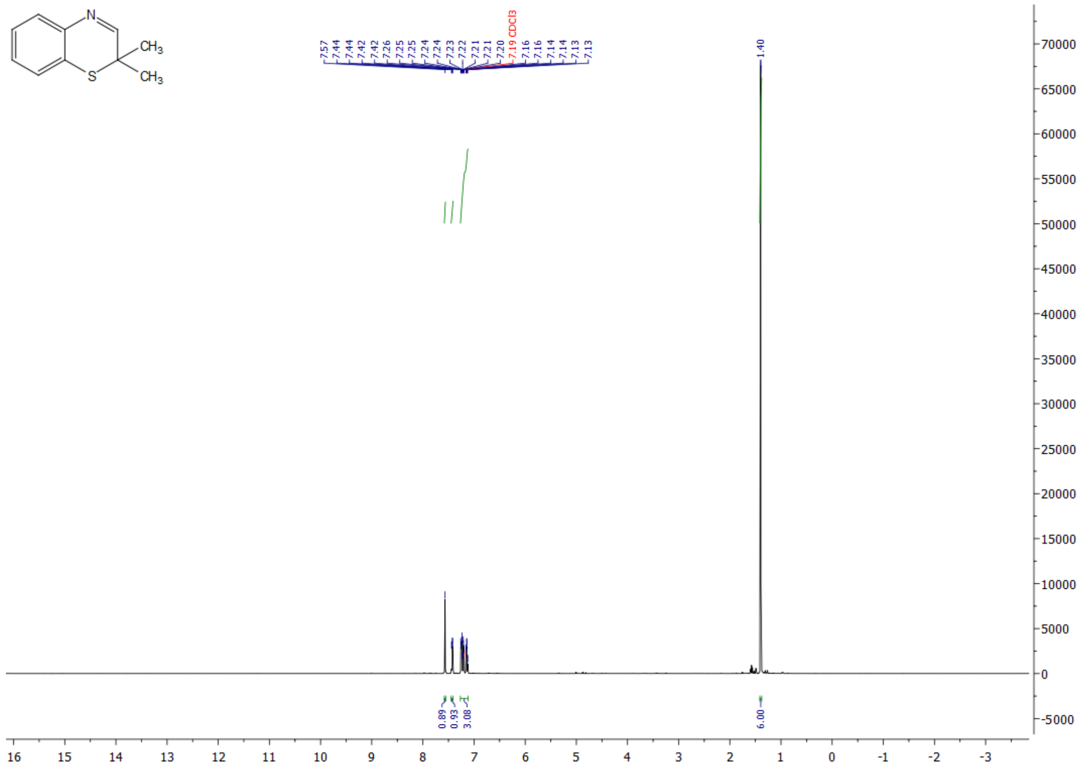

Supplementary Figure 87. ^{1}H NMR spectra of 2,2-dimethyl-*2H*-1,4-benzothiazine (3a).

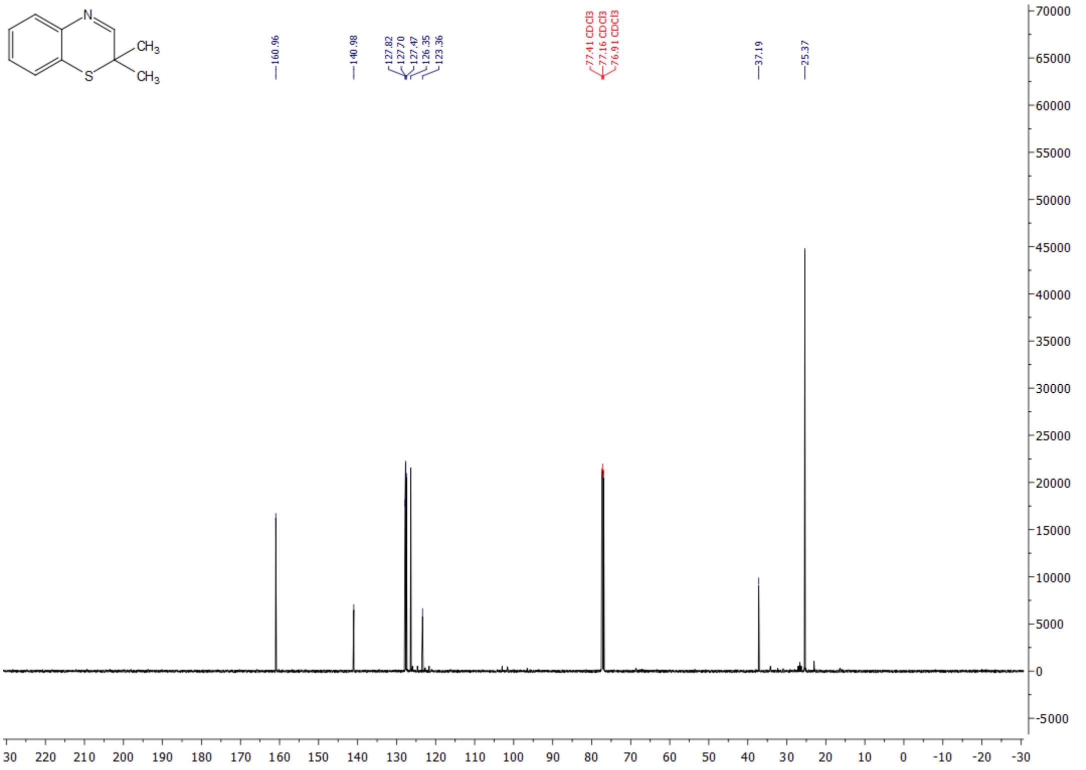

Supplementary Figure 88. ^{13}C NMR spectra of 2,2-dimethyl-*2H*-1,4-benzothiazine (3a).

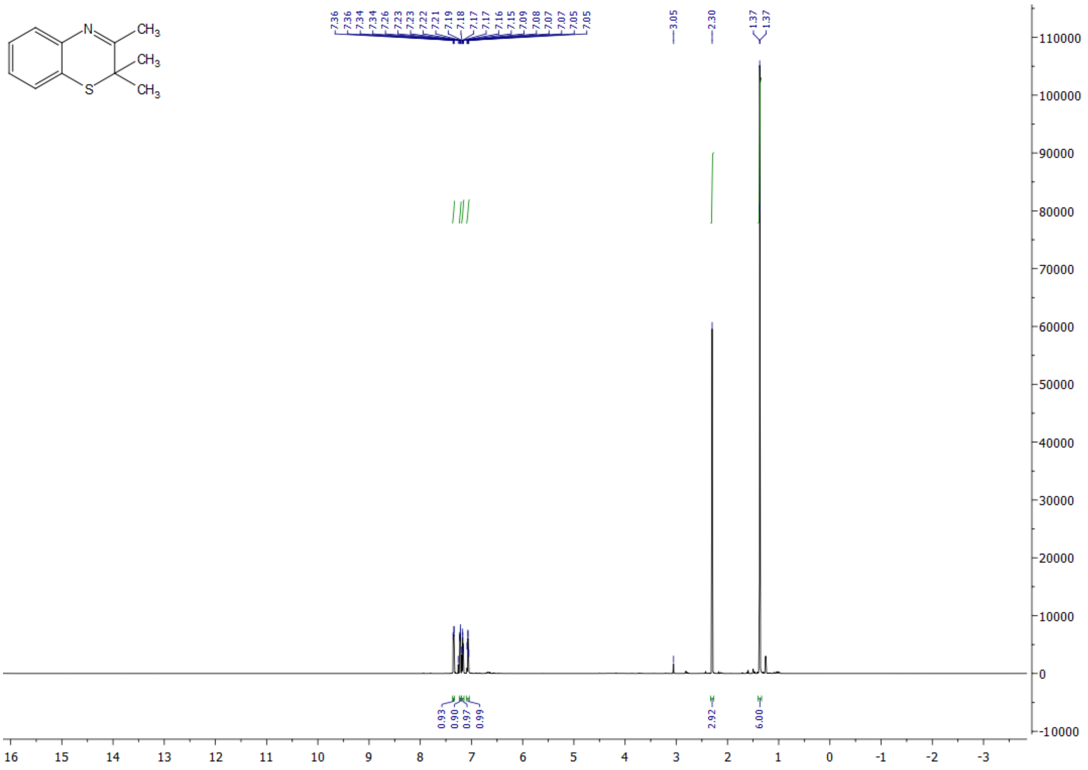

Supplementary Figure 89. ^{1}H NMR spectra of 2,2,3-trimethyl-*2H*-1,4-benzothiazine (3b).

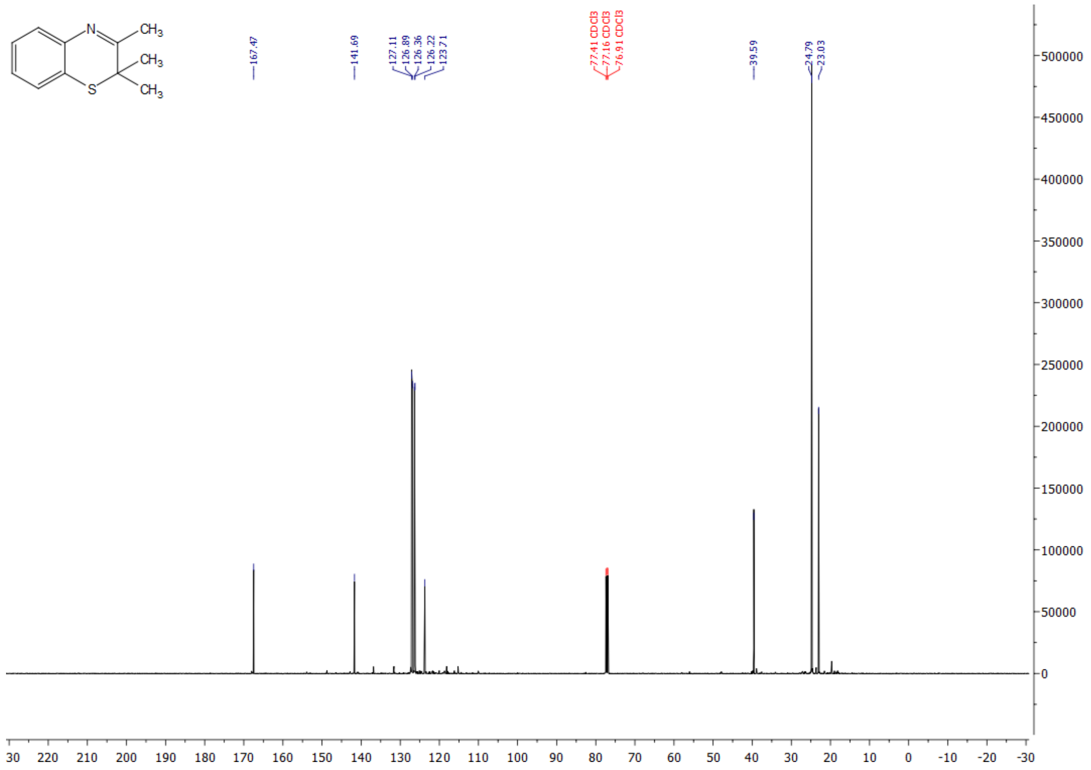

Supplementary Figure 90. ^{13}C NMR spectra of 2,2,3-trimethyl-*2H*-1,4-benzothiazine (3b).

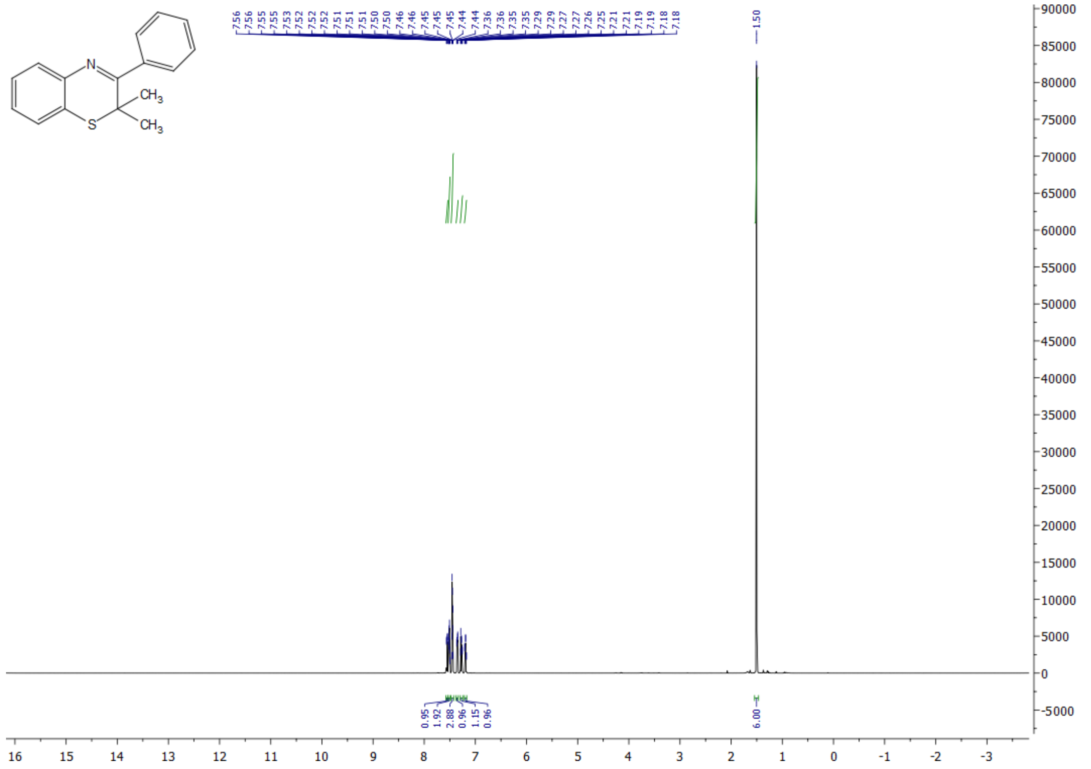

Supplementary Figure 91. ^{1}H NMR spectra of 3c.

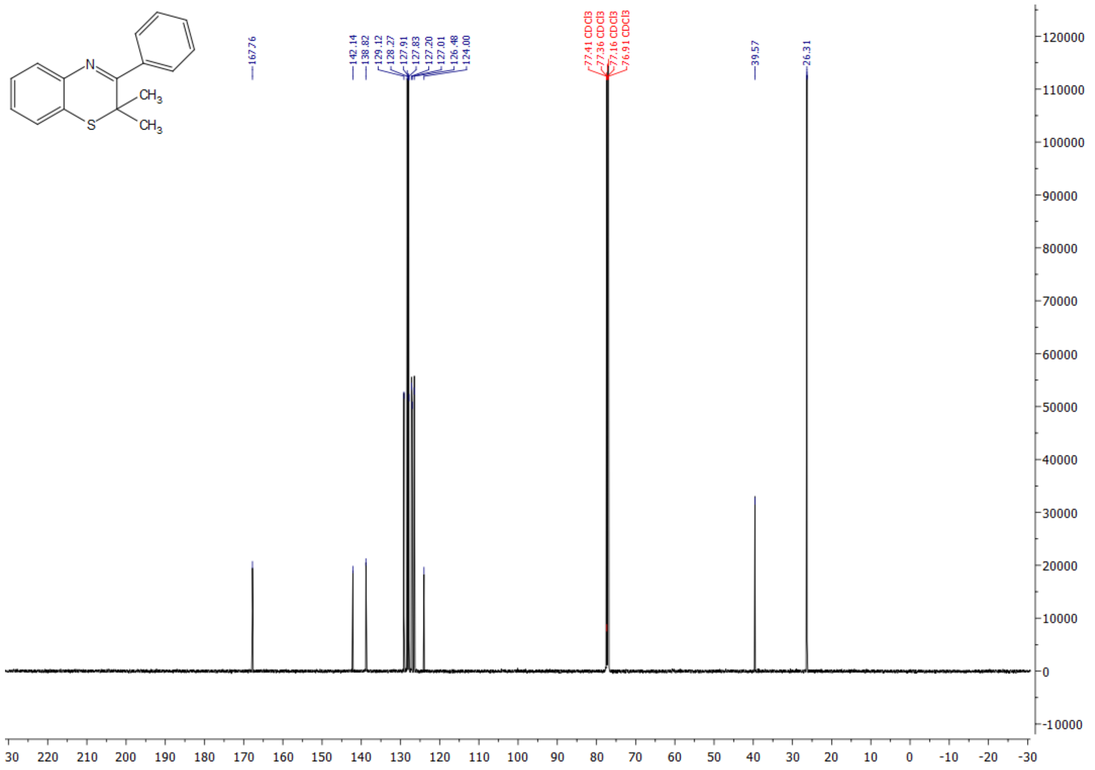

Supplementary Figure 92. ^{13}C NMR spectra of 3c.

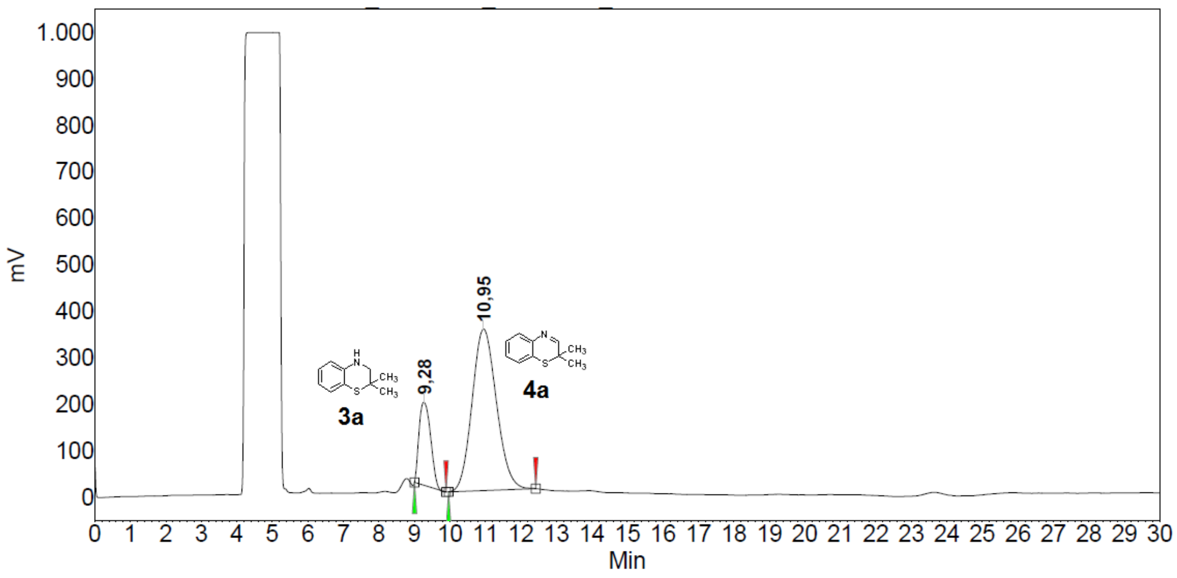

Supplementary Figure 93. HPLC chromatogram of 3a and 4a.

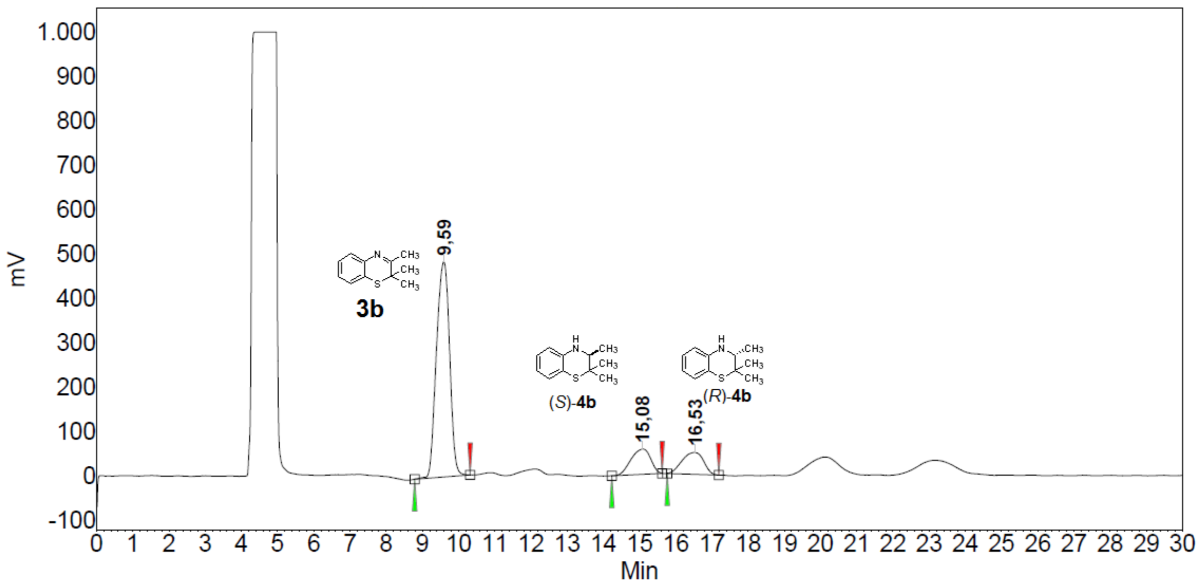

Supplementary Figure 94. Chiral HPLC chromatogram of 3b, (*S*)-4b and (*R*)-4b.

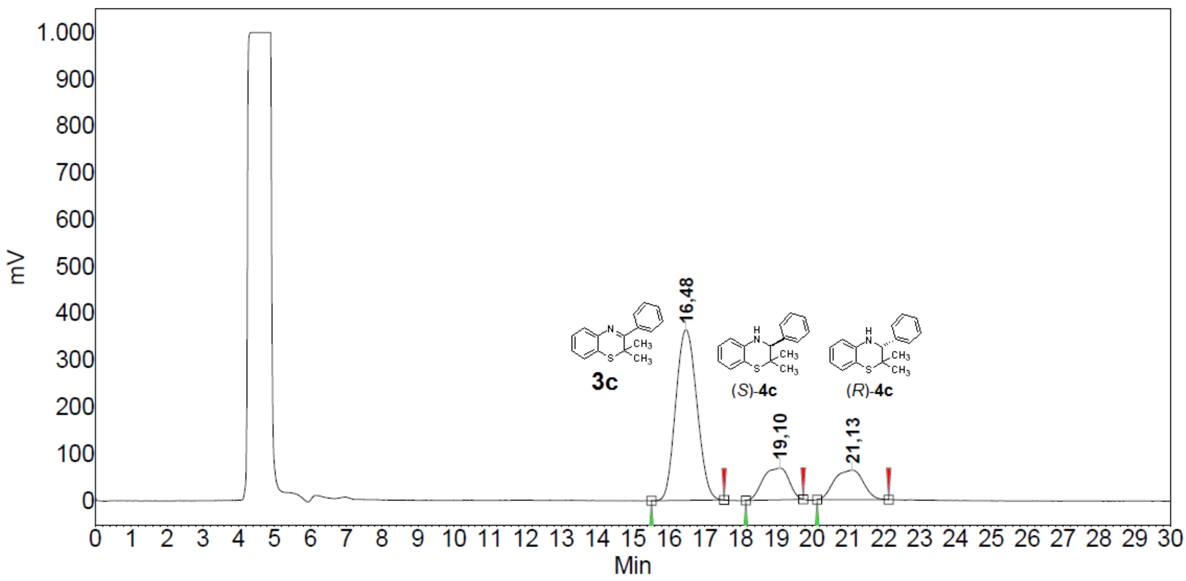

Supplementary Figure 95. Chiral HPLC chromatogram of 3b, (*S*)-4c and (*R*)-4c.

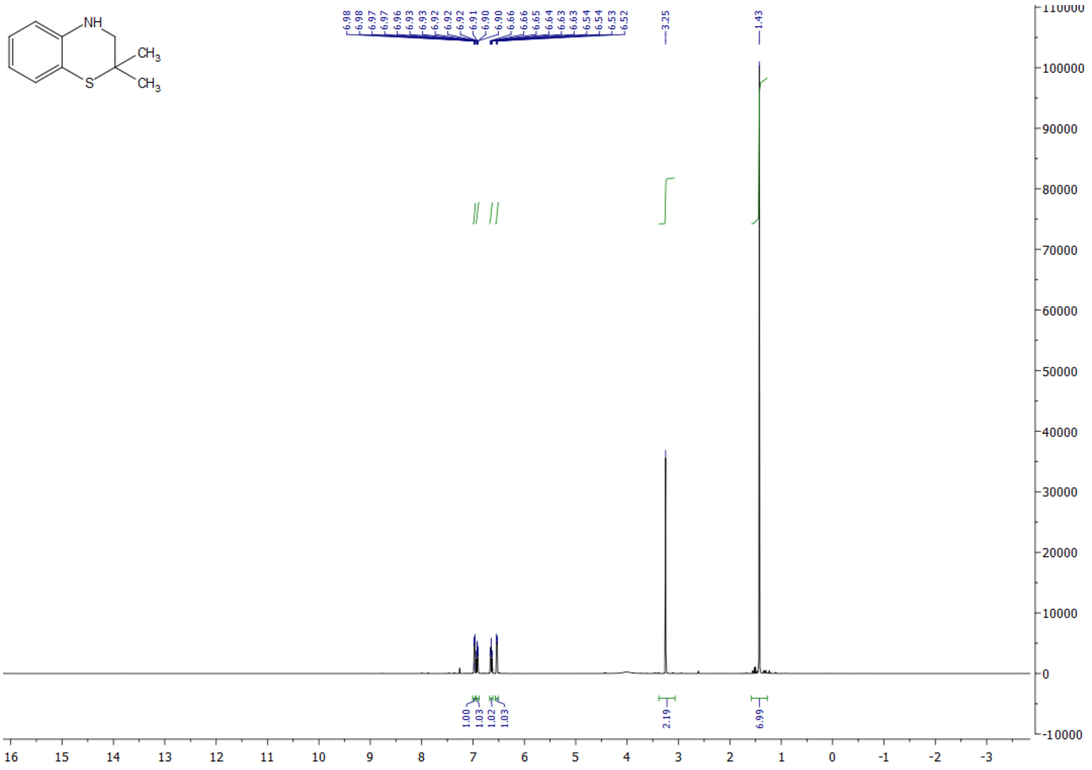

Supplementary Figure 96. ^{1}H NMR spectra of 4a.

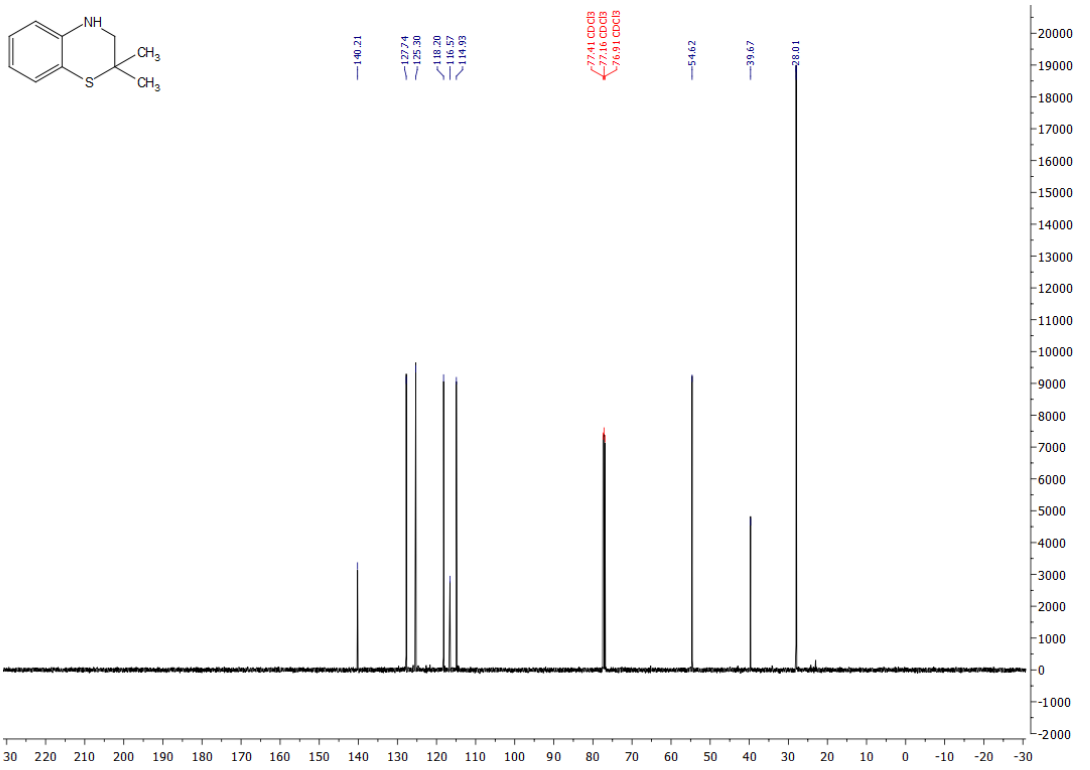

Supplementary Figure 97. ^{13}C NMR spectra of 4a.

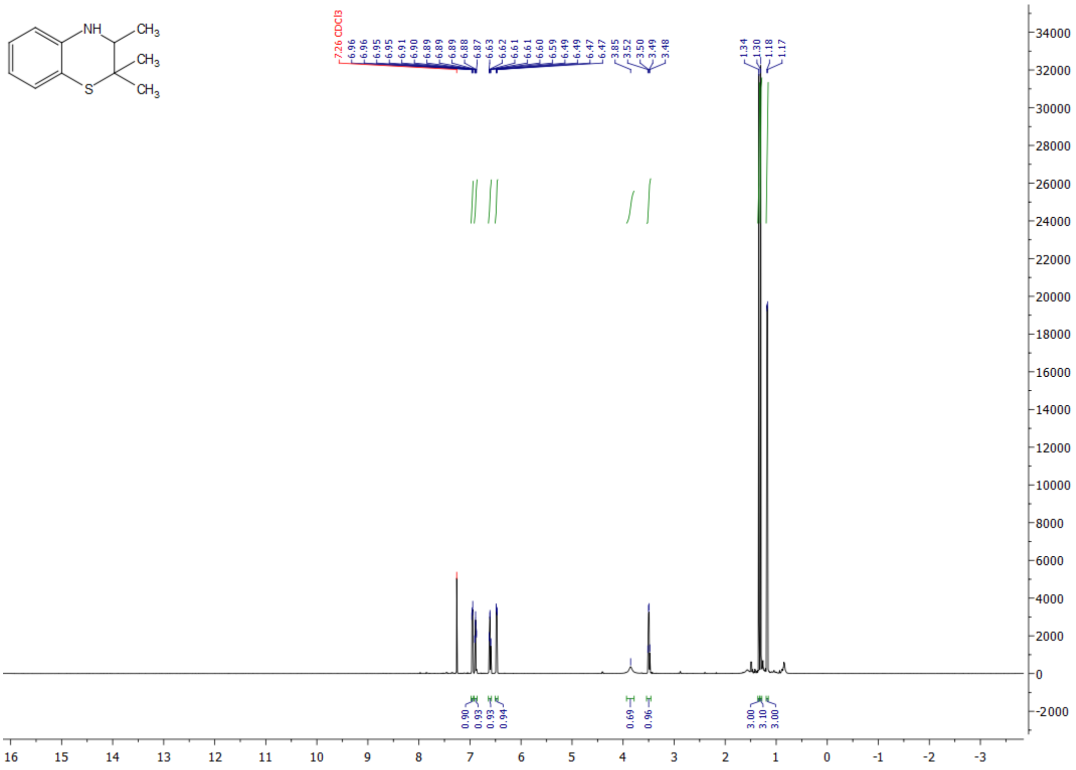

Supplementary Figure 98. ^{1}H NMR spectra of rac-4b.

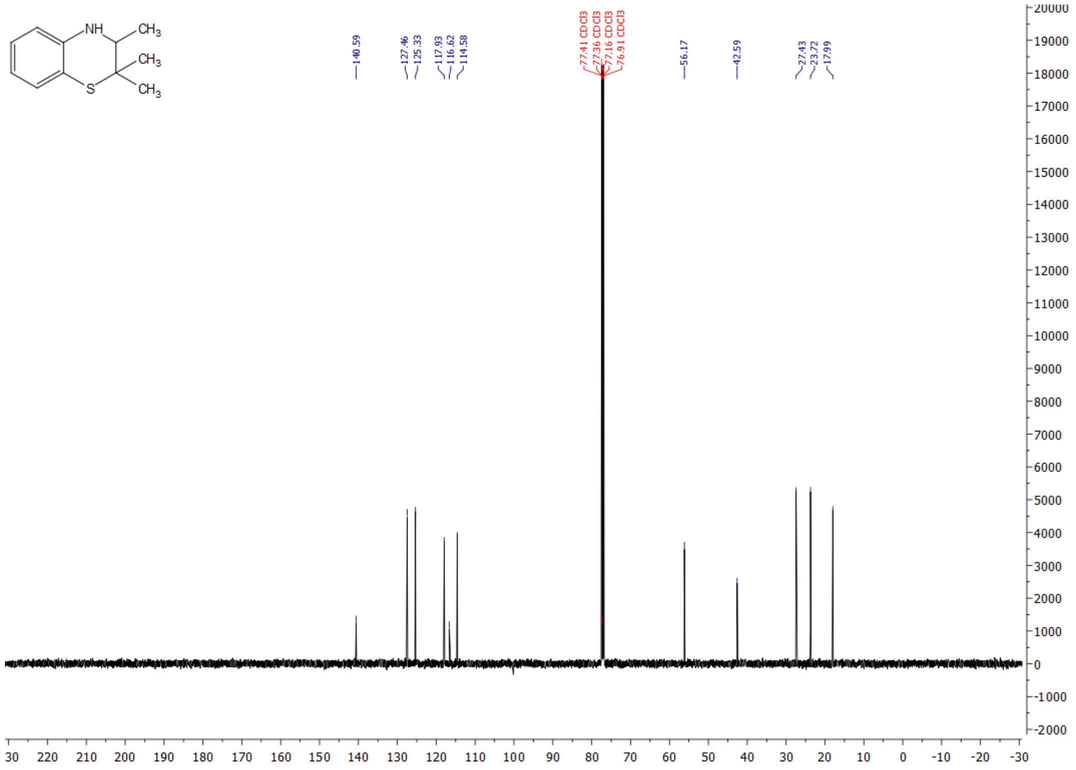

Supplementary Figure 99. ^{13}C NMR spectra of rac-4b.

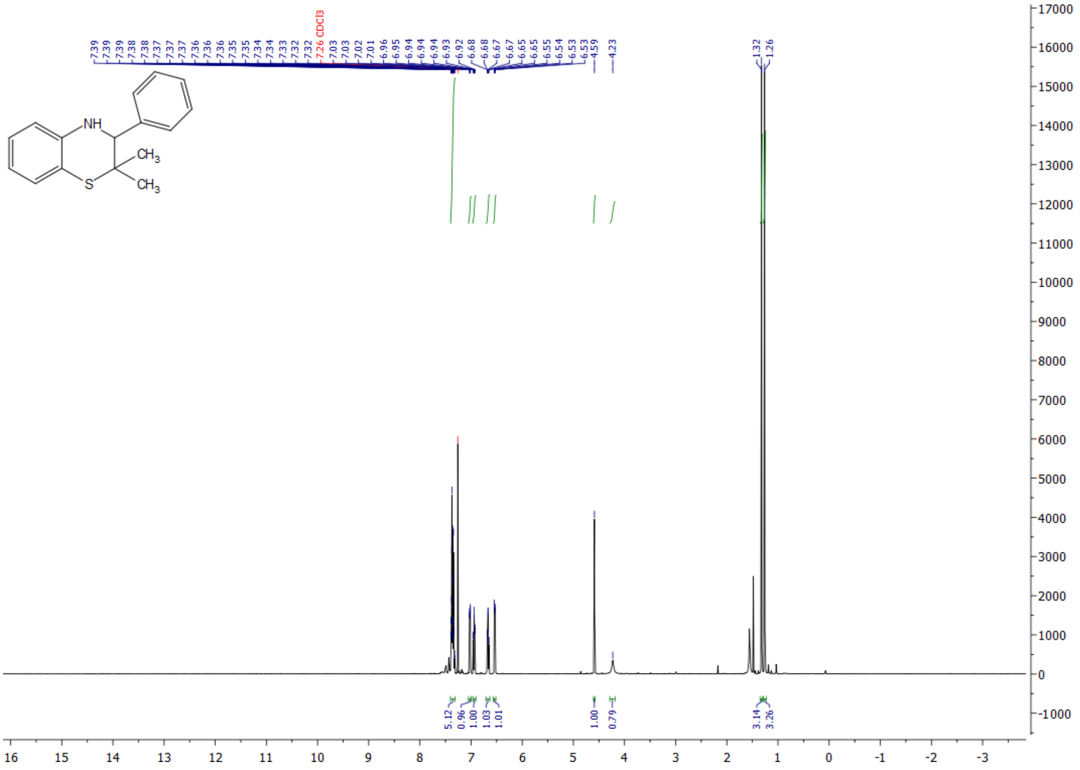

Supplementary Figure 100. ^{1}H **NMR spectra of rac-4c.**

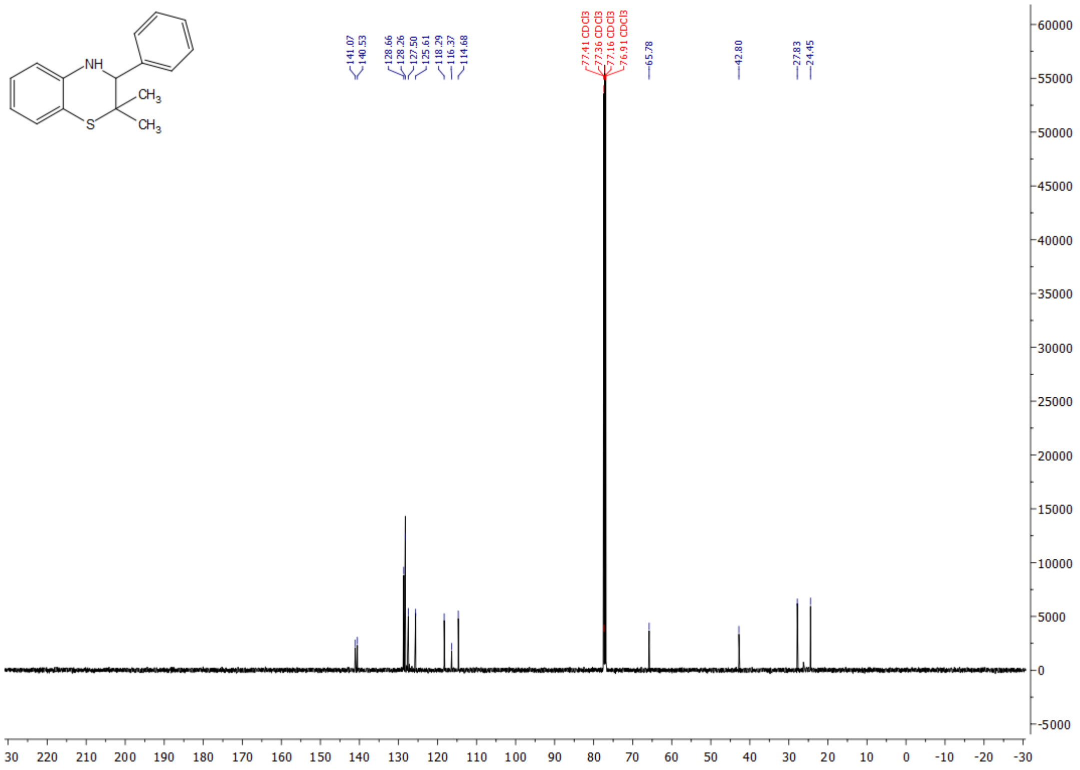

Supplementary Figure 101. ^{13}C **NMR spectra of rac-4c.**

Supplementary Tables

Supplementary Table 1. Results of reduction of 3-thiazoline 1a by Hantzsch ester. The attempts towards reduction using Hantzsch ester are explained in Supplementary Methods.

**Hantzsch ester** / equ.	**DPP** / mol%	**Thiourea cat.** / mol%	**T** / °C	**t** / h	**conversion** / %
1.4	5	-	40	66	0
1.4	10	-	40	86	0
1.4	20	-	40	86	0
1.1	-	10	rt	86	0
1.1	-	20	rt	86	0

Supplementary Table 2. Results of colorimetric pH shift assay. Potential positive IREDs were identified by a colour change from blue/green to yellow. No colour change of the negative control and pET22b_empty is designated as negative. For all substrates there was no colour change of the two negative controls. Exemplary pictures of the colorimetric pH shift assay for 3-thiazoline **1f** are shown in Supplementary Figure 4.

substrate	potentiel positive IREDs	negative control	pET22b_empty
**1a**	IRED5, IRED27	negative	negative
**1b**	IRED5, IRED6, IRED8, IRED13, IRED19, IRED24, IRED27, IRED28, IRED29	negative	negative
**1c**	IRED8, IRED24, IRED27,	negative	negative
**1d**	IRED8, IRED24, IRED27	negative	negative
**1e**	IRED8, IRED24	negative	negative
**1f**	IRED8	negative	negative
**3a**	IRED2, IRED5, IRED6, IRED8, IRED13, IRED14, IRED21, IRED22, IRED24, IRED25, IRED28, IRED30, IRED31	negative	negative
**3b**	IRED1, IRED4, IRED5, IRED8, IRED9, IRED12, IRED13, IRED14, IRED18, IRED21, IRED22, IRED27, IRED28, IRED30	negative	negative
**3c**	IRED5, IRED8, IRED28, IRED29	negative	negative

Supplementary Table 3. Results of spectrophotometric activity assay. Activity was calculated by Supplementary Equation 1 and specific activity by Supplementary Equation 2 explained in Supplementary Methods.

**substrate**	**IRED**	**activity** / U mL^{-1}	**specific activity** / U mg^{-1}
**1a**	IRED5	0.115 ± 0.008	0.007
**1a**	IRED27	0.141 ± 0.004	0.005
**1b**	IRED5	0.136 ± 0.007	0.008
**1b**	IRED24	0.129 ± 0.014	0.006
**1c**	IRED8	0.131 ± 0.008	0.006
**1c**	IRED24	0.095 ± 0.007	0.004
**1c**	IRED27	0.164 ± 0.008	0.006
**1d**	IRED8	0.130 ± 0.004	0.006
**1d**	IRED24	0.086 ± 0.006	0.004
**1d**	IRED27	0.128 ± 0.005	0.005
**1e**	IRED8	0.165 ± 0.004	0.008
**1e**	IRED24	0.114 ± 0.009	0.005
**1f**	IRED8	0.307 ± 0.021	0.015
**3a**	IRED8	2.647 ± 0.348	0.176
**3a**	IRED24	1.941 ± 0.323	0.088
**3b**	IRED4	2.281 ± 0.077	0.114
**3b**	IRED5	2.557 ± 0.128	0.128
**3c**	IRED5	0.572 ± 0.042	0.029
**3c**	IRED8	0.090 ± 0.059	0.006
**3c**	IRED28	0.695 ± 0.103	0.046
**3c**	IRED29	0.756 ± 0.052	0.039

Supplementary Table 4. Overview of imine reductases. Imine reductases that were used, are literature-known enzymes (IRED1-IRED20[1], IRED21-IRED29[2], IRED30-IRED31[3]).

designation of IREDs in this work	designation of IREDs as described in literature	organism
**IRED1**	IR_1[1]	*Streptomyces sp. Mg1*
**IRED2**	IR_2[1]	*Streptomyces fulvissimus*
**IRED3**	IR_3[1]	*Bacillales*
**IRED4**	IR_4[1]	*Kribbella flavida DSM 17836*
**IRED5**	IR_5[1]	*Cupriavidus sp. HPC(L)*
**IRED6**	IR_6[1]	*Saccharomonospora xinjiangensis*
**IRED7**	IR_9[1]	*Frankia sp. QA3*
**IRED8**	IR_10[1]	*Mycobacterium smegmatis*
**IRED9**	IR_11[1]	*Verrucosispora maris*
**IRED10**	IR_12[1]	*Streptomyces sp. CNB091*
**IRED11**	IR_13[1]	*Saccharopolyspora erythraea NRRL 2338*
**IRED12**	IR_14[1]	*Nocardia cyriacigeorgica GUH-2*
**IRED13**	IR_15[1]	*Actinomadura rifamycini*
**IRED14**	IR_17[1]	*Mycobacterium vaccae*
**IRED15**	IR_18[1]	*Mycobacterium sp. JLS*
**IRED16**	IR_19[1]	*Cellulosimicrobium cellulans*
**IRED17**	IR_20[1]	*Streptomyces tsukubaensis*
**IRED18**	IR_21[1]	*Paenibacillus ehimensis*
**IRED19**	IR_22[1]	*Streptomyces sp. CNH287*
**IRED20**	IR_23[1]	*Streptomyces viridochromogenes*
**IRED21**	IR_24[2]	*Bacillus cereus*
**IRED22**	IR_25[2]	*Paenibacillus sp. HGF5*
**IRED23**	IR_27[2]	*Chitinophaga sp. JGI 0001002-D04*
**IRED24**	IR_28[2]	*Glycomyces tenuis*
**IRED25**	IR_29[2]	*Nocardia brasiliensis*
**IRED26**	IR_30[2]	*Nitratireductor pacificus*
**IRED27**	IR_31[2]	*Mesorhizobium sp. L48C026A00*
**IRED28**	IR_32[2]	*Aeromonas veronii*
**IRED29**	IR_33[2]	*Aeromonas veronii*
**IRED30**	IR_7[3]	*Streptomyces aurantiacus*
**IRED31**	IR_8[3]	*Streptomyces sp. Gf 3546*

Supplementary Table 5. Achiral GC analytics for 3-thiazolines/3-thiazolidines. GC analytics is explained in detail in the Supplementary Methods.

**substrate / product**	**temperature programm**	**retention time** / min
**1b / 2b**	40 °C, 10 °C min^{-1} to 200 °C	**1b**: 3.74<br>**2b**: 4.36
**1c / 2c**	40 °C, 15 °C min^{-1} to 200 °C	**1c**: 3.48<br>**2c**: 3.62
**1d / 2d**	40 °C, 10 °C min^{-1} to 200 °C	**1d**: 4.77<br>**2d**: 5.23
**1e/ 2e**	40 °C, 10 °C min^{-1} to 200 °C	**1e**: 8.02<br>**2e**: 8.14
**1f / 2f**	40 °C, 10 °C min^{-1} to 200 °C	**1f**: 8.52<br>**2f**: 8.96

GC chromatograms are shown in Supplementary Figure 56-60.

Supplementary Table 6. Chiral HPLC analytics for derivatized 3-thiazolidines. Methods used for chiral HPLC analytics are explained in detail in Supplementary Methods.

**derivatized 3-thiazolidine**	**retention time** / min[a]
**5c**	(*S*)-**5c**: 13.38<br>(*R*)-**5c**: 14.89
**5d**	(*S*)-**5d**: 10.52<br>(*R*)-**5d**:11.78
**5e**	(*S*)-**5e**: 18.99<br>(*R*)-**5e**: 33.80
**5f**	(*S*)-**5f**:13.52<br>(*R*)-**5f**:17.57

[a]: Absolute configuration was determined by vibrational circular dichroism for (*S*)-**2f** and absolute configuration for other 3-thiazolidines was determined in analogy by means of chiral SFC-HPLC. Chiral HPLC chromatograms are shown in Supplementary Figure 79-82.

Supplementary Table 7. Chiral HPLC analytics for *2H*-1,4-benzothiazines. Methods used for chiral HPLC analytics are explained in detail in Supplementary Methods.

**substrate / product**	**chiral column**	**solvent A:B** [a]	**retention time** / min[b]
**3a / 4a**	Chiralpak IC	95:5	**3a**: 10.95<br>**4a**: 9.28
**3b / 4b**	Chiralpak OB-H	95:5	**3b**: 9.58<br>(*S*)-**4b**: 15.08<br>(*R*)-**4b**: 16.53
**3c / 4c**	Chiralpak OB-H	90:10	**3c**: 16.48<br>(*S*)-**4c**: 19.10<br>(*R*)-**4c**: 21.13

Chiral HPLC chromatograms are shown in Supplementary Figure 93-95.

Supplementary Methods

General experimental information

Reactions that were sensitive to moisture were performed in dried glassware and under argon atmosphere. All commercially available reagents were used as received. Solvents were either used in high-grade purity or purified by distillation.

Column Chromatography was performed by manual column chromatography with silica 60 (0.04-0.063 µm particle size) or by Biotage „Isolera One“ flash chromatography system with cyclohexane/ethyl acetate mixtures.

NMR spectra were recorded on Bruker Avance III 500 or Bruker Advance III 500HD at a frequence of 500 MHz (^{1}H) or 125 MHz (^{13}C). The chemical shift δ is given in ppm and referenced to the corresponding solvent signal ($CDCl_3$). Coupling constants (*J*) are given in Hz.

Nano-ESI mass spectra were recorded using an Esquire 3000 ion trap mass spectrometer (Bruker Daltonik GmbH, Bremen, Germany) equipped with a standard nano-ESI source. Samples were introduced by static nano-ESI using *in-house* pulled glass emitters. Nitrogen served both as the nebulizer gas and the dry gas. Nitrogen was generated by a Bruker nitrogen generator NGM 11. Helium served as cooling gas for the ion trap and collision gas for MS^n experiments.

HRMS-ESI mass spectra are recorded using an Agilent 6220 time-of-flight mass spectrometer (Agilent Technologies, Santa Clara, CA, USA) in extended dynamic range mode equipped with a Dual-ESI source, operating with a spray voltage of 2.5 kV. Nitrogen served both as the nebulizer gas and the dry gas. Nitrogen was generated by a nitrogen generator NGM 11. Samples are introduced with a 1200 HPLC system consisting of an autosampler, degasser, binary pump, column oven and diode array detector (Agilent Technologies, Santa Clara, CA, USA) using a C18 Hypersil Gold column (length: 50 mm, diameter: 2.1 mm, particle size: 1,9 µm) with a short gradient (in 4 min from 0% B to 98% B, back to 0% B in 0.2 min, total run time 7.5 min) at a flow rate of 250 µL/min and column oven temperature of 40°C. HPLC solvent A consists of 94.9% water, 5% acetonitrile and 0.1% formic acid, solvent B of 5% water, 94.9% acetonitrile and 0.1% formic acid. The mass axis was externally calibrated with ESI-L Tuning Mix (Agilent Technologies, Santa Clara, CA, USA) as calibration standard.

EI mass spectra were recorded using an Autospec X magnetic sector mass spectrometer with EBE geometry (Vacuum Generators, Manchester, UK) equipped with a standard EI source. Samples were

introduced by push rod in aluminium crucibles if not otherwise noted. Ions were accelerated by 8 kV in EI mode.

Chemical attempts towards reduction of 3-thiazolines

Reduction with hydrogen and palladium on activated carbon

2,2,4-trimethyl-3-thiazoline (**1c**) (500 mg, 3.87 mmol) is dissolved in methanol (5 mL) and palladium on activated carbon (10%) (41.0 mg, 0.39 mmol, 10 mol%) is added. The reaction mixture is stirred for 18 h under hydrogen atmosphere at room temperature. Palladium on carbon is filtered off, washed with cold methanol und the solvent evaporated *in vacuo*. **1c** was not converted.

Attempts towards reduction using Hantzsch esters

2,2-dimethyl-3-thiazoline (**1a**) (80.0 mg, 0.70 mmol) is dissolved in dichloromethane (5 or 7 mL). Hantzsch ester (247 mg, 0.98 mmol, or 194 mg, 0.77 mmol) and diphenylphosphate (17.4 mg, 0.07 mmol, 10.0 mol%) or schreiner´s thiourea catalyst (17.4 mg, 0.07 mmol, 5.00 mol%) are added and stirred under argon atmosphere for 66 h or 86 h at 40 °C or room temperature. The solvent is evaporated *in vacuo*. The results are summarized in Supplementary Table 1.

Reduction using $LiAlH_4$

Finely crushed $LiAlH_4$ (58.8 mg, 1.55 mmol) is dissolved in diethylether (4 mL) and cooled to 4 °C. 2,2,4-trimethyl-3-thiazoline (**1c**) (200 mg, 1.55 mmol) is dissolved in diethylether (2 mL) and added to the suspension at 4 °C. The reaction mixture is stirred at room temperature for 2 h. Ice water and diethylether are added and the organic phase is decanted carefully. The salts are washed three times with diethylether. The combined organic phases are dried over magnesium sulfate and the solvent is evaporated *in vacuo*. **1c** was not reduced to the desired 3-thiazolidine. In contrast to this ring opening of the *N,S*-acetal and other cleavage products were observed.

Reduction using NaB(Boc-Pro)$_3$H

Boc-DL-Pro-OH (11.0 g, 51.1 mmol) is dissolved in tetrahydrofuran (25 mL) and finely crushed $NaBH_4$ (0.65 g, 17.0 mmo) is added. The reaction mixture is stirred at room temperature for 2 h. The mixture is cooled to 4 °C and 2,2,4-trimethyl-3-thiazoline (**1c**) (1.00 g, 7.74 mmol), dissolved in tetahydrofuran (10 mL) is added. The mixture is stirred at 4 °C for 24 h. Hydrochloric acid (20 mL) is added and the mixture is stirred for 30 minutes at 60 °C. The pH of the mixture is increased by adding potassium carbonate and extracted three times with ethyl acetate. The combined organic phases are washed with brine, dried over magnesium sulfate and the solvent is evaporated *in vacuo*. **1c** was not converted.

Reduction using $NaBH_4$

2,2,4-trimethyl-3-thiazoline (**1c**) (200 mg, 1.55 mmol) is dissolved in methanol (3 mL) and cooled to 4 °C Finely crushed $NaBH_4$ (58.6 mg, 1.55 mmol) is added slowly. The reaction mixture is stirred at room temperature for 2 h. dH_2O is added and the mixture is extracted three times with dichloromethane. The combined organic phases are dried over magnesium sulfate and the solvent is evaporated *in vacuo*. Most of **1c** was not converted. 0.7% desired product could be found.

Reduction with zinc dust

2,2,4-trimethyl-3-thiazoline (**1c**) (250 mg, 1.93 mmol) is dissolved in aqueous potassiumhydroxide solution (5 w%, 9 mL). Zinc dust (1.89 g, 29.0 mmol) is added and the mixture is stirred under argon atmosphere for 24 h at room temperature. Ethylacetate is added, zinc dust is filtered off and washed with ethylacetate. Phases are separated and the aqueous phase is extracted two times with ethylacetate. The combined organic phases are washed two times with dH_2O, dried over magnesium sulfate and the solvent is evaporated *in vacuo*. **1c** was not converted.

Reduction using DIBAL

2,2,4-trimethyl-3-thiazoline (**1c**) (250 mg, 1.93 mmol) is dissolved in toluene (3.4 mL) and cooled to -78 °C. DIBAL (1 M in toluene, 4.4 mL, 2.30 equ.) is added slowly and stirred at room temperature for 5 minutes. Methanol (0.67 mL) is added and then citric acid (cooled to 0 °C, 10%, 9.2 mL) is added. The reaction mixture is stirred for 2 h and is extracted two times with ethylacetate. The combined organic

phases are washed with brine, dried over magnesium sulfate and the solvent is evaporated *in vacuo*. **1c** was not converted.

DNA and Protein Sequences of imine reductases

Codon optimized DNA sequences and protein sequenes of imine reductases (IREDs) containing N- or C-terminal His_6-Tag on pET-22b(+) vector.

IRED1: IRED from *Streptomyces sp. Mg1* (N-terminal His_6-Tag) 975 bp[1]

ATGCACCATCACCATCATCACAATGCCCCGCAAAACCCGACGACGAGCCAAAACTCCGCCGTTA
CCGTTATCGGTCTGGGTCCGATGGGCCAAGCAATGACCCGTGCCCTGCTGGATAGTGGTCATCC
GGTCACCGTGTGGAACCGTACGGCAGGTCGTGCAGCCGGCGTGGTTGCAGACGGTGCTACCCT
GGCACCGACGCCGGCAGGTGCTGTGGAAGCAAGCGATCTGGTTATTCTGTCTCTGACCGACTAT
CGTGCGATGTACGAAGTGCTGGGCGGTGCTACCGGTTCTCTGGCAGGTCGTACGCTGGTTAATC
TGAGCTCTGATACCCCGGACCGTACGCGTGAAGCAGCTCGTTGGGCAGCAGGTCACGGCGCAG
CTTTTCTGACCGGCGGTGTTATGGTCCCGGCACCGATGGTCGGTACCGAAGCAGCCCATGTGTA
TTACAGTGGCGGTGGCGAAGCAGCTCGTTCCCACCTGGCAACCCTGGCACCGCTGGGTACGCC
GCGCTATCTGGGTGAAGATCCGGGCCTGGCGCAGCTGATGTACCAGGCTCAACTGGCGGTGTT
CCTGACCACGCTGTCAGCCCTGATGCATGCAACCGCAATGCTGGGTACGGCAGGTCTGAAAGCC
GGTGAAGCACTGCCGGAACTGCTGAGTTCCGCAGATGCTATTGGCGCCATCCTGCGTGCAGGTG
AAGAACATCCGGGTGCAGCACTGGATGCAGGTGAACACCCGGGTGACCTGTCGACCGTTACGAT
GATGGGTGCGACCGCCGATCACATCGTCGAAACCTCAACGTCGCTGGGTCTGGACCTGGCACTG
CCGCTGGCTGTTCGTGCGCATTATCGTCGCGCCATTGAAGATGGTCACGGTGGCGACAACTGGA
CCCGCATTATCGATGGCATCCGTGGCCCGCGTCGTGCAGACCCGGCGTCCGCAGACCGTGTTAT
CGCAGCCCCGGCAGGCTAA

324 aa

MHHHHHHNAPQNPTTSQNSAVTVIGLGPMGQAMTRALLDSGHPVTVWNRTAGRAAGVVADGATLA
PTPAGAVEASDLVILSLTDYRAMYEVLGGATGSLAGRTLVNLSSDTPDRTREAARWAAGHGAAFLTG
GVMVPAPMVGTEAAHVYYSGGGEAARSHLATLAPLGTPRYLGEDPGLAQLMYQAQLAVFLTTLSAL

MHATAMLGTAGLKAGEALPELLSSADAIGAILRAGEEHPGAALDAGEHPGDLSTVTMMGATADHIVET
STSLGLDLALPLAVRAHYRRAIEDGHGGDNWTRIIDGIRGPRRADPASADRVIAAPAG

IRED2: IRED from *Streptomyces fulvissimus DSM 40593* (N-terminal His_6-Tag) 954 bp[1]

ATGCACCATCACCATCATCACAGTAGTGCTCGTCAACAACAACAGTCCGTCACCGTCATTGGTCT
GGGTCCGATGGGTCGCGCAATGGTCGCCGCTCTGCTGGATCGCCAGTATGCAGTTACCGTCTG
GAACCGTACGCCGTCACGTGCAGGTGATCTGGTGGCACGTGGTGCTGTTCTGGCACCGAGTCC
GGCTGAAGCAGTGGCAGCCAATGAAGCGGTGGTTATTTCCCTGACCGATTATGCAGCTGTCTAC
GACGTGCTGGAAGCAGCAGCACCGGCTCTGCAGGATCGTGCACTGCTGAACCTGACCAGCGCA
ACGCCGGAAGAAGCCCGTGCAGGTGCTCGTTGGGCTGCAGGTCGTGGTGCAGTCCAACTGACC
GGCGGTGTGAATTCACCGCCGTCGGGCATTGGTAAACCGGATAGCTCTACGTTTTATTCTGGCC
CGCGTGAAGTGTTCGACCGTCATCGTCCGCTGCTGGAAGCACTGACCGGTCGTGCAGATCATCG
TGGTGAAGACCCGGGTCACGCAGCACTGCTGTATCAGATCGGCGTTGGCATGTTTTGGACCAGC
ATGCTGTCTTACTGGCAAGCGATTGCACTGGCACGTGCTAACGGTCTGACGGCTGCGGATATCC
TGCCGCACGCTGATGACACCGCGAATTCACTGACGCAATTTTTCGCGTTCTACACCGATCGTATC
GACGCCGTTGAACATACGGGCGATGTCGACCGCCTGGCCATGGGTATGGCATCGGTTGAACAC
GTCCTGCGCACCAACGCGGATGCCGGTGTTGACACGGCACTGCCGGCAGCAGTCGTGGACCTG
TTTCGTCGCGGCATGGAAGCCGGTCATGCAACCGACAGTTTCTCCGCGCTGGTTGAAGTGATGG
GCAAACCGACCGATCCGGGCACCGATGGCCGTGTTGGTCAAGCGGGTCCGTTCCTGCGTTAA

317 aa

MHHHHHHSSARQQQQSVTVIGLGPMGRAMVAALLDRQYAVTVWNRTPSRAGDLVARGAVLAPSPA
EAVAANEAVVISLTDYAAVYDVLEAAAPALQDRALLNLTSATPEEARAGARWAAGRGAVQLTGGVNS
PPSGIGKPDSSTFYSGPREVFDRHRPLLEALTGRADHRGEDPGHAALLYQIGVGMFWTSMLSYWQAI
ALARANGLTAADILPHADDTANSLTQFFAFYTDRIDAVEHTGDVDRLAMGMASVEHVLRTNADAGVDT
ALPAAVVDLFRRGMEAGHATDSFSALVEVMGKPTDPGTDGRVGQAGPFLR

IRED3: IRED from *Bacillales* (N-terminal His_6-Tag) 945 bp[1]

ATGCACCATCACCATCATCACAAATCTGACCACATCGAAAACATCAACAAATCGGCAAGCCACGG
CACGGAAAAAGTCGGCAGTCGTCTGTCGGTTACGGTTATCGGCCTGGGCCCGATGGGTAAAGCT

ATTGTGGGCGCGTTTCTGGATAAAGGTTATGAAGTCACCGTGTGGAACCGTACGCTGTCGAAAG

CCGATGACCTGATGGCAAAAGGCGCTATGAAAGCGTCAACCGTCTCGGAAGCAATTACGTCAAAT

GATCTGATCGTGCTGTCGCTGACCGACTATCGCGCCATGTACGCAATTTTCGAACCGATCTCCGA

ACAGCTGACCGGTAAAGTTATCGTCAACCTGAGCTCTGATACGCCGGAAAAAGTTCGTGAAGCCT

CCGAATGGCTGGCCGAACGCAACGCAGTTCAACTGACCGGCGGTGTCCTGGCATCACCGCCGG

GCATTGGTAATAAAGAAAGTGTTACCCTGTATTCCGGCCCGCGTAAAACGTTTGATGACCATCAG

AATATCCTGGAAGTCCTGACCAGTACGTCCTACAAAGGCGAAGATCCGGGTCTGGCTATGCTGTA

TTACCAGCTGCAAATTGACGTGTTCTGGACCGCGATGCTGAGCAACCTGCACGCAGTGGCTGTT

GCGCGTGCCAATGGTATTACCGCTGAACAGCTGCTGGCGTATGTTAGCGATATCCTGTCTACGAT

GCCGAAACTGCTGGAATTTTACGCCCCGCGCATTGATGCAGGCACCCATAGCGGTGACGTGGAA

AAACTGGCTATGGGCCTGGCGAGCGTTGAACACGTGGTTCAAACGTCTAACGAAGCCGGTATCG

ATGCAAGTCTGCCGGCAGCAGTCCTGGACGTGTTCAAACGCGGCGTTGCTCGTGGTCATGCGG

GTGATTCTTTTACCTCGCTGATTGATATGTTCCAGAAACACTAA

314 aa

MHHHHHHKSDHIENINKSASHGTEKVGSRLSVTVIGLGPMGKAIVGAFLDKGYEVTVWNRTLSKADDL

MAKGAMKASTVSEAITSNDLIVLSLTDYRAMYAIFEPISEQLTGKVIVNLSSDTPEKVREASEWLAERNA

VQLTGGVLASPPGIGNKESVTLYSGPRKTFDDHQNILEVLTSTSYKGEDPGLAMLYYQLQIDVFWTAM

LSNLHAVAVARANGITAEQLLAYVSDILSTMPKLLEFYAPRIDAGTHSGDVEKLAMGLASVEHVVQTSN

EAGIDASLPAAVLDVFKRGVARGHAGDSFTSLIDMFQKH

IRED4: IRED from *Kribella flavida DSM 17836* (N-terminal His_6-Tag) 909 bp[1]

ATGCACCATCACCATCATCACCCGCCGACGGATCGTACGCCGGTCACGCTGATTGGCCTGGGTC

CGATGGGTCAAGCTATGACGCGCGCCCTGCTGGCTGCGGGTCATCCGGTTACCGTCTGGAACC

GTACGCCGGCACGTGCAGCCGGCGTGGTTGCAGATGGTGCAGTTCTGGCAGCTAGCCCGGTGG

AAGCTGTTGAAGCGGGCGATCTGGTTATTCTGTCTCTGACCGACTATCAGGCCATGTACGATGTC

CTGGAACCGGCAACCGGCAGCCTGGCAGGTCGTACGGTCGTGAATCTGAGCTCTGACACCCCG

GATCGTACGCGTGCAGCAGCAGATTGGGCTACCGAACATGGCGCGACCTTTCTGACGGGCGGT

GTCATGATTCCGGCTCCGATGGTGGGCACCGAAGAAGCGTATGTGTATTACTCCGGTCCGGCGG

AAGTCTTCGAAAAACACCGTACCACGCTGACCGTGATCGGTGCACCGCGTTATCTGGGTGAAGA

TACGGGTCTGGCCCAACTGATGTACCAGGCACAACTGGACGTGTTTCTGACCACGCTGAGTTCC

CTGATGCATGCAACCGCACTGCTGGGTACGGCCGGTGTGTCAGCTGCAGAATCGATGCCGGAAC
TGATTGGCATGCTGCGTACCGTTCCGGCTATGCTGGAAGCGGGCGGTGAAAACCCGGGTGCCG
ATATTGACGCAGATAAACATCCGGGCGACCTGAGTACCATCACGATGATGGGTGCTACCGCGGA
TCACATTGTCGGCGCTTCAGAAACGGCGGGTATCGACCTGGCACTGCCGCGTGCAGTGCAGGC
ACACTACCGTCGCGCAATCGAAAACGGCCACGGTGGTGACAACTGGACCCGCATTATTGACGGT
ATTCGCTCCCCGCGTTAA

302 aa
MHHHHHHPPTDRTPVTLIGLGPMGQAMTRALLAAGHPVTVWNRTPARAAGVVADGAVLAASPVEAV
EAGDLVILSLTDYQAMYDVLEPATGSLAGRTVVNLSSDTPDRTRAAADWATEHGATFLTGGVMIPAP
MVGTEEAYVYYSGPAEVFEKHRTTLTVIGAPRYLGEDTGLAQLMYQAQLDVFLTTLSSLMHATALLGT
AGVSAAESMPELIGMLRTVPAMLEAGGENPGADIDADKHPGDLSTITMMGATADHIVGASETAGIDLA
LPRAVQAHYRRAIENGHGGDNWTRIIDGIRSPR

IRED5: IRED from *Cupriavidus sp. HPC(L)* (N-terminal His_6-Tag) 909 bp[1]
ATGCACCATCACCATCATCACAAAACCGTCGCAGTCATCGGCCTGGGTCAAATGGGCACCACGC
TGGCTCGTCTGTTCATCGAAGCGGGTATGCAAGTCCGTGTCTGGAACCGTACCCGCTCAAAAGC
TGAACCGCTGGCATCCCGTGGTGCAATTGTCGCAGCAACGGCAGCTGCAGCAATGGCAGATGCT
GAAGCGGTGGTTATTTGCGTTCATGACTATCGCGCGACCCACGATATCCTGTCAGACGTTGCAGT
CAAATCGGCTCTGAAAGGTAAACTGCTGCTGCAGCTGACCACGGGCAGCCCGCAAGATGCACGT
GACATGGCAGAACTGGCAGCTCGTATCGGTGCAGGTTATCTGGATGGTGCACTGCAGGTGGCTC
CGGAACAGATGGGCCAACCGGATACCACGGTGCTGGTTAGCGGCTCTGGTGAAGACCATGCCC
TGGCACGTGAACTGCTGGCAGTGCTGGGCGGTAACGTCGTGTACCTGGGTGAAGATGTTGCAGC
AGCAGCTACCATGGACCTGGCAACGCTGAGCTATGTGTACGGCGCCTCTATGGGCTTTTTCCAG
GGTGCAGCACTGGCTCAAGCAGAAGGTCTGGATGTCGGCGTGTATGGCGGTATTGTTGAAGCAA
TGAGTCCGTCCTTTGGCGCGTTCCTGCGTCACGAGGGTAACGTTATCGATAATGGCGACTACGC
GGTCTCACAGTCGCCGCTGAGCATTTCTATCGATGCCACCGGTCGCATTGAACAGGCAATGCGT
CAAAAAGGCCTGCGCAGTGAACTGCCGTCCCTGATCGCACGTCTGCTGCGTGATGCAGAAGAAG
CAGGCTACGGTAATGAAGAATTTGCTGCTGTGGCGAAAATCCTGCGTGGTGCTGCGGAACCGGC
CCCGGTGCGTTAA

302 aa

MHHHHHHKTVAVIGLGQMGTTLARLFIEAGMQVRVWNRTRSKAEPLASRGAIVAATAAAAMADAEAV

VICVHDYRATHDILSDVAVKSALKGKLLLQLTTGSPQDARDMAELAARIGAGYLDGALQVAPEQMGQP

DTTVLVSGSGEDHALARELLAVLGGNVVYLGEDVAAAATMDLATLSYVYGASMGFFQGAALAQAEGL

DVGVYGGIVEAMSPSFGAFLRHEGNVIDNGDYAVSQSPLSISIDATGRIEQAMRQKGLRSELPSLIARL

LRDAEEAGYGNEEFAAVAKILRGAAEPAPVR

IRED6: IRED from *Saccharomonospora xinjiangensis XJ-54* (N-terminal His_6-Tag) 918 bp[1]

ATGCACCATCACCATCATCACACCACGACGGCTACGGGTACGACGGGTTCCCTGGCGGCTGATC

CGGTCACGGTTCTGGGTCTGGGCGACATGGGCTCGGCAATCGCTCGTGCTTTTGTTGAACGTGG

CCATCGCACCACGGTCTGGAACCGTACCGCCTCAAAATGCCGTCCGCTGGTTGAAGCTGGTGCA

TCGGCAGCAGCAACGCCGGATGAAGCTGTGGAAGCGAGTCGCTTCGTGGTTGTCTGTCTGCTGG

ATAGCGCTGCGGTGGACGAAGTTCTGGGCTCTGTTACCAGCTCTCTGGCCGGTAAAGTCCTGGT

GAACCTGACGAGTGGCTCCCCGTCACAGGCACGTAGCAATGAACGCTGGGCCCGTGAACGCGG

TGCAGAATATCTGGATGGCAAAATTATGGGTGATCCGCCGGACGTGGGCACCAGCAATGTTTCG

CTGAGCTTTTCTGGTAGTCGTTCCGCCTTCGATGCACATGAACCGATCCTGCGCGAACTGGGCG

GTGTGGCTTATCACGGTGAAGACGCAGGTCTGGCAGCAGTCGAATTTCTGGCTCAAGTGGCGAT

GGGCTACGAACTGCTGATTGGTTTCCTGCATACCCTGAGCGTGGTTCACGCCGAAGGCGTTGAA

GTCGAAGCCTTTGCAGAACGTGTTGCAGGTTCTGTCGCTGCGTATCCGCCGCTGCTGACGATGA

TGGGCAAAGCCATTGGCAGTGGTGAATACGGCCCGGATCTGGGTTCCCTGCGTGTTCAGGCCG

CACTGATGGATGACCTGATCTCACACCGCGAATCGCTGGGTGTCGAAGCGGTGCGTATGCGCGA

AGTGAAAGAACTGATGGACCAACGCATTGCGGACGGTCACGGCGGCCAAGGCTTCTCATCGCTG

TTTGAACTGCTGACGAAACGCTAA

305 aa

MHHHHHHTTTATGTTGSLAADPVTVLGLGDMGSAIARAFVERGHRTTVWNRTASKCRPLVEAGASA

AATPDEAVEASRFVVVCLLDSAAVDEVLGSVTSSLAGKVLVNLTSGSPSQARSNERWARERGAEYLD

GKIMGDPPDVGTSNVSLSFSGSRSAFDAHEPILRELGGVAYHGEDAGLAAVEFLAQVAMGYELLIGFL

HTLSVVHAEGVEVEAFAERVAGSVAAYPPLLTMMGKAIGSGEYGPDLGSLRVQAALMDDLISHRESL

GVEAVRMREVKELMDQRIADGHGGQGFSSLFELLTKR

IRED7: IRED from *Frankia sp. QA3* (N-terminal His_6-Tag) 999 bp[1]

ATGCACCATCACCATCATCACACCGACCCGGCACCGCGTAATGACCGCCCGCGTGACGTTGACG

GCCCGCACCACGTTGTTGACGACCGCCCGCCGAATGGCTCCCGCGGTCGTGCACGTCAGCCGG

ATCGTGGTGAACCGGCACCGCTGGCTGTGCTGGGTCTGGGTGCAATGGGTACCGCACTGGCTC

GTACGTGGCTGGCAGCAGGTCATCCGACCACGGTTTGGAACCGTACCCGTGCACGTGCAGAAC

CGCTGGTCGCGGAAGGTGCAACCCTGGCAGACACGGCAGCTGAAGCAGTTGCAGCAACCCCGC

TGATTGTGGTTTGCCTGCTGGATGACGCAAGCGTGGGCGCAGCTCTGGCAGATGCTGAACTGGC

CGGTCGCGACCTGGTTAATCTGACCACGGGCTCCCCGGCACAAGCCCGTGCACGTGCGGCCTG

GGCGCACGAACGCGGTGCCCGTTATCTGGATGGCGGTATCATGGCAGTCCCGCCGATGGTGGG

CAGCTCTCCGACCCGCGGTTATGTCTTTTACAGTGGCTCCCGTGCACTGTTCGATGACCGTGGTC

GTACGCTGGCAGTTCCGCTGGACCCGCGTTACGTCGGTGCCGACCCGGGTCTGGCAGCTCTGC

ATGATGTTGCCCTGCTGAGTGCAATGACCGGCATGTTTGCGGGTATTTCACACGCTTTCGCACTG

ATCCGTGCAGCCGGTGTCCCGGCTCGTCCGTTTGCACCGCTGCTGGTTGAATGGCTGCGTGCCA

TGGCAACCTCAGCTTCGGCGACGGCCGAACATCTGGATAGCGGCGACTATACCACGGGTGTGAT

GTCTAACCTGGCGATGCAGGTTGCTGGTAATGCGACCCTGCTGCGTACGGCAGCTGAACTGGGC

GTGGATGCCGAACTGCTGACCCCGTACATGGCGGCCATGCAACGTCGCCTGGCTGATGGTCAC

GCCGACGAAGGTGTCACGGGTGTTATCGACCAACTGCTGTCATAA

332 aa

MHHHHHHTDPAPRNDRPRDVDGPHHVVDDRPPNGSRGRARQPDRGEPAPLAVLGLGAMGTALAR

TWLAAGHPTTVWNRTRARAEPLVAEGATLADTAAEAVAATPLIVVCLLDDASVGAALADAELAGRDLV

NLTTGSPAQARARAAWAHERGARYLDGGIMAVPPMVGSSPTRGYVFYSGSRALFDDRGRTLAVPLD

PRYVGADPGLAALHDVALLSAMTGMFAGISHAFALIRAAGVPARPFAPLLVEWLRAMATSASATAEHL

DSGDYTTGVMSNLAMQVAGNATLLRTAAELGVDAELLTPYMAAMQRRLADGHADEGVTGVIDQLLS

IRED8: IRED from *Mycobacterium smegmatis ATCC 700084* (N-terminal His_6-Tag) 897 bp[1]

ATGCACCATCACCATCATCACACGACGACGCCGACGGTTACGGTCCTGGGTCTGGGTCCGATGG

GTCAAGCCCTGTCTCGCGCCCTGCTGGATGCGGGTCACACGGTTACCGTGTGGAACCGTACGG

AAAGCAAAGCACAGGCTCTGCGTGATCGTGGTGCACTGAGTGCTCCGACCCCGGCAGCAGCAAT

TGCTGCATCCGATCTGGCGCTGGTTAATGTGGTTGATCATGACGCGGTCGATGCCATTCTGACC

GCAGCAGGTGACGCACCGGCAGGTCGTACGGTTATCGGTCTGAGCTCTGATACCCCGGACCGT

GCACGTCGCACGGCTAAACTGGTCGGTAACGTGGGCGGTCGTTATCTGGATGGCGCCATTATGA
CCCCGATTGACACCATCGGCACGCGCGGTGCATCAATCCTGTTTGCGGGCCCGCAGGCCCTGTT
CGATGAACATCGTGGTGTCCTGGACACCCTGGGCCAACTGACGTGGGTGGGTGAAGATCACGGT
CGTGCTGCAGCCTTTGATATGGCGCTGCTGGACCTGTTTTGGACCAGCGTGGGCGGCTTTGGTC
ACGCACTGATGGTTGCACGTGCTAATGGCATTGAACCGTCAGAACTGATGCCGCATGCGCACGG
CATTGTGGGTATCCTGTCGCCGATCTTTACGGAAGTGGCCCAACGTGTTGAAGATGACCGCCATA
GCGATGCGAGCGCCTCTGTTAGTTCCGTCGCGTCATCGGTTCGTCACCTGATCGCAGCTTCTCG
CGAAGCAGGCGTCGATGCTGGTCTGCTGGAAGCATTCCGCGGTTACGTCGACGCGACCGTGGC
GGCCGGCCATGGTGACGACGAAATCTCCCGTATTGCCAGCGAAATGACGACCCTGACCCGTGGT
TAA

298 aa
MHHHHHHTTTPTVTVLGLGPMGQALSRALLDAGHTVTVWNRTESKAQALRDRGALSAPTPAAAIAAS
DLALVNVVDHDAVDAILTAAGDAPAGRTVIGLSSDTPDRARRTAKLVGNVGGRYLDGAIMTPIDTIGTR
GASILFAGPQALFDEHRGVLDTLGQLTWVGEDHGRAAAFDMALLDLFWTSVGGFGHALMVARANGIE
PSELMPHAHGIVGILSPIFTEVAQRVEDDRHSDASASVSSVASSVRHLIAASREAGVDAGLLEAFRGYV
DATVAAGHGDDEISRIASEMTTLTRG

IRED9: IRED from *Verrucosispora maris AB-18-032* (DSM 45365) (N-terminal His_6-Tag) 909 bp[1]
ATGCACCATCACCATCATCACGCGGCGGATTCGCGTGCTCCGGTCACCGTTATCGGCCTGGGCG
CAATGGGTTCGGCTCTGGCTCGTGCTTTCCTGGCAGCGGGTCACCCGACCACGGTTTGGAACCG
TTCACCGGATAAAGCTGATGACCTGGTTGGTCAGGGTGCAGTCCGTGCAGCAACCGTGGCAGAT
GCAATGTCGGCAGGCAATCTGATTGTCATCTGCGTGCTGGACTATCGTGCCATGCGCGAAATTAT
CGATAGCACCGGTCATTCTCCGGCGGACCGTGTGATTGTTAACCTGACCAGTGGTACGCCGGGT
GATGCTCGTGCAACCGCAGCTTGGGCACAGGAACAAGGCATGGAATACATTGACGGTGCCATCA
TGGCAACCCCGAGTATGATTGGCTCCGAAGAAACGCTGATCTTTTATGGCGGTCCGCAAGAAGT
GTACGATGCCCATGCAGACACCCTGCGTAGCATTGCTGGCGCGGGCACGTATCTGGGCGAAGA
ACCGGGTCTGCCGTCTCTGTACGATGTCGCACTGCTGGGTCTGATGTGGACCACGTGGGCAGGT
TTCATGCACTCAGCGGCCCTGCTGGCCTCGGAAAAAGTGCCGGCAGCTGCGTTTCTGCCGTATG
CCCAGGCATGGTTCGAATACGTTATCTCTCCGGAAGTCCCGAACCTGGCTACCCAAGTTGATACG
GGCGCGTATCCGGATAATGACAGTACCCTGGGTATGCAGACGGTGGCCATTGAACATCTGGTTG

AAGCATCCCGTACCCAAGGTGTCGATCCGACGCTGCCGGAATTTCTGCACGCTCGCGCGGAACA
GGCGATCCGTCGCGGTCATGCGGGTGATGGCTTTGGTGCGGTGTTTGAAGTGCTGCGTGCTCC
GGCTGCCCAGTAA

302 aa
MHHHHHHAADSRAPVTVIGLGAMGSALARAFLAAGHPTTVWNRSPDKADDLVGQGAVRAATVADA
MSAGNLIVICVLDYRAMREIIDSTGHSPADRVIVNLTSGTPGDARATAAWAQEQGMEYIDGAIMATPS
MIGSEETLIFYGGPQEVYDAHADTLRSIAGAGTYLGEEPGLPSLYDVALLGLMWTTWAGFMHSAALLA
SEKVPAAAFLPYAQAWFEYVISPEVPNLATQVDTGAYPDNDSTLGMQTVAIEHLVEASRTQGVDPTLP
EFLHARAEQAIRRGHAGDGFGAVFEVLRAPAAQ

IRED10: IRED from *Streptomyces sp. CNB091* (N-terminal His_6-Tag) 1.014 bp[1]
ATGCACCATCACCATCATCACGCCCCGCACAATCAACACCAACACCAAACGCAACACCAAACCCC
GGTCACGGTTATCGGCCTGGGCGCAATGGGCTCGGCACTGGCTGCGGCCTTTATTGCAGCTGG
CCATCCGACCACGGTGTGGAACCGTACCGCATCACGTGCAGCACCGCTGGTTGCCCGTGGTGC
AGCTCACCCGGAAACGGTTGCTGAAGCAGTCGCAGCATCGCCGCTGGTTATCACCTGCCTGACC
ACGTATGAAGATACGGTCGAAGCACTGGAACCGGCAGCTGCAGCACTGAAAGGTCGTGACCTGG
TTACCCTGAACAGTGGCTCCCCGGCAGGTGCACGTCGCGCAGCTGAATGGGCTCGTGGTCATG
GTGCACGTTACCTGGGCGGTGCAATTAAAAATGTGCCGCCGGCTGTTGGCGCGGAAGATACCCT
GCTGTATTACAGTGGTGATGCCACCGTTTTTACGGACCACGAACCGGTGCTGCGTGTTCTGGGC
GGTGATACCGTGTATCTGGGTGCCGATCCGGACCTGGCAGCACTGTACGAAATGGCAGTCGGC
GGTACCCTGCTGCCGGCCCTGGTGGGCTTTTTCCAGGGTGCAGCTGCACTGCGTGCACGCGGT
CTGGAAGCCGCAAGCATGGTGCGTTTCTCTGAACAGTGGCTGCAAATGATTGCCTCTGTCCTGC
CGGTGCTGGCACGCGAAATCGATTCAGGCGACTATTCGGAACCGCTGAGCTCTGTGAATGTTTTT
GTCGCCGGTGCTGCGCATGATGCAGAACTGGGCAAAGAAGCTGGTCTGGACGTCGAATGGCATA
AACCGTTCCACGAACTGCTGGAACGCGCCGTGAAAGCAGGCTACGGTACCCAAAGTATTGCCGC
ACTGACGGAAATCCTGATGGAACCGCGTCGCGATGCGCTGACCCCGCCGACGTCCCCGAGTGG
CACCAGCCCGCGTCGTGCGTCATCACCGGCCCGCCCGCGTTCAGGCACCCCGTCGTAA

331 aa

MHHHHHHAPHNQHQHQTQHQTPVTVIGLGAMGSALAAAFIAAGHPTTVWNRTASRAAPLVARGAAH

PETVAEAVAASPLVITCLTTYEDTVEALEPAAAALKGRDLVTLNSGSPAGARRAAEWARGHGARYLG

GAIKNVPPAVGAEDTLLYYSGDATVFTDHEPVLRVLGGDTVYLGADPDLAALYEMAVGGTLLPALVGF

FQGAAALRARGLEAASMVRFSEQWLQMIASVLPVLAREIDSGDYSEPLSSVNVFVAGAAHDAELGKE

AGLDVEWHKPFHELLERAVKAGYGTQSIAALTEILMEPRRDALTPPTSPSGTSPRRASSPARPRSGTP

S

IRED11: IRED from *Saccharopolyspora erythraea NRRL 2338* (N-terminal His_6-Tag) 921 bp[1]

ATGCACCATCACCATCATCACCACAACGGTTTCGCTGCCCCGGTTACGGTCGTGGGCCTGGGTC

CGATGGGTTACGCACTGGCTGAAGCATTCCTGGCGGCGGGTCACCCGACCACGGTGTGGAACC

GTAGTGCTCATAAAGCCGATCCGCTGGTTGCGGAAGGTGCAGTGCGTGCAGCAACCGCAGCTGA

AGCACTGGCGGCCTCAGATCTGGCAGTGGTTTGCGTTGCTGACTATGCAGCTATGCATGCAGCA

CTGGACCACTGCGGTACCGCACTGAGCGGTAAAGTTCTGGTCAACCTGTGTTCGGGTACGCCGC

AGGAAGCTCGTGAAGCACTGACCTGGGCAACGGCACATGGTGCAGGTTATCTGGATGGCGCCAT

TATGGTGCCGGTTGAAGTCATCGGTACCCCGAGCTCTGTCGTGTTTTACAGCGGCGCACGTGAA

CCGTTCGATGCACACCGTAATACGCTGGACGCACTGGGCGGTGTCCCGCGTTACCTGGGCGGT

GATGCCGGTCTGGCAGTGCTGCATAACACCGCACTGCTGGGTCTGATGTGGGCAACGGTGAATG

GTTTTCTGCACGCAGCTGCGCTGGTTGAAAGCGGCGGTGTGGGCGTTGCTGATTTTGCGGAAAC

CGCCGTTGACTGGTTCCTGCCGTCTGTCACGGGTGAAATTCTGCGTGCGGAAGCCGCACGTATC

GATCGCGAAGAATTCCCGGGTGACGGCGGTACCCTGGCAATGTGTCTGACGGCCATTGAACACA

TCGTTCGTACCAGCCGCGATGCCGGTATTAGTGACGAAGTGCCGTCCCAACTGAAAGCACTGGG

TGATCGTGCTGTTGCTGCAGGTTATGGTGATGAAAACTACATGAGCCTGATTAAAGTGCTGCGTG

TTCCGTCCGCTGCTACCCACCGTTAA

306 aa

MHHHHHHHHNGFAAPVTVVGLGPMGYALAEAFLAAGHPTTVWNRSAHKADPLVAEGAVRAATAAEAL

AASDLAVVCVADYAAMHAALDHCGTALSGKVLVNLCSGTPQEAREALTWATAHGAGYLDGAIMVPVE

VIGTPSSVVFYSGAREPFDAHRNTLDALGGVPRYLGGDAGLAVLHNTALLGLMWATVNGFLHAAALV

ESGGVGVADFAETAVDWFLPSVTGEILRAEAARIDREEFPGDGGTLAMCLTAIEHIVRTSRDAGISDEV
PSQLKALGDRAVAAGYGDENYMSLIKVLRVPSAATHR

IRED12: IRED from *Nocardia cyriacigeorgica GUH-2* (N-terminal His_6-Tag) 888 bp[1]

ATGCACCATCACCATCATCACACGAACAACGCAACGCCGGTCTCAATCCTGGGCCTGGGTCTGA
TGGGTCAAGCTCTGGCACGCGCCTTCCTGAAAGCCGGTCATCCGACCACGGTCTGGAACCGTAC
CCCGGGCAAAGCGGATCAGCTGATGGCGGAAGGTGCCCAAGTTGCACCGACCGCGGCCGAAGC
TATTGATGCGAGCTCTCTGACGGTGATCTGCGTTAGTGACTATCCGGCGATGTACGAACTGCTGG
ATGCTTCCGACCTGGCAGGTACCACGCTGCTGAATCTGACCAGTGGTGATTCCGCACAGGCTCG
TCAAGCAGCTCGTTGGGCAGAACAGCGTGGTGCACATTATCTGGACGGTGCCATTATGGCAATC
CCGCAAGCAATCGGCACCGATGACGCGGTGATTCTGATCAGCGGTGCACAGGCAGATGCAGAC
GCTCATCGTCCGACGCTGGAAGCACTGGGTACCCTGACGTATCTGGGCGCAGATCACGGTCTGG
CTAGCCTGTACGACGTTGCTGGTCTGGCGATGATGTGGTCTGTCCTGAACGCATGGCTGCAGGG
TACCGCACTGCTGCGTACGGCCGGTGTGGATGCAGCAACCTTTGCACCGTTCGCACAGCAAATG
GCAGCTGGCGTTGCAGGTTGGCTGCCGGGCCACGCACAGGAAATTGATGCCGGTAGCTTTGCA
ACCGAAGTCGCTTCTCTGGATACCCATGTGCGCACGATGGACCACCTGATTGAAGAATGTGAAG
CGGCCGGCATCAATGCGGAACTGCCGCGTCTGATTAAATCAATGGCCGATCGCTCGCTGGCAGC
AGGTCATGGTGCGGCGTCATACAGCGTTCTGATTGAAGAATTTGCGAAACCGGCTTAA

295 aa

MHHHHHHTNNATPVSILGLGLMGQALARAFLKAGHPTTVWNRTPGKADQLMAEGAQVAPTAAEAID
ASSLTVICVSDYPAMYELLDASDLAGTTLLNLTSGDSAQARQAARWAEQRGAHYLDGAIMAIPQAIGT
DDAVILISGAQADADAHRPTLEALGTLTYLGADHGLASLYDVAGLAMMWSVLNAWLQGTALLRTAGV
DAATFAPFAQQMAAGVAGWLPGHAQEIDAGSFATEVASLDTHVRTMDHLIEECEAAGINAELPRLIKS
MADRSLAAGHGAASYSVLIEEFAKPA

IRED13: IRED from *Actinomadura rifamycini* (N-terminal His_6-Tag) 897 bp[1]

ATGCACCATCACCATCATCACATGAAAGCGCCGGTTACGGTTGTGGGCCTGGGTCCGATGGGCA
AAGCAATGGCTGAAACGTTCCTGAAAAACGGTCACCCGACGACGGTGTGGAACCGTACCGCATC
AAAAGCAGCACCGCTGGTTGAACAGGGTGCAACGCTGGCAGCTACCCCGGATGACGCACTGGC

AGCATCAGGCCTGGTGGTTATTTCGCAAACCGATTATAAAGCAATGTACGATTCACTGGACGGTG
CTGAAATGAAAGGCCGCGTCCTGGTGAATCTGAGCTCTGGTTCGCCGGACGAACTGCGTCGCGC
AGCTGAATGGGCAGCCGGTAAAGGTGCCGAACTGCTGACGGGCGGTGTTATGGTGCCGCCGCC
GGGTATTGGTCAGCCGGGTGCATATATCATGTACAGCGGCCCGGAAGCTCTGCTGGATCGTCAT
CGCGAAACCCTGCGTGTCCTGGGTGATACCACGTATGTGGGTGCCGACGTTGGCCTGTCTAACC
TGTATTACCAGGCACAACTGTACCTGTTTTGGAGTACCCTGACGGCGTACCTGCACTCCATTGCC
ATGCTGCAGAGTGCAGGCGTTTCCGCTGAACAATTTCGTCCGTTCGCGACCGAAACGGTCACCA
GCCTGGGCGTGGATGGTCCGATGGGCTTCCTGCGCATCCTGGCCGAAGAAGCAGACGCTGGTC
ATAGCCCGGGCGGTGAAAATTCTATGCTGATGATGGCGGTTGGCGCCGATCACATGGTCGAAGC
AGCTGAAGCGGCCGGTATCGATACGATGGGTCCGCGTGCACTGCGTGACCTGTTTTGGCGCACC
GTGAATGCCGGTCATGGTGCCGATGGTCTGGGTTCTGTGATTGAAGTCGTTCGCAAAGGTGCCT
AA

298 aa
MHHHHHHMKAPVTVVGLGPMGKAMAETFLKNGHPTTVWNRTASKAAPLVEQGATLAATPDDALAAS
GLVVISQTDYKAMYDSLDGAEMKGRVLVNLSSGSPDELRRAAEWAAGKGAELLTGGVMVPPPGIGQ
PGAYIMYSGPEALLDRHRETLRVLGDTTYVGADVGLSNLYYQAQLYLFWSTLTAYLHSIAMLQSAGVS
AEQFRPFATETVTSLGVDGPMGFLRILAEEADAGHSPGGENSMLMMAVGADHMVEAAEAAGIDTMG
PRALRDLFWRTVNAGHGADGLGSVIEVVRKGA

IRED14: IRED from *Mycobacterium vaccae* (N-terminal His_6-Tag) 876 bp[1]
ATGCATCACCATCACCATCACACCACGGTCGCGGTGATTGGCCTGGGTCCGATGGGTCGTGCAC
TGGCCGCAGCACTGCTGTCAGCAGGCTATCGTGTTACCGTCTGGAACCGCACGGAAAGTAAAGC
TGCGCAGCTGCTGTCCTGTGGTGCACATTGGGCTCCGACCCCGGGTAAAGCGGTTGCAGCAGG
TGATCTGACGCTGATTAATGTGGTTGATCATGACGCGGTGGATGCCGTCGTTCATGCTGCCGCG
GATGCCGTTGCGGGCCGTCTGCTGGTTGGCCTGAGCTCTGATACCCCGGACCGTGCACGTAGC
ACCGCTGAACTGGTTGTCGCAGCTGGCGGTCGCTATCTGGATGGCGCGATCATGACCCCGACG
GACGTGGTTGGTACCGCAGAAGCTTCTGTTCTGTATGCCGGCCCGTATGACCTGTTTGGCGGTC
ATCGTGAACTGTTCGAAACCCTGGGTCAAGCCACCTGGCTGGGTGAAGACCCGGGTCGTGCCG
CAGCATACGATATGGCACTGCTGGACGTGTTTTGGACCGCTGCGGGCGGTTTCCTGCACGCACT

GGGTACCGCCCGTGCACACGGTATTTCACCGGTTGAACTGCTGCCGCACGCGGTCGGTATTGCC
GCAATCCTGCCGCCGGTGTTTTCGGAAGTTGCAGAACGTGTCGAAGCTGGCCGCCATGATGACG
CAAACGCTACCGTTAGTTCCGCTGCCGCATCACTGAGCCACCTGGTGGCCACCAGCGAAGCCTC
TCGTGTTGATGCGGGTGCCCTGAAAGCAATGAAACGCTACGCGGATGACCTGGTGGCAGCTGGC
CATGGTGATGCGGAAATCTCCCGCCTGGTGGAAGCCATGGGCGTTTAA

291 aa

MHHHHHHTTVAVIGLGPMGRALAAALLSAGYRVTVWNRTESKAAQLLSCGAHWAPTPGKAVAAGDL
TLINVVDHDAVDAVVHAAADAVAGRLLVGLSSDTPDRARSTAELVVAAGGRYLDGAIMTPTDVVGTAE
ASVLYAGPYDLFGGHRELFETLGQATWLGEDPGRAAAYDMALLDVFWTAAGGFLHALGTARAHGISP
VELLPHAVGIAAILPPVFSEVAERVEAGRHDDANATVSSAAASLSHLVATSEASRVDAGALKAMKRYA
DDLVAAGHGDAEISRLVEAMGV

IRED15: IRED from *Mycobacterium sp. JLS* (N-terminal His_6-Tag) 870 bp[1]

ATGCATCACCATCACCATCACATCTCCGTGCTGGGTCAGGGTCCGATGGGTCAAGCTCTGACCA
ATGCCCTGCTGCATGCGGGTTGCCGTACCACCGTTTGGAATCGTACCGCCGCACGTGCCGATGG
TGTCCGTGCACGCGGTGCTCGTTGGGCCGATAGCCCGGCCGATGCAATTGCAGCTGCGGATGT
TACCCTGGTCAACGTGGTTGACCAGGCAGTGCTGGATGACGTCGTGACCGCGGCAGGTCATGCA
GTTGCTGGTCGTGTTATTGTTGGTCTGGCAAGCGATACCCCGGACACCGCACGCGATACCGCTA
TGCTGGTTGAAAAACTGGGCGGTCGTTATCTGGATGGTGCGATTATGACGCCGACCGACACGAT
CGGCTCAGCGCATGCCTCGATTCTGTTTAGCGGTCCGCGCGATCTGTACGACACCCACCGTGAA
GTGTTCGATGTTCTGGCCACCACCACCTGGCTGGGTGATGACCCGGGTCGTGCTGCCGCCTTTG
ATATGGCCCTGCTGGACCTGTTCTGGACCAGTGTCTCCGGCGTGCTGCATGCAGTGAACGTTGC
ACGTGCTAATGGTATCTCTCCGATGGAACTGCTGCCGCATGCCCAGGGTATTGTCGGTATCCTGC
CGCCGATCGTGGATGAACTGCTGGAACGTATTGATGCCGACCGCCATGATGACTCCCGTGCCCA
AGTTGCATCTGTCGCAGCTAGTGTGCGCCACCTGATTGCCGCATCACGCGCAGTCGGCGTGGAT
GCCGGTGCACTGGAAGCTTTTCGCGGCTATGTGGATACCGCGGTTGCAGCTGGCTACGGTGCC
GATGAAATTTCACGTATCGGTCAAACGATGAGCTCTTAA

289 aa

MHHHHHHISVLGQGPMGQALTNALLHAGCRTTVWNRTAARADGVRARGARWADSPADAIAAADVTL

VNVVDQAVLDDVVTAAGHAVAGRVIVGLASDTPDTARDTAMLVEKLGGRYLDGAIMTPTDTIGSAHAS

ILFSGPRDLYDTHREVFDVLATTTWLGDDPGRAAAFDMALLDLFWTSVSGVLHAVNVARANGISPMEL

LPHAQGIVGILPPIVDELLERIDADRHDDSRAQVASVAASVRHLIAASRAVGVDAGALEAFRGYVDTAV

AAGYGADEISRIGQTMSS

IRED16: IRED from *Cellulosimicrobium cellulans* (N-terminal His_6-Tag) 957 bp[1]

ATGCATCACCATCACCATCACAGTGATCAGCCGGCCCGTCCGAGCGAACGTGCAGTTACCGTCC

TGGGTCTGGGTGCAATGGGTCGTGCACTGGCCGCAGCAGCTGTGGCGGCCGGTCATCCGACCA

CGGTTTGGAACCGTACCCCGGGTCGTGCCGGTGCCCTGGTGGGCGCAGGTGCTCGTGAAGCCA

CGTCAGTTCGCGATGCAGTCACCGCTAGCCCGCTGGTTGTGGCAGTGCTGCTGGACCATGCTTC

TGTTCACCAAACGCTGGACCCGGTTGCCGAAGCACTGGCAGGTCGTACCCTGGTGAATCTGGTT

ACCACGACCCCGGAAGAAAGCCGTGAACTGGCAGCTTGGGCGGGCTCTCATGGTGTCACCTATC

TGGACGGCGGTATTATGGCAGTGCCGGGTATGATCGGCGGTCCGGGTGCGGAAGTTCTGTACA

GCGGTTCTCGCGCCGCATTTGATGATGCCCGTCCGGTCCTGGATACCTGGGGCGGTAGCGCCT

GGTTTGGTGAAGACCCGGGTCTGGCCCCGCTGTATGATCTGGGCCTGCTGGCAGGCATGTACG

CGATGTTTGCCGGCTTTTTCCATGGTGTTGCCATGGTGGGTACCGCTGGTGTGAGTGCCAGCGA

TTTTGCACGTCGCGCAGCTCCGTGGATTGCCGCCATGACCGCAGAACTGGCTGGCTATGCGGAT

GTGATCGACCGTCGCGATTACGGCGGTCCGGGTCAGCAAAGTCTGGAATTTTCAGACCTGTCGG

ATATGGTTCGTGCTAGCGCCGAAGCGGGTCTGGCCACCGATGTTGTGGCAGCTGTGCAGGCCCT

GGTTCGTCGCCAAGTCGACGCAGGCCACGGTGCTGATGGCTTCGCACGTGCTGTTGAAAGCATT

CGCGAACCGTCTGGTACGGCCGACCGTACCCCGGATCTGACGGCGACCGCCGGCGGTGCGCG

CTAA

318 aa

MHHHHHHSDQPARPSERAVTVLGLGAMGRALAAAAVAAGHPTTVWNRTPGRAGALVGAGAREATS

VRDAVTASPLVVAVLLDHASVHQTLDPVAEALAGRTLVNLVTTTPEESRELAAWAGSHGVTYLDGGIM

AVPGMIGGPGAEVLYSGSRAAFDDARPVLDTWGGSAWFGEDPGLAPLYDLGLLAGMYAMFAGFFH

GVAMVGTAGVSASDFARRAAPWIAAMTAELAGYADVIDRRDYGGPGQQSLEFSDLSDMVRASAEAG

LATDVVAAVQALVRRQVDAGHGADGFARAVESIREPSGTADRTPDLTATAGGAR

IRED17: IRED from *Streptomyces tsukubensis* (N-terminal His_6-Tag) 952 bp[1]

ATGCATCACCATCACCATCACTCAGCGACCACGAACACCACGTCGGCAGATGGCGTGGCTGGTC
CGGGCGGTCCGGGCGGTCGTCCGCCGGTCACCGTGCTGGGTCTGGGTCAGATGGGTGCCGCA
ATTGCCGGTGCACTGCTGGCAGCTGGTCATCCGGTTACCGTCTGGAACCGCACGCCGGGTAAAG
CCGCGCCGCTGGTTGAACAAGGCGCAGTTCTGGCTGGTAGCGTCGCAGAAGCTGTGGCAGCTA
GTCCGCTGGTGCTGTCCGTGGTTCTGGATTATCCGGCGCTGTACGGCATTCTGGACCCGGAACC
GGACGCGCTGAAAGGTCGTGCGCTGGTCAATCTGACCACCGGTACCCCGGAACAGGCCGGTGA
AGCCGCGGAATGGGCAGCTCGTCATGGTGTTGATTATCTGGATGGTGCAATTATGACCACGCCG
CCGGGCGTCGGTACCCGTGAAGTGATGTTTCTGTACAGTGGCGATCGTGCCGTGTTTGATGCAC
ATCATGCCGCACTGGATGTTCTGGGTGAACCGCTGCATCTGGGCACCGAACCGGGTCTGGCAGC
TCTGTATGACGTGAATCTGCTGGGTCTGATGTGGGCCACCATGGCAGGTTGGCTGCATGGTACC
GCAGTTGTGGGTGCTGAAGGTACCCGTGCAGTTGATTTTACGGAAGTCGCGATTCGCTGGCTGG
GTACCGTTAACAATTTCATCCGTCGCTATGCGGCCCAGGTTGATGAAGGCGTCTACCCGGGTGAT
GACGCCACGGTGGACGTTCAGATCGCAGTTGTCGAACATCAACTGCACGCAGCTGAAGCGCGTG
GCGTGGATAACCGCCTGCCGGAACTGCTGAAAACCCTGATGCTGGAAGCGAATGCCAAAGGCCA
TGGTCAAGACAGCTTTGGCTCTGTGGTTGAAGTTCTGCGTAAAGGTGCCCGTCGCTAAA

316 aa

MHHHHHHSATTNTTSADGVAGPGGPGGRPPVTVLGLGQMGAAIAGALLAAGHPVTVWNRTPGKAA
PLVEQGAVLAGSVAEAVAASPLVLSVVLDYPALYGILDPEPDALKGRALVNLTTGTPEQAGEAAEWAA
RHGVDYLDGAIMTTPPGVGTREVMFLYSGDRAVFDAHHAALDVLGEPLHLGTEPGLAALYDVNLLGL
MWATMAGWLHGTAVVGAEGTRAVDFTEVAIRWLGTVNNFIRRYAAQVDEGVYPGDDATVDVQIAVV
EHQLHAAEARGVDNRLPELLKTLMLEANAKGHGQDSFGSVVEVLRKGARR

IRED18: IRED from *Paenibacillus ehimensis* (N-terminal His_6-Tag) 945 bp[1]

ATGCATCACCATCACCATCACAAACATAGCTCTCCGTCAGAAAAAGAAACCCACGAACAGGCCGG
TGCCGCGGGTCGTACCCCGGTCACGGTGATTGGCCTGGGTATGATGGGCTCCGCCCTGGCAGA
TGCTTTTCTGAACGCGGGTCATCGTACCACGGTGTGGAATCGCAGCGCCGATAAAGCGGATGCC
CTGGTGGCGAAGGGTGCGGTTCGTGCAGCTTCTGCCGCAGAAGCAGTTTCAGCTTCGCCGCTGA
TTGTGGTTTGCGTTCTGGATTATGAAGCCGTTCATGAAATTCTGGGTCCGGCAGGCGGTCGTCTG
GCTGGTCGTACCCTGGTGAACCTGACGAATGGCAAACCGGAACAGGCGCGTAAAGCAGCTAAAT

GGGCGAACGAACAAGGTGCCAATTATCTGGATGGCGGTATTATGGCAGTCCCGCAGATGATCGC

AGGCCCGGAAGCTTTTCTGCTGTATAGCGGTTCTCCGGAAGCCTTCGAAACCTATCGTCGCGAA

CTGGATGTTCTGGGTGCCGGTAAATACCTGGGCGAAGATGCAGGTCTGGCGGCCCTGTATGACC

TGGCACTGCTGACCACGGCTTACGGCCTGATTGGCGGCTTTTTCCATGCAGTGGCTCTGGTTGG

TACCGAAAAAGTGGAAGCAGCTGCGTTTACGGTTCTGGTCATTCCGTGGCTGCAGGCGATGATC

GCCAGTCTGCCGTCCCAGGCGCAAGCCATTGATGCGAACAATCACACCACGGACGTTAGTTCCC

TGAACATTAATAAAGTCGGCTTCGTGAACCTGATCGAAGCCTCACAGGAACAAGGTGTCAGCACC

GAACTGGTGGCGCCGATCCAGGCACTGGTTAATCGTGCAGTCGCTGATGGTTATGGTGCCGATG

GTCTGACCCGCCTGGTGGAACTGCTGAAAAAACCGCAACTGCTGTAA

314 aa

MHHHHHHKHSSPSEKETHEQAGAAGRTPVTVIGLGMMGSALADAFLNAGHRTTVWNRSADKADALV

AKGAVRAASAAEAVSASPLIVVCVLDYEAVHEILGPAGGRLAGRTLVNLTNGKPEQARKAAKWANEQ

GANYLDGGIMAVPQMIAGPEAFLLYSGSPEAFETYRRELDVLGAGKYLGEDAGLAALYDLALLTTAYG

LIGGFFHAVALVGTEKVEAAAFTVLVIPWLQAMIASLPSQAQAIDANNHTTDVSSLNINKVGFVNLIEAS

QEQGVSTELVAPIQALVNRAVADGYGADGLTRLVELLKKPQLL

IRED19: IRED from *Streptomyces sp. CNH287* (N-terminal His_6-Tag) 945 bp[1]

ATGCATCACCATCACCATCACAGCACCACGCGTTCTGCGGCCGCAACCGGTCCGGCAACCGCTC

CGAGCCCGGCCGTCGGCGTGCTGGGCCTGGGTCTGATGGGTCAGGCACTGGCTGCCGCCCTG

GTTGGCGCAGGTCATCCGACCACGGTCTGGAACCGCTCTCCGGATAAAGCAGCTGACCTGGTTG

CACAAGGTGCGACCCTGGCCGCATCAGCCCATGATGCAGTGACCACGTCGGAAGTGGTTATTGT

TTGCGTCACGGAATATGATGCAGTGCGTGCTCTGGTTGAACCGCTGGCCGAAGCACTGCGTGGT

CGTGTGCTGGTTAATCTGACCTCTGGTAGCTCTGCACAGGCTCGCGAATTTGCAGCTTGGGCGG

CCGAACATGGCGTTGATTACCTGGACGGTGCGCTGATGGCAATTCCGCCGGTGATTGGTACCCC

GCACGCATTCGTTCTGTATGCGGGCGGTCGTCCGGTCTACGAAGCAGCTGAACCGGTTCTGCGT

GTCCTGGCCCCGGCAGGTACCACGCATCTGGGCACGGATCACGGTCTGAGTTCCCTGTATGATG

TGGCCCTGCTGGGTCTGATGTGGGGTGCCCTGAACAGCTTTCTGCACGGTGCCGCACTGCTGG

GTACCGTCGGTGTGCCGGCAGCTGATTTTGCACCGTTCGCTAACCAGTGGCTGAATAGTGTCAC

GGGCTTCGTGTCCGCGTATGCGGCCCAAATTGATGCCGGTGAATACCCGGCGCATGACGCCAAA

ATCGAAACCCACCTGGCGACGATGCATCACCTGCGTCATGAAAGTGAAGCCGGCGGTGTTGATA

CCGCACTGCCGCTGTTTGTGCAGGCACTGGCTGACCAGGCGATTGCCCAAGGCCACGGCGGTT

CATCGTACGCAGCTGTGATCGAACAATTCCGCGCGGGTACCGCGGCCAGCTAA

314 aa

MHHHHHHSTTRSAAATGPATAPSPAVGVLGLGLMGQALAAALVGAGHPTTVWNRSPDKAADLVAQ

GATLAASAHDAVTTSEVVIVCVTEYDAVRALVEPLAEALRGRVLVNLTSGSSAQAREFAAWAAEHGV

DYLDGALMAIPPVIGTPHAFVLYAGGRPVYEAAEPVLRVLAPAGTTHLGTDHGLSSLYDVALLGLMWG

ALNSFLHGAALLGTVGVPAADFAPFANQWLNSVTGFVSAYAAQIDAGEYPAHDAKIETHLATMHHLRH

ESEAGGVDTALPLFVQALADQAIAQGHGGSSYAAVIEQFRAGTAAS

IRED20: IRED from *Streptomyces viridochromogenes* (N-terminal His_6-Tag) 942 bp[1]

ATGCATCACCACCACCACCACAATCGCCAGTTTACCAGCACCCGCCTGAACGCAATGACGGATAA

TGCCTCAAGCCCGACCCCGGTTACCCTGCTGGGCACCGGTGCGATGGGCAGCGCACTGGCTCG

TGCCTGGCTGGCCGCAGGTCATCCGGTTACCGTTTGGAATCGTACGCCGGCACGTGCAGAAGCT

CTGGCCGGTGAAGGTGCAGCTGTTGCCGCAAGCGCCGATGCAGCTGTTGCCGCCAATCGCCTG

GTGGTTGCCTGCCTGCTGGATGACGATTCTCTGGGTGAAGCACTGGCAACCGCTGACCTGGGCG

GTCGTGATCTGGTGAACCTGACCACCGGTACGCCGGGTCAGGGTCGTGCCCGTGCAGCTTGGG

CGGAAGCCCGCGGTGCCCGTTTTGTTGATGGCGGTATTATGGCAGTCCCGCCGATGATCGGCAG

TCCGGATTCCGGTGCCTTTGTGTTCTATAGCGGCTCTGCGGCCCTGTTTGAAGAACACCGTGATG

TGCTGGCCGTTCCGGCAGGTACCGCTTATGTCGGCGCAGATGCTGGTTTCGCAGCTCTGCATGA

CGTTGCGCTGCTGTCCGCCATGTACGGCATGTTTGGCGGTATTGCGCACGCCTTCGCACTGATT

CGTCGCGAAGACATCGCACCGACCGATTTTGCGCCGCTGCTGGTGTCTTGGCTGACCGCTATGG

CCCCGGCCGCACTGGAATCAGCCGGTAAACTGGAATCGGGTGACTATACCCGCGATGTCGTGTC

AAATCTGGCAATGCAGGTTGCTGGCATCCCGACCTTCCTGCGTACGGCCGACGAACAAGGTGTC

CGCCCGGATCTGGTGCGTCCGTACCTGGACCTGATGCGTCGCCGTCTGGAATGTGGTCCGCAC

GCTGACGAAGATACGACGGGTGTTATTGACCTGCTGACGGCAAGCTAA

313 aa

MHHHHHHNRQFTSTRLNAMTDNASSPTPVTLLGTGAMGSALARAWLAAGHPVTVWNRTPARAEAL

AGEGAAVAASADAAVAANRLVVACLLDDDSLGEALATADLGGRDLVNLTTGTPGQGRARAAWAEAR

GARFVDGGIMAVPPMIGSPDSGAFVFYSGSAALFEEHRDVLAVPAGTAYVGADAGFAALHDVALLSA

MYGMFGGIAHAFALIRREDIAPTDFAPLLVSWLTAMAPAALESAGKLESGDYTRDVVSNLAMQVAGIP

TFLRTADEQGVRPDLVRPYLDLMRRRLECGPHADEDTTGVIDLLTAS

IRED21: IRED from *Bacillus cereus* (N-terminal His_6-Tag) 972 bp[2]

ATGCATCACCATCACCATCACAAGAAAAACGATCAGTCTGAAAAAGAACAGAACATCAGTCAAGTT

TCCGATACGGACGTCTCGATGATGGAAAACCCGAATCGTAGCCCGGTGACCGTTATTGGTCTGG

GTCCGATGGGTCAGGCACTGGCAGGCACGTTTCTGATGAACGGCCATCCGACCACGCTGTGGAA

TCGCACCGCGGAAAAAGCCGATTATCTGGTTAGCCAAGGTGCCATTCTGAGCAACTCTGTGATCG

CGGCCGTTAGTGCATCCCCGCTGGTCATTATCTGCGTGCTGGATTACAATATTGTTCGTGAAGTC

CTGGCTCCGGCCGGTGATGCCCTGAAAGGTCGCACGCTGGTTAACCTGACCGCCGATAGCCCG

AAACGTGCACGCGAAATGGCTACCTGGGCAGCTCAGCATGGCGTCGATTATCTGGACGGTGCGA

TTATGACCCCGACGCCGACCATTGGTACGCCGGCCGCCAGCGTTCTGTACTCTGGTCCGGAAAG

TATTTTCAAAGCACACCAACCGACCCTGGCTTCCCTGGGCGGTACCACGTCATATCTGGGTGCAG

ACCCGGGTCGTGCAGCTGCGTACGATGTGGCGCTGCTGGACCTGTTTTGGACGTCAATGTCGGG

CTATGCCCATGCACTGGCTCTGGCGACCGCCGAAAATATCCCGGCCAAAGAATTCGCAGTGTAC

GCTCAGGGCATTATCGGTATTCTGCCGGATATCATGGCGTATCTGGCCAACGAAGTTGATTCTGG

CCACTACCCGGGTGACAAAAGTAATATTATCAGCGCATCTGCTGGCATGGAACATATTATCCACG

CCGCACAGCATCACGGTCTGGACGTCTCAGTGCTGTCGGCTGCGATGGCGGTGACCCAGCAAG

CCATTAACGAAGGCTATGGTACGGATGGTTTTTCCCGCCTGACCGAACTGCTGAAAAAACCGAGT

GCGTAA

323 aa

MHHHHHHKKNDQSEKEQNISQVSDTDVSMMENPNRSPVTVIGLGPMGQALAGTFLMNGHPTTLWN

RTAEKADYLVSQGAILSNSVIAAVSASPLVIICVLDYNIVREVLAPAGDALKGRTLVNLTADSPKRAREM

ATWAAQHGVDYLDGAIMTPTPTIGTPAASVLYSGPESIFKAHQPTLASLGGTTSYLGADPGRAAAYDV

ALLDLFWTSMSGYAHALALATAENIPAKEFAVYAQGIIGILPDIMAYLANEVDSGHYPGDKSNIISASAG

MEHIIHAAQHHGLDVSVLSAAMAVTQQAINEGYGTDGFSRLTELLKKPSA

IRED22: IRED from *Paenibacillus sp. HGF5* (N-terminal His_6-Tag) 957 bp[2]

ATGCATCACCATCACCATCACAAACCGAGTAAACAGCTGCAAGATCAGATGCTGGAAACCGAAAC

GCGTCAAACCCCGGCAAACGGCAGCCAGACCGCTGTGACGGTTCTGGGTCTGGGTCCGATGGG

TCAGGCACTGGCAGGTGCTTTTATTCGTAGCGGCCATTCTACCACGGTGTGGAATCGCACCAGC

GCGAAAGCCGATTCTCTGGTGAAACAGGGTGCGGTTCTGGCCCCGAGCGTTAAAGACGCAGTCC

TGGCTTCTCAGCTGATTATCATTTGCGTCCTGAACTATGATGCGGTCAATGCCGTGCTGAGCTCT

GAAACCAGCGCGCTGAAAGGTAAAACCCTGATTAACCTGACGGCGGATGTTCCGGAACGTGCCC

GCGAAATGGCAGAATGGGCTTTCCATAATGGCATCGATTACATTGACGGTGCGATTATGACCCCG

ATCCCGACGATTGGCGAACCGAGTGCCGTTATCCTGTATTCCGGTCCGGAAGATGTCTACCGTA

GTCGCCAATCCATTCTGGCATCACTGGGCGGTACCGCGTCGTTTCTGGGTGAAGACCCGGGTCG

TGCCGCGGCGTATGATGTCGCCCTGCTGGACGTGTTCTGGACGGCAATGTCTGGCTATGTGCAT

GCACTGGCTATTGCCCGTGCAGAAAACATCGCTGCGGAAGATATTGCGCCGTATGCCCATAATAT

CATTCGTATCATGCCGGACATTATGACCTATATGGCACACGATGCTGACCGCGGCGTGTACCCG

GGTGATAGTTCCAACCTGATTTCAAATGTTACGTCGATGGAACATATCATTCACGCCGCAGAACAT

CACGGCATCGATTCATCGGTTCTGATCGCTGCGAAAGCAATTGCTCAGAAAGCGATCCATGCCG

GCCACGGTGAAGACGGTTTTAGTCGCCTGATTGAATACAACCTGACCAGCCCGTAA

318 aa

MHHHHHHKPSKQLQDQMLETETRQTPANGSQTAVTVLGLGPMGQALAGAFIRSGHSTTVWNRTSA

KADSLVKQGAVLAPSVKDAVLASQLIIICVLNYDAVNAVLSSETSALKGKTLINLTADVPERAREMAEW

AFHNGIDYIDGAIMTPIPTIGEPSAVILYSGPEDVYRSRQSILASLGGTASFLGEDPGRAAAYDVALLDV

FWTAMSGYVHALAIARAENIAAEDIAPYAHNIIRIMPDIMTYMAHDADRGVYPGDSSNLISNVTSMEHII

HAAEHHGIDSSVLIAAKAIAQKAIHAGHGEDGFSRLIEYNLTSP

IRED23: IRED from *Chitinophaga sp. JGI 0001002-D04* (N-terminal His_6-Tag) 909 bp[2]

ATGCATCACCATCACCATCACACGGCAACCACGAAACATCCGGCTATTAGCGTCATTGGTCTGGG

TTCTATGGGTGCCGCACTGGCTCGTGCCCTGGTTAGCAAAGGCTTTCAGGTTACCGTCTGGAAC

CGCAATATGGAAAAAGCCCAACCGCTGATTGCGGCTGATGCCATTGCCGCGGCAGATGCAAAAG

CTGCCATTGAAGCAAGTCCGGTCATCGTGGTTTGCGTGTCCGAATATAAAGTTACCCGTAAAATT

CTGGAAGCAGATGGTGTTGCCCCGGCACTGAAAGGCCGTACGCTGGTCCAGCTGTCTACCGGTA

CGCCGAAAGATGCGCGCGAACTGGACACCTGGGCGAAACAGCAAGGTGCCTGCTGTCTGAACG

GCGATATTATGGCGTGGCCGAAACAGATGGGTACCGACGCCGCAACGATCAGCGTCTCTGGCGA

TGCCGACGTGTATCGTCAGCAAGAAGATGTTCTGCGCGCTCTGGCGGGCAATGTCGTGTATCTG

GGTGCAGAACCGGGTGCTTCAGGCGGTCTGTTTCATGCCGTTCTGGCATATCTGGCTGGCTCGT

GGATCGGTTTCTGTCACGGCGCGCTGGTTGCGGAAAAAGAAGGTCTGCGTCCGGAAGACCTGG

GCATTCTGCTGGAACAGATTAGTCCGATCCTGTCCGCCGAACTGAAACACATGGGTGAAGTGATC

CAACACGGCCGCTTCTCAGATCCGGAATCGACCGTGAAAACCACGGGTGAAGACCTGCTGCTGC

TGGTTCAGCAAGCAAAAGAAGCTGGCATTAACTCAGAACTGCCGGAATTTGCTGCGAAACTGTTC

AAACAGGCGATGGATGCCGGCTACGGTCAAGAAGAACACGCCGCAGTGATCAAAGTTCTGCGCC

AGACCGCGTAA

302 aa

MHHHHHHTATTKHPAISVIGLGSMGAALARALVSKGFQVTVWNRNMEKAQPLIAADAIAAADAKAAIE

ASPVIVVCVSEYKVTRKILEADGVAPALKGRTLVQLSTGTPKDARELDTWAKQQGACCLNGDIMAWP

KQMGTDAATISVSGDADVYRQQEDVLRALAGNVVYLGAEPGASGGLFHAVLAYLAGSWIGFCHGALV

AEKEGLRPEDLGILLEQISPILSAELKHMGEVIQHGRFSDPESTVKTTGEDLLLLVQQAKEAGINSELPE

FAAKLFKQAMDAGYGQEEHAAVIKVLRQTA

IRED24: IRED from *Glycomyces tenuis* (N-terminal His_6-Tag) 897 bp[2]

ATGCATCACCATCACCATCACAGTGCGAAAAAATCCGTGACCGTTCTGGGTCTGGGCCCGATGG

GTCGTGCTACCGTCAAAATTCTGCTGGAAGCGGGCCTGGATGTCACCGTGTGGAACCGCACGCC

GGGTAAAGCAGAAGCTCTGGCGGAACTGGGTGCCGCACCGGCAGCTACCGTGGCAGACGCAAT

TGCGGCCAGTGATACGGTTCTGCTGTCCCTGATCCATTATGACGCTATGTACGGTGTCCTGGAAC

AGGGCCCGGCAGATCTGACCGGTAAAACGATTGTGAACCTGAGCTCTGACTCACCGGCTAATAC

CGCGAAAGGTGCAGCTTGGGTTCTGGATCGTGGCGGTCGCTTTCTGACCGGCGCCTATATGACG

CAGTCCGATGACATCCGTCATCCGGCCTCACACCTGTACGTGTCGGGTCCGGCAGAACTGCATG

ATGAACTGCGTCCGCTGCTGGAACTGCTGTGTGCCAATGTTTATCTGGGTCCGGATTACGGCCT

GGCCCAGCTGTATTACCAAGCCGGCCTGGCAATGTTTCACGCGTATCTGATCAGCCTGCAGCAA
GCTCTGGCGATGATTGAACGTGGCGGTGGCGATATCGACACCTTCCTGGAACTGTCTAAAGATG
ACGCAGATAGCCAGCGCGACTTTTCTGTGTACTTTGCCCAGGCCGCAAAACAAGGTGGCTGGGG
TGATCTGGCCTCACTGAAAATGATGCATGCCGGCGCACAACACGTTATTGATACCTCGGAAGACG
CCGGTACGGATGCAGAACTGACCAAAACGGTCCAGGACTATTACCAACGTGCGCTGGATGCCAC
CGAACGTACCGGTGCCATTGTTCCGGTCTATCAGATTATCCGTGGTGATAACGGCAATGAATAA

298 aa
MHHHHHHSAKKSVTVLGLGPMGRATVKILLEAGLDVTVWNRTPGKAEALAELGAAPAATVADAIAAS
DTVLLSLIHYDAMYGVLEQGPADLTGKTIVNLSSDSPANTAKGAAWVLDRGGRFLTGAYMTQSDDIRH
PASHLYVSGPAELHDELRPLLELLCANVYLGPDYGLAQLYYQAGLAMFHAYLISLQQALAMIERGGGDI
DTFLELSKDDADSQRDFSVYFAQAAKQGGWGDLASLKMMHAGAQHVIDTSEDAGTDAELTKTVQDY
YQRALDATERTGAIVPVYQIIRGDNGNE

IRED25: IRED from *Nocardia brasiliensis ATCC 700358* (N-terminal His_6-Tag) 900 bp[2]
ATGCATCACCATCACCATCACAGCGAACAGCATACCCCGCGTAGTGTTTCCGTGGTTGGCCTGG
GTCCGATGGGCCAAAGTATGGTCCGTGCACTGCTGGACGCTGGTGTCGAAGTGACCGTTTGGAA
CCGCAGCACGGATAAAGTCGATGCCATGGTGGAACTGGGTGCCGTTCGTGCGGAAACCGTTGC
CGCCGCACTGGCTGCGAATGATGTCACCGTGCTGAGCCTGACGCATTATGCCGCAATGTACTCT
GTGCTGGAACAGGCTGCGGACCAACTGGCCGGTAAAGTTATTGTCAACCTGAGCTCTGATAGTC
CGGAAAAAGCGCGTAAGGGTGCGGAATGGGTCCGTTCCCATGGTGCAGAATTTCTGAGCGGCG
GTGTGATGTCTGCAGGCGACAATATTGCACATCCGGCTAGTTATATCTTTTACTCCGGTCCGCGT
GAAGTTTTCGATGCACACGCTGAACTGCTGCGCCCGCTGTCACCGCAGGAATATCTGGGCACCG
ATGACGGTCTGTCGCAGGTGTATTACCAAGCGCTGCTGACCATTTTTCATCCGTGGCTGCTGGCC
TTCGATCAGGCGACGGCCATGATCGAACGTTCAGGCAACTCGATTGCGCAATTTATCCCGTTCGC
CGTTCGCAGCGCCGCAGCTTATCCGTACTTTATGGAAGAATTCTCTGTGGCGAACCAGAATGGC
GGTTGGGCGACCCTGGCCTCTCTGAAAATGATGGATGCAGGCGCTCAACATATTATCGACGCAA
GTGAAGAAGTGGGTGTTGATGCGACCTTCTCCCACACGGCGCAGGCCTATTGGCGTAAAGCGGT
GGCGGCCTCAGAAGAAAAAGGCGAAGCAGTTTCGACCTACGCTCTGATGCGCGGTGCAGATGCT
TAA

299 aa

MHHHHHHSEQHTPRSVSVVGLGPMGQSMVRALLDAGVEVTVWNRSTDKVDAMVELGAVRAETVAA

ALAANDVTVLSLTHYAAMYSVLEQAADQLAGKVIVNLSSDSPEKARKGAEWVRSHGAEFLSGGVMSA

GDNIAHPASYIFYSGPREVFDAHAELLRPLSPQEYLGTDDGLSQVYYQALLTIFHPWLLAFDQATAMIE

RSGNSIAQFIPFAVRSAAAYPYFMEEFSVANQNGGWATLASLKMMDAGAQHIIDASEEVGVDATFSHT

AQAYWRKAVAASEEKGEAVSTYALMRGADA

IRED26: IRED from *Nitratireductor pacificus pht-3B* (N-terminal His_6-Tag) 936 bp[2]

ATGCATCACCATCACCATCACACCACGACCATTTGCGTGATTGGTGCGGGTCGTATGGGCAGCT

CTCTGGCTCGTACCCTGCTGAATGCGGGTCGTCCGACCTGGGTTTGGAATCGTACCGCCGCACG

TTGTGCGCCGCTGGTCGCTCTGGGTGCCAAAACCGCAAATGCTCTGGCCGATGCGGTGCAGGC

CAGCGAACTGATTCTGATCAATGTTATTGATCATGACGCCTCTGCAGCTCTGCTGCGCCAGGAAG

CGGTTAGCTCCGCCCTGAGCGGCCGTACGGTCATCCAACTGACCAGCGGTTCTGCCCGTCTGG

CACGCGAAGAAGCGCTGTGGGTTGAAGCTCAGGGTGCCCGTTATCTGGATGGTGCCATTATGGC

AACCCCGGACTTTATTGGTCGTCCGGAAGCCGCACTGCTGTACAGTGGTTCCCTGGCAAGCTTC

GAAGCTCATCGCGATATTCTGCTGACCCTGGGCGGTCGTTCAGCACATGTTGGTGATGTTCCGG

GTCAGGCAAGCGCTCTGGATACCGCACTGCTGACCCAGATGTGGGGCGGTCTGTTTGGCGCACT

GCAGGGTATGGCGGTCGCCGATGCAGAAGGCCTGAGCCTGGATGTGTTTCGCGACCAACTGTCT

GCGTTCAAACCGGTGGTTGATGCAGCTCTGTATGATACGATCGACCGTACCGCCGCACGTCGCT

TTGCTGGTGATGCCGAAACGCTGGCGTCACTGGGTGCCCATCACTCGGCATTCACCCACCTGCT

GGAAGCATGCGAAGATCAGGGCCTGGACCAAGGTCTGCCGCGCGAAATGGCGCGTCTGTTTCG

CGAAGGCCTGAGTCGTAACGGTCCGGAAGCCGATTTTGCATCCCTGGCTCCGCTGCTGCGCGG

CGGTCCGTCAAGCGAAGCAGGTGAAGTGCGTCCGGACGCGTAA

311 aa

MHHHHHHTTTICVIGAGRMGSSLARTLLNAGRPTWVWNRTAARCAPLVALGAKTANALADAVQASEL

ILINVIDHDASAALLRQEAVSSALSGRTVIQLTSGSARLAREEALWVEAQGARYLDGAIMATPDFIGRPE

AALLYSGSLASFEAHRDILLTLGGRSAHVGDVPGQASALDTALLTQMWGGLFGALQGMAVADAEGLS

LDVFRDQLSAFKPVVDAALYDTIDRTAARRFAGDAETLASLGAHHSAFTHLLEACEDQGLDQGLPRE

MARLFREGLSRNGPEADFASLAPLLRGGPSSEAGEVRPDA

IRED27: IRED from *Mesorhizobium sp. L48C026A00* (N-terminal His_6-Tag) 933 bp[2]

ATGCATCACCATCACCATCACGCAAGCAACGTGTGCGTTCTGGGTGCTGGCCGTATGGGCAGCT
CTATTGCCCGTACCCTGCTGGATCGCGGTTATCCGACCTGGGTCTGGAATCGTACCGCCGCAAA
ATGTGAACCGCTGGCAGCTCTGGGTGCGAAAGTCGCCAGTTCCGTGCAGGAAGGCATTCAAGCG
GCCGAAGTGGTTATTATCAACGTTCTGGATTACGCAGCTTCAGACGCCCTGCTGAAACGTGATGG
TATCGCATCGGCTCTGGCGGGCAAAGCGGTCGTGCAACTGACCTCAGGCTCGCCGCGTCTGGC
ACGTGAAGAAGCTCGCTGGGTGGAAGCACATGGTGCTGGCTATCTGGATGGTGCGATTATGGCC
ACCCCGGACTTTATCGGCAAACCGGAAACGGCCATGCTGTATAGCGGTTCTCGTGATGTTTACGA
AAAACACAAACCGCTGCTGTTTGCCCTGGGCGGTGGCACCAATTATGTTGGTGAACTGCCGGGT
CAGGCATCCGCACTGGATACCGCACTGCTGACCCAGATGTGGGGTGGCCTGTTTGGTGCACTGC
AAGGCATGGCTGTGGCGGAAGCCGAAGGCCTGGATCTGGAAACGTTTCGTAACCATCTGAGTGC
GTTCAAACCGGTTGTCGACGCCTCCCTGTTTGATCTGGTTGACCGCACCAATGCGCGTCGCTTC
GCCGGTGATGACGCAACGCTGGCTAGCCTGGGCGCACATTATTCTGCTTTCCAGCACCTGCTGG
AAGCGTGCGAAGAACGTGGTCTGGATGCGGCCATGCCGCGTGCAATGGACATGATCTTTCGCCA
AGCGCTGAGTCTGGGCTCCATGGAAGATGATCTGGCCAGCCTGGCACTGCTGTTCCGTAATGGT
TCACCGCGTCAGAGCCGTGAACCGGCAAATGCTTAA

310 aa

MHHHHHHASNVCVLGAGRMGSSIARTLLDRGYPTWVWNRTAAKCEPLAALGAKVASSVQEGIQAAE
VVIINVLDYAASDALLKRDGIASALAGKAVVQLTSGSPRLAREEARWVEAHGAGYLDGAIMATPDFIGK
PETAMLYSGSRDVYEKHKPLLFALGGGTNYVGELPGQASALDTALLTQMWGGLFGALQGMAVAEAE
GLDLETFRNHLSAFKPVVDASLFDLVDRTNARRFAGDDATLASLGAHYSAFQHLLEACEERGLDAAM
PRAMDMIFRQALSLGSMEDDLASLALLFRNGSPRQSREPANA

IRED28: IRED from *Aeromonas veronii AER39* (N-terminal His_6-Tag) 897 bp[2]

ATGCATCACCATCACCATCACCGTCATCTGAGCGTGATTGGCCTGGGTGCCATGGGCTCTGCAC
TGGCTACCACGCTGCTGAAAGCGGGTCATCCGGTGACCGTTTGGAATCGCAGCGCCGCAAAAGC
GGCTCCGCTGCAGGCACTGGGTGCTACCCTGGCCCCGAGTGTGGGTGCCGCAATTGCAGCTTC
CGATATCACGCTGGTCTGCGTGGACAATTATGCAGTTTCACAACTGCTGCTGGATGAAGCCAGCG
ATGCCGTTGCAGGTAAACTGCTGGTGCAGCTGAGTACCGGCTCCCCGCAAGGTGCACGTGCTCT
GGAAAGCTGGTCTCATGCCCGTGGCGCACGCTACCTGGATGGTGCAATTCTGTGCTTTCCGGCT

CAGATCGGCACCTCAGACGCATCGATTATCTGTAGCGGTGCTTCTGCGGCCTTCAGCGAAGCCG
AACCGGTCCTGTCTCTGCTGGCCCCGACCCTGGATCATGTTGCCGAAGCGGTTGGTGCAGCTGC
CGCACAGGACTGTGCGGTTGCAGCTTATTTTGCCGGCGGTCTGCTGGGTGCACTGCACGGTGCT
CTGATTTGCGAAGCGGAAGGTCTGCCGGTTGCGAAAGTCTGTGCCCAGTTTAGTGAACTGTCCC
CGATCCTGGGCGGTGATGTGGCCCATCTGGGCAAAACCCTGGCAAGTGGTGATTTCGACCACCC
GTACGCCTCTCTGAAAACCTGGAGCGCCGCAATTAGCCGCCTGGCTGGTCATGCCACCGATGCA
GGTATCGACAGCCGTTTTCCGCGCTTCGCAGCTGACCTGTTTGAAGAAGGCGTTGCGCAGGGCT
TCGGTCAGCAAGAAGTTTCCGCGCTGATCAAAGTCCTGCGTGCCCGCAACGGTGCGGCCCAATA
A

298 aa

MHHHHHHRHLSVIGLGAMGSALATTLLKAGHPVTVWNRSAAKAAPLQALGATLAPSVGAAIAASDITL
VCVDNYAVSQLLLDEASDAVAGKLLVQLSTGSPQGARALESWSHARGARYLDGAILCFPAQIGTSDA
SIICSGASAAFSEAEPVLSLLAPTLDHVAEAVGAAAAQDCAVAAYFAGGLLGALHGALICEAEGLPVAK
VCAQFSELSPILGGDVAHLGKTLASGDFDHPYASLKTWSAAISRLAGHATDAGIDSRFPRFAADLFEE
GVAQGFGQQEVSALIKVLRARNGAAQ

IRED29: IRED from *Aeromonas veronii AER397* (N-terminal His_6-Tag) 897 bp[2]

ATGCATCACCATCACCATCACCGTCATCTGTCAGTGATTGGCCTGGGTGCAATGGGCTCGGCACT
GGCTACCACGCTGCTGAAAGCGGGTCATCCGGTGACCGTTTGGAATCGCAGCGCCGCAAAAGC
GGCTCCGCTGCAGGCACTGGGTGCTACCCTGGCCCCGAGCGTGGGTGCGGCCATTGCAGCTTC
TGATATCAACCTGGTCTGCGTGGACAATTATGCCGTTAGTCAGCAACTGCTGGATGAAGCGAGCG
ATGCCGTGGCAGGTAAACTGCTGGTTCAGCTGAGTACCGGCTCCCCGCAAGGTGCACGTGCTCT
GGAAAGCTGGTCTCATGCGCGTGGCGCACGTTACCTGGATGGTGCAATTCTGTGCTTTCCGGAT
CAGATCGGCACCTCAGACGCGTCGATTATCTGTAGCGGTGCCTCTGCGGCCTTCTCCGATGCAG
AACCGGTCCTGCGTCTGCTGGCCCCGACCCTGGACCATGTTGCCGAAGCGGTTGGTGCAGCTG
CCGCACAGGATTGTGCAGTTGCAGCTTATTTTGCTGGCGGTCTGCTGGGTGCACTGCACGGTGC
TCTGATTTGCGAAGCGGAAGGTCTGCCGATCGCGAAAGTTTGTGCCCAATTTAGTGAACTGTCCC
CGATTCTGGGCGGTGATGTCGCTCATCTGGGCAAAACCCTGGCGAGCGGTGATTTCGACCACCC
GTACGCCTCTCTGAAAACCTGGTCAGCGGCCATTAGCCGCCTGGCTGGTCATGCCACCGATGCA

GGTATCGACAGTCGTTTTCCGCGCTTCGCAGCTGACCTGTTTGAAGAAGGCGTTGCGCAGGGCC
TGGGTCAGCAAGAAGTCTCTGCCCTGATCAAAGTGCTGCGTGCACGCAATGGTGCGGCCCTGTA
A

298 aa
MHHHHHHRHLSVIGLGAMGSALATTLLKAGHPVTVWNRSAAKAAPLQALGATLAPSVGAAIAASDINL
VCVDNYAVSQQLLDEASDAVAGKLLVQLSTGSPQGARALESWSHARGARYLDGAILCFPDQIGTSDA
SIICSGASAAFSDAEPVLRLLAPTLDHVAEAVGAAAAQDCAVAAYFAGGLLGALHGALICEAEGLPIAKV
CAQFSELSPILGGDVAHLGKTLASGDFDHPYASLKTWSAAISRLAGHATDAGIDSRFPRFAADLFEEG
VAQGLGQQEVSALIKVLRARNGAAL

IRED30: IRED from *Streptomyces aurantiacus* (C-terminal His_6-Tag) 936 bp[3]
ATGTCACAGTCCGTCACTGTCATCGGTCTCGGCCCCATGGGGCAGGCGATGGCCGCCGCGTAT
CTGGACCGCGGCTACGAGGTCACGCTCTGGAACCGCACCGCGTCCCGGGCGGACGCCCTGGT
GGCGCGCGGCGCCAAGCTGGCCGCCACCCCCGAACAGGCGCTGTCGGCCAATGAGTTGGTGAT
ACTGAGCCTGATCGACTACGACGCGATGTACGGCGTGCTCGAGGGCGCGGAGGAGGCGGTCGC
GGGCCGGGTGCTGGTGAACCTCAGCTCGGACACCCCGGAGAAGGCCCGCGCGGCCGCGCGCC
GGGTGGCGGAGCTGGGCGGCACGCACCTCACCGGCGGCGTCCTCTCGCCGCCGCCGGGGATC
GGCAGCCCGGACATGTCGACGTTCTACAGCGGCCCGCGCGCCGCGTACGACCAGCACCGCGC
GGCCCTCGAAGTGATCACCGGCAAGACGGACTACCGGGGCGAGGACCCGGGCCTGGCCGCCC
TCATGTACCAGCTCAACATGGTCGTCTTCTGGCCGGCGATGCTCTCGTACTGGCAGGCCGTGGC
CCTGGCCGACGCGCACGGGCTCACGGCGGCGGACATCGCCCCGTACGTCTCCGAGAACTTCGC
GGGGATGGGGCAGTTCATCGACTTCTACGCGGCCCGCATCGACGCCGGCAACCACGCCGGCGA
CGTCGACCGCCTCTCGATGGGCGTCGCCAGCATGGAACACGTCGTCCACACGAACGCGGACTC
GGGCGTGGACACGGCGTTCCCGCGTGCGGTCCTCGACGCGTTCCACCGGGGCGCCGACGCCG
GTTTCGGCGCGGACAGCTTCTCCAGCGTGATCAAACTGATGAAGAAGCAGCCCGAGGATCCGAA
TTCGAGCTCCGTCGACAAGCTTGCGGCCGCACTCGAGCACCACCACCACCACCACTGA

311 aa

MSQSVTVIGLGPMGQAMAAAYLDRGYEVTLWNRTASRADALVARGAKLAATPEQALSANELVILSLID
YDAMYGVLEGAEEAVAGRVLVNLSSDTPEKARAAARRVAELGGTHLTGGVLSPPPGIGSPDMSTFYS
GPRAAYDQHRAALEVITGKTDYRGEDPGLAALMYQLNMVVFWPAMLSYWQAVALADAHGLTAADIA
PYVSENFAGMGQFIDFYAARIDAGNHAGDVDRLSMGVASMEHVVHTNADSGVDTAFPRAVLDAFHR
GADAGFGADSFSSVIKLMKKQPEDPNSSSVDKLAAALEHHHHHH

IRED31: IRED from *Streptomyces sp. GF3546* (C-terminal His_6-Tag) 939 bp[3]

ATGTCCAAACAGTCTGTTACCGTGATTGGGCTTGGTCCAATGGGCCAAGCGATGGTTAACACGTT
CCTGGATAACGGCCACGAAGTGACTGTCTGGAATCGCACCGCTTCAAAGGCAGAAGCACTGGTT
GCTCGTGGTGCAGTTCTGGCCCCTACCGTAGAAGATGCCCTGAGTGCCAACGAACTGATTGTGC
TGTCTCTGACCGATTATGACGCTGTCTATGCGATTTTGGAACCCGTTACTGGCAGCTTATCGGGC
AAAGTGATTGCCAATCTCAGCAGTGACACACCGGATAAAGCACGCGAAGCCGCGAAATGGGCGG
CCAAACACGGTGCCAAACATCTGACGGGTGGTGTACAGGTGCCTCCACCGCTGATTGGGAAACC
GGAATCATCGACCTATTACTCCGGCCCGAAAGACGTCTTTGATGCGCATGAGGACACCCTCAAAG
TCCTGACTAATGCCGACTACCGTGGTGAGGATGCGGGTCTGGCGGCAATGTACTATCAGGCGCA
GATGACCATCTTTTGGACAACCATGTTGTCGTACTACCAAACACTGGCTTTAGGCCAAGCGAATG
GCGTGAGCGCGAAGGAGTTACTCCCGTATGCAACGATGATGACGTCCATGATGCCCCACTTTCT
GGAACTTTATGCGCAGCATGTCGACTCTGCCGATTATCCGGGAGATGTGGATCGCCTTGCAATG
GGAGCGGCAAGTGTTGATCACGTACTGCATACCCATCAGGATGCGGGCGTAAGCACGGTGTTAC
CAGCGGCTGTTGCAGAGATCTTCAAAGCCGGGATGGAGAAGGGCTTTGCCGAAAACAGCTTCAG
CTCACTGATCGAAGTGTTGAAGAAACCGGCTGTGGGGGATCCGAATTCGAGCTCCGTCGACAAG
CTTGCGGCCGCACTCGAGCACCACCACCACCACCACTGA

312 aa

MSKQSVTVIGLGPMGQAMVNTFLDNGHEVTVWNRTASKAEALVARGAVLAPTVEDALSANELIVLSLT
DYDAVYAILEPVTGSLSGKVIANLSSDTPDKAREAAKWAAKHGAKHLTGGVQVPPPLIGKPESSTYYS
GPKDVFDAHEDTLKVLTNADYRGEDAGLAAMYYQAQMTIFWTTMLSYYQTLALGQANGVSAKELLPY
ATMMTSMMPHFLELYAQHVDSADYPGDVDRLAMGAASVDHVLHTHQDAGVSTVLPAAVAEIFKAGM
EKGFAENSFSSLIEVLKKPAVGDPNSSSVDKLAAALEHHHHHH

Gene expression

Expression of IREDs in deep-well plates

For the colorimetric pH shift assay all 31 imine reductases as well as the empty pET-22b(+) vector are expressed simultaneously in deep-well plates. 1.5 mL autoinduction medium (TB medium, 2 g L^{-1} lactose,0.5 g L^{-1} glucose) and 100 µg μL^{-1} carbenicillin is added to each well. Wells are inoculated fourfold for each IRED directly from agar plates and incubated for 3 h at 37 °C in Heidolph Titramax 1000 incubator. Production of recombinant protein is induced over night (ca. 20 h) at 20 °C and 1350 rpm. Cells are harvested by centrifugation at 4000 rpm and 4 °C for 30 minutes and cell pellets are stored at -20 °C.

For cell lysis, the cell pellets are resuspended in lysis-buffer (50 mM KPi pH 7, 2 mg/mL lysozyme, 0.04 mg/mL DNAseI) and incubated for 1 h at 37 °C and 1350 rpm in Heidolph Titramax 1000 incubator. Cell suspensions are frozen at -20 °C for 30 minutes and then incubated again at 37 °C and 1350 rpm for 30 minutes. The crude extract is obtained as supernatant after centrifugation at 4 °C at 4000 rpm for 30 minutes and then directly used in the colorimetric pH shift assay.

Expression of IREDs for biotransformations

A preculture of *E. coli* BL21(DE3) carrying the recombinant plasmid with the IRED gene was cultivated over night at 37 °C in 10 mL LB medium, containing 100 µg mL^{-1} of carbenicillin. The main culture in 300 mL TB medium, containing 100 µg mL^{-1} of carbenicillin, was inoculated with the preculture to a final concentration of 1%. At an OD_{600} between 0.4 und 0.6, the production of recombinant protein was induced by addition of isopropyl-β-D-thiogalactopyranoside (IPTG) to a final concentration of 0.5 mM. Cultures were shaken at 25 °C for 20 h and harvested by centrifugation at 4000 g and 4 °C for 30 min and cell pellets were stored at -20 °C. Cells were resuspended in 3 mL 50 mM KP_i pH 7 per g wet cell weight. Cell disruption was performed by sonification (Bandelin Sonopuls HD 2070) with 3x 120 s burst (5 cycles, 20% energy) and 120 s intervals and both steps being carried out on ice. After centrifugation at 21 000 g for 5 min at 4 °C, cell debris was removed and the supernatant was centrifugated again at 21 000 g for 25 min at 4 °C. The crude extract was obtained as supernatant, whereas inclusion bodies were obtained as pellets. The protein concentration was determined using the Bradford assay against BSA as a concentration standard. Overexpression of imine reductases was checked by SDS-Page (Supplementary Figure 39).

Synthesis of substrates

General Procedure 1 (GP 1): α-chlorination of aldehydes and ketones

The synthesis is conducted to an adapted procedure from Rodig *et. al*.[4]

Sulfuryl chloride (0.50 mol, 1.00 equ.) is added under cooling at vacuum of 900 mbar to the aldehyde or ketone, keeping the temperature stable at 40 °C. The reaction mixture is stirred afterwards for 2 h at 45 °C and 900 mbar. The crude product is purified by fractional distillation.

2-chloro-2-methylpropanal

2-Chloro-2-methylpropanal was prepared according to GP 1 using isobutanal (92.0 mL, 1.00 mol) and sulfuryl chloride (81.0 mL, 1.00 mol). The crude product is purified by fractional distillation (150 °C oil bath temperature), yielding 2-chloro-2-methylpropanal (67.6 g, 0.63 mol, 63%) as colorless oil.

^{1}H-NMR (500 MHz, $CDCl_3$): δ (ppm) = 9.37 (s, 1 H, **H**-C=O), 1.58 (s, 6 H,C**H_3**)

^{13}C-NMR (126 MHz, $CDCl_3$): δ (ppm) = 195.1 (H-**C**=O), 69.4 (**C**-Cl), 26.0 (**C**H_3)

MS (EI) m/z calculated for C_4H_8ClO $[M+H]^+$: 107.02, found: 107.00

The analytical data corresponds with literature data.[4]

3-chloro-3-methyl-2-butanone

3-Chloro-3-methyl-2-butanone is prepared according to GP 1 using 3-methylbutanone (53.2 mL, 0.50 mol) and sulfuryl chloride (40.5 mL, 0.5 mol). The crude product is purified by fractional distillation (160 °C oil bath temperature), yielding 3-chloro-3-methyl-2-butanone (46.3 g, 0.38 mol, 77%) as colorless oil.

^{1}H-NMR (500 MHz, $CDCl_3$): δ (ppm) = 2.29 (s, 3 H, **CH_3**-C=O), 1.59 (s, 6 H, 2x **CH_3**).

^{13}C-NMR (126 MHz, $CDCl_3$): δ (ppm) = 204.7 (**C**=O), 70.4 (**C3**-Cl), 28.5 (2x **C**H_3), 24.1 (O=C-**C**H_3).

MS (EI) m/z calculated for $C_5H_{10}ClO$ $[M+H]^+$: 121.03, found: 121.0.

2-chloro-2-methyl-1-phenyl-1-propanone

2-Chloro-2-methyl-1-phenyl-1-propanone is prepared according to GP 1 using isobutyrophenone (45.0 mL, 0.30 mol) and sulfuryl chloride (24.3 mL, 0.30 mol). The crude product is purified by fractional distillation (30 mbar), yielding 2-chloro-2-methyl-1-phenyl-1-propanone (48.8 g, 0.27 mol, 89%) as yellow oil.

^{1}H-NMR (500 MHz, $CDCl_3$): δ (ppm) = 8.15–8.13 (m, 2 H, Ar-**H**), 7.54–7.51 (m, 1 H, Ar-**H**), 7.45–7.42 (m, 2 H, Ar-**H**), 1.88 (s, 6 H, 2x C**H$_3$**).

^{13}C-NMR (126 MHz, $CDCl_3$): δ (ppm) = 197.1 (**C**=O), 134.5 (Ar-**C**), 132.6 (Ar-**C**), 130.1 (Ar-**C**), 128.2 (Ar-**C**), 68.4 (**C**-Cl), 30.6 (2x **C**H$_3$).

MS (EI) m/z calculated for $C_{10}H_{11}ClO$ $[M]^+$: 182.05, found: 182.1.

The analytical data corresponds with literature data.[5]

GP 2: Synthesis of 3-thiazolines

The synthesis is conducted according to Martens *et al*.[6] and Reiners *et al*.[7]

Acetone (20.0 mL, 260 mmol, 1.00 equ.), ammonia-solution (13.3 M, 34. mL) and sodiumhydrogensulfid-monohydrate (14.58 g, 260 mmol, 1.00 equ.) were dissolved and cooled to 0 °C. α-Chlorated aldehyde or ketone (260 mmol, 1.00 equ.) is dissolved in dichloromethane (100 mL) and added to the yellow mixture, keeping the temperature under 10 °C. The reaction mixture is stirred over night, phases are seperated and the aqueous phase is extracted with dichloromethane (3x30 mL). The combined organic phases are dried over magnesium sulfate, the solvent is evaporated *in vacuo* and the crude product is purified by fractional distillation.

2,2-Dimethyl-3-thiazoline (1a)

2,2-Dimethyl-3-thiazoline (**1a**) is prepared according to GP 2 using acetone (45.0 mL, 610 mmol), ammonia-solution (13.3 M, 41.0 mL), dH_2O (56.0 mL), sodiumhydrogensulfid-monohydrate (13.5 g, 240 mmol) and aqueous α-chloroacetaldehyde (27.5 mL, 200 mmol), yielding **1a** (9.89 g, 86.7 mmol, 43%) *via* fractional distillation *in vacuo* (100 mbar, boiling point 78…80 °C) as yellow oil.

^{1}H-NMR (500 MHz, $CDCl_3$): δ (ppm) = 7.30 (t, *J* = 1.35 Hz, 1°H, C4-**H**), 4.04 (d, *J* = 1.38 Hz, 2 H, C5-**H_2**), 1.66 (s, 6 H, C2 (C**H_3**)**$_2$**).

^{13}C-NMR (126 MHz, $CDCl_3$): δ (ppm) = 156.9 (**C**4), 89.5 (**C**2-(CH_3)$_2$), 45.1 (**C**5), 32.8 (C2 (**C**H_3)$_2$).

HRMS (ESI) m/z calculated for $C_5H_{10}NS$ $[M+H]^+$: 116.05285, found: 116.0531.

IR (neat) / cm^{-1}: 2967, 2920, 1711, 1644, 1432, 1362, 1310, 1218, 931, 889, 802, 779, 681, 573, 545, 529.

2,2,5,5-Tetramethyl-3-thiazoline (1b)

2,2,5,5-Tetramethyl-3-thiazoline (**1b**) is prepared according to GP 2 using acetone (14.7 mL, 0.20 mol), ammonia-solution (13.3 M, 30.0 mL), sodiumhydrogensulfid-monohydrate (11.2 g, 0.20 mol) and 2-chloro-2-methylpropanal (21.3 g, 0.20 mol,), yielding **1b** (6.32 g, 0,04 mol, 22%) *via* fractional distillation *in vacuo* (13 mbar, boiling point 40…50 °C) in colorless crystals.

^{1}H-NMR (500 MHz, $CDCl_3$): δ (ppm) = 6.89 (s, 1 H, C4-**H**), 1.67 (s, 6 H, C2-(C**H_3**)**$_2$**), 1.55 (s, 6 H, C5-(C**H_3**)**$_2$**).

^{13}C-NMR (126 MHz, $CDCl_3$): δ (ppm) = 165.7 (**C4**), 89.5 (**C**2), 65.8 (**C**5), 33.9 (C2-(**C**H_3)$_2$), 30.1 (C5-(**C**H_3)$_2$).

MS (EI) m/z calculated for $C_7H_{13}NS$ $[M]^+$: 143.08, found: 143.10.

The analytical data corresponds with literature data.[8]

2,2,4,5,5-Pentamethyl-3-thiazoline (1d)

2,2,4,5,5-Pentamethyl-3-thiazoline (**1d**) is prepared according to GP 2 using acetone (22.1 mL, 300 mmol), ammonia-solution (13.3 M, 45.0 mL), sodiumhydrogensulfid-monohydrate (16.8 g, 300 mmol) and 3-chloro-3-methyl-2-butanone (36.2 g, 300 mmol), yielding **1d** (29.3 g, 186 mmol, 62%) *via* fractional distillation *in vacuo* (10 mbar, boiling point 50 °C) as yellow oil.

^{1}H-NMR (500 MHz, $CDCl_3$): δ (ppm) = 1.98 (s, 3 H, C4-C**H_3**), 1.59 (s, 6°H, C2-(C**H_3**)**$_2$**), 1.53 (s, 6 H, C5-(C**H_3**)**$_2$**).

^{13}C-NMR (126 MHz, $CDCl_3$): δ (ppm) = 172.3 (**C**4), 82.8 (**C**2), 66.8 (**C**5), 33.8 (C2-(**C**H_3)$_2$), 30.7 (C5-(**C**H_3)$_2$), 15.0 (C4-**C**H_3).

HRMS (ESI) m/z calculated for $C_8H_{16}NS$ $[M+H]^+$: 158.09980, found: 158.0995.

IR (neat) / cm^{-1}: 2966, 2924, 2857, 1656, 1456, 1438, 1363, 1258, 1204, 1169, 1145, 1098, 854, 640, 591, 574, 483.

3-Methyl-1-thia-4-azaspiro[4.4]non-3-ene (1e)

3-Methyl-1-thia-4-azaspiro[4.4]non-3-ene (**1e**) is prepared according to GP 2 using cyclopentanone (17.7 mL, 200 mmol), ammonia-solution (13.3 M, 30.0 mL), sodiumhydrogensulfid-monohydrate (11.2 g, 200 mmol,) and 3-chloroacetone (16.1 mL, 200 mmol), yielding **1e** (1.52 g, 9.66 mmol, 5%) *via* fractional distillation *in vacuo* and following column chromatography (6-50% EE in cyclohexane) as brown oil.

^{1}H-NMR (500 MHz, $CDCl_3$): δ (ppm) = 3.91 (s, 2 H, C2-**H_2**), 2.17–1.72 (m, 8°H, (C**H_2**)$_4$), 2.10 (s, 3 H, C3-C**H_3**).

^{13}C-NMR (126 MHz, $CDCl_3$): δ (ppm) = 165.9 (**C**3), 97.6 (**C**5), 46.3 (**C**2), 43.6, 24.6 (4x **C**H_2), 19.8 (C3-**C**H_3).

HRMS (ESI) m/z calculated for $C_8H_{14}NS$ $[M+H]^+$: 156.08415, found: 156.0844.

IR (neat) / cm^{-1}: 2956, 2869, 1742, 1664, 1435, 1372, 1268, 1196, 1152, 956, 896, 869, 775, 526, 471, 432.

2,2,3-Trimethyl-1-thia-4-azaspiro[4.4]non-3-ene (1f)

2,2,3-Trimethyl-1-thia-4-azaspiro[4.4]non-3-ene (**1f**) is prepared according to GP 2 using cyclopentanone (17.7 mL, 200 mmol), ammonia-solution (13.3 M, 30.0 mL), sodiumhydrogensulfid-monohydrate (11.2 g, 200 mmol,) and 3-chloro-3-methyl-2-butanone (24.1 g, 200 mmol), yielding **1f** (20.5 g, 112 mmol, 56%) *via* fractional distillation *in vacuo* (7.4 10^{-1} mbar, boiling point 80…88 °C) as yellow oil.

^{1}H-NMR (500 MHz, $CDCl_3$): δ (ppm) = 2.00 (s, 3 H, C3-(C**H_3**), 2.15–1.71 (m, 8°H, (C**H_2**)$_4$), 1.54 (s, 6 H, C5-(C**H_3**)$_2$).

^{13}C-NMR (126 MHz, $CDCl_3$): δ (ppm) = 172.4 (**C**3), 91.6 (**C**5), 65.5 (**C**2), 44.0, 30.2 (4x **C**H_2), 24.6 (C2-(**C**H_3)$_2$, 15.0 (C3-**C**H_3).

MS (ESI) m/z calculated for $C_{10}H_{18}NS$ $[M+H]^+$: 184.1, found: 184.1.

The analytical data corresponds with literature data.[7]

GP 3: Synthesis of *2H*-1,4-benzothiazines

The synthesis is conducted according to an adapted procedure from Stalling *et al*.[9]

Sodium (4.64 g, 200 mmol, 1.00 equ.) is dissolved in ethanol p.A. (400 mL) under cooling with ice. 2-aminothiophenole (21.4 mL, 200 mmol, 1.00 equ.) being dissolved in ethanol p.A. (40 mL) is added at room temperature. α-Chlorated aldehyde or ketone (200 mmol, 1.00 equ.) is added and the reaction mixture is stirred at room temperature for 2 h. Precipitated sodium chloride is filtered off and the solvent is evaporated *in vacuo*. The residue is dissolved in diethylether, the precipitated sodium chloride is filtered off again and the solvent evaporated *in vacuo*. The crude product is purified *via* fractional distillation.

2,2-Dimethyl-*2H*-1,4-benzothiazine (3a)

2,2-Dimethyl-*2H*-1,4-benzothiazine (**3a**) is prepared according to GP 3 using 2-aminothiophenole (21.4 mL, 200 mmol) and 2-chloro-2-methylpropanal (21.3 g, 200 mmol), yielding **3a** (6.42 g, 36.2 mmol, 18%) *via* fractional distillation *in vacuo* (10 mbar, boiling point 160 °C) as orange solid.

^{1}H-NMR (500 MHz, $CDCl_3$): δ (ppm) = 7.56 (s, 1 H, C3-**H**), 7.43 (dd, *J* = 1.35, 7.80 Hz, 1 H, Ar-**H**), 7.26–7.19 (m, 2 H, Ar-**H**), 7.14 (td, *J* = 1.49, 7.54 Hz 1 H, Ar-**H**) 1.39 (s, 6 H, 2 x C**H₃**).

^{13}C-NMR (126 MHz, $CDCl_3$): δ (ppm) = 160.7 (**C**3), 141.0 (Ar-**C**), 127.8 (Ar-**C**), 127.7 (Ar-**C**), 127.5 (Ar-**C**), 126.4 (Ar-**C**), 123.4 (Ar-**C**), 37.2 (**C**2), 25.4 (2 x **C**H_3).

MS (ESI) m/z calculated for $C_{10}H_{11}NS$ $[M+H]^+$: 178.06, found: 177.97

The analytical data corresponds with literature data.[9]

2,2,3-Trimethyl-*2H*-1,4-benzothiazine (3b)

2,2,3-Trimethyl-*2H*-1,4-benzothiazine (**3b**) is prepared according to GP 3 using 2-aminothiophenole (21.4 mL, 200 mmol) and 3-chloro-3-methyl-2-butanone (24.1 g, 200 mmol), yielding **3b** (27.4 g, 143 mmol, 71%) *via* fractional distillation *in vacuo* (40 mbar boling point 170 °C) as green oil.

^{1}**H-NMR** (500 MHz, $CDCl_3$): δ (ppm) = 7.35 (dd, *J* = 1.39, 7.83 Hz, 1 H, Ar-**H**), 7.22 (dd, *J* = 1.49, 7.65 Hz, 1 H, Ar-**H**), 7.17 (td, *J* = 1.49, 7.63, 7.66 Hz, 1 H, Ar-**H**), 7.07 (td, *J* = 1.39, 7.45, 7.52 Hz, 1 H, Ar-**H**), 2.30 (s, 3 H, C3-C**H₃**),1.37 (s, 6 H, 2 x C**H₃**).

^{13}**C-NMR** (126 MHz, $CDCl_3$): δ (ppm) = 167.5 (**C**3), 141.7 (Ar-**C**), 127.1 (Ar-**C**), 126.9 (Ar-**C**), 126.4 (Ar-**C**), 126.2 (Ar-**C**), 123.7 (Ar-**C**), 39.6 (**C**2), 24.8 (2 x **C**H_3), 23.0 (C3-**C**H_3).

HRMS (ESI) m/z calculated for $C_{11}H_{14}NS$ $[M+H]^+$: 192.08415, found: 192.0845.

IR (neat) / cm^{-1}: 2956, 1616, 1587, 1476, 1455, 1440, 1420, 1386, 1364, 1305, 1245, 1173, 1118, 1071, 1030, 853, 750, 725, 690, 649, 453, 436.

2,2-Dimethyl-3-phenyl-*2H*-1,4-benzothiazine (3c)

2,2-dimethyl-3-phenyl-*2H*-1,4-benzothiazine (**3c**) is prepared according to GP 3 using 2-aminothiophenole (10.7 mL, 100 mmol) and 2-chloro-2-methyl-1-phenyl-1-propanone (18.3 g, 100 mmol), yielding **3c** (18.7 g, 73.7 mmol, 74%) *via* column chromatography (1-14% ethylacetate in cyclohexane) as white-yellow solid.

^{1}**H-NMR** (500 MHz, $CDCl_3$): δ (ppm) = 7.55 (dd, *J* = 1.41, 7.79 Hz, 1 H, Ar-**H**), 7.53–7.50 (m, 2 H, Ar-**H**), 7.46–7.44 (m, 3 H, Ar-**H**), 7.36 (dd, *J* = 1.45, 7.70 Hz, 1 H, Ar-**H**), 7.29–7.27 (m, 1 H, Ar-**H**), 7.19 (td, *J* = 1.37, 7.44, 7.51 Hz 1 H, Ar-**H**), 1.50 (s, 6 H, 2 x C**H₃**).

^{13}**C-NMR** (126 MHz, $CDCl_3$): δ (ppm) = 167.8 (**C**3), 142.1 (Ar-**C**), 138.8 (Ar-**C**), 129.1 (Ar-**C**), 128.3 (Ar-**C**), 127.9 (Ar-**C**), 127.8 (Ar-**C**), 127.2 (Ar-**C**), 127.0 (Ar-**C**), 126.5 (Ar-**C**), 124.0 (Ar-**C**), 39.6 (**C**2), 26.3 (2 x **C**H_3).

HRMS (ESI) m/z calculated for $C_{16}H_{16}NS$ $[M]^+$: 254.09980, found: 254.1001.

IR (neat) / cm^{-1}: 1455, 1440, 1368, 1365, 1305, 1293, 1170, 1119, 1075, 998, 978, 968, 763, 740, 703, 689, 669, 631, 561, 453.

Synthesis of reference compounds

GP4: Synthesis of 3-thiazolidines

The synthesis is conducted according to an adapted procedure from Reiners *et al.*[7]

3-Thiazoline (14.4 mmol, 1.00 Äqu.) is dissolved in toluene (40 mL). Catecholborane (52.1 mmol, 3.00 equ.) is dissolved in toluene (10 mL) and slowly added at room temperature. The reaction mixture

is stirred for 48 h, dH_2O is carefully added and the mixture is extracted with sodiumhydroxide solution (2 M, 3x). The combined organic phases are dried over magnesium sulfate, the solvent is evaporated *in vacuo* and the crude product is purified *via* column chromatography.

2,2,5,5-Tetramethyl-3-thiazolidine (2b)

2,2,5,5-Tetramethyl-3-thiazolidine (**2b**) is prepared according to GP 4 using 2,2,5,5-tetramethyl-3-thiazoline (1.16 g, 8.09 mmol) and catecholborane (2.60 mL, 24.3 mmol), yielding **2b** (186 mg, 1.28 mmol, 16%) *via* column chromatography (12%-100% ethylacetate in cyclohexane) as colorless oil.

^{1}H-NMR (500 MHz, $CDCl_3$): δ (ppm) = 3.02 (s, 2°H, C4-$\mathbf{H_2}$), 1.59 (s, 6 H, C2-(C$\mathbf{H_3}$)$_2$), 1.43 (s, 6 H, C5-(C$\mathbf{H_3}$)$_2$).
^{13}C-NMR (126 MHz, $CDCl_3$): δ (ppm) = 64.6 (**C**2, **C**5), 59.8 (**C**4), 32.6 (C2-(**C**H_3)$_2$), 30.4 (C2-(**C**H_3)$_2$).
HRMS (ESI) m/z calculated for $C_7H_{16}NS$ $[M+H]^+$: 146.09980, found: 146.1000.

rac-2,2,4-Trimethyl-3-thiazolidine (2c)

rac-2,2,4-Trimethyl-3-thiazolidine (**2c**) is prepared according to GP 4 using 2,2,4-Trimethyl-3-thiazoline (1.00 g, 7.74 mmol) and catecholborane (2.48 mL, 23.2 mmol), yielding **2c** (29.9 mg, 0.23 mmol, 3%) *via* column chromatography (12%-100% ethylacetate in cyclohexane) as colorless oil.

^{1}H-NMR (500 MHz, $CDCl_3$): δ (ppm) = 3.57–3.50 (m, 1°H, C4-**H**), 3.22 (dd, *J* = 5.61, 10.26 Hz, 1 H, C5-**H**), 2.59 (dd, *J* = 5.55, 9.99 Hz, 1 H, C5-**H**), 1.67 (s, 3 H, C2-C$\mathbf{H_3}$), 1.55 (s, 3 H, C2-C$\mathbf{H_3}$), 1.35 (d, *J* = 6.31 Hz, 3°H, (C4-C$\mathbf{H_3}$).
^{13}C-NMR (126 MHz, $CDCl_3$): δ (ppm) = 75.8 (**C**2), 59.7 (**C**4), 44.7 (**C**5), 33.2 (C2-**C**H_3), 31.7 (C2-**C**H_3), 19.4 (C4-**C**H_3).
HRMS (ESI) m/z calculated for $C_6H_{14}NS$ $[M+H]^+$: 132.08415, found: 132.0844.

rac-2,2,4,5,5-Pentamethyl-3-thiazolidine (2d)

rac-2,2,4,5,5-Pentamethyl-3-thiazolidine (**2d**) is prepared according to GP 4 using 2,2,4,5,5-pentamethyl-3-thiazoline (2.73 g, 17.4 mmol) and catecholborane (5.55 mL, 52.1 mmol), yielding **2d**

(860 mg, 5.40 mmol, 31%) *via* column chromatography (12%-100% ethylacetate in cyclohexane) as colorless oil.

^{1}H-NMR (500 MHz, $CDCl_3$): δ (ppm) = 3.27 (q, *J* = 6.60 Hz, 1°H, C4-**H**), 1.60 (s, 3 H, C2-C**H₃**), 1.55 (s, 3 H, C2-C**H₃**), 1.41 (s, 3 H, C5-C**H₃**), 1.19 (s, 3 H, C5-C**H₃**)1.11 (d, *J* = 6.59 Hz, 3°H, (C4-C**H₃**).

^{13}C-NMR (126 MHz, $CDCl_3$): δ (ppm) = 72.0 (**C**2), 66.6 (**C**4), 61.4 (**C**5), 33.9 (C2-**C**H_3), 33.8 (C2-**C**H_3), 27.7 (C5-**C**H_3), 26.1 (C5-**C**H_3), 13.6 (C4-**C**H_3).

HRMS (ESI) m/z calculated for $C_8H_{18}NS$ $[M+H]^+$: 160.11545, found: 160.1150.

IR (neat) / cm^{-1}: 2964, 2922, 2851, 2357, 1736, 1454, 1378, 1365, 1201, 1158, 1119, 1043, 853, 797, 750, 632, 551.

rac-3-Methyl-1-thia-4-azaspiro[4.4]nonane (2e)

rac-3-Methyl-1-thia-4-azaspiro[4.4]nonane (**2e**) is prepared according to GP 4 using 3-methyl-1-thia-4-azaspiro[4.4]non-3-ene (735 g, 4.73 mmol) and catecholborane (1.50 mL, 14.2 mmol), yielding **2e** (58.9 mg, 0.38 mmol, 8%) *via* column chromatography (6%-50% ethylacetate in cyclohexane) as brown oil.

^{1}H-NMR (500 MHz, $CDCl_3$): δ (ppm) = 3.39–3.33 (m, 1°H, C3-**H**), 3.17 (dd, *J* = 5.76, 10.12 Hz, 1 H, C2-**H**), 2.55 (t, *J* = 9.88 Hz, 1 H, C2-**H**), 2.19–1.67 (m, 8 H, C**H_2**)**$_4$**), 1.35 (d, *J* = 6.27 Hz 3 H, C3-C**H_3**).

^{13}C-NMR (126 MHz, $CDCl_3$): δ (ppm) = 85.3 (**C**5), 59.9 (**C**3), 44.2 (**C**2), 43.6, 41.1, 24.7, 24.2 (**C**H_2)**$_4$**), 19.1 (C3-**C**H_3).

HRMS (ESI) m/z calculated for $C_8H_{16}NS$ $[M+H]^+$: 158.09980, found: 158.0997.

rac-2,2,3-Trimethyl-1-thia-4-azaspiro[4.4]nonane (2f)

2,2,3-Trimethyl-1-thia-4-azaspiro[4.4]nonane (**2f**) is prepared according to GP 4 using 2,2,3-Trimethyl-1-thia-4-azaspiro[4.4]non-3-ene (2.16 g, 11.81 mmol) and catecholborane (3.78 mL, 35.4 mmol), yielding **2f** (933 mg, 5.03 mmol, 42%) *via* column chromatography (6%-50% ethylacetate in cyclohexane) as colorless oil.

^{1}**H-NMR** (500 MHz, $CDCl_3$): δ (ppm) = 3.11 (q, *J* = 6.60 Hz, 1°H, C3-**H**), 2.13–1.63 (m, 8 H, (C**H**$_2$)$_4$), 1.41 (s, 3 H, C2-C**H**$_3$), 1.19 (s, 3 H, C2-C**H**$_3$), 1.10 (d, *J* = 6.59 Hz 3 H, C3-C**H**$_3$).

^{13}**C-NMR** (126 MHz, $CDCl_3$): δ (ppm) = 81.5 (**C**5), 67.1 (**C**3), 59.9 (**C**2), 44.5, 42.4, 24.4, 23.9 (**C**H_2)$_4$), 27.9 (C2-**C**H_3), 26.1 (C2-**C**H_3), 13.7 (C3-**C**H_3).

MS (ESI) m/z calculated for $C_{10}H_{20}NS$ $[M+H]^+$: 186.12, found: 186.1.

The analytical data corresponds with literature data.[7]

GP 5: Derivatization of 3-thiazolidines with phenylisocyanate

The synthesis is conducted according to Reiners *et al.*[7]

3-Thiazolidine (0.36 mmol, 1.00 equ.) is dissolved in diethylether (0.5 mL). Phenylisocyanate (0.38 mmol, 1.05 Äqu.) and cyclohexane (0.25 mL) are added and the reaction mixture is stirred over night and the solvent is evaporated meanwhile. The white solid is dried *in vacuo*.

rac-2,2,4-Trimethyl-*N*-phenylthiazolidine-3-carboxamide (5c)

rac-2,2,4-Trimethyl-*N*-phenylthiazolidine-3-carboxamide (**5c**) is prepared according to GP 5 using rac-2,2,4-trimethyl-3-thiazolidine (**2c**) (32.5 mg, 0.25 mmol) and phenylisocyanate (28.0 µL, 0.25 mmol), yielding **5c** (49.4 mg, 0.19 mmol, 79%) as white solid.

^{1}**H-NMR** (500 MHz, $CDCl_3$): δ (ppm) = 7.35–7.26 (m, 4 H, Ar-**H**), 7.05–7.02 (m, 1 H, Ar-**H**), 6.23 (s, 1 H, N**H**), 4.40–4.35 (m, 1°H, C4-**H**), 3.33 (d, *J* = 5.86, 11.68 Hz,1 H, C5-**H**), 2.61 (d, *J* = 11.67 Hz, 1 H, C5-**H**), 1.90 (s, 3 H, C2-C**H**$_3$), 1.87 (s, 3 H, C2-C**H**$_3$) 1.50 (d, *J* = 6.25 Hz, 3 H, C4-C**H**$_3$).

^{13}**C-NMR** (126 MHz, $CDCl_3$): δ (ppm) = 152.1 (**C**=O), 138.9, 129.0, 123.4, 120.4 (Ar-**C**), 72.4 (**C**2), 58.7 (**C**4), 34.9 (**C**5), 30.2 (C2-**C**H_3), 29.9 (C2-**C**H_3), 20.8 (C4-**C**H_3).

HRMS (ESI) m/z calculated for $C_{13}H_{19}N_2OS$ $[M+H]^+$: 251.12126, found: 251.1209.

rac-2,2,4,5,5-Pentamethyl-*N*-phenylthiazolidin-3-carboxamide (5d)

rac-2,2,4,5,5-Pentamethyl-*N*-phenylthiazolidin-3-carboxamide (**5d**) is prepared according to GP 5 using rac-2,2,4,5,5-pentamethyl-3-thiazolidine (**2d**) (57.0 mg, 0.36 mmol) and phenylisocyanate (41.0 µL, 0.38 mmol), yielding **5d** (91.8 mg, 0.33 mmol, 92%) as white solid.

^{1}**H-NMR** (500 MHz, $CDCl_3$): δ (ppm) = 7.38–7.35 (m, 2 H, Ar-**H**), 7.32–7.28 (m, 2 H, Ar-**H**), 7.06–7.03 (m, 1 H, Ar-**H**), 3.90 (q, *J* = 6.52 Hz, 1°H, C4-**H**), 2.02 (s, 3 H, C2-C**H₃**), 1.86 (s, 3 H, C2-C**H₃**), 1.66 (s, 3 H, C5-C**H₃**), 1.46 (d, *J* = 6.51 Hz, 3°H, (C4-C**H₃**), 1.32 (s, 3 H, C5-C**H₃**).

^{13}**C-NMR** (126 MHz, $CDCl_3$): δ (ppm) = 152.8 (**C**=O), 138.9, 129.1, 123.4, 120.4 (Ar-**C**), 71.9 (**C**2), 68.7 (**C**4), 51.3 (**C**5), 33.3 (C2-**C**H_3), 33.1 (C2-**C**H_3), 31.7 (C5-**C**H_3), 24.2 (C5-**C**H_3), 17.8 (C4-**C**H_3).

HRMS (ESI) m/z calculated for $C_{15}H_{23}N_2OS$ $[M+H]^+$: 279.15256, found: 279.1531.

IR (neat) / cm^{-1}: 2364, 1631, 1529, 1441, 745.

rac-3-Methyl-*N*-phenyl-1-thia-4-azaspiro[4.4]nonan-4-carboxamide (5e)

rac-3-Methyl-*N*-phenyl-1-thia-4-azaspiro[4.4]nonan-4-carboxamide (**5e**) is prepared according to GP 5 using rac-3-methyl-1-thia-4-azaspiro[4.4]nonan (**2e**) (2.51 mg, 0.02 mmol) and phenylisocyanate (1.90 µL, 0.02 mmol), yielding **5e** (8.46 mg, 0.02 mmol, 100%) as white solid.

^{1}**H-NMR** (500 MHz, $CDCl_3$): δ (ppm) = 7.37–7.27 (m, 4 H, Ar-**H**), 7.07–7.02 (m, 1 H, Ar-**H**), 4.39–4.34 (m, 1°H, C3-**H**), 3.27 (dd, *J* = 5.95, 11.74 Hz, 1 H, C2-**H**), 2.85 (dd, *J* = 5.18, 9.36 Hz, 1 H, C2-**H**), 2.74–1.61 (m, 8 H, C**H₂**)**₄**), 1.53 (d, *J* = 6.25 Hz 3 H, C3-C**H₃**).

^{13}**C-NMR** (126 MHz, $CDCl_3$): δ (ppm) = 151.8 (**C**=O), 138.9, 129.1, 123.4, 120.4 (Ar-**C**), 81.5 (**C**5), 57.9 (**C**3), 40.1 (**C**2), 38.4, 35.6, 25.4, 25.1 (**C**H_2)**₄**), 20.7 (C3-**C**H_3).

HRMS (ESI) m/z calculated for $C_{15}H_{21}N_2OS$ $[M+H]^+$: 277.13691, found: 277.1374.

rac-2,2,3-Trimethyl-*N*-phenyl-1-thia-4-azaspiro[4.4]nonan-4-carboxamide (5f)

rac-2,2,3-Trimethyl-*N*-phenyl-1-thia-4-azaspiro[4.4]nonan-4-carboxamide (**5f**) is prepared according to GP 5 using rac-2,2,3-trimethyl-1-thia-4-azaspiro[4.4]nonane (**2f**) (54.1 mg, 0.29 mmol) and phenylisocyanate (33.5 µL, 0.31 mmol), yielding **5f** (82.3 mg, 0.27 mmol, 93%) as white solid.

^{1}**H-NMR** (500 MHz, $CDCl_3$): δ (ppm) = 7.38–7.33 (m, 2 H, Ar-**H**), 7.32–7.27 (m, 2 H, Ar-**H**), 7.06–7.03 (m, 1 H, Ar-**H**), 3.88 (q, *J* = 6.40 Hz, 1°H, C3-**H**), 3.03–1.63 (m, 8 H, (C**H₂**)**₄**), 1.63 (s, 3 H, C2-C**H₃**), 1.44 (d, *J* = 6.35 Hz 3 H, C3-C**H₃**), 1.32 (s, 3 H, C2-C**H₃**).

^{13}C-NMR (126 MHz, $CDCl_3$): δ (ppm) = 152.4 (**C**=O), 138.9, 129.1, 123.4, 120.4 (Ar-**C**), 80.8 (**C**5), 68.0 (**C**3), 51.9 (**C**2), 41.7, 41.1, 32.1, 23.7 ($\mathbf{C}H_2)_4$), 25.1 (C2-$\mathbf{C}H_3$), 25.1 (C2-$\mathbf{C}H_3$), 17.5 (C3-$\mathbf{C}H_3$).

HRMS (ESI) m/z calculated for $C_{17}H_{25}N_2OS$ $[M+H]^+$: 305.16821, found: 305.1687.

IR (neat) / cm^{-1}: 2357, 2012, 1632, 1594, 1548, 1501, 1440, 1346, 754.

GP 6: Synthesis of 3,4-dihydro-*2H*-1,4-benzothiazines

The synthesis is conducted according to Rueping *et al.*[10]

2H-1,4-benzothiazine (0.78 mmol, 1.00 equ.) is dissolved in dichloromethane (5 mL). Hantzsch ester (1.18 mmol, 1.40 equ.) and diphenylphosphate (0.04 mmol, 5 mol%) are added and the reaction mixture is stirred for 24 h under argon atmosphere at 40 °C. The solvent is evaporated *in vacuo* and the crude product is purified *via* column chromatography.

2,2-Dimethyl-3,4-dihydro-*2H*-1,4-benzothiazine (4a)

2,2-Dimethyl-3,4-dihydro-*2H*-1,4-benzothiazine (**4a**) is prepared according to GP 6 using 2,2-dimethyl-*2H*-1,4-benzothiazine (107 mg, 0.60 mmol), Hantzsch ester (213 mg, 0.84 mmol) and diphenylphosphate (6.94 mg, 0.03 mmol,), yielding **4a** (90.1 mg, 0.29 mmol, 49%) *via* column chromatography (9% ethylacetate in cyclohexane) as white solid.

^{1}H-NMR (500 MHz, $CDCl_3$): δ (ppm) = 6.97 (dd, *J* = 1.47, 7.71 Hz, 1 H, Ar-**H**), 6.92 (ddd, *J* = 1.49, 7.23, 7.93 Hz, 1°H, Ar-**H**), 6.65 (ddd, *J* = 1.27, 7.51, 7.56 Hz, 1 H, Ar-**H**), 6.53 (dd, *J* = 1.26, 8.02 Hz, 1 H, 1 H, Ar-**H**), 3.25 (, 2 H, $C\mathbf{H_2}$), 1.43 (s, 6 H, C2-($C\mathbf{H_3})_2$).

^{13}C-NMR (500 MHz, $CDCl_3$): δ (ppm) = 140.2 (Ar-**C**), 127.7 (Ar-**C**), 125.3 (Ar-**C**), 118.2 (Ar-**C**), 116.6 (Ar-**C**), 114.9 (Ar-**C**), 54.6 (**C**3), 39.7 (**C**2), 28.0 (C2-($\mathbf{C}H_3)_2$).

MS (EI) m/z calculated for $C_{10}H_{13}NS$ $[M]^+$: 179.08, found: 179.1.

The analytical data corresponds with literature data.[11]

2,2,3-Trimethyl-3,4-dihydro-*2H*-1,4-benzothiazine (4b)

2,2,3-Trimethyl-3,4-dihydro-*2H*-1,4-benzothiazine (**4b**) is prepared according to GP 6 using 2,2,3-trimethyl-*2H*-1,4-benzothiazine (150 mg, 0.78 mmol), Hantzsch ester (298 mg, 1.18 mmol) and

diphenylphosphate (9.75 mg, 0.04 mmol), yielding **4b** (51.4 mg, 0.27 mmol, 34%) *via* column chromatography (2%-14% ethylacetate in cyclohexane) as brown oil.

^{1}H-NMR (500 MHz, $CDCl_3$): δ (ppm) = 6.95 (dd, *J* = 1.45, 7.67 Hz, 1 H, Ar-**H**), 6.89 (ddd, *J* = 1.47, 7.33, 8.26 Hz, 1 H, Ar-**H**), 6.61 (td, *J* = 1.23, 7.48, 7.50 Hz, 1 H, Ar-**H**), 6.48 (dd, *J* = 1.26, 7.89 Hz 1 H, Ar-**H**),3.85 (s, 1 H, N**H**), 3.50 (q, 1 H, *J* = 6.43, 6.45, 6.45 Hz, C3-**H**), 1.34 (s, 3 H, C2-C**H$_3$**),1.30 (s, 3 H, C2-C**H$_3$**), 1.18 (d, *J* = 6.44, 3 H, C3-C**H$_3$**).

^{13}C-NMR (126 MHz, $CDCl_3$): δ (ppm) = 140.6 (Ar-**C**), 127.5 (Ar-**C**), 125.3 (Ar-**C**), 117.9 (Ar-**C**), 116.6 (Ar-**C**), 114.6 (Ar-**C**), 56.2 (**C**3), 42.6 (**C**2), 27.4 (C2-**C**H_3), 23.7 (C2-**C**H_3), 18.0 (C3-**C**H_3)-

HRMS (ESI) m/z calculated for $C_{11}H_{16}NS$ $[M+H]^+$: 194.09980, found: 194.1001.

IR (neat) / cm^{-1}: 2961, 1590, 1480, 1307, 1123, 738.

2,2-Dimethyl-3-phenyl-3,4-dihydro-*2H*-1,4-benzothiazine (4c)

2,2-Dimethyl-3-phenyl-3,4-dihydro-*2H*-1,4-benzothiazine (**4c**) is prepared according to GP 6 using 2,2,3-trimethyl-*2H*-1,4-benzothiazine (450 mg, 1.78 mmol), Hantzsch ester (210 mg, 0.83 mmol) and diphenylphosphate (7.40 mg, 0.03 mmol), yielding **4b** (295 mg, 1.16 mmol, 65%) *via* column chromatography (100% cyclohexane) as white solid.

^{1}H-NMR (500 MHz, $CDCl_3$): δ (ppm) = 7.39–7.34 (m, 5 H, Ar-**H**), 7.02 (dd, *J* = 1.45, 7.75 Hz, 1 H, Ar-**H**), 6.96–6.92 (m, 1 H, Ar-**H**), 6.67 (td, *J* = 1.27, 7.51, 7.53 Hz, 1 H, Ar-**H**), 6.54 (dd, *J* = 1.23, 8.00 Hz 1 H, Ar-**H**), 4.59 (s, 1 H, C3-**H**), 4.23 (s, 1 H, N**H**), 1.32 (s, 3 H, C2-C**H$_3$**), 1.26 (s, 3 H, C2-C**H$_3$**).

^{13}C-NMR (126 MHz, $CDCl_3$): δ (ppm) = 141.1(Ar-**C**), 140.5 (Ar-**C**), 128.7 (Ar-**C**), 128.3 (Ar-**C**), 127.5 (Ar-**C**), 125.6 (Ar-**C**), 118.3 (Ar-**C**), 116.4 (Ar-**C**), 114.7 (Ar-**C**), 65.8 (**C**3), 42.8 (**C**2), 27.8 (C2-**C**H_3), 24.5 (C2-**C**H_3).

MS (EI) m/z calculated for $C_{16}H_{18}NS$ $[M+H]^+$: 256.11, found: 256.1.

The analytical data corresponds with literature data.[7]

GC analytics

Conversions for biotransformations of 3-thiazolines **1b-f** to the corresponding 3-thiazolidines **2b-f** were determined by analyzing the organic phase directly after extraction. Analysis was carried out using the gaschromatograph system GC-2010 Plus equipped with ZB-5MSi column (Phenomenex, 30 m x 0.25 mm x 0.25 µm; N_2; linear velocity 46.9 cm s^{-1} split mode 1:10; total flow 28.8 mL min^{-1}; purge flow 3.0 mL min^{-1}; column flow 2.34 mL min^{-1};pressure 140.4 kPa) and coupled to an AOC-20i/s auto injector/auto sampler. Relative conversions of substrates were determined based on area% of remaining substrate and product. Temperature programs and retention times are given in Supplementary Table 5, GC chromatograms are shown in Supplementary Figure 56-60.

Chiral HPLC analytics for derivatized 3-thiazolines

For analysis of the enantiomeric excess of 3-thiazolidines **2b**-**2f**, samples were derivatized according to GP5. The corresponding solids were dissolved in dichloromethane after derivatization and analyzed by means of a LC2000 SFC-HPLC system from Jasco (Easton, USA) with HPLC column Chiralpak IC from Daicel (supercritical CO_2:EtOH (Et_2NH) = 90:10 (0.01), 1 mL min^{-1}, 20 °C, 10 MPa, 210 nm). Enantiomeric excess was determined based on area% of the enantiomers. Retention Times are given in Supplementary Table 6, HPLC chromatograms are shown in Supplementary Figures 79-82.

Chiral HPLC analytics for *2H*-1,4-benzothiazines/3,4-dihydro-*2H*-1,4-benzothiazines

For the analysis of the biotransformations of 2*H*-1,4-benzothiazines a combined approach for the determination of the conversions and the enantiomeric excess was used. HPLC measurements were performed by analyzing the organic phase directly after extraction. Analysis was carried out by LC2000 SFC-HPLC system from Jasco (Easton, USA) with HPLC column Chiralpak IC or OB-H from Daicel at 20 °C with supercriticical CO2 / ethanol (0.1% Et2NH) as mobile phase, flow rate of 1 mL min^{-1} and 12 Mpa backpressure. Peaks are detected at 210 nm wavelength. Relative conversions of substrates **3a**-**c** to products **4a**-**c** were determined based on area% of remaining substrate and product. Enantiomeric excess was determined based on area% of the enantiomers. Details on HPLC methods and retention times are given in Supplementary Table 7. HPLC chromatograms are shown Supplementary Figures 93-95.

Colorimetric pH shift assay

The colorimetric pH shift assay is conducted according to Pick *et al*.[12]

This assay is an indirect screening method, based on a color change of bromthymolblue depending on the pH. A decrease of pH under 5 leads to a color change from blue/green to yellow. The formation of gluconic acid due to the consumption of the substrate and the regeneration of NADPH decreases the pH, resulting in the color change (Supplementary Figure 3).

The screening of IREDs with 3-thiazolines and *2H*-1,4-benzothiazines is performed doublefold for each imine reductase in 96 well microtiterplates in 10 mM KPi pH 7.4 (+ 0.01 % BTB), containing 20 mM D-glucose, 10 mM substrate (100 mM stock in methanol or dimethylsulfoxide), 10 µL GDH, 10 or 40 µL IRED (10 µL for screening of 2*H*-1,4-benzothiazines and 40 µL for 3-thiazoline screening) and 0.1 mM NADPH. Moreover 2 different negative controls were performed in doublefold. One negative control comprising everything except IRED crude extract (named negative) and one with crude extract of an empty pET-22b(+) vector (named pET22b_empty) were also performed in doublefold.

Both negative controls showed no color change, indicating that tested 3-thiazolines **1a-f** and *2H*-1,4-benzothiazines **3a-c** are not converted by GDH, which is used for cofactor-regeneration. Recently Roth *et al*. showed that GDH shows side-activity towards imine reduction in some cases.[13] For our tested substrates we could not detect side-activity by GDH. Results of the colorimetric pH shift assay are shown exemplary as pictures for 3-thiazoline **1f** in Supplementary Figure 4. Results of the colorimetric pH shift assay for all substrates are summarized in Supplementary Table 2.

Spectrophotometric activity assay

For the determination of the specific activity, a spectrophotometric activity study was performed, measuring the consumption of NADPH spectrophotometrically at 340 nm for 60 seconds at the Tecan Reader Spark 10M (Tecan Trading AG, Switzerland) in 96 well microtiterplates. For this assay, 3-thiazolines **1a-f** (1.0 mM) and 2H-1,4-benzothiazines **3a-c** (0.5 mM) with 4% MeOH as a co-solvent in KPi buffer (100 mM, pH 7) and a final concentration of 0.25 mM NADPH was used. Imine reductases (10 – 60 µL, depending on substrate) were used as crude extract. Amount of total protein was determined in advance by the Bradford assay against BSA as a concentration standard.

The activities were measured at least four times and calculated according to Supplementary equation 1. Specific activity was calculated according to Supplementary equation 2.

$$\text{activity / U mL}^{-1} = \frac{\Delta E}{\Delta t}\frac{V_T}{\varepsilon \cdot V_E \cdot d} \cdot f \quad (1)$$

(V_T = total volume (here 0.25 mL); ε = extinction coefficient (here: 0.63 mL μmol^{-1} mm^{-1}); V_E = volume of enzyme solution; d = thickness of cuvette (here: 8 mm); f = dilution factor)

$$\text{specific activity / U mg}^{-1} = \frac{\text{activity / U mL}^{-1}}{\text{total protein concentration / mg mL}^{-1}} \quad (2)$$

The results for the spectrophotometric activity assay are shown in Supplementary Table 3 and in Supplementary Figure 6 for 3-thiazolines **1a-f** and in Supplementary Figure 7 for 2*H*-1,4-benzothiazines **3a-c**.

Details for DFT calculations

The DFT calculations were performed with the Gaussian09 suite of programs (Gaussian 09, Revision D.01, Frisch, M. J. *et al.* Gaussian, Inc., Wallingford CT, USA (2009)), using the B3LYP[14,15] density functional with the 6-311+G** basis. Coordinates for the initial complexes of the starting materials and the transition states are given below.

Benzothiazin: complex of starting materials

Charge = +1, Multiplicity = singlet

C	3.06526000	0.79921200	-0.56710300
C	1.99697000	0.83478900	0.34637400
C	1.63824500	-1.54071200	0.37243000
C	3.02698100	-1.91323600	-0.05452800
H	0.90276200	-2.31946300	0.56014900
N	1.24654000	-0.33253700	0.60253200
S	3.49569200	-0.70630500	-1.39050400
C	3.98253100	-1.83497700	1.15101300

H	3.67231900	-2.54271700	1.92603500
H	4.98971200	-2.10189000	0.82498600
H	4.01419700	-0.83610100	1.58806100
C	3.04308500	-3.31202200	-0.68527900
H	4.04909900	-3.56037800	-1.02752800
H	2.75173400	-4.05812900	0.05906700
H	2.36296600	-3.38238800	-1.53639700
C	1.60359400	2.03184600	0.95502600
C	2.27727600	3.20495400	0.64938700
C	3.32808200	3.18343600	-0.27212600
C	3.72198800	1.99325400	-0.87553600
H	0.78143400	2.02270500	1.66108300
H	1.98631900	4.13392000	1.12319700
H	3.85263200	4.09985800	-0.51465800
H	4.54667200	1.98435000	-1.57795400
H	0.25917800	-0.20381700	0.98566500
C	-4.36459400	-0.29279800	0.33648100
C	-3.04232600	0.03296400	0.44020800
C	-2.24734900	0.46107100	-0.78370700
C	-3.16885200	0.70257400	-1.95436700
C	-4.46433700	0.38649600	-1.93569200
H	-4.91404000	-0.69667000	1.17880000
H	-1.67326400	1.37053900	-0.56605700

H	-2.75756700	1.14445000	-2.85364900
H	-5.11941200	0.54875100	-2.78136600
N	-5.08754900	-0.16823500	-0.80216100
C	-6.46785600	-0.63636700	-0.90389100
H	-7.10522500	0.15462000	-1.30554400
H	-6.53773500	-1.51359300	-1.55342800
H	-6.83354400	-0.90539800	0.08629900
H	-1.49631400	-0.29811100	-1.05190500
C	-2.33627400	-0.08576300	1.70404900
O	-1.08144400	-0.10354100	1.76372400
N	-3.03064200	-0.19668300	2.87156000
H	-2.48568500	-0.24676000	3.71892800
H	-3.97873500	0.12929300	2.95452000

Benzothiazin: transition state

Charge = +1, Multiplicity = singlet

C	2.84474400	-0.42754500	-0.50660700
C	2.46276400	0.79910000	0.06463900
C	0.44584500	-0.08424800	1.16934200
C	0.99428600	-1.49838400	1.26734600
H	-0.25537400	0.20715000	1.95172100
N	1.29815400	0.91692000	0.83942100
S	1.82844200	-1.88110600	-0.35220900
C	1.98866600	-1.58883500	2.43868000

H	1.48831700	-1.33817000	3.37959300
H	2.37065800	-2.60882800	2.51359900
H	2.83503000	-0.91361100	2.31054700
C	-0.13733200	-2.51632800	1.44484500
H	0.26967000	-3.52744800	1.50649400
H	-0.67094100	-2.31690700	2.37883100
H	-0.85205200	-2.48678700	0.62080900
C	3.25375500	1.93861000	-0.13360200
C	4.40606200	1.86573700	-0.90258100
C	4.78666500	0.64989700	-1.47368200
C	4.01428800	-0.48867700	-1.26825700
H	2.95692300	2.87669700	0.32368600
H	5.01017300	2.75229500	-1.05007700
H	5.68976300	0.58483300	-2.06799600
H	4.31744000	-1.43649600	-1.69764300
H	0.93242300	1.85259400	0.99138800
C	-3.72436200	0.39540300	-0.10157500
C	-2.48891500	0.97620200	-0.17452700
C	-1.37326100	0.24674700	-0.78199800
C	-1.77849200	-0.92648300	-1.54313500
C	-3.03174300	-1.42579000	-1.43997100
H	-4.54785300	0.86410800	0.42194200
H	-0.60402500	0.87276600	-1.23791000

H	-1.06109500	-1.42751900	-2.17905500
H	-3.34913400	-2.31627100	-1.96662900
N	-4.00389400	-0.80620300	-0.67957000
C	-5.33026400	-1.42770200	-0.55420800
H	-5.72880600	-1.65513700	-1.54414300
H	-5.26543000	-2.34682100	0.03127100
H	-6.00521200	-0.73650800	-0.05337700
H	-0.55205600	-0.13601700	0.18193300
C	-2.19644300	2.29585700	0.45441100
O	-1.06804000	2.54222800	0.87737600
N	-3.21917300	3.18568900	0.56808300
H	-3.00003700	4.08404100	0.97515300
H	-4.03232800	3.13570900	-0.02406600

3-Thiazolin: complex of starting materials

Charge = +1, Multiplicity = singlet

C	-2.63997900	0.99546800	0.70885600
C	-3.41664800	-1.24210600	-0.66712900
N	-1.65393200	0.29490700	-0.15518300
S	-4.13838000	-0.11708200	0.63574200
C	-2.08918900	1.08497100	2.13386200
H	-1.19551800	1.71443900	2.15553600
H	-2.83977700	1.53807300	2.78391700
H	-1.84782300	0.09775800	2.52902200

C	-2.91689900	2.37982700	0.11402700
H	-3.66367000	2.89067400	0.72387400
H	-2.00299000	2.97990500	0.10937500
H	-3.30059800	2.30464300	-0.90391500
C	-4.14336300	-1.10134900	-2.02293500
H	-3.68110500	-1.74394400	-2.77847200
H	-5.18244000	-1.41383700	-1.90554000
H	-4.13745400	-0.07106800	-2.38053100
C	-3.42083300	-2.70927100	-0.19405300
H	-4.45057700	-3.03457500	-0.03428400
H	-2.97936000	-3.35974100	-0.95502400
H	-2.87721500	-2.83254400	0.74325800
C	-2.01732800	-0.74179900	-0.79972300
H	-1.29856800	-1.23512400	-1.45118800
H	-0.66734800	0.70145700	-0.24139600
C	4.01101500	0.31244700	-0.44921900
C	2.70504900	0.41800700	-0.06376800
C	2.04226100	-0.68305200	0.74829500
C	3.07237800	-1.66995100	1.24078000
C	4.34185500	-1.65749600	0.83167600
H	4.46403000	1.04018800	-1.11220500
H	1.49701600	-0.25331600	1.59812300
H	2.76642100	-2.42753700	1.95181800

H	5.07516800	-2.37462300	1.17605500
N	4.83293700	-0.69471100	-0.06876600
C	6.19544600	-0.82541500	-0.58121900
H	6.90357500	-0.91125300	0.24621000
H	6.28900000	-1.70595400	-1.22279700
H	6.44973700	0.05897700	-1.16394600
H	1.27897700	-1.20826000	0.15126200
C	1.88854000	1.54891800	-0.46964900
O	0.63468300	1.51812900	-0.40097500
N	2.47435700	2.68045600	-0.95143500
H	1.85973600	3.44210400	-1.19521000
H	3.43003900	2.91209500	-0.73912200

3-Thiazolin: transition state

Charge = +1, Multiplicity = singlet

C	-2.91341800	0.68597100	0.53329900
C	-1.71536000	-1.34184600	-0.83957700
N	-1.75931400	0.99102600	-0.32895100
S	-2.90025800	-1.18207500	0.59074500
C	-2.72135600	1.24687600	1.94818600
H	-2.68052800	2.34015800	1.91804800
H	-3.56330300	0.96418500	2.58370500
H	-1.80749600	0.86796800	2.40712800
C	-4.19959400	1.23951400	-0.09410100

H	-5.06125500	0.97864000	0.52290700
H	-4.14577800	2.33128000	-0.15733900
H	-4.36046900	0.84077500	-1.09495800
C	-2.45114600	-1.44317500	-2.19108700
H	-1.73071700	-1.53777200	-3.01048000
H	-3.08970300	-2.32845400	-2.19628300
H	-3.07461100	-0.57031900	-2.38298700
C	-0.82391700	-2.56989000	-0.63924500
H	-1.42271200	-3.48130400	-0.69289900
H	-0.07432800	-2.62544400	-1.43527100
H	-0.31706900	-2.55682700	0.32503400
C	-0.96830600	-0.01097300	-0.77688000
H	-0.34788000	0.25078300	-1.63723100
H	-1.40106700	1.93969200	-0.38274000
C	3.28711700	0.20407500	-0.27329800
C	2.13399300	0.85449100	0.07483700
C	1.13439300	0.16865900	0.88601900
C	1.62183500	-1.02997500	1.53964000
C	2.79156700	-1.60093600	1.16457900
H	4.00879100	0.64010200	-0.95225400
H	0.47417400	0.81549100	1.46297100
H	1.03675600	-1.49678900	2.32103300
H	3.16110100	-2.51709200	1.60582400

N	3.60754400	-1.02508600	0.21460200
C	4.81684900	-1.73293000	-0.23428100
H	5.34079700	-2.14512000	0.62837200
H	4.55341800	-2.54070200	-0.91995900
H	5.47590000	-1.03260900	-0.74398000
H	0.07800100	-0.17844000	0.06374700
C	1.81457100	2.22723900	-0.41940400
O	0.64593500	2.57724200	-0.55605700
N	2.85756200	3.04395300	-0.73271800
H	2.62567500	3.97895000	-1.03791700
H	3.77504700	2.90626300	-0.34057200

In addition, calculations utilizing the SMD intrinsic solvation model[16] were also performed, and parameters for water and chloroform were used. All geometries were reoptimized with the intrinsic solvation model using the functional and basis set mentioned above. Coordinates for the initial complexes of the starting materials and the transition states are given below.

Benzothiazin: complex of starting materials in water

Charge = +1, Multiplicity = singlet

C	3.14466400	0.43900400	-0.75892600
C	2.09039100	0.94171300	0.01930400
C	1.40635200	-1.19030200	0.91367800
C	2.70546700	-1.89649700	0.68005900
H	0.58521800	-1.73110200	1.37513000
N	1.19417200	0.05686600	0.66509200

S	3.36821600	-1.30693200	-0.95710200
C	3.66801600	-1.58165400	1.83850400
H	3.22383300	-1.91279900	2.78197100
H	4.60028300	-2.12870700	1.68207200
H	3.88900500	-0.51609700	1.91448500
C	2.47490400	-3.40628600	0.55322900
H	3.41916700	-3.91505000	0.34996800
H	2.07895800	-3.78751200	1.49776000
H	1.76596200	-3.64153700	-0.24276900
C	1.86380700	2.31618100	0.12281400
C	2.69117100	3.20020300	-0.55687300
C	3.73474100	2.70886200	-1.34464500
C	3.96358100	1.33952200	-1.44460900
H	1.04446200	2.67525100	0.73404200
H	2.52172200	4.26663800	-0.47555800
H	4.38090000	3.39523500	-1.87897900
H	4.78134300	0.96756000	-2.05036100
H	0.25194300	0.44106600	0.93983700
C	-4.36645000	0.09902500	0.31206900
C	-3.01792600	0.30462100	0.37266400
C	-2.09992600	-0.22600000	-0.71904000
C	-2.90413300	-0.72615000	-1.89326600
C	-4.23181600	-0.86523900	-1.85606100

H	-5.03495500	0.41245200	1.10368600
H	-1.40434300	0.55555900	-1.04767600
H	-2.37821800	-1.00291600	-2.79955500
H	-4.80952800	-1.24224400	-2.68996500
N	-4.98919200	-0.52682800	-0.72026300
C	-6.44785600	-0.60664400	-0.79564600
H	-6.85023700	0.15849800	-1.46676900
H	-6.74477600	-1.58999700	-1.16379600
H	-6.86917200	-0.46397900	0.19823500
H	-1.45989700	-1.03338300	-0.33351800
C	-2.39853400	0.98904800	1.49665000
O	-1.14165400	1.03371000	1.61614900
N	-3.15061500	1.58453500	2.45793200
H	-2.65971200	2.10840900	3.16810500
H	-4.12945400	1.78741300	2.32933200

Benzothiazin: transition state in water

Charge = +1, Multiplicity = singlet

C	2.91579600	-0.48530500	-0.41501500
C	2.52911600	0.78819900	0.03715000
C	0.44660400	0.04402300	1.09813100
C	0.90995600	-1.38044100	1.30576500
H	-0.33057600	0.39850300	1.77066300
N	1.32637700	0.98864400	0.73330800

S	1.86673000	-1.90575000	-0.20470400
C	1.79544300	-1.43872700	2.56330600
H	1.22690200	-1.08671600	3.42953500
H	2.09546300	-2.47352200	2.74294700
H	2.69239500	-0.82541000	2.46646800
C	-0.27810200	-2.33523600	1.44726200
H	0.07697500	-3.35522700	1.60972300
H	-0.87093300	-2.04166900	2.31777700
H	-0.92363700	-2.32564200	0.56798300
C	3.34706000	1.89727600	-0.21179200
C	4.53614300	1.74858600	-0.91299300
C	4.92342300	0.48551200	-1.36683200
C	4.12037800	-0.62223000	-1.11169700
H	3.03362200	2.87083200	0.14948500
H	5.15953400	2.61419200	-1.10192000
H	5.85201700	0.35969200	-1.91076700
H	4.42590000	-1.60364400	-1.45656100
H	0.99753900	1.94802800	0.80595100
C	-3.71174100	0.31071200	-0.09109200
C	-2.52368700	0.97685000	-0.22390800
C	-1.38358700	0.31122500	-0.88440400
C	-1.77530600	-0.86613900	-1.66743000
C	-2.98279400	-1.44398900	-1.50054100

H	-4.54740200	0.71318300	0.46555600
H	-0.69923000	0.99221400	-1.39514900
H	-1.07056400	-1.30788400	-2.35925800
H	-3.28521800	-2.33786300	-2.02988200
N	-3.93533400	-0.90608000	-0.65226400
C	-5.23995900	-1.57019500	-0.52811600
H	-5.80066000	-1.47968900	-1.46103900
H	-5.09111900	-2.62497000	-0.29739400
H	-5.80155500	-1.10436300	0.27868500
H	-0.58614200	-0.04955000	-0.01256800
C	-2.29822000	2.31666100	0.35872800
O	-1.13578900	2.74495900	0.51316800
N	-3.36403600	3.06489600	0.72086700
H	-3.19452700	3.97781000	1.11853100
H	-4.31233000	2.82113100	0.47863900

3-Thiazolin: complex of starting materials in water

Charge = +1, Multiplicity = singlet

C	-2.75647300	1.09139800	0.51958200
C	-3.19017700	-1.39850400	-0.55087700
N	-1.61779500	0.32750100	-0.04811500
S	-4.18005600	-0.11187700	0.37736700
C	-2.46544400	1.43094000	1.97958800
H	-1.60912400	2.10829100	2.04015200

H	-3.33127900	1.93629900	2.41170700
H	-2.25528500	0.53249600	2.56105800
C	-2.98752700	2.34525300	-0.32330700
H	-3.85343000	2.88444800	0.06496300
H	-2.11655900	3.00348100	-0.26044400
H	-3.16989300	2.09113800	-1.36826400
C	-3.67306200	-1.55819000	-2.00462200
H	-3.04290900	-2.28350400	-2.52692400
H	-4.69882900	-1.93227700	-2.00264900
H	-3.64308300	-0.60936500	-2.54192900
C	-3.20616900	-2.74832800	0.18564400
H	-4.23127300	-3.12181000	0.23406500
H	-2.60230000	-3.47595700	-0.36343600
H	-2.81651500	-2.65549200	1.20024200
C	-1.81497400	-0.82785900	-0.55000000
H	-0.97925300	-1.37193400	-0.98106700
H	-0.66447300	0.77535600	-0.04948000
C	3.97006300	0.31719000	-0.40219500
C	2.66824000	0.47465900	-0.02417400
C	1.92491500	-0.64189100	0.69406700
C	2.88543600	-1.72197100	1.12752300
C	4.15579300	-1.76914000	0.71768500
H	4.50172200	1.07621300	-0.96247100

H	1.38658800	-0.24657900	1.56362200
H	2.52224300	-2.50537100	1.78242500
H	4.84102000	-2.55436100	1.00935400
N	4.70734000	-0.79135500	-0.12962600
C	6.13863200	-0.84079300	-0.42816900
H	6.73580400	-0.58586500	0.45303200
H	6.41001300	-1.84386400	-0.76015400
H	6.36398300	-0.13573200	-1.22686500
H	1.14568700	-1.07160000	0.04748300
C	1.92802300	1.68940700	-0.32899900
O	0.68681700	1.76236600	-0.10985800
N	2.54986200	2.77040100	-0.86574500
H	2.00088000	3.60947800	-0.98465900
H	3.55310900	2.86877200	-0.86483700

3-Thiazolin: transition state in water

Charge = +1, Multiplicity = singlet

C	-2.91069500	0.68666700	0.48780300
C	-1.65981800	-1.36558600	-0.82561600
N	-1.75359600	0.97950500	-0.37515600
S	-2.95183200	-1.18491700	0.51300100
C	-2.68887600	1.22450400	1.90360600
H	-2.62911200	2.31698000	1.87463300
H	-3.52848000	0.94902400	2.54623100

H	-1.76868700	0.83024800	2.33626600
C	-4.18431600	1.27219700	-0.12580400
H	-5.04862700	1.02379800	0.49385100
H	-4.10100500	2.36279100	-0.17237800
H	-4.35064300	0.88827100	-1.13299700
C	-2.30593200	-1.52047200	-2.21601500
H	-1.52626600	-1.57436400	-2.98245900
H	-2.88260900	-2.44771600	-2.24856200
H	-2.96982000	-0.68804700	-2.45195200
C	-0.75594500	-2.56266900	-0.53228600
H	-1.32910700	-3.48967900	-0.60761500
H	0.04804400	-2.60677800	-1.27308600
H	-0.31412900	-2.50874100	0.46188800
C	-0.95023800	-0.02330300	-0.76472200
H	-0.24118800	0.20978200	-1.55927700
H	-1.35792000	1.91630800	-0.34360200
C	3.22375900	0.18270800	-0.29288300
C	2.10046300	0.87262600	0.08537300
C	1.09931700	0.21255200	0.93355500
C	1.61187600	-0.96812500	1.62422500
C	2.75092600	-1.56937500	1.21961500
H	3.93825000	0.57805300	-1.00234600
H	0.48984800	0.88496000	1.53806300

H	1.04959100	-1.39892400	2.44196800
H	3.13432200	-2.47313600	1.67358600
N	3.52760200	-1.04048400	0.20531800
C	4.71068900	-1.78171500	-0.25270400
H	5.33174700	-2.04565200	0.60413900
H	4.40394000	-2.69182200	-0.77064500
H	5.28222200	-1.15517200	-0.93373300
H	0.11825700	-0.16156100	0.18724500
C	1.82802000	2.24680000	-0.39052300
O	0.67311200	2.71627200	-0.34334900
N	2.85452300	2.98071600	-0.87480900
H	2.66135200	3.92218300	-1.18657200
H	3.81935700	2.70867700	-0.76307700

Benzothiazin: complex of starting materials in chloroform

Charge = +1, Multiplicity = singlet

C	3.05439700	0.66443300	-0.66161200
C	2.00874200	0.88861800	0.24892300
C	1.52788000	-1.43786100	0.64394200
C	2.86520300	-1.96355600	0.21680100
H	0.76664700	-2.13613500	0.98207100
N	1.21205700	-0.18905200	0.69868700
S	3.38952300	-0.96665300	-1.26691900
C	3.86573100	-1.82918600	1.37797600

H	3.51184500	-2.39823400	2.24340600
H	4.82917900	-2.23873800	1.06650000
H	4.00886900	-0.79136900	1.68206700
C	2.74572900	-3.42244800	-0.24125900
H	3.71302000	-3.78804600	-0.59181300
H	2.43739300	-4.04463100	0.60314300
H	2.01601100	-3.53779500	-1.04547400
C	1.68963400	2.18021000	0.67791900
C	2.41402700	3.26032000	0.19326200
C	3.44587500	3.04878600	-0.72451700
C	3.76760500	1.76271400	-1.14852000
H	0.88120500	2.32043600	1.38600500
H	2.17578800	4.26283200	0.52667300
H	4.01154700	3.89031900	-1.10715300
H	4.57686500	1.60891600	-1.85265800
H	0.24955200	0.04577300	1.08170500
C	-4.31497700	-0.16575100	0.33912000
C	-2.99632000	0.17392200	0.43927100
C	-2.13503400	0.34687900	-0.80369400
C	-2.98600400	0.29078700	-2.04907400
C	-4.28512300	-0.01785600	-2.03075200
H	-4.91525800	-0.37275000	1.21735500
H	-1.59505000	1.30162800	-0.76373500

H	-2.51827100	0.50467600	-3.00313300
H	-4.88940200	-0.06627600	-2.92761400
N	-4.97682300	-0.28967000	-0.83852800
C	-6.35528800	-0.76702700	-0.90455900
H	-6.94899700	-0.10867500	-1.54293500
H	-6.40073700	-1.78468300	-1.30479800
H	-6.78622800	-0.76279100	0.09601200
H	-1.35138200	-0.42321600	-0.85434400
C	-2.35868100	0.32905800	1.73878100
O	-1.10734100	0.32561400	1.86510800
N	-3.11617900	0.46935100	2.85959300
H	-2.62561200	0.61480500	3.73030000
H	-4.07437600	0.77884000	2.81794500

Benzothiazin: transition state in chloroform

Charge = +1, Multiplicity = singlet

C	2.90349200	-0.43313600	-0.47467300
C	2.46984200	0.79276500	0.05983000
C	0.43887200	-0.10829100	1.11281000
C	0.98184100	-1.51829200	1.22890300
H	-0.31194500	0.17625700	1.84780600
N	1.27246700	0.89699000	0.78347200
S	1.90903400	-1.90338300	-0.34136200
C	1.91033200	-1.60387100	2.45336800

H	1.35370400	-1.34998800	3.36134300
H	2.28523200	-2.62496200	2.55358000
H	2.76259900	-0.92762100	2.37513400
C	-0.15213200	-2.54044900	1.34829100
H	0.25504100	-3.54898200	1.44859000
H	-0.73955900	-2.32784300	2.24626200
H	-0.81890400	-2.52160500	0.48454700
C	3.24156100	1.94736300	-0.12420500
C	4.42756200	1.88987600	-0.84351300
C	4.86066600	0.67438200	-1.37776100
C	4.10578800	-0.47902600	-1.18598100
H	2.89802000	2.88363900	0.30329500
H	5.01516600	2.78956200	-0.98221600
H	5.78889200	0.62113200	-1.93434300
H	4.44801800	-1.42515300	-1.58988800
H	0.88294300	1.82998700	0.90475100
C	-3.72886800	0.39088500	-0.05880700
C	-2.49778100	0.97848600	-0.17033400
C	-1.39782300	0.25420100	-0.82804100
C	-1.85004400	-0.88759600	-1.62262200
C	-3.09595500	-1.38979800	-1.47758600
H	-4.53643100	0.84823400	0.49912200
H	-0.65716900	0.89494800	-1.31269400

H	-1.16766700	-1.36448600	-2.31377300
H	-3.44427900	-2.25914500	-2.01991100
N	-4.02666000	-0.79868900	-0.64470200
C	-5.34143300	-1.43039300	-0.47635000
H	-5.80871900	-1.57960500	-1.45152300
H	-5.23181500	-2.39432200	0.02431900
H	-5.97559700	-0.78603900	0.12912500
H	-0.59802900	-0.16669500	0.05956100
C	-2.20102100	2.29982700	0.43977800
O	-1.04335600	2.59700500	0.76390700
N	-3.23217100	3.15587600	0.64390400
H	-3.02086600	4.06154800	1.03962500
H	-4.12118400	3.04839800	0.18046100

3-Thiazolin: complex of starting materials in chloroform

Charge = +1, Multiplicity = singlet

C	-2.61486300	0.87642800	0.86917900
C	-3.23283000	-1.19332300	-0.82950100
N	-1.61566300	0.42558800	-0.13173300
S	-4.05407700	-0.28362100	0.58189400
C	-2.03559500	0.70987300	2.27392200
H	-1.18529300	1.38378700	2.41236900
H	-2.79643000	0.97100300	3.01208200
H	-1.71654300	-0.31737800	2.45408900

C	-2.99503100	2.32901300	0.57733400
H	-3.77386600	2.64550000	1.27385700
H	-2.12737400	2.98086000	0.71551500
H	-3.37035400	2.44455800	-0.44030700
C	-4.00993700	-1.01739700	-2.14740600
H	-3.49775500	-1.53949800	-2.96104100
H	-5.00576700	-1.45263900	-2.03922200
H	-4.11955200	0.03453500	-2.41484300
C	-3.04564000	-2.68600800	-0.49931600
H	-4.02504200	-3.15173300	-0.37001700
H	-2.53374300	-3.19411600	-1.32196700
H	-2.47216200	-2.82623200	0.41825000
C	-1.90361500	-0.52629800	-0.92789400
H	-1.17036200	-0.83813400	-1.66758400
H	-0.67163000	0.91134000	-0.19185400
C	3.90085700	0.23972000	-0.49048800
C	2.62844700	0.51477300	-0.07813100
C	1.90438100	-0.41078000	0.88928100
C	2.85655700	-1.43734800	1.45258600
C	4.10206200	-1.59886200	0.99890000
H	4.39681900	0.84130000	-1.24245100
H	1.45259100	0.16865700	1.70411300
H	2.51111800	-2.07814900	2.25557300

H	4.77939600	-2.34489700	1.39472500
N	4.64060300	-0.79559500	-0.01975300
C	5.95420700	-1.12563100	-0.56634900
H	6.68710500	-1.20991600	0.23986600
H	5.92142600	-2.07123600	-1.11595400
H	6.27287000	-0.33670500	-1.24623200
H	1.06081300	-0.91726100	0.39659000
C	1.89754900	1.66472100	-0.59295300
O	0.65056200	1.76059800	-0.46402800
N	2.55485200	2.66169000	-1.24586400
H	2.00382200	3.45653100	-1.53741100
H	3.54281400	2.81787900	-1.11975600

3-Thiazolin: transition state in chloroform

Charge = +1, Multiplicity = singlet

C	-2.89238400	0.69041200	0.53541800
C	-1.70869400	-1.35155400	-0.84917300
N	-1.73901300	0.98063400	-0.33011700
S	-2.94225800	-1.18106500	0.54288700
C	-2.66435200	1.20799400	1.95931100
H	-2.59019800	2.30019500	1.95017400
H	-3.50638200	0.93474600	2.60004200
H	-1.75166100	0.79432400	2.39003500
C	-4.16129800	1.29902000	-0.06958300

H	-5.03191500	1.04628700	0.53953900
H	-4.07035000	2.38996300	-0.09705800
H	-4.32737500	0.93774900	-1.08486600
C	-2.41354500	-1.45456100	-2.21616800
H	-1.67166000	-1.50841100	-3.01998000
H	-3.01748200	-2.36427100	-2.24936000
H	-3.06624300	-0.60119400	-2.40413900
C	-0.82463100	-2.57758200	-0.62034300
H	-1.42345300	-3.48943700	-0.68037500
H	-0.05833400	-2.63618600	-1.40000400
H	-0.33453200	-2.55489000	0.35255800
C	-0.96371100	-0.02501700	-0.77482100
H	-0.29750500	0.22444000	-1.60210800
H	-1.34427300	1.91836800	-0.33473100
C	3.26337900	0.19782900	-0.27809900
C	2.11153400	0.85371500	0.06944200
C	1.11109500	0.17183900	0.89477000
C	1.62379300	-1.00722600	1.57910600
C	2.79040200	-1.57874500	1.20260400
H	3.98348700	0.61736800	-0.96888300
H	0.47089300	0.83017200	1.48374200
H	1.05066000	-1.45978900	2.37766700
H	3.17762300	-2.47980800	1.65928900

N	3.58814500	-1.02004800	0.22586900
C	4.77876600	-1.74304100	-0.24020000
H	5.28751800	-2.19047200	0.61390700
H	4.49357200	-2.52789500	-0.94422100
H	5.45380100	-1.04537800	-0.73257600
H	0.11345100	-0.20210100	0.11663000
C	1.80648700	2.22490500	-0.41910000
O	0.63918800	2.62971400	-0.46649600
N	2.84366100	3.00123500	-0.82142800
H	2.63537500	3.94516000	-1.11700300
H	3.79699900	2.79971100	-0.56176900

Negative controls for biotranformation of 3-thiazolines

Negative controls were performed on 10 mL scale at 30 °C and 500 rpm in 100 mM KP_i buffer pH 7, with 2% methanol as cosolvent containing 40 mM D-glucose, 20 mM 3-thiazoline **1a-f**, 100 U of GDH and 0.1 mM $NADP^+$. After 24 h, the reaction was stopped by adding 200 µL of 32% NaOH solution and 10 mL of dichloromethane. Phase separation was promoted by centrifugation and the conversion was determined by analyzing the organic phase by means of achiral GC (Supplementary Table 5 and Supplementary Methods).

Biotransformations of *2H*-1,4-benzothiazines

Biotransformations of *2H*-1,4-benzothiazines were performed on 0.5 mL scale at 30 °C and 850 rpm in 100 mM KP_i buffer pH 7, with 4% methanol (in case of **3a** and **3b**) or dimethylsulfoxide (in case of **3c**) as cosolvent containing 40 mM D-glucose, 20 mM *2H*-1,4-benzothiazine **3a-c**, 0.2 mg mL^{-1} (in case of substrate **3b**) or 0.6 mg mL^{-1} (in case of substrate **3a** and **3c**) IRED crude extract, 6 U (in case of 0.2 mg mL^{-1} IRED), 12 U (in case of 0.6 mg mL^{-1} IRED) of GDH and 0.1 mM $NADP^+$. After 4, 6 or 8 h, the

reaction was stopped by adding 10 µL of 32% NaOH solution and 300 µL of dichloromethane. Phase separation was promoted by centrifugation and the conversion was determined by analyzing the organic phase by SFC-HPLC (Supplementary Figures 93-95, Supplementary Table 7 and Supplementary Methods; synthesis of racemic 3-3,4-dihydro-2*H*-1,4-benzothiazine reference compounds is described in Supplementary Methods and related NMR data are shown in Supplementary Figures 96-101). The results of these experiments are shown in Table 2 (main manuscript).

Negative controls were performed on 0.5 mL scale at 30 °C and 850 rpm in 100 mM KP_i buffer pH 7, with 4% methanol (in case of **3a** and **3b**) or dimethylsulfoxide (in case of **3c**) as cosolvent containing 40 mM D-glucose, 20 mM *2H*-1,4-benzothiazine **3a-c**, 12 U of GDH and 0.1 mM $NADP^+$. After 4, 6 or 8 h, the reaction was stopped by adding 10 µL of 32% NaOH solution and 300 µL of dichloromethane. Phase separation was promoted by centrifugation and the conversion was determined by analyzing the organic phase by means of LC2000 SFC-HPLC system from Jasco (Easton, USA) (Supplementary Table 7 and Supplementary Methods).

Determination of the absolute configuration of (*S*)-2f

The absolute configuration of (*S*)-2,2,3-Trimethyl-1-thia-4-azaspiro[4.4]nonane ((*S*)-**2f**) was determined by vibrational circular dichroism (VCD) spectroscopy. The IR and VCD spectra were recorded for a 0.3 M solution of (*S*)-**2f** in $CDCl_3$ at a pathlength of 100 µm over the course of 8 hrs accumation time (~35000 scans). The VCD baseline was corrected by subtraction of the spectrum of the racemic mixture **2f** recorded under identical condition. The experimentally obtained spectra are shown in Figure 4 (main manuscript).

In order to determine the absolute configuration, a conformational analysis was carried out for (S)-**2f** at the MMFF level of theory using Spartan 14 (Spartan 14, Wavefunction Inc., Irvine, CA, USA (2014)). Subsequently, all eight obtained conformers were subjected to further geometry optimizations followed by spectra calculations at the B3LYP/6-311g++(2d,p)/IEFPCM($CHCl_3$) level of theory (Gaussian 09 Rev. E01, Frisch, M.J. *et al.* Gaussian, Inc., Wallingford CT, USA, (2013)). The relative Gibbs free energies ΔG_{298K} and the corresponding Boltzmann weights are of the two populated conformers are shown in Figure 4 (main manuscript). Finally, the IR and VCD spectra were simulated by assigning a Lorentzian band shape to the dipole and rotational strength calculated for each conformer and subsequent Boltzmann-averaging of the spectra. Direct comparison of the resulting simulated IR and VCD spectra

with the experimental data, as indicated by the assignments given in Figure 4 (main manuscript), reveals a very good agreement. Therefore, the absolute configuration can with very high confidence be assigned as (*S*)-**2f**.

Construction and preparation of whole cell-catalyst

Escherichia coli strain BL21(DE3), which was used for expression, and pACYCDuet-1 vector were purchased from Novagen (Madison, USA). The whole-cell catalyst was constructed as a two-plasmid-system, harbouring the gene for the glucose dehydrogenase from *Bacillus subtilis* in a pACYCDuet-1 vector[17,18] and the gene for the imine reductase from *Mycobacterium smegmatis* in the commercially available pET-22b(+) vector.[1] A preculture of *E. coli* BL21(DE3) carrying the two recombinant plasmids was cultivated over-night at 37 °C in 10 mL LB medium, containing 80 µg mL^{-1} of carbenicillin and 28 µg mL^{-1} of chloramphenicol. The main culture containing 600 mL TB medium, 80 µg mL^{-1} of carbenicillin and 28 µg mL^{-1} of chloramphenicol, was inoculated with the starting culture to a final concentration of 1%. At an OD_{600} between 0.4 und 0.6, the production of recombinant protein was induced by addition of isopropyl-thio-β-D-galactoside (IPTG) to a final concentration of 0.5 mM. Cultures were shaken at 25 °C for 20 h and harvested by centrifugation. For lyophilization of the cells a 50% cell suspension in water was used, and the resulting lyophilized cells were stored in a freezer at -20 °C.

Preparative scale biotransformation

The biotransformation on preparative scale (40 mL) was performed starting from a 100 mM concentration of 2,2,3-trimethyl-1-thia-4-azaspiro[4.4]non-3-ene (**1f**), 10 mg mL^{-1} of lyophilized whole-cell catalyst (prepared from 20 mg mL^{-1} of cell suspension in 50 mM KPi buffer pH 7, construction of the whole-cell catalyst is described in the Supplementary Information), 240 mM of D-glucose, 0.1 mM of $NADP^+$ and 2% of MeOH as cosolvent in distilled water. The flask was equipped with the titration device and pH electrode of a pH stat apparatus and stirred at 30 °C. By addition of aqueous NaOH solution, the pH was kept stable at 7. The reaction was stopped by adding 2 mL of 32% NaOH solution and 30 mL of dichloromethane. Phase separation was promoted by centrifugation. The organic phase was dried over magnesium sulfate and the conversion was determined by analyzing the organic phase by means of achiral GC (Supplementary Figures 35-39, Supplementary Table 3 and Supplementary Methods). The solvent was evaporated *in vacuo* and part of the product was derivatized according to General

Procedure 5 (Supplementary Methods) and then analyzed by chiral SFC-HPLC (Supplementary Table 4 and Supplementary Methods). For isolation of 2,2,3-trimethyl-1-thia-4-azaspiro[4.4]nonane (**2f**) the crude product was dissolved in dichloromethane and was washed with dH_2O (2 x 30 mL) and brine (30 mL). The organic phase was dried over magnesium sulfate and the solvent was evaporated *in vacuo*. (*S*)-2,2,3-Trimethyl-1-thia-4-azaspiro[4.4]nonane ((*S*)-**2f**) (578.5 mg, 3,12 mmol, 78%) was obtained as a yellowish oil with an isolated yield of 78%, a purity of 97% (determined by ^{1}H NMR spectroscopy) and 99% *ee*. The result of this experiment is shown in Figure 7 (main manuscript).

^{1}H-NMR (500 MHz, $CDCl_3$): δ (ppm) = 3.10 (q, *J* = 6.60 Hz, 1°H, C3-**H**), 2.16–1.64 (m, 8 H, (C$\mathbf{H_2}$)$_4$), 1.41 (s, 3 H, C2-C$\mathbf{H_3}$), 1.19 (s, 3 H, C2-C$\mathbf{H_3}$), 1.09 (d, *J* = 6.61 Hz 3 H, C3-C$\mathbf{H_3}$).

^{13}C-NMR (126 MHz, $CDCl_3$): δ (ppm) = 81.5 (**C**5), 67.1 (**C**3), 59.9 (**C**2), 44.8, 42.4, 24.4, 23.9 (**C**H_2)$_4$), 27.8 (C2-**C**H_3), 26.1 (C2-**C**H_3), 13.7 (C3-**C**H_3).

HRMS (ESI) m/z calculated for $C_{10}H_{20}NS$ $[M+H]^+$: 186.13110, found: 186.13090.

The analytical data corresponds with literature data.[7] ^{1}H and ^{13}C NMR spectra of (*S*)-**2f** are shown in Supplementary Figure 83 and 84.

Supplementary References

1. Wetzl, D. *et al*. Expanding the imine reductase toolbox by exploring the bacterial protein-sequence space. *ChemBioChem* **16**, 1749-1756 (2015).

2. Wetzl, D. *et al*. Asymmetric reductive amination of ketones catalyzed by imine reductases. *ChemCatChem* **8**, 2023-2026 (2016).

3. Huber,T. *et al.* Direct reductive amination of ketones: structure and activity of (*S*)-selective imine reductases from *Streptomyces. ChemCatChem* **6**, 2248-2252 (2014).

4. Rodig, M.J., Snow, A.W., Scholl, P. & Rea, S. Synthesis and low temperature spectroscopic observation of 1,3,5-trioxane-2,4,6-trione: the cyclic trimer of carbon dioxide. *J. Org. Chem.* **81**, 5354-5361 (2016).

5. Jobin-Des Lauriers, A. & Legault, C.Y. Iodine(III)-mediated oxidative hydrolysis of haloalkenes: access to α-halo ketones by a release-and catch mechanism. *Org. Letters* **18**, 108-111 (2016).

6. Martens, J., Offermanns, H. & Scherberich, P. Facile synthesis of racemic cysteine. *Angew. Chem. Int. Ed. Engl.* **20**, 668 (1981).

7. Reiners, I., Gröger, H. & Martens, J. A new enantioselective synthetic approach to β-aminothio-compounds via enantioselective reduction of N,S-heterocyclic imines. *J. Prakt. Chem.* **339**, 541-546 (1997).

8. Brockmeyer, F., van Gerven, D., Saak, W. & Martens, J. Two sequential multicomponent reactions: synthesis of thiazolidin-4-yl-1,3,4-oxadiazoles under mild conditions. *Synthesis* **46**, 1603-1612 (2014).

9. Stalling, T., Johannes, K., Polina, S. & Martens, J. Stereospecific synthesis of β-lactams from heterocyclic imines using the staudinger reaction. *J. Heterocyclic Chem.* **50**, 654-659 (2013).

10. Rueping, M., Azap, C., Sugiono, E. & Theissmann, T. Brønsted acid catalysis: organocatalytic hydrogenation of imines. *Synlett* **15**, 2367-2369 (2005).

11. Shimizu, H., Ueda, N., Kataoka, T. & Hori, M. Non-stereospecific ring expansion reactions of benzothiazoline sulfoxides. *Chem. Pharm. Bull.* **32**, 2571-2590 (1984).

12. Pick, A. *et al.* Identification and characterization of two new 5-keto-4-deoxy-D-glucarate dehydratases/decarboxylases. *BMC Biotechnol.* **16**, 80-89 (2016).

13. Roth, S. *et al.* Extended catalytic scope of a well-known enzyme: asymmetric reduction of iminium substrates by glucose dehydrogenase. *ChemBioChem* **18**, 1703-1706 (2017).

14. Becke, A.D. Density-functional thermochemistry. III. The role of exact exchange. *Chem. Phys.* **98**, 5648-5652 (1993).

15. Lee, C., Yang, W. & Parr, R.G. Development of the Colle-Salvetti correlation-energy formula into a functional of the electron density. *Phys. Rev. B* **37**, 785-789 (1988).

16. Marenich, A.V., Cramer, C.J. & Truhlar, D.G. Universal solvation model based on solute electron density and on a continuum model of the solvent defined by the bulk dielectric constant and atomic surface tensions. *J. Phys. Chem. B* **113,** 6378–6396 (2009).

17. Zumbrägel, N., Wetzl, D., Iding, H. & Gröger, H. Asymmetric biocatalytic reduction of cyclic imines: design and application of a tailor-made whole-cell catalyst. *Heterocycles* **95,** 1261-1271 (2017).

18. Biermann, M., Bakonyi, D., Hummel, W. & Gröger, H. Design of recombinant whole-cell catalysts for double reduction of C=C and C=O bonds in enals and application in the synthesis of Guerbet alcohols as industrial bulk chemicals for lubricants. *Green Chem.* **19**, 405-410 (2017).
